# Proteomic Analysis of Pathways Involved in Estrogen-Induced Growth and Apoptosis of Breast Cancer Cells

**DOI:** 10.1371/journal.pone.0020410

**Published:** 2011-06-27

**Authors:** Zhang-Zhi Hu, Benjamin L. Kagan, Eric A. Ariazi, Dean S. Rosenthal, Lihua Zhang, Jordan V. Li, Hongzhan Huang, Cathy Wu, V. Craig Jordan, Anna T. Riegel, Anton Wellstein

**Affiliations:** 1 Lombardi Cancer Center, Georgetown University, Washington, D.C., United States of America; 2 Protein Information Resource, Georgetown University, Washington, D.C., United States of America; 3 Fox Chase Cancer Center, Philadelphia, Pennsylvania, United States of America; The University of Kansas Medical Center, United States of America

## Abstract

**Background:**

Estrogen is a known growth promoter for estrogen receptor (ER)-positive breast cancer cells. Paradoxically, in breast cancer cells that have been chronically deprived of estrogen stimulation, re-introduction of the hormone can induce apoptosis.

**Methodology/Principal Findings:**

Here, we sought to identify signaling networks that are triggered by estradiol (E2) in isogenic MCF-7 breast cancer cells that undergo apoptosis (MCF-7:5C) versus cells that proliferate upon exposure to E2 (MCF-7). The nuclear receptor co-activator AIB1 (Amplified in Breast Cancer-1) is known to be rate-limiting for E2-induced cell survival responses in MCF-7 cells and was found here to also be required for the induction of apoptosis by E2 in the MCF-7:5C cells. Proteins that interact with AIB1 as well as complexes that contain tyrosine phosphorylated proteins were isolated by immunoprecipitation and identified by mass spectrometry (MS) at baseline and after a brief exposure to E2 for two hours. Bioinformatic network analyses of the identified protein interactions were then used to analyze E2 signaling pathways that trigger apoptosis versus survival. Comparison of MS data with a computationally-predicted AIB1 interaction network showed that 26 proteins identified in this study are within this network, and are involved in signal transduction, transcription, cell cycle regulation and protein degradation.

**Conclusions:**

G-protein-coupled receptors, PI3 kinase, Wnt and Notch signaling pathways were most strongly associated with E2-induced proliferation or apoptosis and are integrated here into a global AIB1 signaling network that controls qualitatively distinct responses to estrogen.

## Introduction

Estrogen induces proliferation of estrogen receptor (ER)-positive breast cancer cells [1]. This response is consistent with the finding that antihormone therapies, such as tamoxifen or aromatase inhibitors, can enhance survivorship and reduce recurrence in patients with ER-positive breast cancers [2], [3]. However, the majority of tumors eventually become unresponsive to antihormone treatments [4], [5] and molecular mechanisms and markers of antihormone resistance have been described [6], [7]. Once patients have failed on antihormone therapy, one treatment option has been the use of pharmacologic doses of estrogens [8], [9] based on well-established findings that some breast cancers shrink during high dose estrogen treatment [10], [11], [12]. This phenomenon has also been observed in laboratory models of ER-positive breast cancer with acquired anti-hormone resistance that regress and undergo apoptosis in the presence of physiologic concentrations of estrogen [13], [14] and was reviewed recently for its potential clinical implications [15].

Estrogen exerts diverse effects including genomic and non-genomic effects through multiple signaling pathways, that are significantly altered in anti-hormone resistant ER positive breast cancer cells. In antihormone resistant cells, for example, there is a general increase in EGFR and IGFR tyrosine kinase signaling [16], [17], accompanied by increased ligand-independent phosphorylation of ER [18] and nuclear receptor co-activators such as AIB1/SRC3 (Amplified in Breast Cancer 1/Steroid Receptor Co-activator3) [19]. Overexpression and activation of AIB1 is associated with endocrine resistance in human breast cancer [20], [21], [22] and has been shown to be rate-limiting for estrogen-induced growth of breast cancer cells [23], [24]. Beyond its role in these effects of estrogen, AIB1 was also shown to be rate-limiting for the growth of estrogen-insensitive breast cancer cells [25] as well as prostate cancer [26], pancreatic cancer [27] and lymphoma cells [28]. Furthermore, in AIB1 knockout mice, responses to hormones [29] as well as growth factor signaling [30] are blunted whereas overexpression of an AIB1 transgene leads to increased estrogen and growth factor responses resulting in hyperplasia and neoplasia of mammary glands [31], [32], [33]. Thus, a large body of data support a crucial role for AIB1 in estrogen and growth factor signaling (reviewed in Refs [34], [35]) and provides the rationale for the experimental paradigm used here.

To identify pathways that initiate estrogen-induced apoptosis versus growth, we used a combined proteomics and systems biology approach to elucidate triggering events and associated signaling pathways. We focused on changes of AIB1 interacting proteins, because of its central role in estrogen control of phenotypic behavior of breast cancer cells outlined above. AIB1 also coactivates IGF1R, EGFR and HER2 through modulation of tyrosine phosphorylation of these transmembrane receptors and phosphorylation of their subsequent signaling intermediaries [27], [30], [33], [34]. Thus, to complement the analysis of direct AIB1 interacting proteins, we also monitored changes of phosphotyrosine (pY)-containing protein complexes, that are most likely regulated by growth factor signaling, as a means of discovering global intersecting pathways. As a model system, we used MCF-7 cells that proliferate in response to E2 [1], but also respond to EGF and heregulin [36] and have high levels of AIB1 protein due to gene amplification [37]. Wild-type MCF-7 cells were compared with MCF-7:5C cells that had been isolated under estrogen-free growth conditions [38], [39]. MCF-7:5C cells were derived following long-term culture of MCF-7 cells in phenol red-free media. MCF-7:5C cells are ER-positive and undergo apoptosis after exposure to physiological concentrations of E2. In contrast, wild-type parental MCF-7 cells proliferate in the presence of the same concentration range of E2 [38], [39]. The MCF-7:5C cells represent many of the characteristics of Phase II SERM resistant cells [40]. A parallel analysis after estrogen stimulation of these isogenic breast cancer cell lines served as a basis for the comparisons of signaling responses.

**Figure 1:**
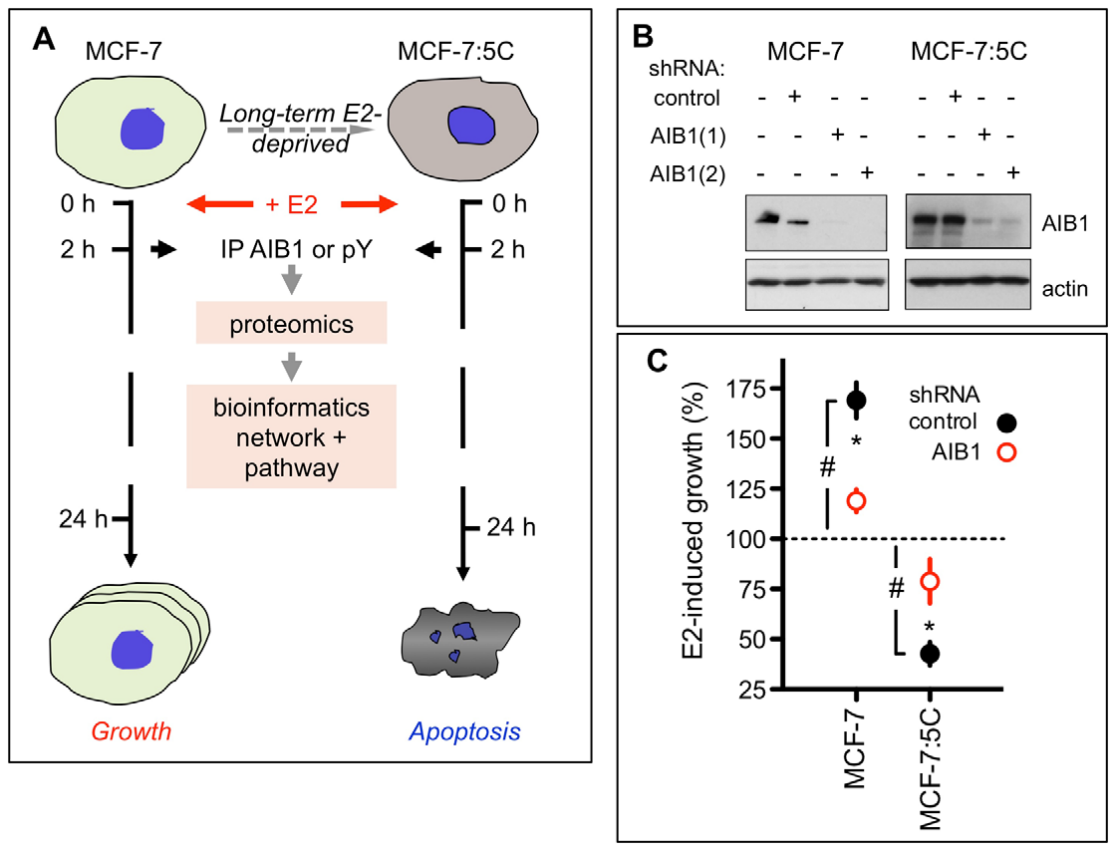
Phenotypic impact of AIB1 depletion on estradiol (E2) growth response in MCF-7 or MCF-7:5C cells. (*A*) The experimental paradigm. The differential responses to estradiol (E2) treatment of MCF-7 (cell growth) and long-term estrogen deprived MCF-7:5C cells (apoptosis) are indicated. Proteomics profiles of the two cell lines at baseline and after a brief (2 h) E2 treatment were generated using immunoprecipitations (IP). Proteins interacting with AIB1 or phosphotyrosine containing protein complexes were isolated by IP followed by mass spectrometry. Data were then subjected to an integrated bioinformatics analysis of signaling pathways and protein networks. (*B,C*) Reversal of E2-dependent effects on MCF-7 and MCF-7:5C after depletion of endogenous AIB1 protein using two different lentiviral shRNAs. MCF-7 or MCF-7:5C cells were infected with lentiviral particles expressing control or AIB1-targeting shRNAs. *(B)* RNAi-mediated knockdown was assayed by Western blot analysis for AIB1 relative to an actin loading control. *(C)* Cell growth was assayed 6 days after plating without or with E2. The E2 effect is shown relative to the respective untreated controls (mean ±S.E.M.). Closed circles: control shRNA; Open circles (red): AIB1 shRNA. #, p<0.05 E2 treatment effect vs. no treatment in control shRNA cells; *, p<0.05 E2 treatment effect in control shRNA cells vs. E2 treatment in AIB1 depleted cells. Representative data from one of at least three independent experiments are shown.

Here, we show that RNAi-mediated depletion of AIB1 reduces E2-induced growth of MCF-7 cells, and reverses the estrogen-induced apoptosis in MCF-7:5C cells. AIB1-interacting and pY-containing protein complexes were immunoprecipitated from short-term E2-treated cells, and the complexed proteins were identified by mass spectrometry (MS) analysis (Fig. 1A). From a comparison of the data sets obtained with MCF-7 versus MCF-7:5C cells treated with or without E2, and from a computationally-derived global AIB1-interacting network prediction, we identified pathways that participate in the differential response to E2 in these breast cancer cells. We found that a limited number of major cellular signaling pathways i.e. GPCR, PI3 kinase, Wnt, Notch and their associated molecules were involved in the control of estrogen induced proliferative or apoptotic responses. This information will be useful for determining appropriate targets to induce apoptosis in endocrine resistant human breast cancer.

## Results and Discussion

### Impact of AIB1 depletion on E2-induced growth effects in MCF-7 and MCF-7:5C cells

To determine the role of AIB1 in the E2-induced, distinct growth phenotypes of MCF-7:5C and wild-type MCF-7 cells, both cell lines were infected with lentiviral vectors that express control or two distinct AIB1-targeted shRNAs, and selected in puromycin for stable integrants. Both MCF-7 and MCF-7:5C cells were depleted of AIB1 protein, compared to uninfected and control shRNA infected cells with either of the shRNAs (Fig. 1B). Treatment with E2 significantly induced growth of control shRNA-infected MCF-7 cells and reduced the growth of MCF-7:5C cells (Fig. 1C, black symbols). In contrast to this, in AIB1-depleted, wild-type MCF-7 cells, E2 did not stimulate growth significantly above baseline and in AIB1 depleted MCF-7:5C, E2 lost its apoptosis-inducing effect (Fig. 1C, red symbols). These data suggest that AIB1 is a significant control hub of the E2-controlled growth phenotype in these ER-positive breast cancer cells.

### Global analysis of AIB1- and phosphotyrosine-complexed proteins

Because AIB1 is rate-limiting for the E2-induced changes in the growth phenotype of MCF-7 and MCF-7:5C cells, we performed AIB1-specific immunoprecipitations of lysates from untreated and E2-treated (2 hrs) MCF-7 and MCF-7:5C cells to fractionate the respective proteome. Immunoprecipitation of phosphotyrosine-containing protein complexes was also performed to complement the AIB1-specific proteome fractionation (Fig. 1A). The immunoprecipitates were released from the beads, separated by denaturing gel electrophoreses (SDS-PAGE) and followed by Coomassie Blue staining of proteins in the gels (Fig. S7). Visible bands and the same region in parallel gel lanes were harvested and proteins present identified by mass spectrometry (MS). Stringent filtering of the initial proteomic data resulted in a subset of 101 proteins that either interacted with AIB1 (n = 58, Table S1) or are present in pY-protein complexes (n = 56, Table S2), with 13 proteins common to both.

The analytical approach emphasizes reliable identification of proteins by correlating mass spectrometry ID with the apparent molecular mass obtained from the SDS-PAGE (Fig. S7). This approach mimics Western blotting without having to rely on the availablility of antibodies, appropriate sensitivity, suitability for Western blotting and specificity. Still, we used Western blotting of some proteins identified by MS and show two examples in Fig. S8 (see below). To validate the mass spectrometry findings, separate experiments with independent mass spectrometry analyses were run. We found 48% of the proteins reported here in two and 16% in three or more independent experiments. This compares favorably with a recent HUPO study where only 7 of 27 laboratories identified all 20 proteins present at equimolar concentrations in a test sample [41]. In our experiments, the abundance of individual endogenous proteins captured in the immunoprecipitates covers a wide range (see Fig. S7). Thus, we expected that lower abundance proteins may drop below detection in repeat experiments. A combination of bioinformatics and mass spectrometry analysis was thus applied to meet this challenge as also described elsewhere [42], [43].

**Figure 2:**
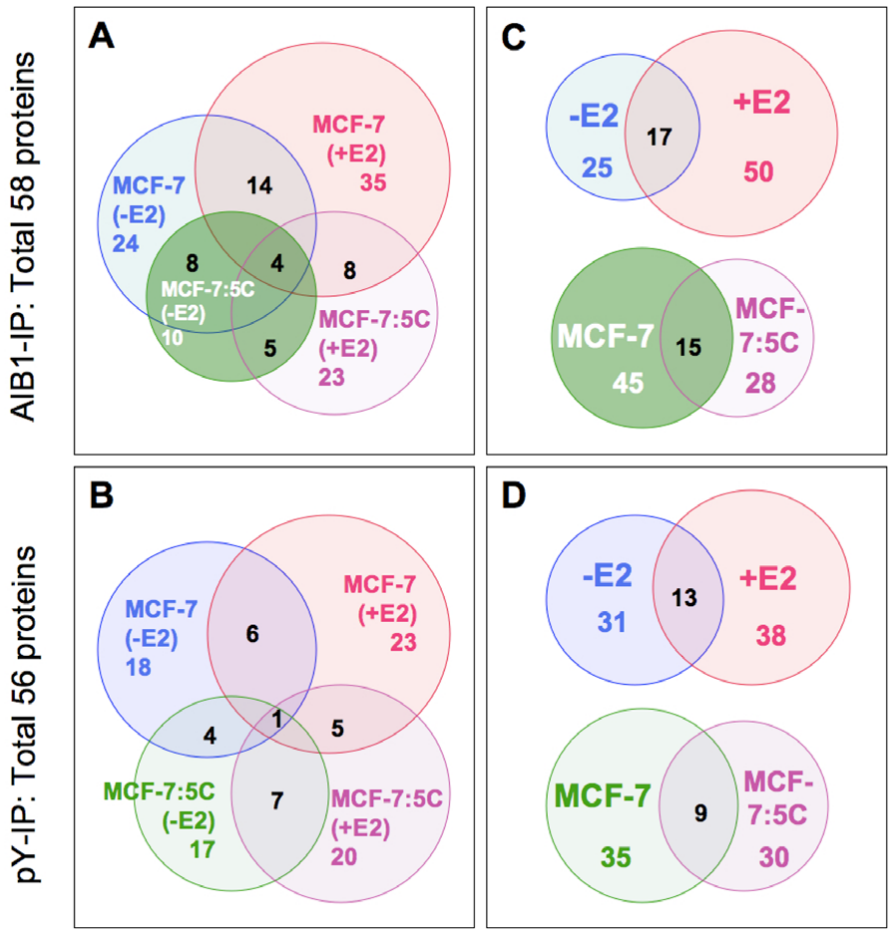
Summary of proteins identified under different conditions. Venn diagrams of proteins identified from anti-AIB1 (*A,C*) or anti-pY IP (*B,D*) experimental groups. (*C,D*) Proteins in combined AIB1-IP or pY-IP data sets. Individual proteins and subgroups are shown in Tables S1 & S2.

The Venn diagrams of proteins pulled down with anti-AIB1 or anti-pY (Fig. 2) show the distribution of proteins between E2-treated and untreated, as well as wild-type MCF-7 versus MCF-7:5C cells (A and B), or between E2-treated and untreated cells regardless of cell type (C, *top*; and D, *top*), or between MCF-7 and MCF-7:5C cells regardless of treatment (C, *bottom*; and D, *bottom*). The number of pY-complexed proteins identified was affected very little by E2 treatment (18 vs. 25 proteins) with 13 proteins in either treatment group (Fig. 2D). In contrast, there was a significant, 4-fold higher number of AIB1-interacting proteins in the E2-treatment group (8 vs. 33 proteins; p<0.05, chi-square test; Fig. 2C) with 17 proteins not impacted in their interaction with AIB1. This suggests that AIB1-mediated protein-protein interactions are more responsive to E2 treatment, and new protein complexes are induced by E2 (Fig. 2A,C). In addition, the total number of proteins in complexes with AIB1 that overlap between MCF-7 and MCF-7:5C cells was not altered by the treatment, although the fraction of proteins per cell line that overlap decreases by 1/2 with E2-treatment (31% to 16%; Fig. 2A). Finally, while pathways activated by E2 gave rise to different sets of pY-containing protein complexes in both MCF-7 and MCF-7:5C cells, the percentage of proteins that overlap between cell lines remain almost constant regardless of treatment (4 vs. 5 in Fig. 2B).

**Figure 3:**
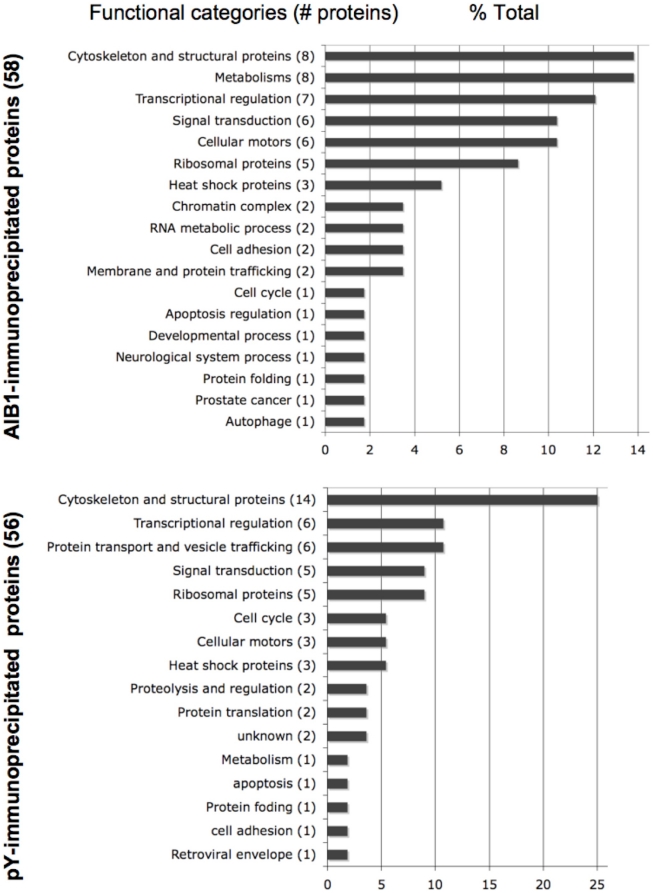
Functional categories of anti-AIB1 (upper) and anti-pY immunoprecipitated proteins (lower) from MCF-7 and MCF-7:5C breast cancer cells. Numbers in parenthesis are the number of proteins belonging to the respective category. Proteins profiled are those with CI values ≥95% from mass spectrometry.

Figure 3 shows the functional categories ascribed to the AIB1-associated (top) and pY-complexed (bottom) proteins. Tables S1 and S2 identify the proteins in each of these categories, cell lines (MCF-7 versus MCF-7:5C), and conditions (+/− E2) under which they were identified. Nearly half of the AIB1-interacting proteins fall into four categories, i.e. cytoskeleton and structural proteins, metabolism, transcription regulation, and signal transduction. Most of the pY-complexed proteins fall into four major functional categories: cytoskeleton and structural proteins, transcription regulation, signal transduction, and protein transport and vesicle trafficking. Thirteen proteins were found to be both AIB1-interacting and pY-complexed in MCF-7 and MCF-7:5C cells (Table S1).

Distinct profiles were observed for metabolism-related proteins between AIB1- and pY-complexed proteins, where the AIB1 complexes contained eight different enzymes in contrast to only one in the anti-pY group. This is consistent with studies demonstrating that AIB1 plays a role in the control of basal metabolic processes [44], [45] that resulted in growth retardation and reduced hormonal responses in AIB1 knock-out mice [46]. Quite strikingly, all of these proteins were identified in E2 treated cells (e.g. 5-oxoprolinase in MCF-7:5C and fatty acid synthase in MCF-7 cells), whereas only three were identified in untreated as well as E2 treated cells. Seven AIB1-interacting proteins were detected in the categories of transcriptional regulation and chromatin complex, consistent with the role of AIB1 as a transcriptional coactivator. Interestingly, several proteins were found with pY immunoprecipitation that were unique to E2-treated MCF-7:5C cells, one of which was FAK1 (PTK2; Table S2). FAK1 is known to complex with EGFR as well as with an isoform of AIB1 and thus contribute to cellular signaling in breast cancer cells [47]. The MS based identification of FAK1 in the anti-pY immunoprecipitates was also seen by Western blot (Fig. S8A).

### AIB1-containing protein complexes in E2-treated MCF-7:5C cells

We identified 18 proteins (CI >95%) that interact with AIB1 in E2-treated but not in untreated MCF-7:5C cells, 10 of which are also unique to MCF-7:5C cells (Table S1; Fig. 2A). These E2-induced AIB1-interacting proteins in MCF-7:5C cells mainly segregate in the category “transcriptional regulation” (6 of 18), several of which are also known to be involved in the control of apoptosis. For example, PRDM5, a PR domain and zinc-finger transcriptional regulator is a putative tumor suppressor and has been linked to cancer cell apoptosis [48]. TLE3, a transcriptional corepressor that binds to a number of transcription factors [49], can form a transcriptional repressor complex with RUNX3 [50], a known tumor suppressor that has been shown to be involved in apoptosis in gastric and colon cancer [51]. TLE3 has also been associated with the development of anti-estrogen resistance [52]. The MS identification of the 83 kDa TLE3 in AIB1 immunoprecipitations (IP) by was also seen by Western blot analysis (Fig. S8B). IASPP was identified in complex with AIB1 in both E2-treated MCF-7 and MCF-7:5C cells, but not in untreated cells. IASPP, a member of ASPP family of proteins, exerts anti-apoptosis effects through modulation of p53 [53], [54], [55]. Interestingly PRPF6, identified here as AIB1-interacting, is an U5 snRNP-associated protein involved in pre-mRNA splicing and has been shown to be a coactivator of the androgen receptor and mediates its ligand-independent AF-1 activation [56]. TLE3, PRDM5 and PRPF6 were all uniquely identified in E2-treated MCF-7:5C cells.

### Potential pathways involved in E2-induced growth and apoptosis

To increase the potential of identifying pathways participating in E2-induced growth and apoptosis from the MS data sets, we not only analyzed proteins identified from MS with high confidence (CI ≥95%), but also took a global approach to include all proteins identified at various CI levels (see http://pir.georgetown.edu/iproxpress/coe2) by MS before filtering for pathway mapping with the Ingenuity™ and GeneGO™ pathway tools [43]. We hypothesized that if proteins identified at lower-level confidence by MS are found in known pathways that are consistent with the cellular phenotypes, they may provide valuable mechanistic insights. Also, supporting this approach are data from a recent study [57] with immunoprecipitation of nuclear extracts from MCF-7 cells that identified 13 of the 15 proteins we had seen at CI values in the lower range of 42–90%. The canonical pathway mapping analyses of all identified proteins suggest that several pathways are significantly represented both for proteins immunoprecipitated with anti-AIB1 and for those with anti-pY, including GPCRs, apoptosis, PI3K/AKT, and Wnt/β-catenin and Notch signaling pathways (Fig. S1, S2, S3, S4):

#### GPCR and growth factor signaling

Figure S1 depicts the GPCR-induced cell growth pathway, in which a number of proteins were identified in both AIB1 and pY-associated complexes. Gα(o) (GNAO2, IP-pY) and Rap1GAP (IP-AIB1) (Table S3), for example were identified exclusively in E2-treated MCF-7:5C cells. Gα(o) has been shown to directly bind to Rap1GAP resulting in the inhibition of the Ras-MAPK proliferation pathway [58]. In E2-treated MCF-7 cells, Gα(s) (GAS, GNAS) and CALM1 were coimmunoprecipitated with AIB1, while IP3R (ITPR3) was coimmunoprecipitated with AIB1 in both E2 treated MCF-7 and MCF-7:5C cells (Table S3). Each of these proteins is found downstream of GPCRs, and could lead to MAPK pathway activation and cell proliferation.

GPCRs and growth factors (IGF-1 and EGF) act via phosphorylation of the proapoptotic Bcl-2 family member BAD to regulate mitochondrial-mediated apoptosis (Fig. S2). BAD has been shown to be phosphorylated by Cdc2 (CDK1) at S128 [59] and Cdc2 was identified by anti-pY immunoprecipitation in E2-treated MCF-7:5C cells (Table S2). Also, two phosphatases, PP2B (PPP3CB) and PP2C (WIP1; Table S3, Fig. S2), associated with AIB1 only in MCF-7 cells. Both phosphatases can dephosphorylate BAD and thus modulate apoptosis [60]. In addition, RSK1 and RSK2, identified only in E2-treated cells (Table S3, Fig. S2), are also known to modulate cell survival [61], [62].

Growth factors and cytokines can induce cellular growth and proliferation through PI3K-AKT signaling. A number of proteins complexed with AIB1 were identified in this pathway under different conditions (Fig. S3 and Table S3). The non-receptor tyrosine kinase TYK2 was detected in both MCF-7 and MCF-7:5C cells with or without E2 treatment. Both PI3K catalytic (p110) and regulatory (p85) subunits were pulled down only in E2-treated, not in untreated MCF-7 cells (Fig. S3C). PI3K/p110 was detected, additionally, in untreated but not treated MCF-7:5C cells (Fig. S3B). Thus, PI3K/p110 was isolated only under conditions that promoted proliferation in both cell lines. GSK3β, identified in AIB1 immunoprecipitates in E2-treated MCF-7 cells (Fig. S3C), can be activated by PI3K/AKT, and has also been shown to be a regulator of Wnt signaling (see below). Finally, BCL3, a member of the I-kappa-B family that regulates NFκB-mediated transcription [63], [64], was only identified in E2-treated MCF-7 cells.

#### Wnt/β-catenin and Notch signaling

Our data indicate that Wnt/β-catenin, and Notch signaling pathways participate in E2 responses in both MCF-7 and MCF-7:5C cells (Fig. S4). Several key proteins in the pathway, such as Wnt ligands, cadherin, β-catenin, casein kinases and GSK3β were identified in distinct AIB1- and pY-containing complexes, amongst different cells and treatments (Fig. S4A, B and C). For example, in MCF-7:5C cells, Frizzled-7 (FZD7) and cadherin 22 (CDH22) were identified in pY-containing complexes after E2 treatment, while β-catenin associated with AIB1 regardless of E2 treatment (Table S3). In MCF-7 cells, the Wnt ligand Wnt-7a, CK1δ, and GSK3β were identified in AIB1 immunoprecipitates (Table S3). CK1δ was recently reported to modulate the transcriptional activity of ERα in an estrogen-dependent manner and regulates ER-AIB1 interactions [65]. An additional protein, δ-catenin, or p120^ctn^, a member of armadillo/β-catenin superfamily [66], was identified in the AIB1 immunoprecipitates of E2-treated MCF-7 cells (Table S1).

**Figure 4:**
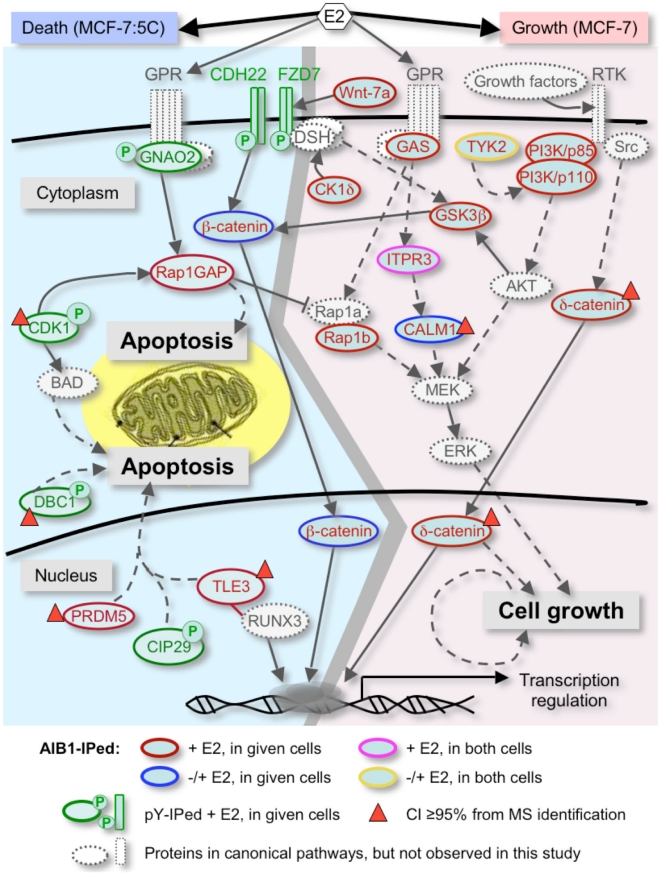
Pathway overview map of proteins involved in E2-induced cell growth or apoptosis in MCF-7 versus MCF-7:5C breast cancer cells. The thick grey line in the middle provides an arbitrary boundary between the pathways. Anti-AIB1 immunoprecipitated (AIB1-IPed) and anti-pY-immunoprecipitated proteins (pY-IPed) are indicated by red or green circles respectively (keys at the bottom). The blue circled proteins are AIB1-IPed proteins from MCF-7 (CALM1) or MCF-7:5C cells (β-catenin) under both E2-treated and untreated conditions; the purple circled one (ITPR3) is an AIB1-IPed protein from both cells only under E2 treated condition, while the yellow circled one (TYK2) is an AIB1-IPed protein from both cells under both E2 treated and untreated conditions. Proteins circled in grey are from known canonical pathways (e.g. ERK in cell growth or BAD in apoptosis) but not identified here. Solid line arrows indicate direct interactions (e.g. CDK1 phosphorylates Rap1GAP) or translocations (e.g. catenins) of proteins, while dashed arrows indicate indirect actions of proteins (e.g. AKT activate MEK through several steps). Hammer-ended lines indicate inhibitory effects on the target. Detailled pathways are shown in Fig. S1, S2, S3, S4.

Our results suggest that multiple proteins found in AIB1 associated complexes, that function in Wnt signaling, also crosstalk with Notch and growth factor-induced signaling in response to E2 treatment in breast cancer cells. TLE3 was detected only in E2-treated MCF-7:5C cells, and Notch1, Notch3, and Numb-like protein were identified only in E2-treated MCF-7 cells (Table S3). TLE3, the mammalian homolog of Gro [67], is a global corepressor mediating transcriptional repression targeted by a number of signal pathways. As shown in Fig. S4D, TLE3 connects the Notch and Wnt pathways [68], [69]. In addition to the apoptosis related proteins discussed above (TLE3, PRDM5, CDK1), DBC1 was isolated from anti-pY immunoprecipitates in E2 treated MCF-7:5C cells (Table S2). Interestingly, DBC1 was recently reported to increase p53 mediated apoptosis in breast cancer cells [70]. Taken together, proteins from GPCR and PI3K/AKT-mediated growth signaling pathways were more prevalent in E2-stimulated MCF-7 cells, whereas proteins related to apoptosis pathways were more prevalent in E2-stimulated MCF-7:5C cells. The respective connectivity of the pathways is depicted in Figure 4.

### Global AIB1 interaction networks

**Figure 5:**
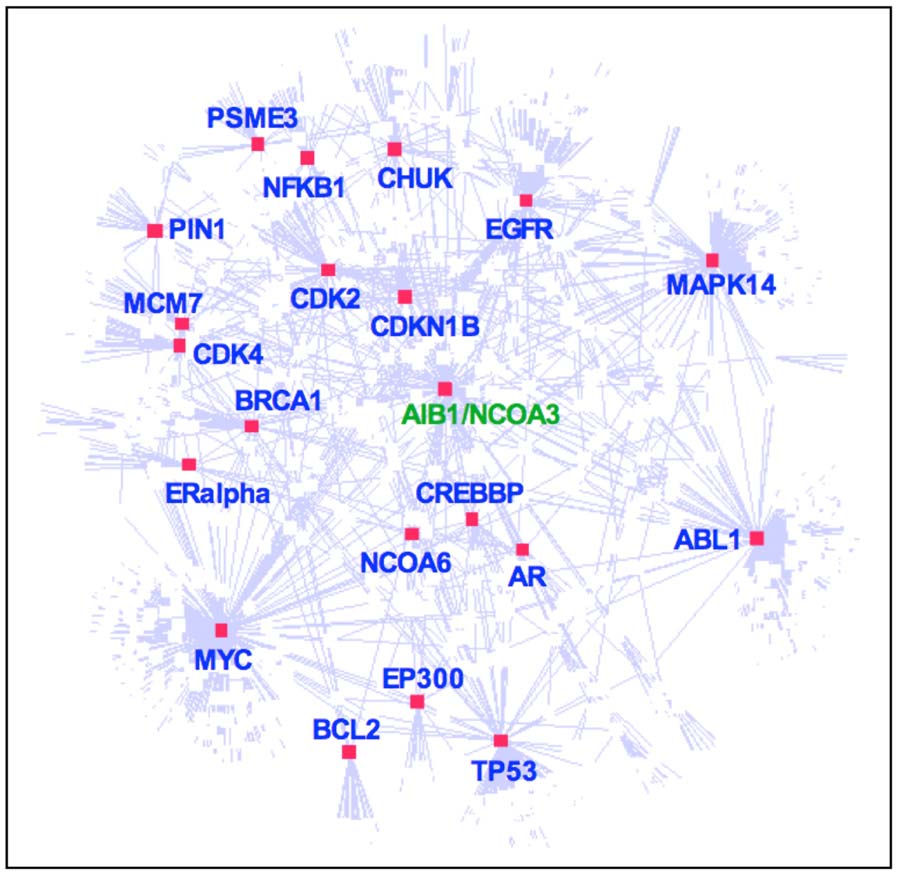
A global AIB1 interaction network showing the major hub proteins. Twenty-one hubs were identified using a cutoff of 20 node degrees. The full names of the respective gene symbols are provided in Table S8. Detailed nodes in the network are shown in Fig. S5.

To extract further information from these experimental data, they were linked with an AIB1 interaction network generated from published data [43]. A computational global AIB1 protein interaction network can be constructed from 91 AIB1 interaction partners (first neighbors) based on the literature published since AIB1 was first described in 1997 [37]. These 91 proteins belong to several major functional categories that include transcription, cell communication, developmental processes and cell cycle regulation. The initial network was expanded to secondary interaction neighbors, based on protein-protein interaction data in the public domain. At this level, the network is composed of 1150 proteins, including 21 highly connected nodes that form major hubs (Fig. 5). These hubs include p53, BRCA1, BCL2, ABL1, CDK2, CDK4, EGFR, ER ( = ESR1), p38, and MYC (Fig. 5 and S5). Closely related subnetworks of AIB1 ( = NCOA3) shown in Figure S5 (*lower panel*), contain four hub proteins: BRCA1, MYC, CDK2 and PSME3. In the present study we identified 26 proteins that are part of the global AIB1 interaction network and function in signal transduction, transcriptional regulation, the cytoskeleton, and the heat shock response.

Eighteen of the proteins experimentally associated with tyrosine-phosphorylated protein complexes are also part of the global AIB1-interaction network. Of these, seven were identified as interacting with AIB1, including CALM1, ACTB, ACTG1, TUBGCP2, MYH9, HSPA1B, and HSPA9. These proteins correspond to interacting hubs, such as CDK4, MYC, PSME3 and CHUK. We conclude that these hubs may participate in the differential cellular responses to E2.

### Connection of E2 transcriptome and proteome effects

An interesting question is to what extent the proteomic pathway mapping parallels mRNA expression profiling in MCF-7 and MCF-7:5C cells. Baseline mRNA expression profiles of these cell lines have been posted earlier (GSE10879; ncbi.nlm.nih.gov/). An analysis of mRNA expression regulation after 48 hrs of E2 treatment of the cells was analyzed and published recently [71]. In MCF-7 cells Bcl-2, a major anti-apoptosis gene, was found upregulated by E2 treatment whereas no change of bcl-2 was seen in MCF-7:5C cells. In our analysis Bcl-2 is one of the major hubs in the AIB1 interaction networks (Fig. 5 and S5). On the other hand, the pro-apototic Bcl-2 antagonists Bak, Bax and Bim mRNAs were found upregulated 2- to 7-fold after E2 treatment of MCF-7:5C cells whereas no mRNA expression change was seen in the MCF-7 cells. Our analysis shows that upstream regulators of the canonical intrinsic mitochondrial pathway such as RSKs, were identified in the proteomics approach (Fig. 4 and S2).

The most differentially regulated mRNA after E2 treatment was Gadd45beta that was found up-regulated 5-fold in MCF-7:5C cells but down-regulated 5-fold in MCF-7 cells [71]. Gadd45beta was described earlier as a hub of the MAP kinase signaling cascade and connects to relA, the NFkappaB p65 subunit (see e.g. Ref. [72]) as well as cell survival in apoptosis resistant cells [73]. We isolated components of GPCR signaling in our proteomics analysis (Fig. 4 and Fig. S1) that can connect to these downstream effectors and can thus serve as trigger mechanisms. Interestingly, GPR30 mRNA was found upregulated in MCF-7:5C cells after estradiol treatment [40] and GPR30 was shown to rapidly transmit non-genomic effects of E2 in breast cancer cells [74]. Overall, the mRNA expression analyses and proteomics data show some interesting convergences especially in apoptotic regulatory pathways which may be functionally relevant as initiators of estradiol–induced apoptosis or cell survival.

### Conclusions

The estrogen induced apoptotic response is most strongly associated with early signaling changes in G-protein coupled receptors, PI3 kinase, Wnt and Notch signaling and are integrated here into a global AIB1 signaling network that controls qualitatively distinct responses to estrogen.

## Materials and Methods

### The overall experimental design

We used combined proteomics and bioinformatics approaches [43] to identify the E2 induced signaling pathways and networks that are associated with AIB1 and/or tyrosine phosphorylated proteins and that differentiate the MCF-7 from MCF-7:5C cells in responses to E2 treatment (Fig. 1A). A single early time point after E2 treatment (2 hrs) was examined to capture signaling events that drive apoptosis or proliferation in these cells. Repeat independent proteomic experiments for each of the 4 experimental conditions and the two different immunoprecipitations were run.

### Cell culture

MCF-7 (ATCC) human breast cancer cells and the MCF-7 variant MCF-7:5C [75] , which is a clonal variant of MCF-7 derived after longterm estrogen deprivation, were cultured in RPMI-1640 without Phenol Red (Invitrogen) supplemented with 10% FBS, or in RPMI-1640 supplemented with 10% charcoal/dextran-stripped FBS (Hyclone) and other supplements, respectively, as described previously [38]. MCF-7 or MCF-7:5C cells deprived of steroid hormones for 2 days were plated at a density of 2,000 and 3,000 cells per well, respectively, in 96-well cell culture plates. One day after plating, cells were treated with E2 (in ethanol) or vehicle (ethanol). To monitor the portion of viable cells after 6 days of growth, the CellTiter-Glo luminescent cell viability assay (Promega) or WST1 colorimetric cell proliferation assay (Roche) were used. Typical readings of baseline growth without E2 were 2.0×10^5^ RLU (CellTiter-Glo) or an OD450 of 0.5 (WST1). Data are shown relative to the baseline.

### Infection of MCF-7 and MCF-7:5C with lentiviral shRNA expression vectors

Prior to infection, MCF-7 and MCF-7:5C cells were plated at a density of 3×10^5^ cells on 10 cm tissue culture dishes. 24 hrs later, cells were infected with lentiviral particles expressing control or AIB1-targeting shRNAs (in pLKO.1). The AIB1(1) shRNA was derived from an siRNA for AIB1 previously described [25], and the AIB1(2) shRNA was from Sigma (TRCN0000019703). The control shRNA used in the experiments is a scrambled sequence described previously [76]. Briefly, 1 ml of lentivirus-containing supernatant was added to 9 ml of growth medium and 8 ng/ml polybrene, and then added to cells for 24 hrs. Medium containing lentivirus was then replaced with growth medium without lentivirus. After two days, cells were treated for 48 hours with 5 µg/ml puromycin for the selection of lentiviral shRNA expression.

### Western blot analysis, immunoprecipitation and protein isolation

Western blot analyses were done as previously described [25], using a monoclonal antibody for AIB1 (SRC3; clone 5E11, Cell Signaling). For the mass spectrometry analysis, protein lysates from cells treated for 2 hours with E2 or vehicle were subjected to immunoprecipitation using gamma-bind G-Sepharose beads and an anti-AIB1 monoclonal antibody (BD Biosciences) as described [77] or an anti-phosphotyrosine monoclonal antibody (4G-10, Millipore). The amount of protein input for immunoprecipitations ranged between 7 mg and 14 mg for each of the experimental conditions with bovine serum albumin used as the standard. It is noteworthy that over a 24 hour period of E2 treatment of cells the AIB1 protein expression levels varied less than 2-fold as illustrated in Figure S6. The immunoprecipitated proteins were separated by denaturing SDS-PAGE on 4–12% Nu-PAGE gels (Invitrogen). After electrophoresis, gels were stained with Coomassie blue overnight and washed with ddH2O overnight to remove background staining. Stained gels were imaged using a color scanner and visible bands were cut from the gels. The corresponding segments of lanes from the different treatments were also cut for analyses and served as controls. Figure S7 shows a representative set of stained gels with an overlay of the grid of segments harvested for the mass spectrometry analyses.

### Mass spectrometry analysis

SDS-PAGE gel slices were subjected to tryptic digest and followed by MS and MS/MS on an ABI MALDI-TOF-TOF. Proteins in the MS or MS/MS analysis were identified based on searches of the Swiss-Prot database using the search engine Mascot 2.0. The Swiss-Prot database searched was based on its 9/24/2007 release (287,050 sequences). The database search parameters used were: 1) enzyme specificity considered, trypsin; 2) number of missed cleavages permitted, 1; 3) fixed modification(s), carbamidomethyl (C); 4) variable modification(s), oxidation (M); 5) mass tolerance for precursor ions, 75ppm; and 6) mass tolerance for fragment ions, 0.3 Da. Trypsin autolysis peaks were excluded from the peak list. GPS Explorer (Version 3.0) with default parameter setting was used to generate the peak list from raw data which were submitted to database searches using Mascot. The confidence interval (CI) for the peptide identification was calculated by GPS Explorer. A CI of ≥95% (or expectation value ≤0.05) was used as a cut off for the high CI proteins.

### Bioinformatics Analysis

#### Protein data filtering

Proteins identified from mass spectrometry were subjected to extensive bioinformatics analysis, including protein data filtering, functional profiling and pathway mapping as described previously [78]. Protein identities from different experimental groups were assigned levels of identification confidence based on statistical processing by GPS Explorer™ of the MASCOT search results. It is commonly known that false negative identification is generated because low-scored proteins may result from factors such as database size, protein abundance and the type of mass spectrometry instrumentation. Therefore, in addition to analyzing the proteomic data based on the prioritized list of proteins with high Confidence Interval (CI; Tables S1, S2), we also used a global approach for pathway mapping on proteins identified at all confidence levels. We provide the identity, CI and spectra of those proteins as well as the reference to the respective pathway figures in Table S3.

We used the following criteria to filter the protein lists. (i) Proteins with MS confidence interval (CI) values smaller than 95% were removed to reduce false-positive results; (ii) Proteins described to be non-specific interactors e.g. HSPA5 and Desmoplakin [79] were removed; (iii) High abundant, non-specific proteins e.g. keratins were removed; (iv) Proteins migrating at an apparent mass in the SDS-PAGE that was different from the calculated mass or the experimentally described mass or the predicted mass were removed. A representative set of Coomassie stained gels after immunoprecipitations is shown in Fig. S7 to illustrate this latter consideration.

#### Protein annotation, profiling and pathway analysis

The iProXpress bioinformatics system (http://pir.georgetown.edu/iproxpress) was used for protein annotation, function and pathway profiling of the proteomics data. The experimental group(s) in which the proteins were identified was annotated for all proteins and integrated into the iProXpress system for direct functional comparison between selected groups, such as cell types, E2 treatment, and experimental repeats. The procedure of using iProXpress system has been described recently [43], [78]. The data sets are accessible at http://pir.georgetown.edu/iproxpress/coe2/. Pathway mapping and network visualization are assisted with Ingenuity Pathways Analysis (IPA) (www.ingenuity.com) and GeneGO MetaCore (www.GeneGO.com) software tools.

#### Data mining for known AIB1 interactors

The global AIB1 interaction network refers to a network of genes or proteins that directly or indirectly interact or are functionally associated with AIB1 regardless of cell/tissue types or species in which the interaction occurs. The network is was computationally generated based on two sources of data, i.e. the published literature (PubMed) and protein-protein interactions (PPI) available from public databases. A list of AIB1 synonyms included as query terms “AIB1 OR AIB-1 OR NCOA3 OR NCOA-3 OR SRC3 OR SRC-3 OR TRAM1 OR ACTR OR pCIP” to search PubMed and retrieved a total of about 650 papers related to AIB1. Of these papers about 250 papers that contain AIB1 interaction or functional association information were curated, and a total of 91 AIB1 interaction partners were thus obtained. The interaction types in the literature included physical interactions, such as “*binding*”, “*complex*”, “*interact*”, “*phosphorylation*”, etc., and functional associations, such as “*activation*”, “*correlated expression*”, “*lead to degradation*”, “*modulate*”, “*promoter binding*”, “*suppression*”, etc. These interacting proteins/genes reported for human as well as other species from mouse to *Xenopus*, were mapped to corresponding human orthologs based on UniProtKB database.

The protein/protein interaction (PPI) data annotated in bioinformatics databases were obtained from IntAct database [80], which contains high throughput PPI data from Y2H and IP in addition to literature data. The AIB1 interaction network was constructed based on the binary interactions of the curated 91 AIB1-interacting proteins and those from the PPI database. The network was clustered and filtered, and major hubs were selected using a cutoff of a node degree of 20. Cytoscape open source software was used to display the network for visual examinations.

## Supporting Information

Figure S1 **Proteins identified in GPCR signaling pathways.** Canonical cell growth pathways initiated by GCPR signaling are depicted based on the MetaCore pathway tool of GeneGO. The AIB1- and pY-IPed proteins identified from the study were mapped to the pathway using MetaCore, which were manually re-annotated in the red-lined white boxes with black arrows pointing to the specific protein depictions. The corresponding experimental conditions under which the proteins were identified are indicated at the bottom. Proteins were AIB1-IPed under conditions indicated as A–D, or pY-IPed indicated by “p”.
(TIF)

Figure S2 **Proteins identified in apoptosis pathways.** The canonical intrinsic mitochondrial apoptosis pathway is depicted based the MetaCore pathway tool of GeneGO. Similar to Fig. S3, the anti-AIB1- and pY-IPed proteins identified from the study were mapped to the pathway and were manually re-annotated with red-lined white boxes with the specific protein identified here.
(TIF)

Figure S3 **Proteins identified in the PI3K/AKT pathway.** The canonical PI3K/AKT pathway is depicted based on the Ingenuity pathway tool. AIB1-IPed proteins that were mapped to the canonical pathway are shown as orange-colored shapes in four panels, each representing the same PI3K/AKT pathway with different mapped proteins that were identified from untreated MCF-7 (A) or MCF-7:5C (B) and E2-treated MCF-7 (C) or MCF-7:5C (D) cells. Some proteins in the pathway were manually re-annotated with green-colored box to indicate the specific protein forms identified in this study that correspond to the protein classes represented in the canonical pathway, e.g. JAK refers to the non-receptor type tyrosine kinases, such as TYK2 here.
(TIF)

Figure S4 **Proteins identified in the Wnt/β-catenin pathway.** The canonical Wnt/β-catenin pathway is depicted based on the Ingenuity pathway tool. AIB1-IPed proteins that can be mapped to the canonical pathway are shown as orange-colored shapes in four panels, each representing the same Wnt/β-catenin pathway with different mapped proteins that were identified from untreated MCF-7 (A) or MCF-7:5C (B) and E2-treated MCF-7 (C) or MCF-7:5C (D) cells. Some proteins in the pathway were manually re-annotated with green-colored box to indicate the specific protein forms identified in the experiment that correspond to the classes represented in the canonical pathway, e.g. Wnt refers to class of Wnt ligands, such as Wnt-4 and Wnt-7a. Some proteins manually labeled with a “P” in red indicate that they were identified as pY-IPed.
(TIF)

Figure S5 **AIB1 interaction network.** A global AIB1 interaction network (upper) and the selected sub-networks (lower) are shown. The overall topology of the network is displayed with Spring-embedded layout using Cytoscape network visualization software before network clustering (image can be zoomed in to view individual node). Proteins that are identified with high confidence in this study are colored as green (AIB1-IPed), yellow (pY-IPed) or dark brown (both AIB1- and pY-IPed) nodes. Hub proteins that are subsequently clustered with AIB1 in several subnetworks are indicated with arrows (*upper*). Individual nodes in AIB1-clustered subnetworks are shown in the lower panel, with major functional categories labeled for the hub proteins.
(TIF)

Figure S6 **Western blot analysis for AIB1.** Cells treated with E2 for different times were harvested and Western blot analysis for AIB1 was performed as described in Materials and Methods.
(TIF)

Figure S7 **Coomassie stained protein gels after anti-AIB1 or -pY immunoprecipitation (IP).** MCF-7 and MCF-7:5C cells were treated or not with E2 for 2 hours, and proteins were extracted for IP. The immunoprecipitated proteins were separated by 4–12% Nu-PAGE, stained, washed with ddH20 and imaged using a color scanner. The images were magnified and analyzed visually on a screen. After identification, bands were cut from the gels and great care was taken to isolate the same segment of all lanes from the different treatments for a parallel MS analysis. Representative stained gels with the segments to be cut for analysis are indicated. Slices numbered 1–10 or 1–13 were cut from the gels for each segment that showed at least one distinctly regulated protein. Molecular masses of marker proteins are indicated (10–250 kDa).
(TIF)

Figure S8 **Western blot analysis confirms that FAK1 and TLE3 are immunoprecipitated from E2 treated MCF7:5C cells.** MCF-7:5C cells were treated or not with E2 for 2 hours, and proteins were extracted for IP/Western analysis A) Tyrosine-phosphorylated endogenous proteins were immunoprecipitated with anti-phosphotyrosine monoclonal antibody (4G-10, Millipore) and the immunoprecipitate was resolved on SDS-PAGE followed by Western analysis. The input is 5% of the amount of total cell lysates for IP. FAK1 was detected on the blot with an anti-FAK1 antibody (A-17, Santa Cruz). B) AIB1 interacting proteins were immunoprecipitated using an anti-AIB1 monoclonal antibody (BD Biosciences). The input is 5% of the amount of total cell lysates for IP. TLE3 was detected on the blot with a TLE3 antibody (Abcam).
(TIF)

Table S1 **AIB1-interacting proteins with a CI value of ≥95%.** AIB1-interacting proteins (n = 58) isolated from MCF-7 and MCF-7:5C cells identified by MALDI-MS/MS with a CI value of ≥95% are listed and assigned with functional categories. The number of peptides identified and % coverage are in Table S4. Various experimental groups in which AIB1-interacting proteins were identified, are shown in the right side columns (with vertical column names), and the number of total proteins in each group is given in parenthesis. Proteins are arranged by their functional categories (see Fig. 3) and the number of proteins in each experimental group of a given category is also indicated in the same row of the category. The column furthest to the right shows AIB1-interacting proteins in this study that are also identified as part of the AIB1 protein interaction (int.) network. “X” indicates the presence of a given protein in a given experimental group or in the AIB1 interaction network. Asterisks by the protein accession indicate AIB1-interacting proteins that are also identified in pY complexes (see Table S2).
(DOC)

Table S2 **Phosphotyrosine complexed proteins with a CI value of ≥95%.** Proteins pulled down with anti-pY in MCF-7 and MCF-7:5C cells identified from MALDI-MS/MS with a CI value of ≥95% are listed and assigned with functional categories. The number of peptides identified and % coverage are in Table S5. Various experimental groups in which tyrosine-phosphorylated proteins are identified are shown in the right columns (with vertical column names), and the number of proteins in each group is given in parenthesis. Proteins are arranged by their functional categories (see Fig. 3) and the number of proteins in each experimental group of a given category is also indicated in the same row of the category. “X” indicates the presence of a given protein in a given experimental group or in the AIB1 interaction network. Asterisks by the protein accession indicate IP-pY complexes that are also identified as AIB1-interacting (see Table S1).
(DOC)

Table S3 **Pathway mapping of proteins identified with a CI<95%.** Proteins are listed alphabetically based on the “gene name” column for anti-AIB1 or anti-pY immunoprecipitated proteins. In the “Experiment” column A to D indicate: A, MCF-7 cells, no E2; B, MCF-7:5C cells, no E2; C, MCF-7 cells, +E2; and D, MCF-7:5C cells, +E2. The “Spec” column references the corresponding mass spectrum for single peptide MS/MS identification in the section “Single peptide spectral data” appended at the end of this table. The “Figures” column indicates in which figure(s) the proteins are depicted, except for a few only discussed in the main text (*text*). In the spectral data section, the underlined C and M in “peptide sequences” column represent fixed (carbamidomethyl) and variable (oxidation) modifications, respectively. *MALDI-TOF-MS generates peptides containing only one charge, and the precursor m/z is thus equal to the precursor mass. NA, not available.
(DOC)

Table S4 **AIB1-complexed proteins identified by MALDI-TOF-TOF.** Proteins were identified based on single MS (MS) or tandem MS (MS/MS) using the search engine Mascot 2.0 from the Swiss-Prot database. Note that the same proteins could be identified under different experimental (“Exp.”) conditions: A, MCF-7 cells, no E2; B, MCF-7:5C cell, no E2; C, MCF-7 cell, +E2; D, MCF-7:5C cell, +E2. For proteins identified from single peptide MS/MS, spectral data (Spec.) are referenced using the labels (A1–A30) to correspond to those shown in Table S6. All spectra for single peptides shown here are manually inspected, including the one that shows CI of 93% but with good ion fragments. *The % coverage for single peptide MS/MS was only stated if the respective peptide covered ≥1% of the protein. The spectra and sequences are in Table S6.
(DOC)

Table S5 **pY-complexed proteins identified by MALDI-TOF-TOF.** Proteins were identified based on single MS (MS) or tandem MS (MS/MS) using the search engine Mascot 2.0 from the Swiss-Prot database. Note that the same proteins could be identified under different experimental (“Exp.”) conditions: A, MCF-7 cells, no E2; B, MCF-7:5C cell, no E2; C, MCF-7 cell, +E2; D, MCF-7:5C cell, +E2. Proteins that were identified more than once from experimental repeats under the same conditions are labeled with * in the “Exp” column. For proteins identified from single peptide MS/MS, spectral data (Spec.) are referenced using the labels (Y1–Y38) to correspond to those shown in Table S7. All spectra for single peptides shown here were manually inspected, including those that show 90%≤ CI ≤95% but with good ion fragments. *The % coverage for single peptide MS/MS was only stated if the respective peptide covered ≥1% of the protein. The spectra and sequences are in Table S7.
(DOC)

Table S6 **MS/MS spectra for single peptide identified AIB1-complexed proteins.** The “No.” column labels the spectra sequentially as referenced in Table S4. The “Exp.” column indicates the experimental conditions under which the respective protein was identified: A, MCF-7 cells, no E2; B, MCF-7:5C cell, no E2; C, MCF-7 cell, +E2; D, MCF-7:5C cell, +E2. The underlined C and M in peptide sequences represent fixed (carbamidomethyl) and variable (oxidation) modifications, respectively. *MALDI-TOF-MS generates peptides containing only one charge and the precursor m/z (not shown) is thus equal to the precursor mass.
(DOC)

Table S7 **MS/MS spectra for single peptide identified pY-complexed proteins.** The “No.” column labels the spectra sequentially as referenced in Table S5. The “Exp.” column indicates the experimental conditions under which the protein was identified: A, MCF-7 cells, no E2; B, MCF-7:5C cell, no E2; C, MCF-7 cell, +E2; D, MCF-7:5C cell, +E2. The underlined C and M in peptide sequences represent fixed (carbamidomethyl) and variable (oxidation) modifications, respectively. *MALDI-TOF-MS generates peptides containing only one charge and the precursor m/z (not shown) is thus equal to the precursor mass.
(DOC)

Table S8 **List of acronyms used.**
(DOC)
