# Supplementary figures and images for: Proteomic Analysis of Pathways Involved in Estrogen-Induced Growth and Apoptosis of Breast Cancer Cells
Source: PLoS One. 2011 Jun 27;6(6):e20410. doi: 10.1371/journal.pone.0020410 (PMC3124472; doi:10.1371/journal.pone.0020410)

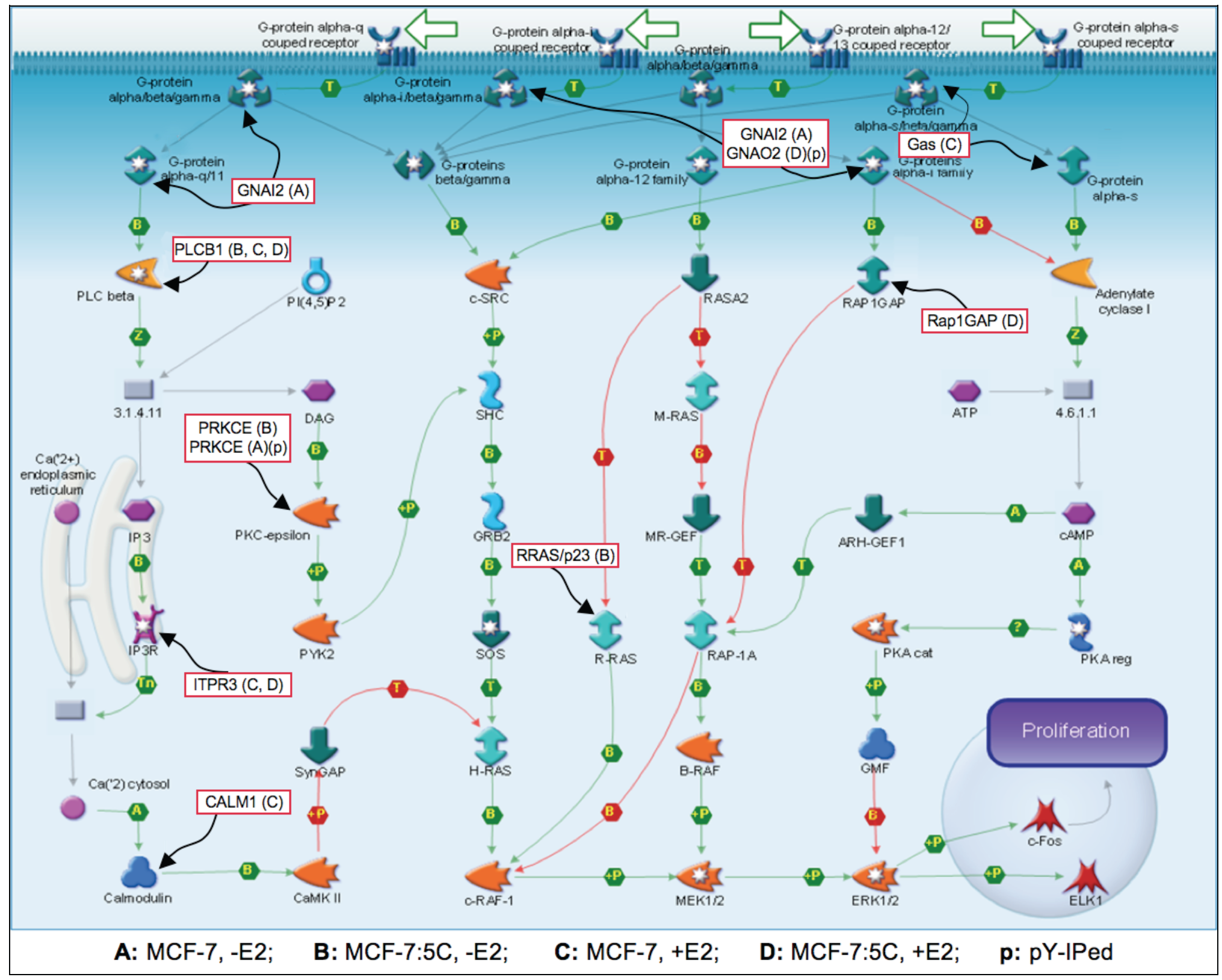

Supplement: Figure S1 — Proteins identified in GPCR signaling pathways. Canonical cell growth pathways initiated by GCPR signaling are depicted based on the MetaCore pathway tool of GeneGO. The AIB1- and pY-IPed proteins identified from the study were mapped to the pathway using MetaCore, which were manually re-annotated in the red-lined white boxes with black arrows pointing to the specific protein depictions. The corresponding experimental conditions under which the proteins were identified are indicated at the bottom. Proteins were AIB1-IPed under conditions indicated as A–D, or pY-IPed indicated by “p”. (TIF) [file pone.0020410.s001.tif]

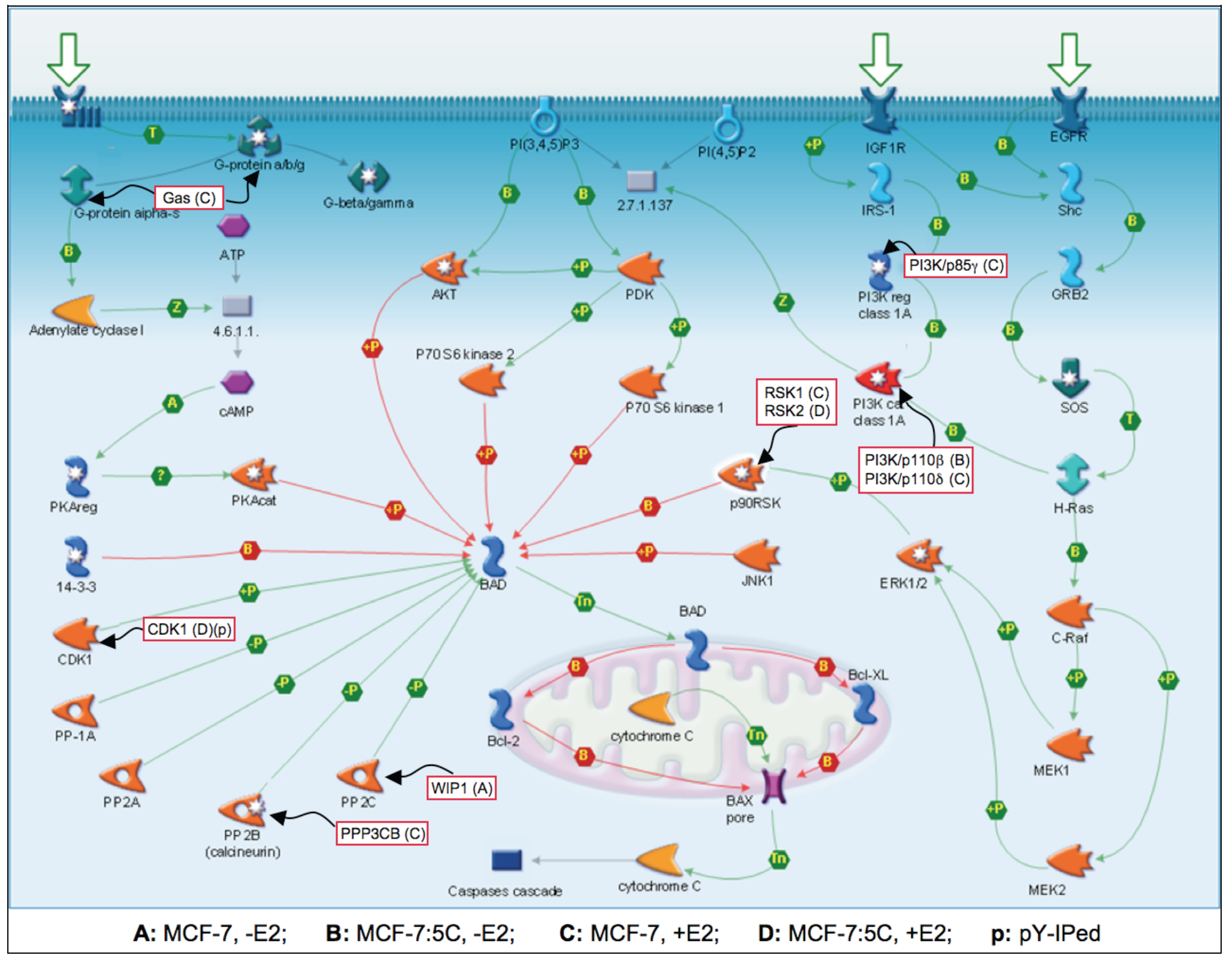

Supplement: Figure S2 — Proteins identified in apoptosis pathways. The canonical intrinsic mitochondrial apoptosis pathway is depicted based the MetaCore pathway tool of GeneGO. Similar to Fig. S3, the anti-AIB1- and pY-IPed proteins identified from the study were mapped to the pathway and were manually re-annotated with red-lined white boxes with the specific protein identified here. (TIF) [file pone.0020410.s002.tif]

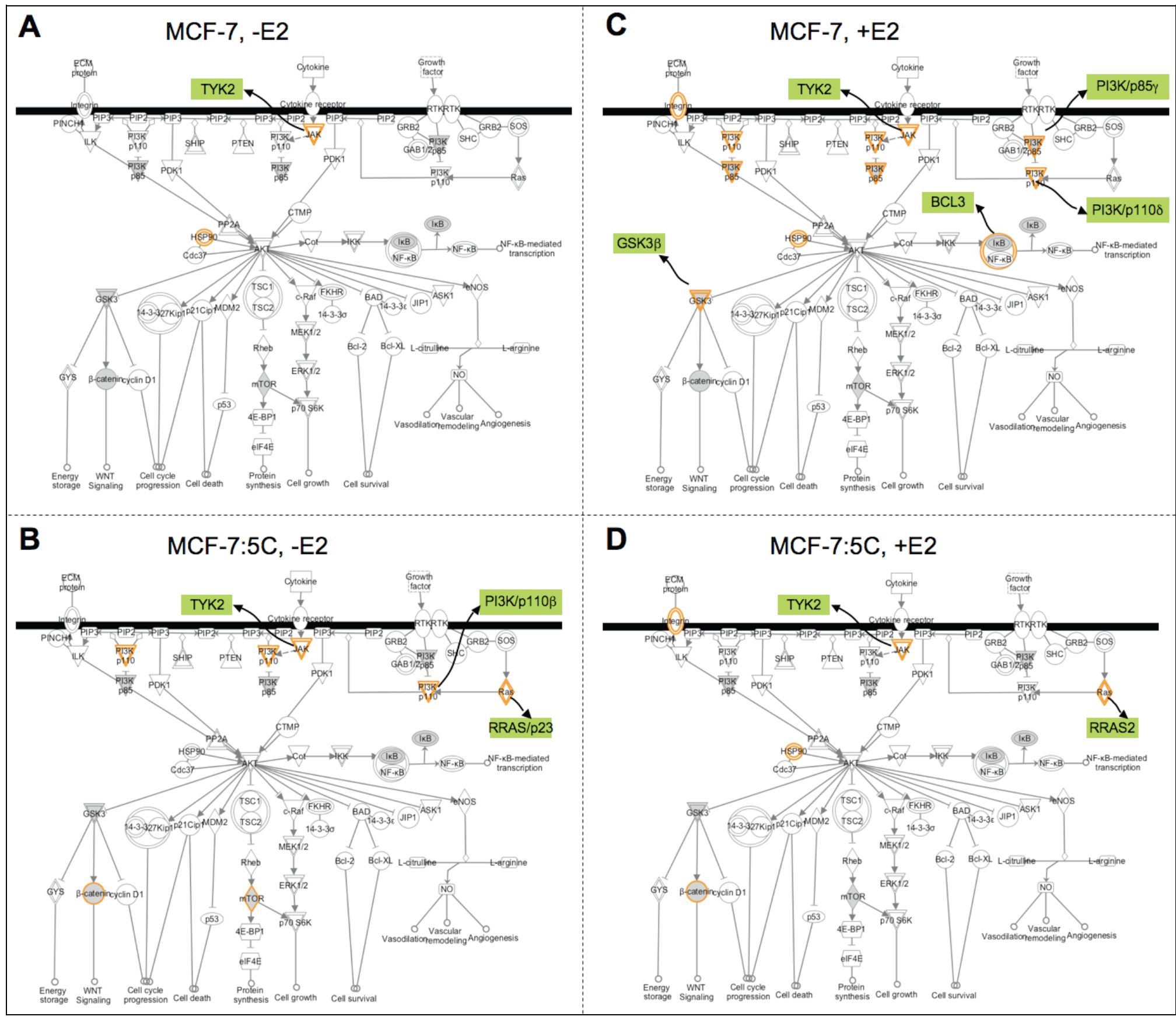

Supplement: Figure S3 — Proteins identified in the PI3K/AKT pathway. The canonical PI3K/AKT pathway is depicted based on the Ingenuity pathway tool. AIB1-IPed proteins that were mapped to the canonical pathway are shown as orange-colored shapes in four panels, each representing the same PI3K/AKT pathway with different mapped proteins that were identified from untreated MCF-7 (A) or MCF-7:5C (B) and E2-treated MCF-7 (C) or MCF-7:5C (D) cells. Some proteins in the pathway were manually re-annotated with green-colored box to indicate the specific protein forms identified in this study that correspond to the protein classes represented in the canonical pathway, e.g. JAK refers to the non-receptor type tyrosine kinases, such as TYK2 here. (TIF) [file pone.0020410.s003.tif]

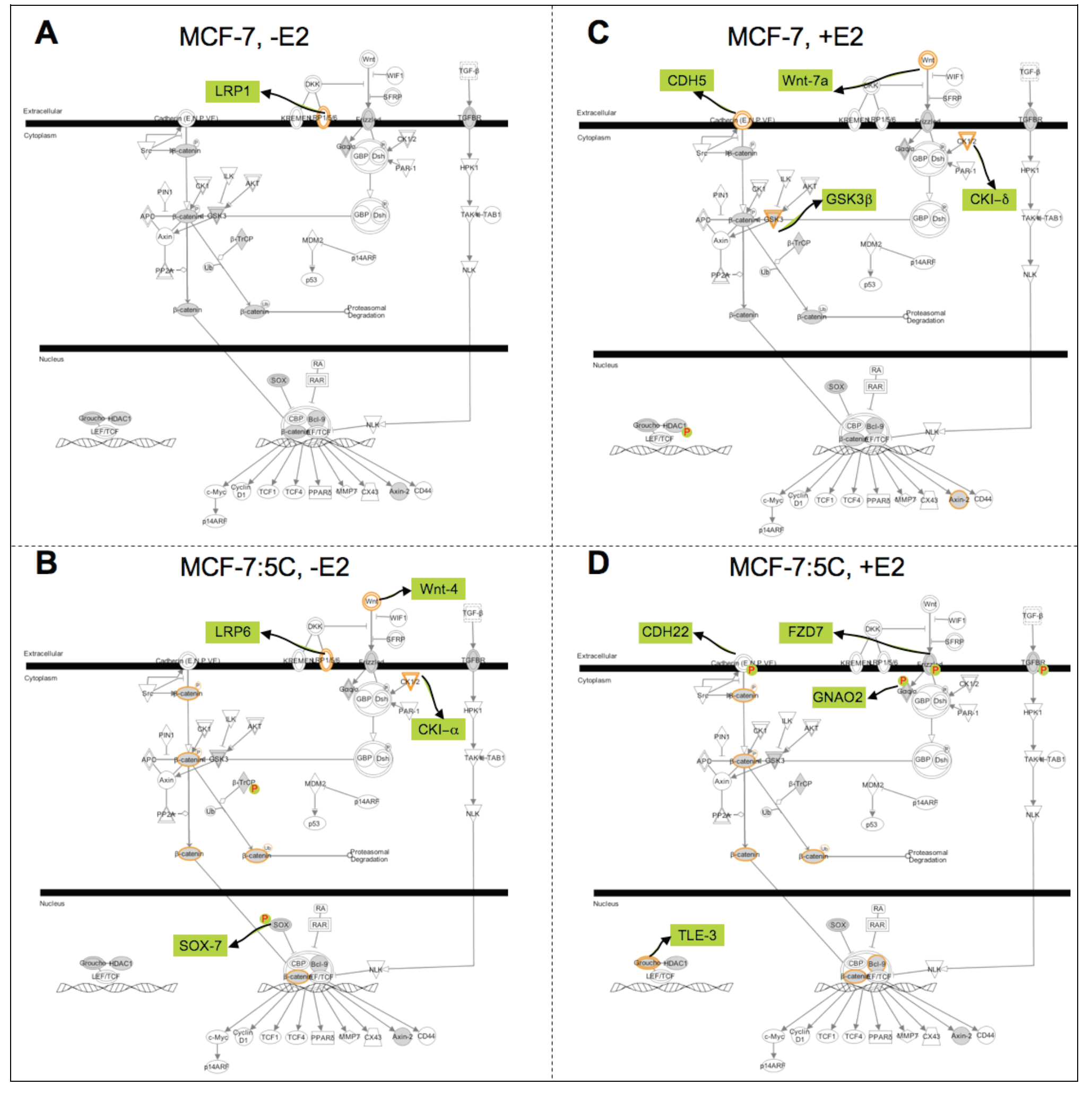

Supplement: Figure S4 — Proteins identified in the Wnt/β-catenin pathway. The canonical Wnt/β-catenin pathway is depicted based on the Ingenuity pathway tool. AIB1-IPed proteins that can be mapped to the canonical pathway are shown as orange-colored shapes in four panels, each representing the same Wnt/β-catenin pathway with different mapped proteins that were identified from untreated MCF-7 (A) or MCF-7:5C (B) and E2-treated MCF-7 (C) or MCF-7:5C (D) cells. Some proteins in the pathway were manually re-annotated with green-colored box to indicate the specific protein forms identified in the experiment that correspond to the classes represented in the canonical pathway, e.g. Wnt refers to class of Wnt ligands, such as Wnt-4 and Wnt-7a. Some proteins manually labeled with a “P” in red indicate that they were identified as pY-IPed. (TIF) [file pone.0020410.s004.tif]

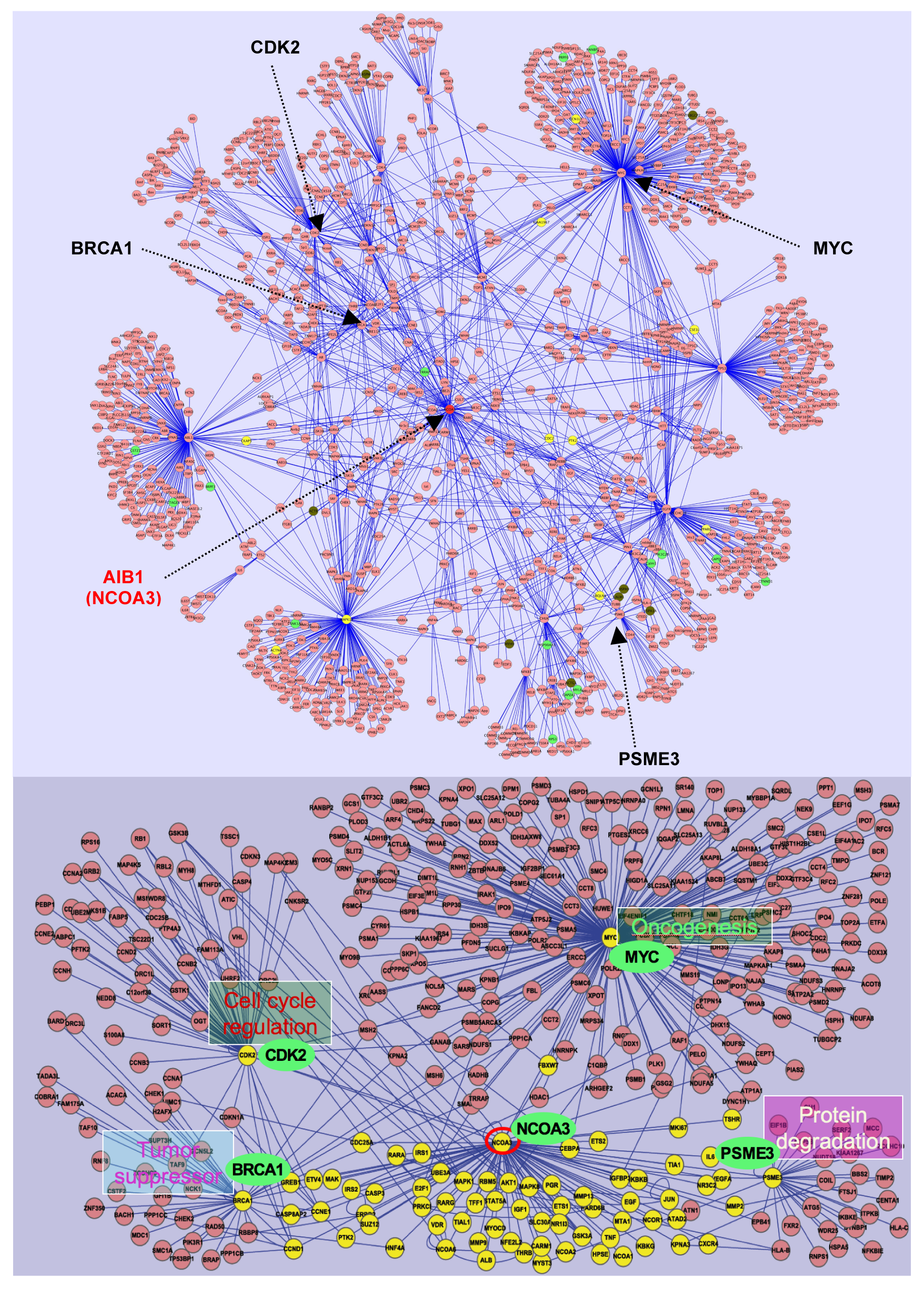

Supplement: Figure S5 — AIB1 interaction network. A global AIB1 interaction network (upper) and the selected sub-networks (lower) are shown. The overall topology of the network is displayed with Spring-embedded layout using Cytoscape network visualization software before network clustering (image can be zoomed in to view individual node). Proteins that are identified with high confidence in this study are colored as green (AIB1-IPed), yellow (pY-IPed) or dark brown (both AIB1- and pY-IPed) nodes. Hub proteins that are subsequently clustered with AIB1 in several subnetworks are indicated with arrows (upper). Individual nodes in AIB1-clustered subnetworks are shown in the lower panel, with major functional categories labeled for the hub proteins. (TIF) [file pone.0020410.s005.tif]

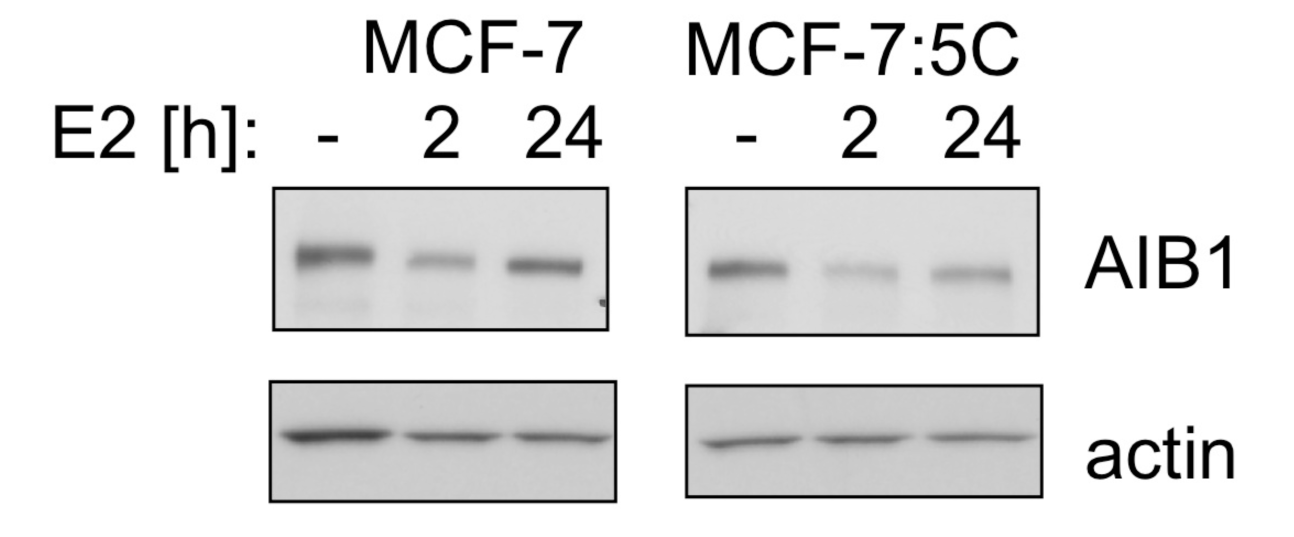

Supplement: Figure S6 — Western blot analysis for AIB1. Cells treated with E2 for different times were harvested and Western blot analysis for AIB1 was performed as described in Materials and Methods. (TIF) [file pone.0020410.s006.tif]

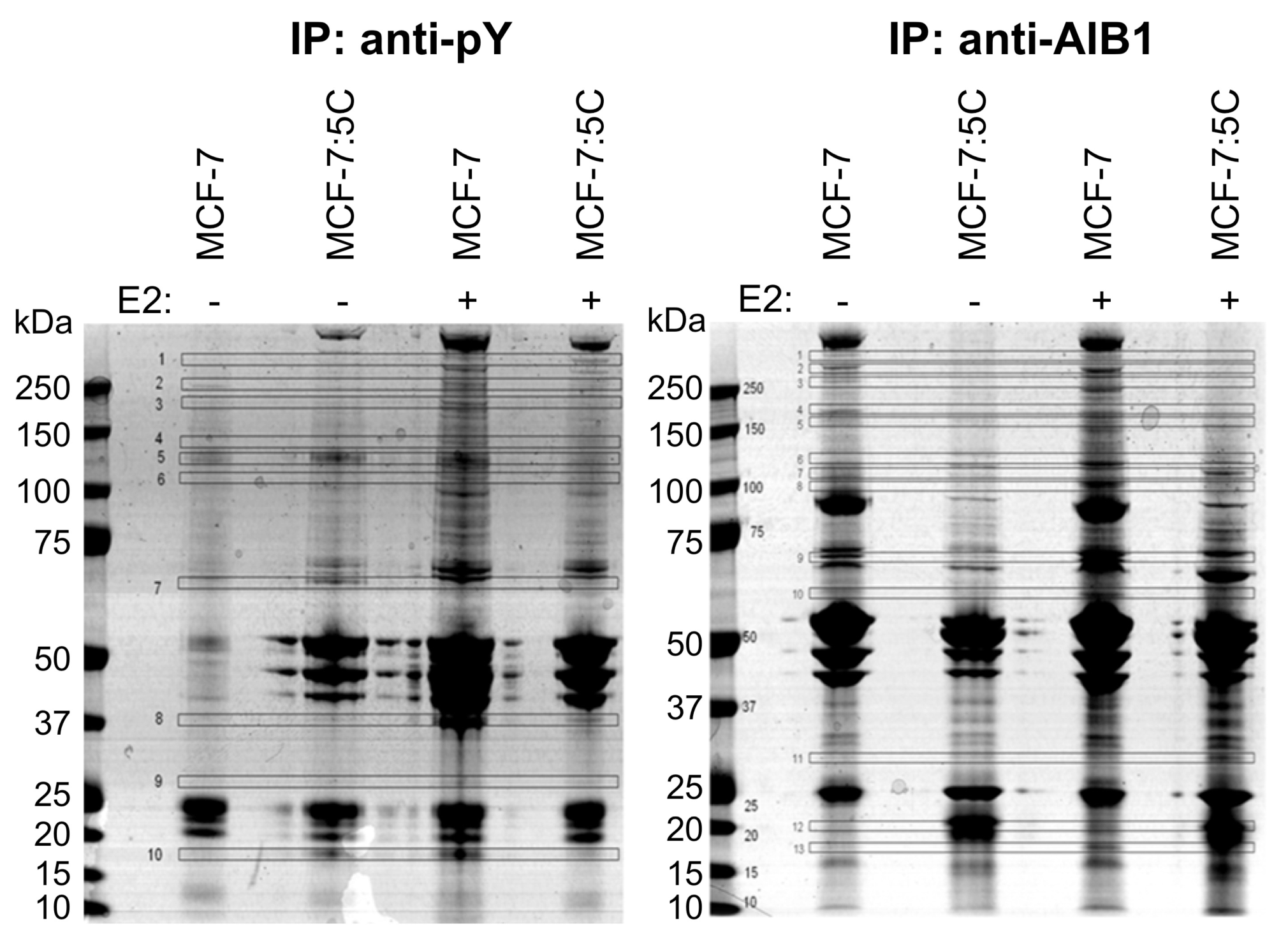

Supplement: Figure S7 — Coomassie stained protein gels after anti-AIB1 or -pY immunoprecipitation (IP). MCF-7 and MCF-7:5C cells were treated or not with E2 for 2 hours, and proteins were extracted for IP. The immunoprecipitated proteins were separated by 4–12% Nu-PAGE, stained, washed with ddH20 and imaged using a color scanner. The images were magnified and analyzed visually on a screen. After identification, bands were cut from the gels and great care was taken to isolate the same segment of all lanes from the different treatments for a parallel MS analysis. Representative stained gels with the segments to be cut for analysis are indicated. Slices numbered 1–10 or 1–13 were cut from the gels for each segment that showed at least one distinctly regulated protein. Molecular masses of marker proteins are indicated (10–250 kDa). (TIF) [file pone.0020410.s007.tif]

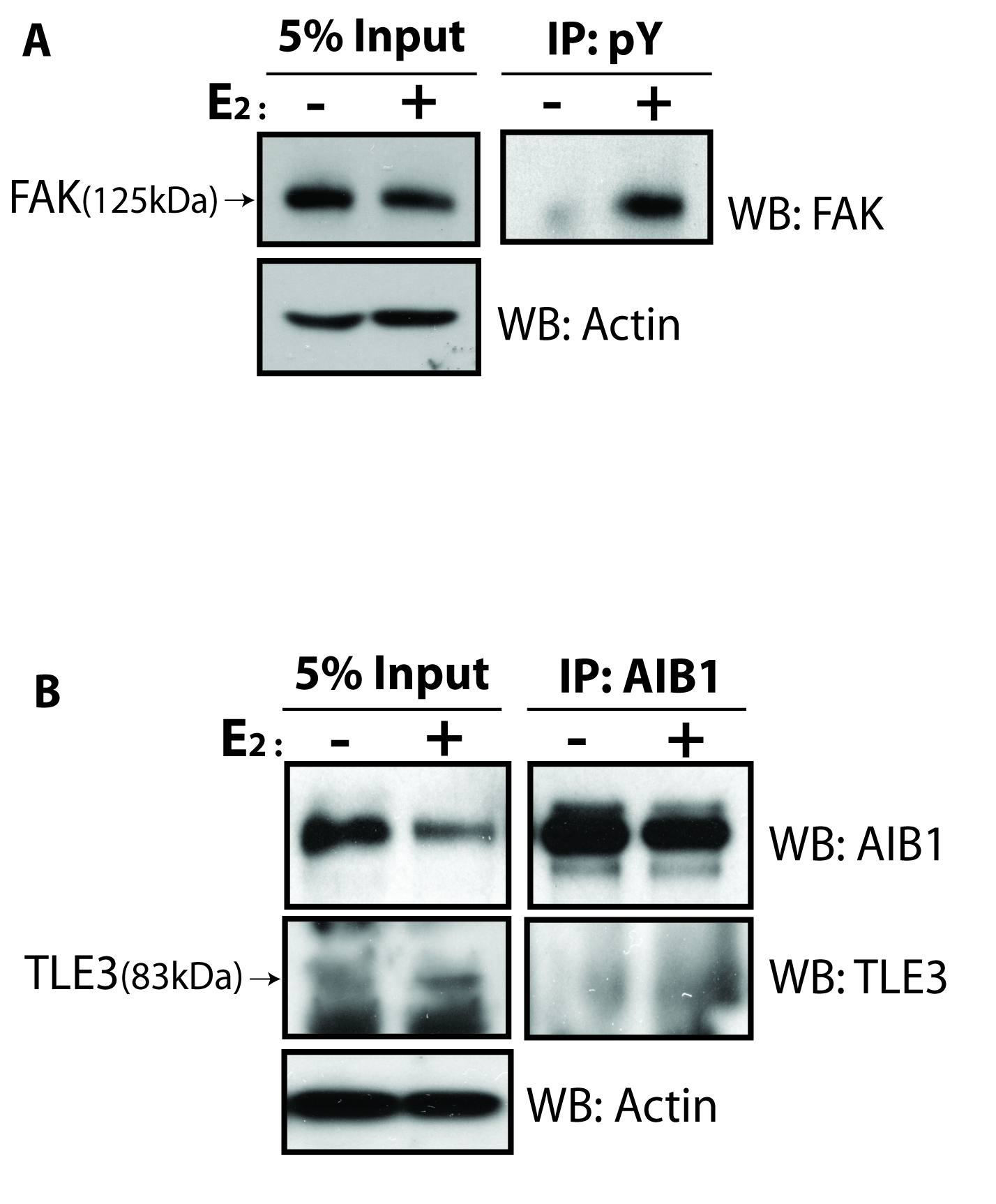

Supplement: Figure S8 — Western blot analysis confirms that FAK1 and TLE3 are immunoprecipitated from E2 treated MCF7:5C cells. MCF-7:5C cells were treated or not with E2 for 2 hours, and proteins were extracted for IP/Western analysis A) Tyrosine-phosphorylated endogenous proteins were immunoprecipitated with anti-phosphotyrosine monoclonal antibody (4G-10, Millipore) and the immunoprecipitate was resolved on SDS-PAGE followed by Western analysis. The input is 5% of the amount of total cell lysates for IP. FAK1 was detected on the blot with an anti-FAK1 antibody (A-17, Santa Cruz). B) AIB1 interacting proteins were immunoprecipitated using an anti-AIB1 monoclonal antibody (BD Biosciences). The input is 5% of the amount of total cell lysates for IP. TLE3 was detected on the blot with a TLE3 antibody (Abcam). (TIF) [file pone.0020410.s008.tif]
