# Supplementary material for: Proteomic Analysis of Pathways Involved in Estrogen-Induced Growth and Apoptosis of Breast Cancer Cells
Source: PLoS One. 2011 Jun 27;6(6):e20410. doi: 10.1371/journal.pone.0020410 (PMC3124472; doi:10.1371/journal.pone.0020410)
Supplement: Table S1 — AIB1-interacting proteins with a CI value of ≥95%. AIB1-interacting proteins (n = 58) isolated from MCF-7 and MCF-7:5C cells identified by MALDI-MS/MS with a CI value of ≥95% are listed and assigned with functional categories. The number of peptides identified and % coverage are in Table S4. Various experimental groups in which AIB1-interacting proteins were identified, are shown in the right side columns (with vertical column names), and the number of total proteins in each group is given in parenthesis. Proteins are arranged by their functional categories (see Fig. 3) and the number of proteins in each experimental group of a given category is also indicated in the same row of the category. The column furthest to the right shows AIB1-interacting proteins in this study that are also identified as part of the AIB1 protein interaction (int.) network. “X” indicates the presence of a given protein in a given experimental group or in the AIB1 interaction network. Asterisks by the protein accession indicate AIB1-interacting proteins that are also identified in pY complexes (see Table S2). (DOC) [file pone.0020410.s009.doc]

| **UniProt**  **Accession** | **Gene**  **Symbol** | **Protein Name**  **(n=58)** | **MCF-7, (-) E2 (24)** | **MCF-7, (+) E2 (35)** | **MCF-7:5C, (-) E2 (10)** | **MCF-7:5C, (+) E2 (23)** | **(-) E2, cells combined (26)** | **(+) E2, cells combined (50)** | **MCF-7 (- / + E2) (45)** | **MCF-7:5C (- / + E2) (28)** | **AIB1 int. network (18)** |
| --- | --- | --- | --- | --- | --- | --- | --- | --- | --- | --- | --- |
| **Cytoskeleton and structural proteins (8)** | | | **5** | **4** | **1** | **2** | **5** | **5** | **7** | **3** | **3** |
| P60709* | ACTB | Actin, cytoplasmic 1 |  |  |  | X |  | X |  | X | X |
| P63261* | ACTG1 | Actin, cytoplasmic 2 (in NURSA set) |  | X |  |  |  | X | X |  | X |
| Q9UPN3 | MACF1 | Microtubule-actin cross-linking factor 1, isoforms 1/2/3/5 |  | X |  |  |  | X | X |  |  |
| Q9BSJ2* | TUBGCP2 | Gamma-tubulin complex component 2 | X |  | X |  | X |  | X | X | X |
| Q9BQE3* | TUBA1C | Tubulin alpha-1C chain | X |  |  |  | X |  | X |  |  |
| Q9NY65 | TUBA8 | Tubulin alpha-8 chain | X |  |  |  | X |  | X |  |  |
| P20929 | NEB | Nebulin | X | X |  | X | X | X | X | X |  |
| P58107* | EPPK1 | Epiplakin (450 kDa epidermal antigen) | X | X |  |  | X | X | X |  |  |
| **Metabolisms (8)** | | | **3** | **4** | **3** | **6** | **3** | **8** | **5** | **6** | **2** |
| O14841 | OPLAH | 5-oxoprolinase |  |  |  | X |  | X |  | X |  |
| Q96A11 | GAL3ST3 | Galactose-3-O-sulfotransferase 3 |  |  |  | X |  | X |  | X |  |
| P04406 | GAPDH | Glyceraldehyde-3-phosphate dehydrogenase | X |  | X | X | X | X | X | X | X |
| O43708 | GSTZ1 | Maleylacetoacetate isomerase | X | X | X | X | X | X | X | X | X |
| Q9BVK2 | ALG8 | Probable dolichyl pyrophosphate Glc1Man9GlcNAc2 alpha-1,3-glucosyltransferase |  | X |  |  |  | X | X |  |  |
| P50053 | KHK | Ketohexokinase |  |  |  | X |  | X |  | X |  |
| P49327 | FASN | Fatty acid synthase |  | X |  |  |  | X | X |  |  |
| P49748 | ACADVL | Very long-chain specific acyl-CoA dehydrogenase, mitochondrial | X | X | X | X | X | X | X | X |  |
| **Transcriptional regulation (7)** | | | **1** | **2** | **0** | **6** | **1** | **7** | **3** | **6** | **2** |
| O60716 | CTNND1 | Catenin delta-1 |  | X |  |  |  | X | X |  | X |
| O94906 | PRPF6 | Pre-mRNA-processing factor 6 |  |  |  | X |  | X |  | X | X |
| Q04726 | TLE3 | Transducin-like enhancer protein 3 |  |  |  | X |  | X |  | X |  |
| Q12926 | ELAVL2 | ELAV-like protein 2 |  |  |  | X |  | X |  | X |  |
| Q9NVU0 | POLR3E | DNA-directed RNA polymerase III subunit RPC5 | X |  |  | X | X | X | X | X |  |
| Q8WUF5 | IASPP | RelA-associated inhibitor (NFkB-interacting protein 1) |  | X |  | X |  | X | X | X |  |
| Q9NQX1 | PRDM5 | PR domain zinc finger protein 5 |  |  |  | X |  | X |  | X |  |
| **Signal transduction (6)** | | | **2** | **3** | **1** | **1** | **3** | **4** | **4** | **2** | **3** |
| P62158* | CALM1 | Calmodulin |  | X |  |  |  | X | X |  | X |
| P04899 | GNAI2 | Guanine nucleotide-binding protein G(i), alpha-2 subunit | X |  |  |  | X |  | X |  |  |
| P48729 | CSNK1A1 | Casein kinase I isoform alpha |  |  | X |  | X |  |  | X | X |
| Q9BZL6 | PRKD2 | Serine/threonine-protein kinase D2 | X | X |  |  | X | X | X |  |  |
| O00750 | PIK3C2B | Phosphatidylinositol-4-phosphate 3-kinase C2 domain-containing beta polypeptide |  |  |  | X |  | X |  | X | X |
| Q86TI0 | TBC1D1 | TBC1 domain family member 1 |  | X |  |  |  | X | X |  |  |
| **Cellular motors (6)** | | | **2** | **5** | **0** | **1** | **2** | **6** | **5** | **1** | **2** |
| O43795* | MYO1B | Myosin-Ib |  | X |  |  |  | X | X |  |  |
| O00159* | MYO1C | Myosin-Ic | X | X |  |  | X | X | X |  |  |
| Q8NEV4 | MYO3A | Myosin IIIA |  | X |  |  |  | X | X |  |  |
| Q9UKX2 | MYH2 | Myosin-2 |  |  |  | X |  | X |  | X |  |
| P60660 | MYL6 | Myosin light polypeptide 6 |  | X |  |  |  | X | X |  | X |
| P35579* | MYH9 | Myosin-9 | X | X |  |  | X | X | X |  | X |
| **Ribosomal proteins (5)** | | | **1** | **5** | **2** | **1** | **2** | **5** | **5** | **2** | **1** |
| P62244 | RPS15A | 40S ribosomal protein S15a |  | X | X |  | X | X | X | X |  |
| P62269* | RPS18 | 40S ribosomal protein S18 | X | X | X | X | X | X | X | X |  |
| P23396 | RPS3 | 40S ribosomal protein S3 |  | X |  |  |  | X | X |  | X |
| P62701 | RPS4X | 40S ribosomal protein S4, X isoform |  | X |  |  |  | X | X |  |  |
| P62888 | RPL30 | 60S ribosomal protein L30 |  | X |  |  |  | X | X |  |  |
| **Heat shock proteins (3)** | | | **3** | **3** | **2** | **2** | **3** | **3** | **3** | **3** | **3** |
| P07900 | HSP90AA1 | Heat shock protein HSP 90-alpha | X | X |  | X | X | X | X | X | X |
| P08107* | HSPA1B | Heat shock 70 kDa protein 1 | X | X | X |  | X | X | X | X | X |
| P38646* | HSPA9 | Stress-70 protein, mitochondrial | X | X | X | X | X | X | X | X | X |
| **Chromatin complex (2)** | | | **2** | **1** | **0** | **1** | **2** | **1** | **2** | **1** | **2** |
| Q9Y4A5 | TRRAP | Transformation/transcription domain-associated protein | X |  |  |  | X |  | X |  | X |
| Q9ULD4 | BRPF3 | Bromodomain and PHD finger-containing protein 3 | X | X |  | X | X | X | X | X | X |
| **RNA metabolic process (2)** | | | **0** | **1** | **0** | **1** | **0** | **2** | **1** | **1** | **0** |
| Q86U44 | METTL3 | N6-adenosine-methyltransferase 70 kDa subunit |  | X |  |  |  | X | X |  |  |
| Q9HAU5 | UPF2 | Regulator of nonsense transcripts 2 |  |  |  | X |  | X |  | X |  |
| **Cell adhesion (2)** | | | **1** | **2** | **0** | **0** | **1** | **2** | **2** | **0** | **0** |
| O14917 | PCDH17 | Protocadherin-17 | X | X |  |  | X | X | X |  |  |
| Q9Y446 | PKP3 | Plakophilin-3 |  | X |  |  |  | X | X |  |  |
| **Membrane and protein trafficking (2)** | | | **2** | **0** | **1** | **1** | **1** | **1** | **2** | **2** | **0** |
| O75131 | CPNE3 | Copine-3 (with possible kinase activity) | X |  | X |  | X |  | X | X |  |
| Q92738 | USP6NL | USP6 N-terminal-like protein (GTPase-activating protein) | X |  |  | X |  | X | X | X |  |
| **Cell cycle (1)** | | | **0** | **1** | **0** | **0** | **0** | **1** | **1** | **0** | **0** |
| Q15019 | Sep-2 | Septin-2 |  | X |  |  |  | X | X |  |  |
| **Apoptosis regulation (1)** | | | **1** | **0** | **0** | **0** | **1** | **0** | **1** | **0** | **0** |
| Q9BXL7 | CARD11 | Caspase recruitment domain-containing protein 11 | X |  |  |  | X |  | X |  |  |
| **Developmental process (1)** | | | **1** | **1** | **0** | **0** | **1** | **1** | **1** | **0** | **0** |
| O75094 | SLIT3 | Slit homolog 3 protein | X | X |  |  | X | X | X |  |  |
| **Neurological system process (1)** | | | **0** | **0** | **0** | **1** | **0** | **1** | **0** | **1** | **0** |
| Q8WUJ3 | KIAA1199 | Protein KIAA1199 |  |  |  | X |  | X |  | X |  |
| **Protein folding (1)** | | | **0** | **1** | **0** | **0** | **0** | **1** | **1** | **0** | **0** |
| P62937* | PPIA | Peptidyl-prolyl cis-trans isomerase A (Cyclophilin A) |  | X |  |  |  | X | X |  |  |
| **Prostate cancer (1)** | | | **0** | **1** | **0** | **0** | **0** | **1** | **1** | **0** | **0** |
| Q7Z407 | CSMD3 | CUB and sushi domain-containing protein 3 |  | X |  |  |  | X | X |  |  |
| **Autophage (1)** | | | **0** | **1** | **0** | **0** | **0** | **1** | **1** | **0** | **0** |
| Q92562 | FIG4 | SAC domain-containing protein 3 |  | X |  |  |  | X | X |  |  |
