# Supplementary material for: Proteomic Analysis of Pathways Involved in Estrogen-Induced Growth and Apoptosis of Breast Cancer Cells
Source: PLoS One. 2011 Jun 27;6(6):e20410. doi: 10.1371/journal.pone.0020410 (PMC3124472; doi:10.1371/journal.pone.0020410)
Supplement: Table S2 — Phosphotyrosine complexed proteins with a CI value of ≥95%. Proteins pulled down with anti-pY in MCF-7 and MCF-7:5C cells identified from MALDI-MS/MS with a CI value of ≥95% are listed and assigned with functional categories. The number of peptides identified and % coverage are in Table S5. Various experimental groups in which tyrosine-phosphorylated proteins are identified are shown in the right columns (with vertical column names), and the number of proteins in each group is given in parenthesis. Proteins are arranged by their functional categories (see Fig. 3) and the number of proteins in each experimental group of a given category is also indicated in the same row of the category. “X” indicates the presence of a given protein in a given experimental group or in the AIB1 interaction network. Asterisks by the protein accession indicate IP-pY complexes that are also identified as AIB1-interacting (see Table S1). (DOC) [file pone.0020410.s010.doc]

| **UniProt**  **Accession** | **Gene**  **Symbol** | **Protein Name**  **(n=56)** | **MCF-7, (-) E2 (18)** | **MCF-7, (+) E2 (23)** | **MCF-7:5C, (-) E2 (17)** | **MCF-7:5C, (+) E2 (20)** | **(-) E2, cells combined (31)** | **(+) E2, cells combined (38)** | **MCF-7 (- / + E2) (35)** | **MCF-7:5C (- / + E2) (30)** | **AIB1 int. network (14)** |
| --- | --- | --- | --- | --- | --- | --- | --- | --- | --- | --- | --- |
| **Cytoskeleton and structural proteins (14)** | | | **4** | **4** | **8** | **6** | **10** | **9** | **7** | **10** | **4** |
| O43707 | ACTN4 | Alpha-actinin-4 |  |  | X | X | X | X |  | X | X |
| P60709* | ACTB | Actin, cytoplasmic 1 | X |  |  |  | X |  | X |  | X |
| P63261* | ACTG1 | Actin, cytoplasmic 2 | X | X | X |  | X | X | X | X | X |
| Q562R1 | ACTBL2 | Beta-actin-like protein 2 | X |  |  |  | X |  | X |  |  |
| Q96PK2 | MACF1 | Microtubule-actin cross-linking factor 1, isoform 4 |  |  | X | X | X | X |  | X |  |
| O14639 | ABLIM1 | Actin-binding LIM protein 1 |  |  | X |  | X |  |  | X |  |
| Q96JY6 | PDLIM2 | PDZ and LIM domain protein 2 |  |  |  | X |  | X |  | X |  |
| Q13813 | SPTAN1 | Spectrin alpha chain, brain |  |  | X | X | X | X |  | X |  |
| O15020 | SPTBN2 | Spectrin beta chain, brain 2 |  |  | X |  | X |  |  | X |  |
| P58107* | EPPK1 | Epiplakin |  | X | X | X | X | X | X | X |  |
| Q9BQE3* | TUBA1C | Tubulin alpha-1C chain |  | X |  |  |  | X | X |  |  |
| Q9BSJ2* | TUBGCP2 | Gamma-tubulin complex component 2 | X |  | X |  | X |  | X | X | X |
| Q9NSV4 | DIAPH3 | Protein diaphanous homolog 3 |  | X |  |  |  | X | X |  |  |
| Q96M96 | FGD4 | FYVE, RhoGEF and PH domain-containing protein 4 |  |  |  | X |  | X |  | X |  |
| **Transcriptional regulation (6)** | | | **2** | **2** | **1** | **2** | **3** | **4** | **4** | **3** | **0** |
| Q16531 | DDB1 | DNA damage-binding protein 1 |  | X |  |  |  | X | X |  |  |
| P52740 | ZNF132 | Zinc finger protein 132 |  |  |  | X |  | X |  | X |  |
| Q14929 | ZNF169 | Zinc finger protein 169 | X |  |  |  | X |  | X |  |  |
| Q9ULJ3 | ZNF295 | Zinc finger protein 295 |  | X |  |  |  | X | X |  |  |
| Q14585 | ZNF345 | Zinc finger protein 345 |  |  | X |  | X |  |  | X |  |
| Q4V348 | ZNF658B | Zinc finger protein 658B | X |  |  | X | X | X | X | X |  |
| **Protein transport and vesicle trafficking (6)** | | | **0** | **2** | **2** | **2** | **2** | **4** | **2** | **4** | **1** |
| P27824 | CANX | Calnexin |  |  |  | X |  | X |  | X |  |
| P53621 | COPA | Coatomer subunit alpha |  | X |  |  |  | X | X |  |  |
| P53618 | COPB1 | Coatomer subunit beta |  | X |  |  |  | X | X |  |  |
| Q13439 | GOLGA4 | Golgin subfamily A member 4 |  |  | X |  | X |  |  | X |  |
| Q14257 | RCN2 | Reticulocalbin-2 |  |  | X |  | X |  |  | X |  |
| P55060 | CSE1L | Exportin-2 |  |  |  | X |  | X |  | X | X |
| **Signal transduction (5)** | | | **2** | **2** | **0** | **2** | **2** | **4** | **3** | **2** | **3** |
| Q92736 | RYR2 | Ryanodine receptor 2 |  | X |  |  |  | X | X |  |  |
| P62158* | CALM1 | Calmodulin | X |  |  |  | X |  | X |  | X |
| Q16539 | MAPK14 | Mitogen-activated protein kinase 14 | X | X |  |  | X | X | X |  | X |
| Q05397 | PTK2 | Focal adhesion kinase 1 = FAK1 |  |  |  | X |  | X |  | X | X |
| P26045 | PTPN3 | Tyrosine-protein phosphatase non-receptor type 3 |  |  |  | X |  | X |  | X |  |
| **Ribosomal proteins (5)** | | | **2** | **4** | **2** | **1** | **3** | **4** | **5** | **2** | **0** |
| P62847 | RPS24 | 40S ribosomal protein S24 | X | X | X |  | X | X | X | X |  |
| P46783 | RPS10 | 40S ribosomal protein S10 |  | X |  |  |  | X | X |  |  |
| P62269* | RPS18 | 40S ribosomal protein S18 |  | X |  |  |  | X | X |  |  |
| P30050 | RPL12 | 60S ribosomal protein L12 |  | X | X | X | X | X | X | X |  |
| P05388 | RPLP0 | 60S acidic ribosomal protein P0 | X |  |  |  | X |  | X |  |  |
| **Cell cycle (3)** | | | **0** | **0** | **0** | **3** | **0** | **3** | **0** | **3** | **2** |
| P06493 | CDC2 | Cell division control protein 2 homolog |  |  |  | X |  | X |  | X | X |
| Q15021 | NCAPD2 | Condensin complex subunit 1 |  |  |  | X |  | X |  | X |  |
| Q8IZT6 | ASPM | Abnormal spindle-like microcephaly-associated protein |  |  |  | X |  | X |  | X | X |
| **Cellular motors (3)** | | | **3** | **1** | **0** | **0** | **3** | **1** | **3** | **0** | **1** |
| O43795* | MYO1B | Myosin-Ib | X |  |  |  | X |  | X |  |  |
| O00159* | MYO1C | Myosin-Ic | X |  |  |  | X |  | X |  |  |
| P35579* | MYH9 | Myosin-9 | X | X |  |  | X | X | X |  | X |
| **Heat shock proteins (3)** | | | **1** | **2** | **2** | **3** | **2** | **3** | **2** | **3** | **2** |
| P08107* | HSPA1B | Heat shock 70 kDa protein 1 | X | X | X | X | X | X | X | X | X |
| P17066 | HSPA6 | Heat shock 70 kDa protein 6 |  | X |  | X |  | X | X | X |  |
| P38646* | HSPA9 | Stress-70 protein, mitochondrial |  |  | X | X | X | X |  | X | X |
| **Proteolysis and regulation (2)** | | | **0** | **1** | **2** | **0** | **2** | **1** | **1** | **2** | **0** |
| Q9UL46 | PSME2 | Proteasome activator complex subunit 2 (Ag processing) |  | X | X |  | X | X | X | X |  |
| Q13219 | PAPPA | Pappalysin-1 |  |  | X |  | X |  |  | X |  |
| **Protein translation (2)** | | | **0** | **2** | **0** | **0** | **0** | **2** | **2** | **0** | **1** |
| P57772 | EEFSEC | Selenocysteine-specific elongation factor |  | X |  |  |  | X | X |  |  |
| Q92616 | GCN1L1 | Translational activator GCN1 |  | X |  |  |  | X | X |  | X |
| **Metabolism (1)** | | | **0** | **1** | **0** | **0** | **0** | **1** | **1** | **0** | **0** |
| P29728 | OAS2 | 2'-5'-oligoadenylate synthetase 2 |  | X |  |  |  | X | X |  |  |
| **Apoptosis (1)** | | | **0** | **0** | **0** | **1** | **0** | **1** | **1** | **1** | **1** |
| Q8N163 | KIAA1967 | Protein KIAA1967 (Deleted in breast cancer gene 1 protein) (DBC1) |  |  |  | X |  | X |  | X | X |
| **Protein folding (1)** | | | **1** | **1** | **0** | **0** | **1** | **1** | **1** | **0** | **0** |
| P62937* | PPIA | Peptidyl-prolyl cis-trans isomerase A (Cyclophilin A) | X | X |  |  | X | X | X |  |  |
| **Cell adhesion (1)** | | | **1** | **0** | **0** | **0** | **1** | **0** | **1** | **0** | **0** |
| Q96RW7 | HMCN1 | Hemicentin-1 | X |  |  |  | X |  | X |  |  |
| **Retroviral envelope (1)** | | | **1** | **0** | **0** | **0** | **1** | **0** | **1** | **0** | **0** |
| P61568 | - | HERV-K_1p13.3 provirus ancestral Env polyprotein | X |  |  |  | X |  | X |  |  |
| **Unknown (2)** | | | **1** | **1** | **0** | **0** | **1** | **1** | **2** | **0** | **0** |
| Q96QE4 | LRRC37B | Leucine-rich repeat-containing protein 37B |  | X |  |  |  | X | X |  |  |
| Q6IN97 | FRMPD2L2 | Putative protein FRMPD2-like | X |  |  |  | X |  | X |  |  |
