# Supplementary material for: Proteomic Analysis of Pathways Involved in Estrogen-Induced Growth and Apoptosis of Breast Cancer Cells
Source: PLoS One. 2011 Jun 27;6(6):e20410. doi: 10.1371/journal.pone.0020410 (PMC3124472; doi:10.1371/journal.pone.0020410)
Supplement: Table S3 — Pathway mapping of proteins identified with a CI<95%. Proteins are listed alphabetically based on the “gene name” column for anti-AIB1 or anti-pY immunoprecipitated proteins. In the “Experiment” column A to D indicate: A, MCF-7 cells, no E2; B, MCF-7:5C cells, no E2; C, MCF-7 cells, +E2; and D, MCF-7:5C cells, +E2. The “Spec” column references the corresponding mass spectrum for single peptide MS/MS identification in the section “Single peptide spectral data” appended at the end of this table. The “Figures” column indicates in which figure(s) the proteins are depicted, except for a few only discussed in the main text (text). In the spectral data section, the underlined C and M in “peptide sequences” column represent fixed (carbamidomethyl) and variable (oxidation) modifications, respectively. *MALDI-TOF-MS generates peptides containing only one charge, and the precursor m/z is thus equal to the precursor mass. NA, not available. (DOC) [file pone.0020410.s011.doc]

| **UniProtKB AC** | | **Gene Name** | | **Experiment** | | **Score** | | **CI%** | | **# peptides** | **Coverage (%)** | **MS** | | **Spec.** | **Figure** | |
| --- | --- | --- | --- | --- | --- | --- | --- | --- | --- | --- | --- | --- | --- | --- | --- | --- |
| P20749 | | BCL3 | | AIB1_C | | 12 | | 21 | | 1 | NA | MS/MS | | S1 | Fig. S3C | |
| P33151 | | CDH5 | | AIB1_C | | 21 | | 84 | | 1 | NA | MS/MS | | S2 | Fig. S4C | |
| P48729 | | CSNK1A1 | | AIB1_B | | 49 | | 85 | | 10 | 28 | MS | |  | Fig. S4B | |
| P48730 | | CSNK1D (CK1d) | | AIB1_C | | 17 | | 64 | | 1 | NA | MS/MS | | S3 | Fig. S4C | |
| P35222 | | CTNNB1 | | AIB1_B | | 6 | | 11 | | 1 | NA | MS/MS | | S4 | Fig. S4B | |
| P35222 | | CTNNB1 | | AIB1_D | | 10 | | 17 | | 1 | NA | MS/MS | | S5 | Fig. S4D | |
| P63092 | | GNAS | | AIB1_C | | 14 | | 23 | | 1 | NA | MS/MS | | S6 | Fig. 4 & S2 | |
| P49841 | | GSK3B | | AIB1_C | | 18 | | 61 | | 1 | NA | MS/MS | | S7 | Fig. 4, S3 & S4 | |
| Q14573 | | ITPR3 | | AIB1_C | | 23 | | 88 | | 1 | NA | MS/MS | | S8 | Fig. S1 | |
| Q14573 | | ITPR3 | | AIB1_D | | 50 | | 87 | | 24 | 10 | MS | |  | Fig. S1 | |
| Q07954 | | LRP1 | | AIB1_A | | 14 | | 28 | | 2 | 1 | MS/MS | |  | Fig. S4A | |
| O75581 | | LRP6 | | AIB1_B | | 14 | | 10 | | 1 | NA | MS/MS | | S9 | Fig. S4B | |
| Q04721 | | Notch2 | | AIB1_A | | 16 | | 48 | | 1 | NA | MS/MS | | S10 | *text* | |
| Q9UM47 | | Notch3 | | AIB1_C | | 20 | | 88 | | 1 | NA | MS/MS | | S11 | *text* | |
| Q9Y6R0 | | NUMBL | | AIB1_C | | 17 | | 62 | | 1 | NA | MS/MS | | S12 | *text* | |
| P42338 | | PIK3CB | | AIB1_B | | 9 | | 20 | | 1 | NA | MS/MS | | S13 | Fig. S2 & S3 | |
| O00329 | | PIK3CD | | AIB1_C | | 16 | | 58 | | 1 | NA | MS/MS | | S14 | Fig. S2 & S3 | |
| Q92569 | | PIK3R3 | | AIB1_C | | 15 | | 41 | | 1 | NA | MS/MS | | S15 | Fig. S2 | |
| Q9NQ66 | | PLCB1 | | AIB1_B | | 16 | | 40 | | 1 | NA | MS/MS | | S16 | Fig. S1 | |
| Q9NQ66 | | PLCB1 | | AIB1_C | | 18 | | 63 | | 1 | NA | MS/MS | | S17 | Fig. S1 | |
| Q9NQ66 | | PLCB1 | | AIB1_D | | 18 | | 59 | | 1 | NA | MS/MS | | S18 | Fig. S1 | |
| O15297 | | PP2C/WIP1 | | AIB1_A | | 8 | | 39 | | 1 | NA | MS/MS | | S19 | Fig. S2 | |
| P16298 | | PPP3CB | | AIB1_C | | 15 | | 80 | | 1 | NA | MS/MS | | S20 | Fig. S2 | |
| Q02156 | | PRKCE | | AIB1_B | | 42 | | 22 | | 11 | 22 | MS | |  | Fig. S1 | |
| P61224 | | RAP1B | | AIB1_C | | 15 | | 54 | | 1 | NA | MS/MS | | S21 | Fig. 4 | |
| P47736 | | RAP1GAP | | AIB1_D | | 21 | | 89 | | 1 | NA | MS/MS | | S22 | Fig. 4 & S1 | |
| Q15418 | | RPS6KA1 (RSK1) | | AIB1_C | | 16 | | 39 | | 1 | NA | MS/MS | | S23 | Fig. S2 | |
| Q15349 | | RPS6KA2 (RSK2) | | AIB1_D | | 12 | | 41 | | 1 | NA | MS/MS | | S24 | Fig. S2 | |
| P10301 | | RRAS | | AIB1_B | | 14 | | 51 | | 1 | NA | MS/MS | | S25 | Fig. S1 | |
| P62070 | | RRAS2 | | AIB1_D | | 44 | | 49 | | 7 | 37 | MS | |  | Fig. S3 | |
| P29597 | | TYK2 | | AIB1-A | | 15 | | 44 | | 1 | NA | MS/MS | | S26 | Fig. 4 & S3 | |
| P29597 | | TYK2 | | AIB1_B | | 14 | | 49 | | 1 | NA | MS/MS | | S27 | Fig. 4 & S3 | |
| P29597 | | TYK2 | | AIB1_C | | 17 | | 83 | | 1 | NA | MS/MS | | S28 | Fig. 4 & S3 | |
| P29597 | | TYK2 | | AIB1_D | | 22 | | 88 | | 1 | NA | MS/MS | | S29 | Fig. 4 & S3 | |
| P56705 | | WNT4 | | AIB1_B | | 49 | | 84 | | 6 | 28 | MS | |  | Fig. S4B | |
| O00755 | | WNT7A | | AIB1_C | | 17 | | 77 | | 1 | NA | MS/MS | | S30 | Fig. 4 & S4C | |
| Q9Y297 | | BTRCP | | pY_B | | 14 | | 22 | | 1 | NA | MS/MS | | S31 | Fig. S4 | |
| Q9UJ99 | | CDH22 | | pY_D | | 52 | | 92 | | 7 | 13 | MS | |  | Fig. 4 & S4 | |
| P09471 | | GNAO1/GNAO2 | | pY_D | | 17 | | 57 | | 2 | 10 | MS/MS | |  | Fig. 4 & S4 | |
| O75084 | | FZD7 | | pY_D | | 21 | | 82 | | 2 | 5 | MS/MS | |  | Fig. 4 & S4 | |
| Q13547 | | HDAC1 | | pY_C | | 47 | | 78 | | 7 | 21 | MS | |  | Fig. S4C | |
| P82979 | | CIP29/HCC1 | | pY_D | | 52 | | 93 | | 8 | 55 | MS | |  | Fig. 4 | |
| Q02156 | | PRKCE | | pY_A | | 54 | | 93 | | 13 | 26 | MS | |  | Fig. S1 | |
| Q9BT81 | | SOX7 | | pY_B | | 15 | | 37 | | 1 | NA | MS/MS | | S32 | Fig. S4 | |
| P36897 | | TGFBR1 | | pY_D | | 15 | | 51 | | 1 | NA | MS/MS | | S33 | Fig. S4 | |
| **Single peptide spectral data (for above proteins from MS/MS)** | | | | | | | | | | | | | | | | |
| **No.** | **UniProtKB AC** | | **Gene name** | | **Score** | | **CI%** | | **Peptide sequence** | | | | **Precursor mass*** | | | **Exp.** |
|  | | | | | | | | | | | | | | | | |
| S1 | P20749 | | BCL3 | | 12 | | 21 | | GADIDAVDIKSGR | | | | 1316.7 | | | AIB1_C |
| 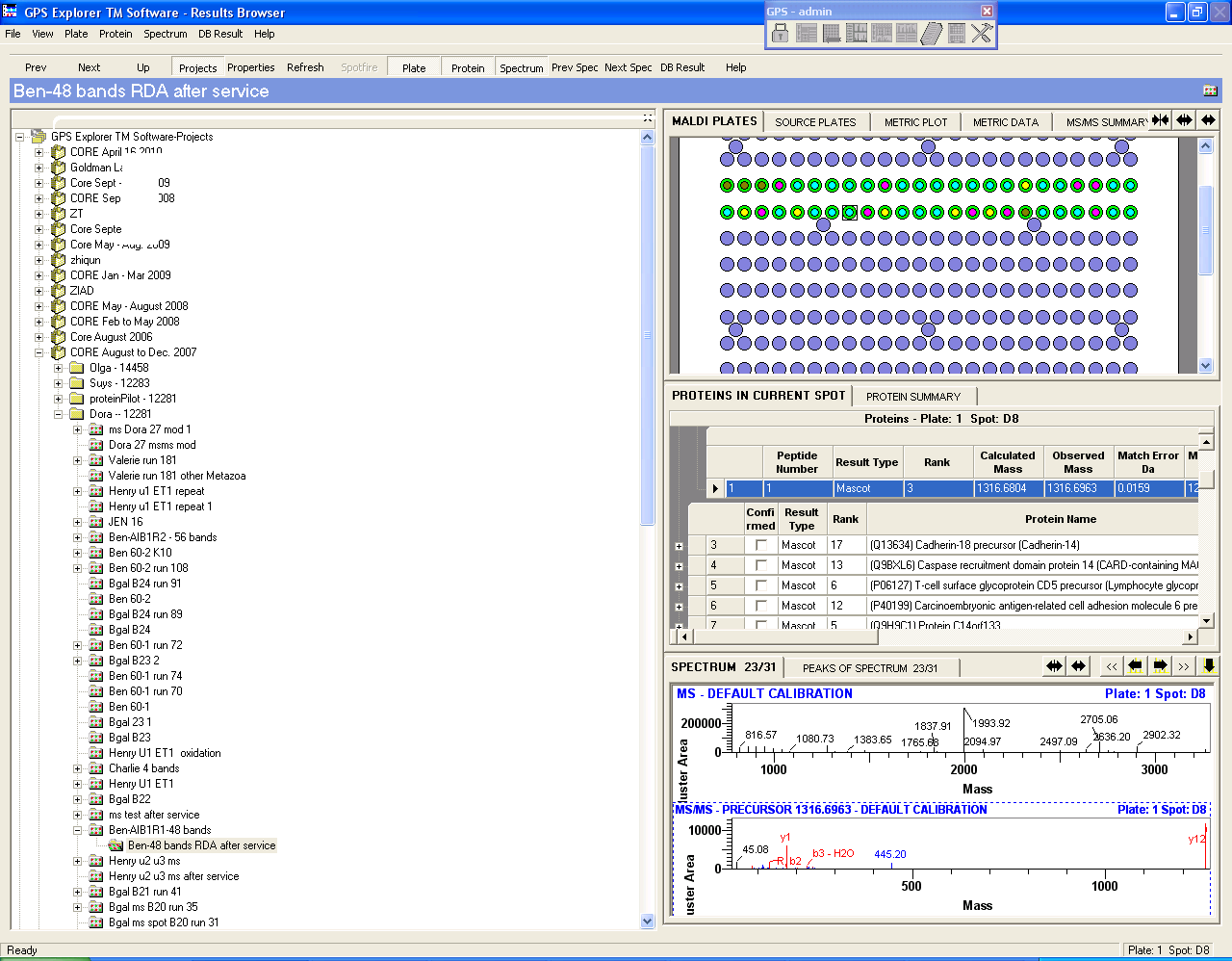 | | | | | | | | | | | | | | | | |
| S2 | P33151 | | CDH5 | | 21 | | 84 | | FKMLAELYGSDPR | | | | 1542.82 | | | AIB1_C |
| 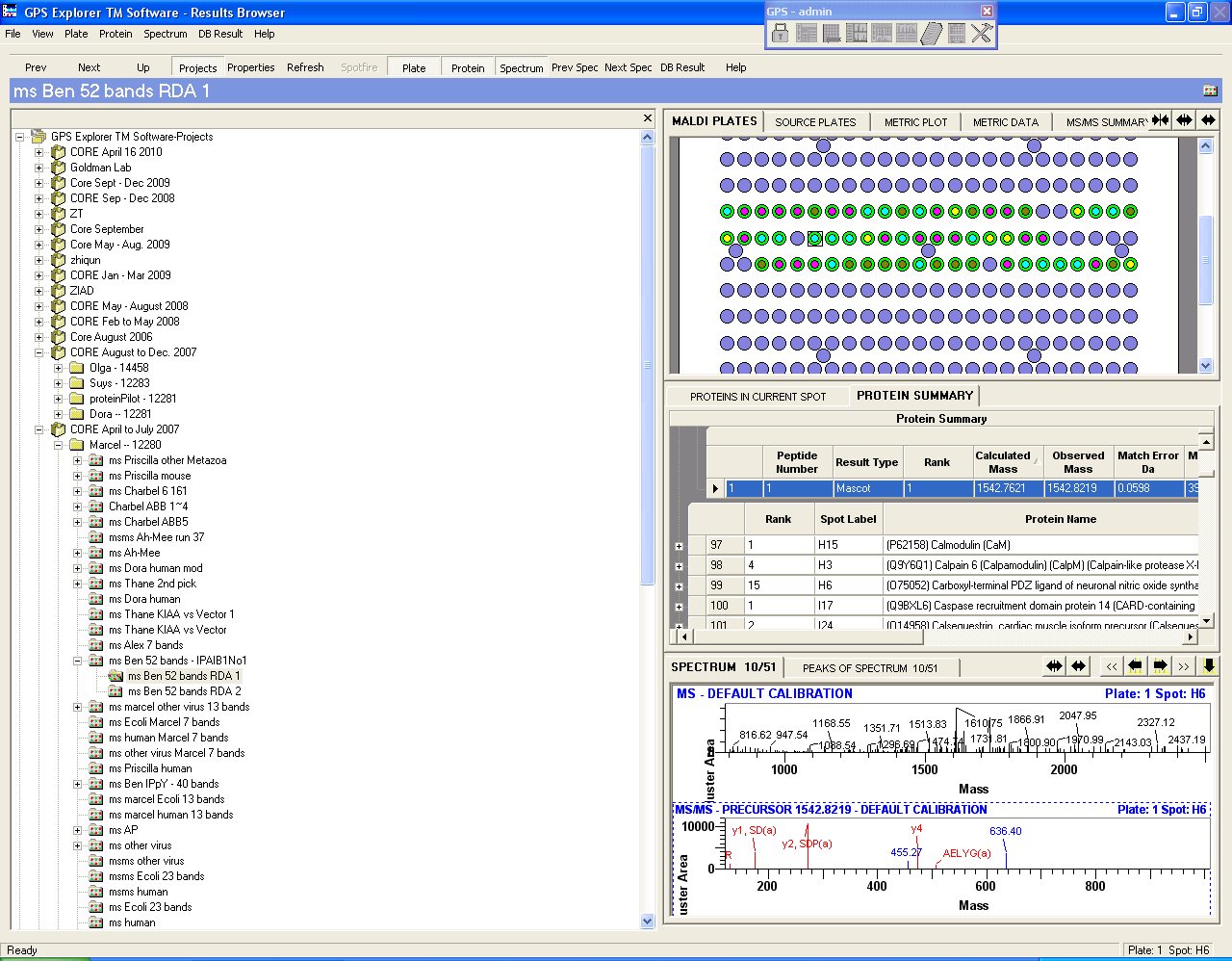 | | | | | | | | | | | | | | | | |
| S3 | P48730 | | CSNK1D | | 17 | | 64 | | QDTSRMSTSQIPGR | | | | 1579.79 | | | AIB1_C |
| 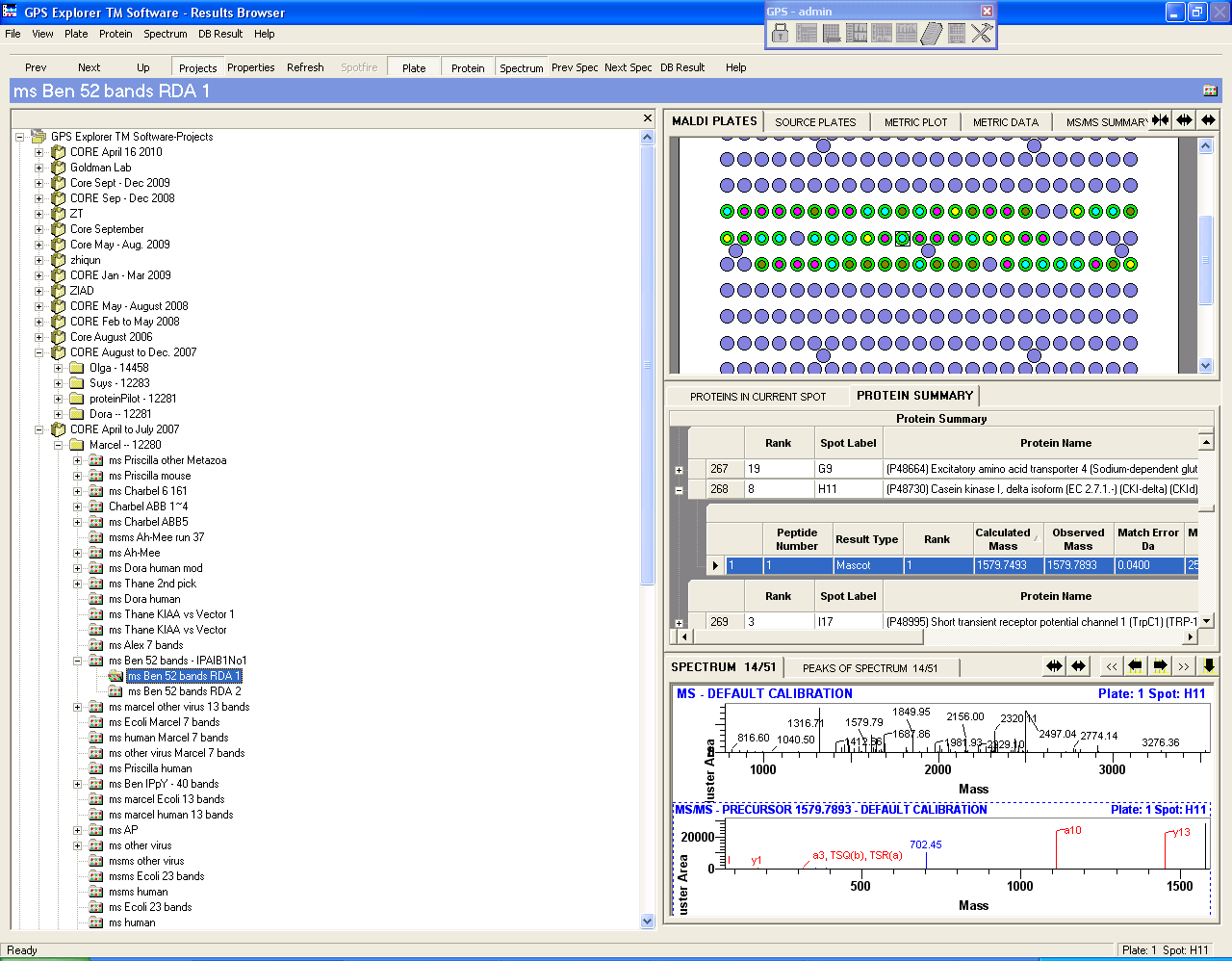 | | | | | | | | | | | | | | | | |
| S4 | P35222 | | CTNNB1 | | 6 | | 11 | | HLTSRHQEAEMAQNAVR | | | | 1993.92 | | | AIB1_B |
| 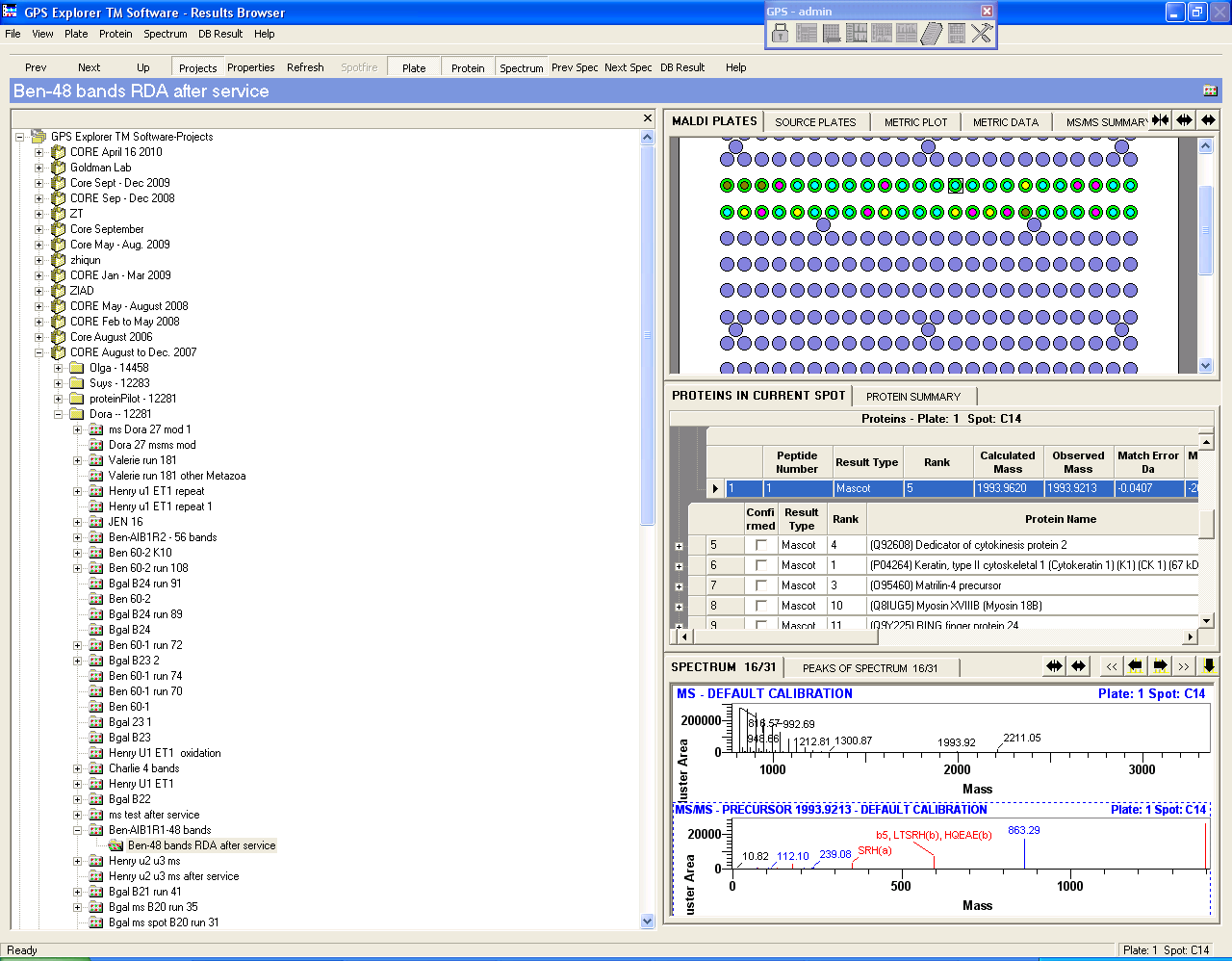 | | | | | | | | | | | | | | | | |
| S5 | P35222 | | CTNNB1 | | 10 | | 17 | | HLTSRHQEAEMAQNAVR | | | | 1993.9 | | | AIB1_D |
| 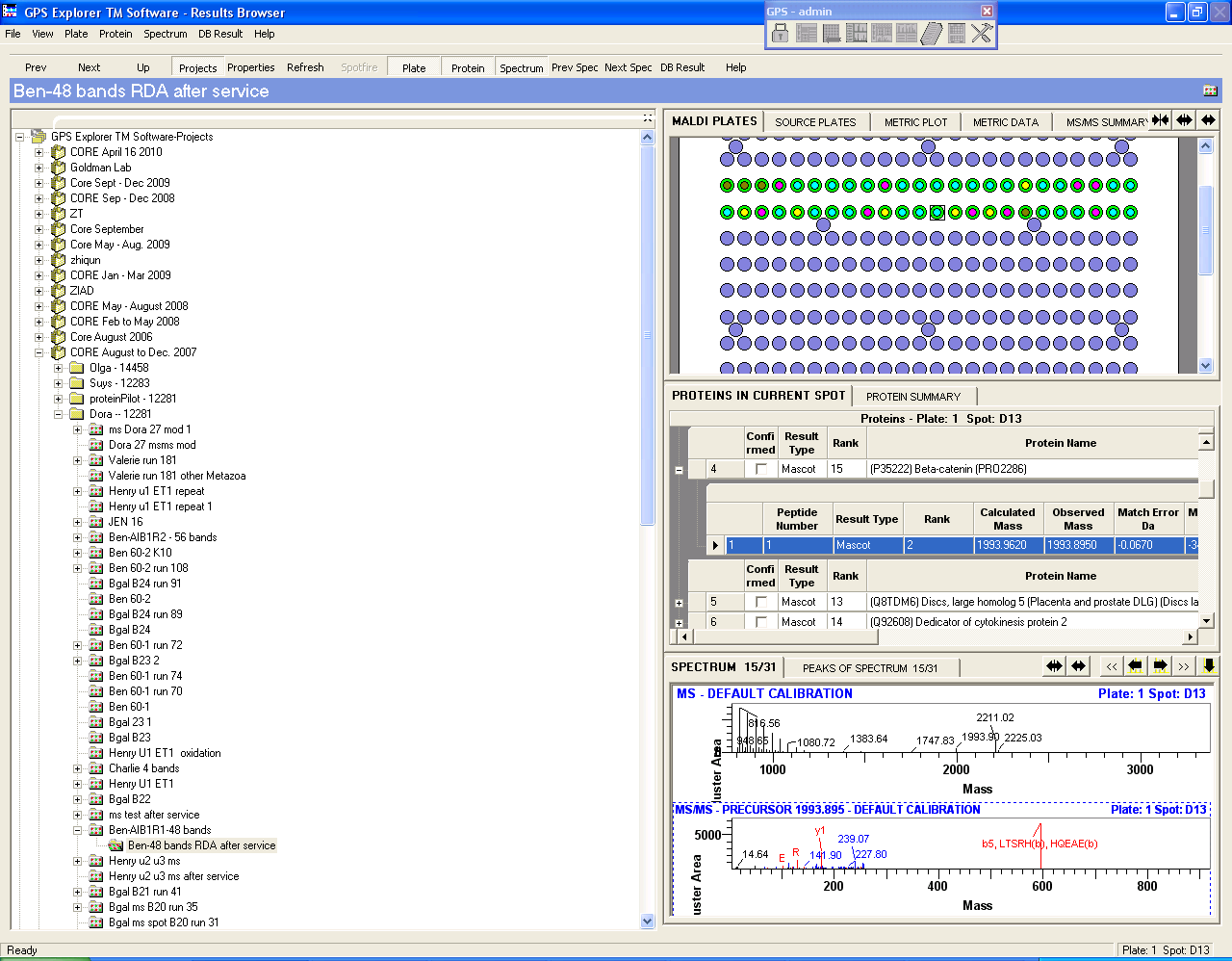 | | | | | | | | | | | | | | | | |
| S6 | P63092 | | GNAS | | 14 | | 23 | | DIIQRMHLR | | | | 1197.68 | | | AIB1_C |
| 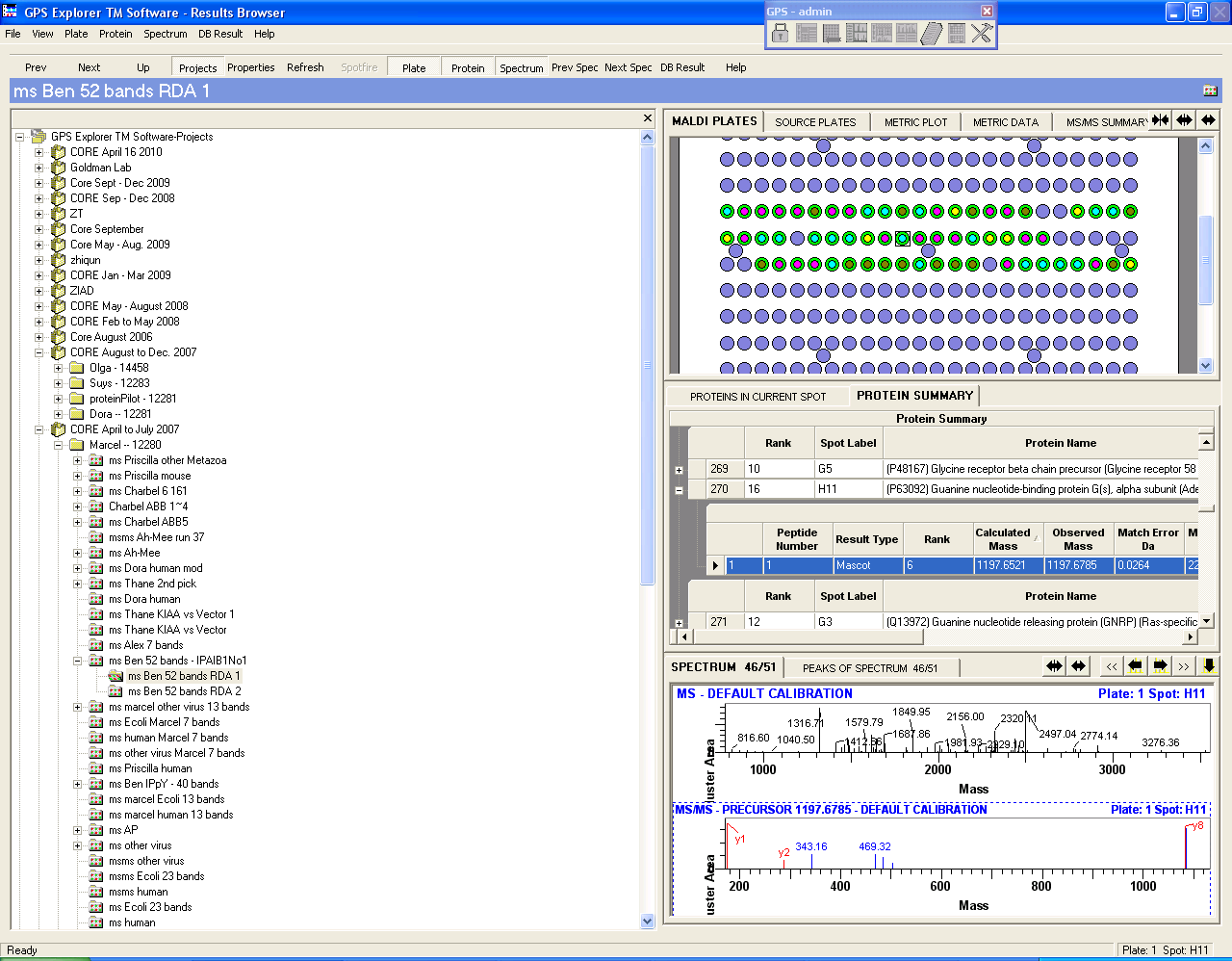 | | | | | | | | | | | | | | | | |
| S7 | P49841 | | GSK3B | | 18 | | 61 | | TTSFAESCKPVQQPSAFGSMKVSR | | | | 2589.32 | | | AIB1_C |
| 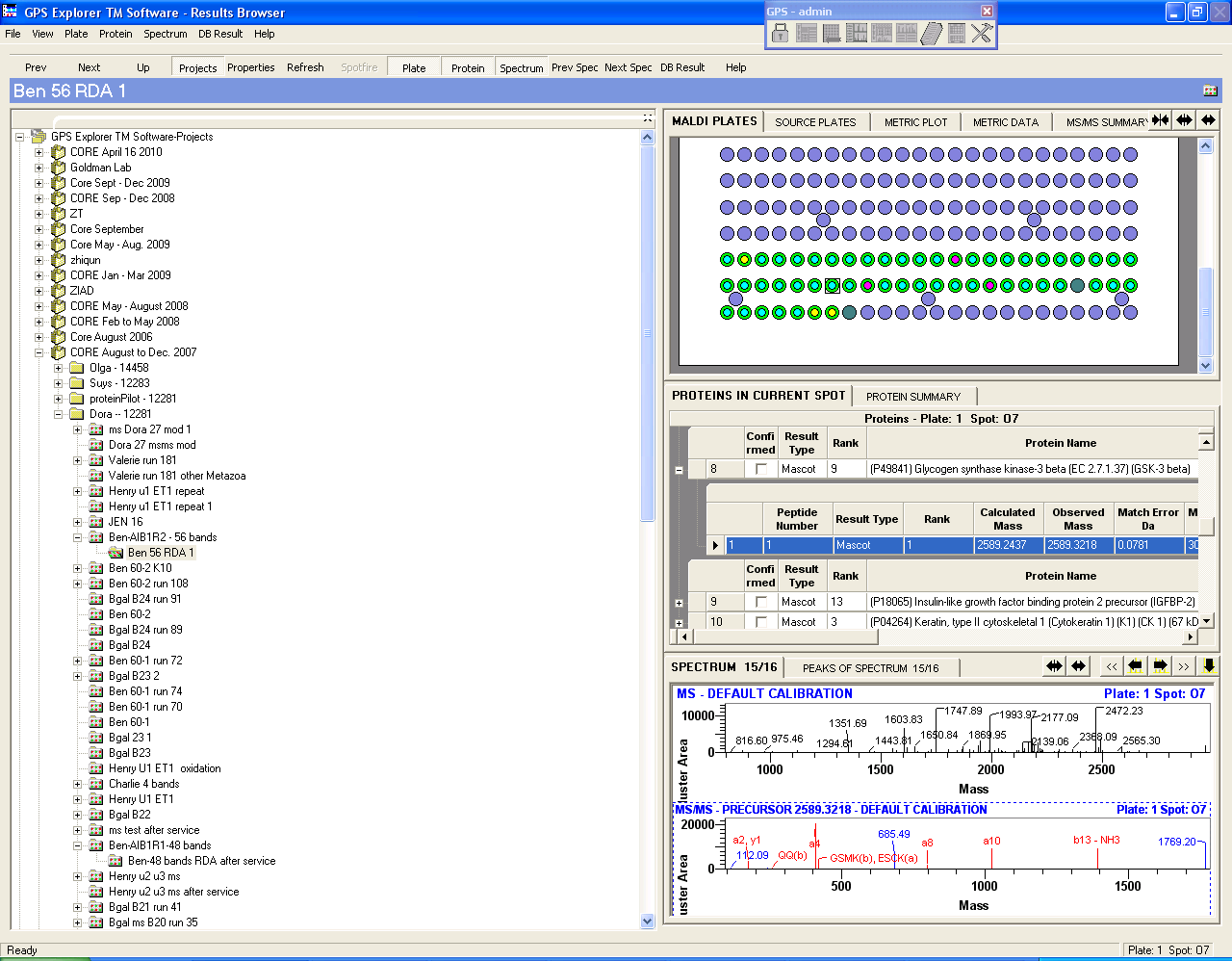 | | | | | | | | | | | | | | | | |
| S8 | Q14573 | | ITPR3 | | 23 | | 88 | | EDKEAFAIVSVPVSEIR | | | | 1888.97 | | | AIB1_C |
| 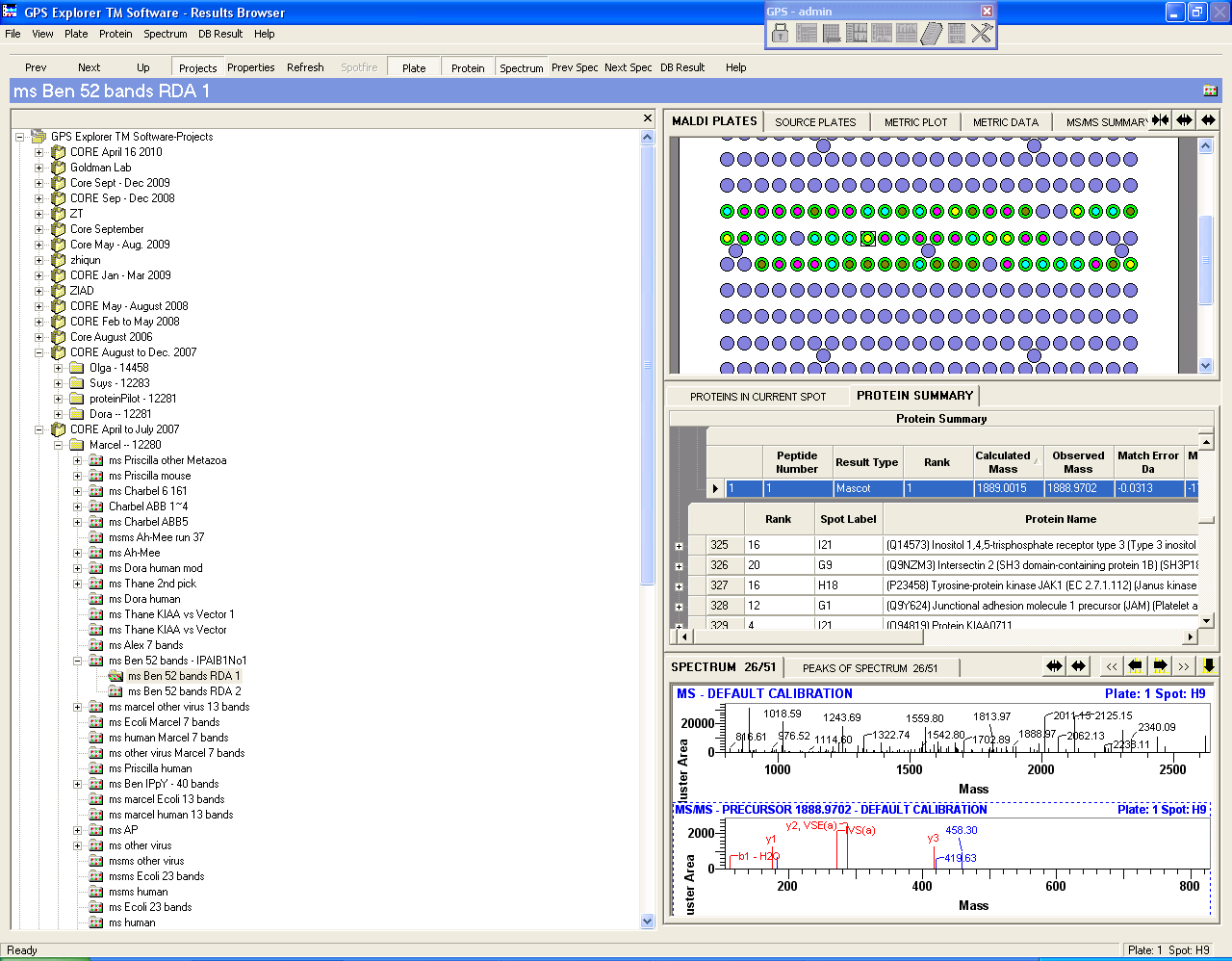 | | | | | | | | | | | | | | | | |
| S9 | O75581 | | LRP6 | | 14 | | 10 | | KVLFWQELDQPR | | | | 1558.84 | | | AIB1_B |
| 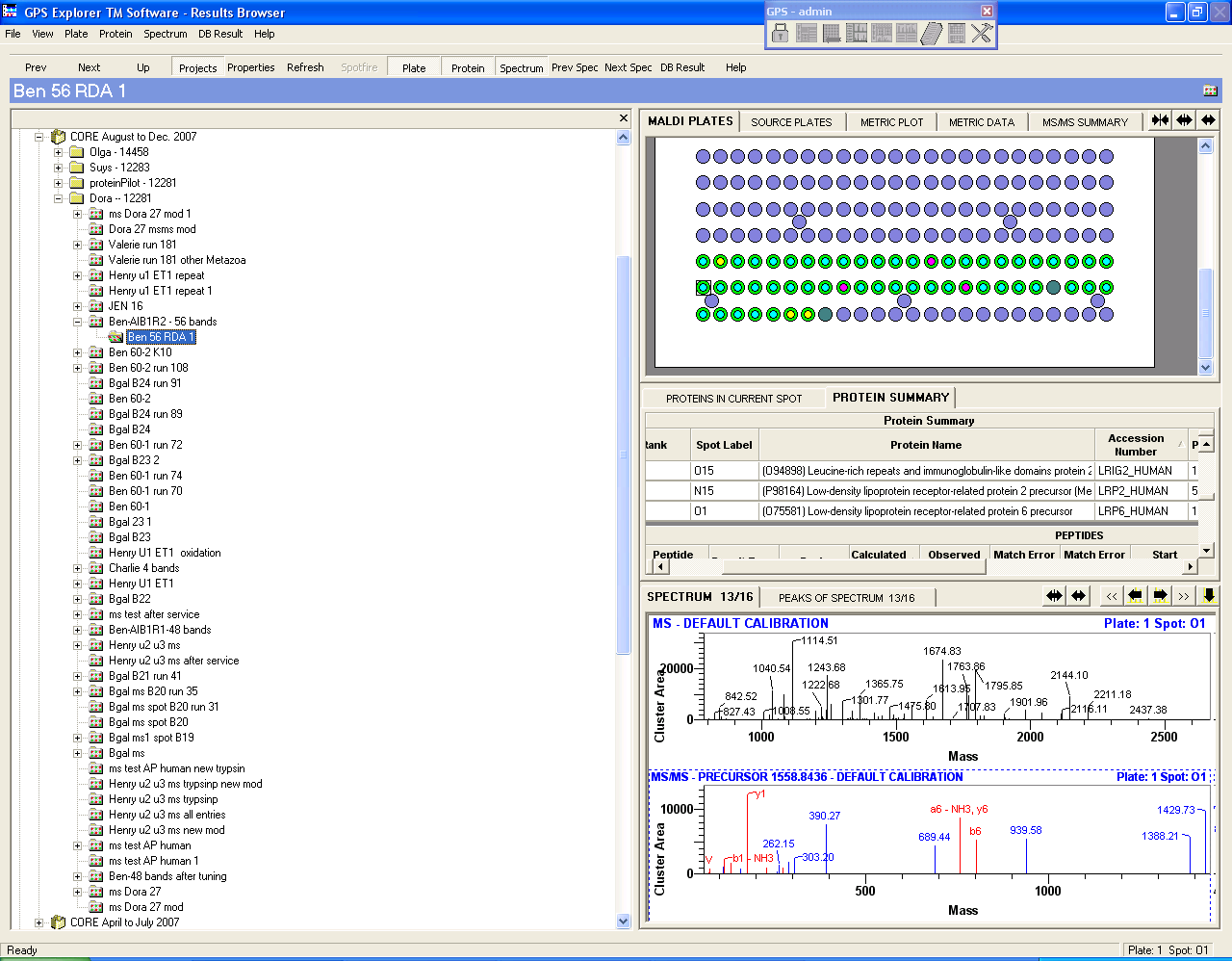 | | | | | | | | | | | | | | | | |
| S10 | Q04721 | | Notch2 | | 16 | | 48 | | GLLCEENIDDCAR | | | | 1507.7 | | | AIB1_A |
| 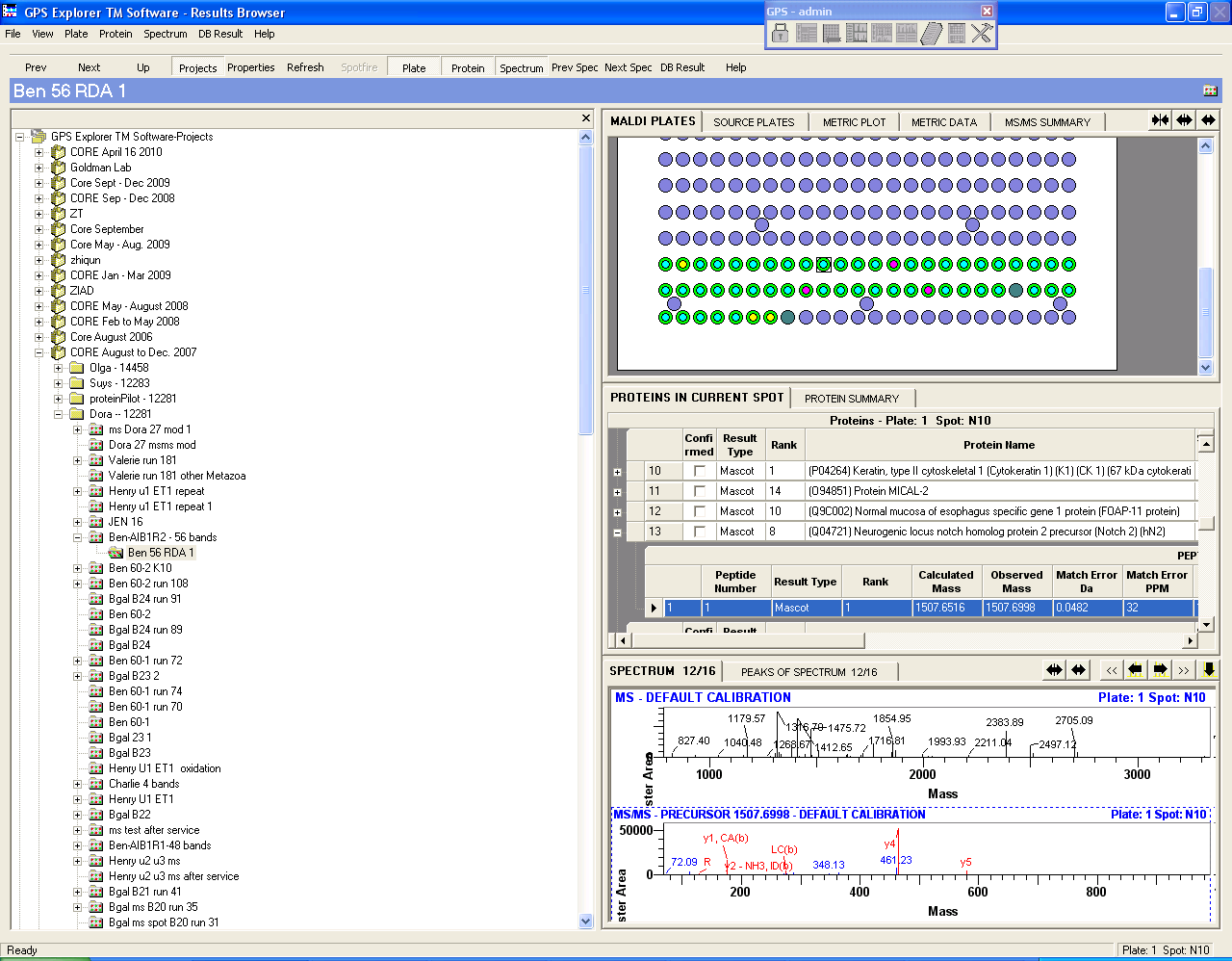 | | | | | | | | | | | | | | | | |
| S11 | Q9UM47 | | Notch3 | | 20 | | 88 | | RPPGKAGLGPQGPR | | | | 1387.7 | | | AIB1_C |
| 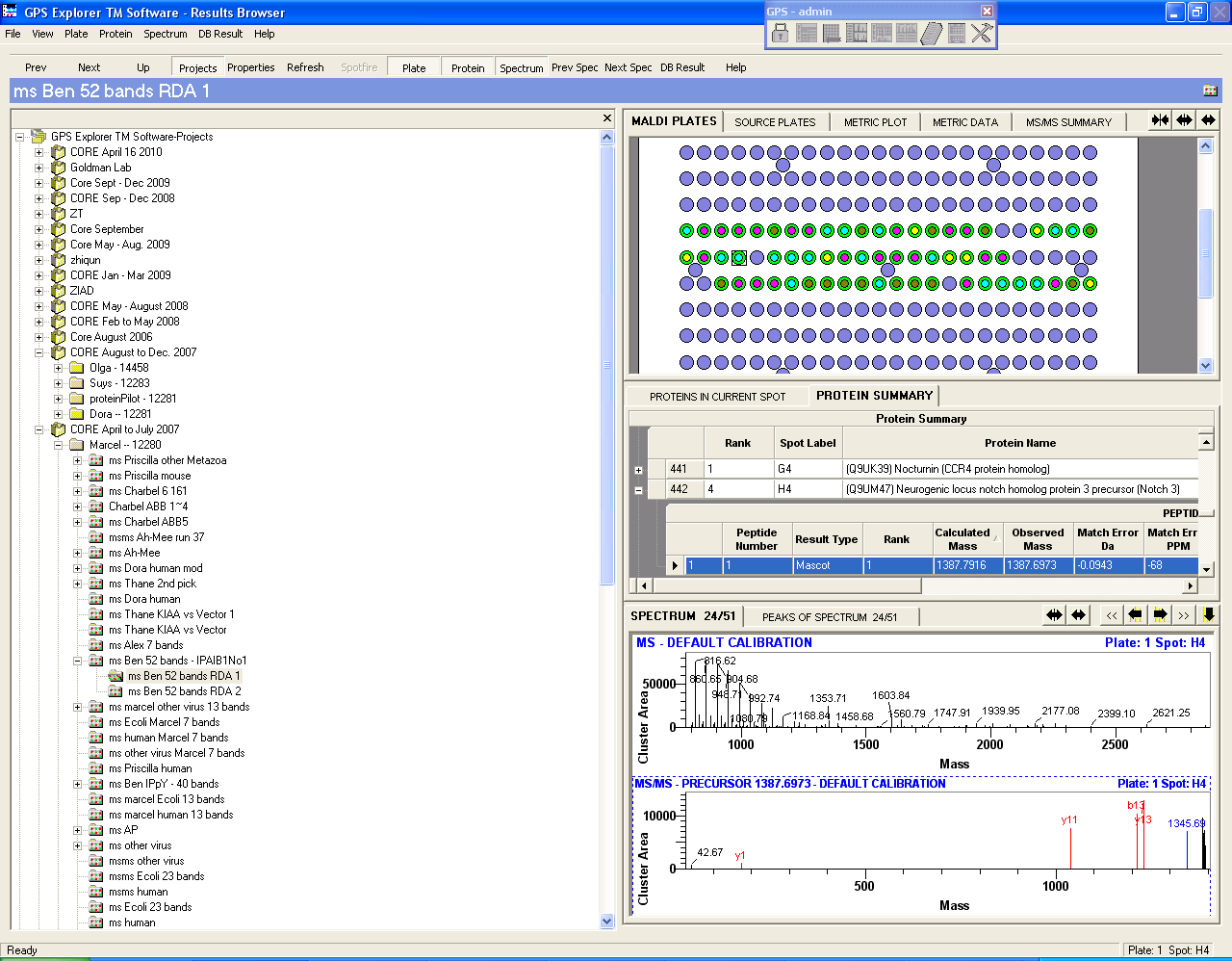 | | | | | | | | | | | | | | | | |
| S12 | Q9Y6R0 | | NUMBL | | 17 | | 62 | | HLPPAPCGAPGPPETCR | | | | 1813.94 | | | AIB1_C |
| 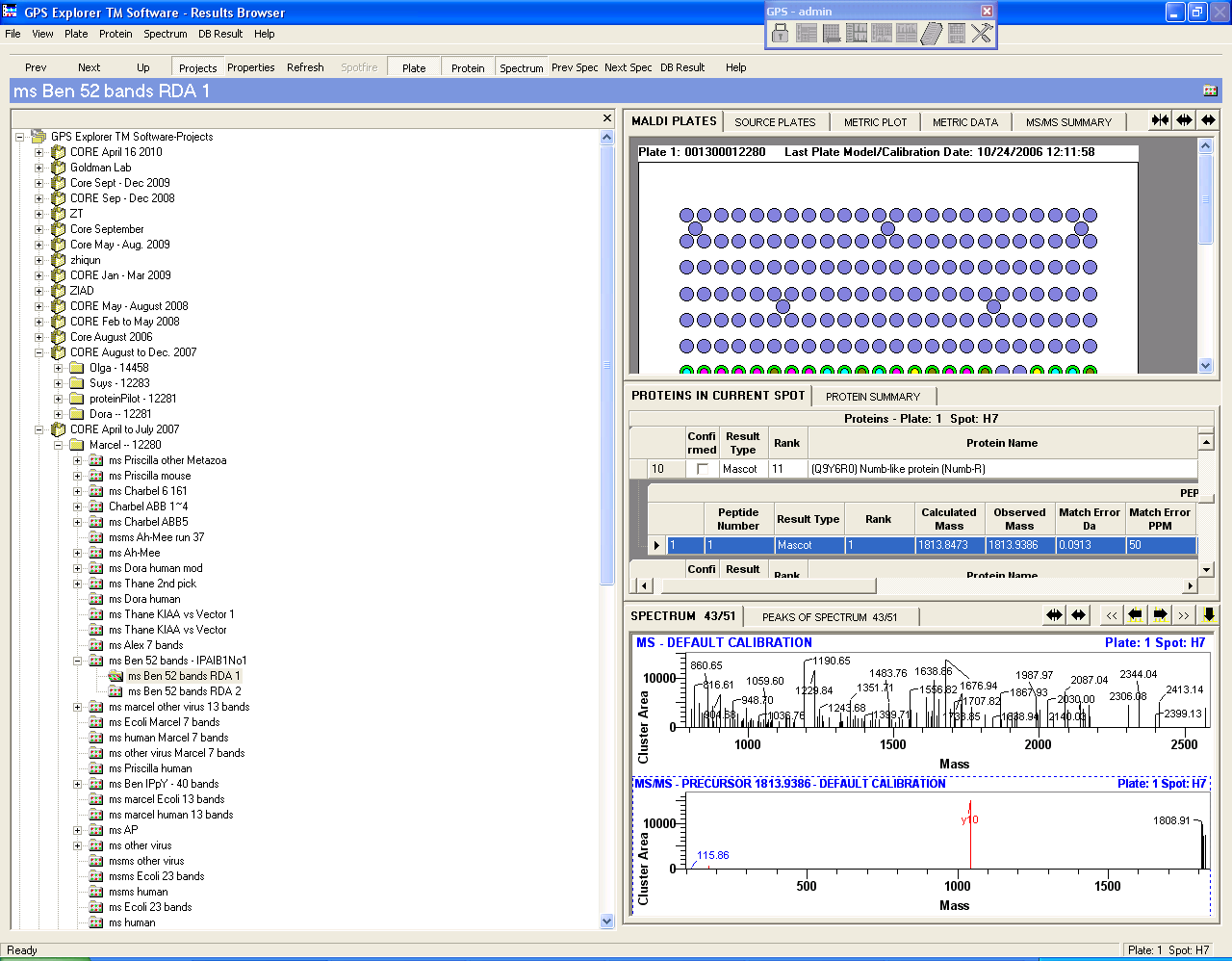 | | | | | | | | | | | | | | | | |
| S13 | P42338 | | PIK3CB | | 9 | | 20 | | QCCEDAYLILR | | | | 1383.65 | | | AIB1_B |
| 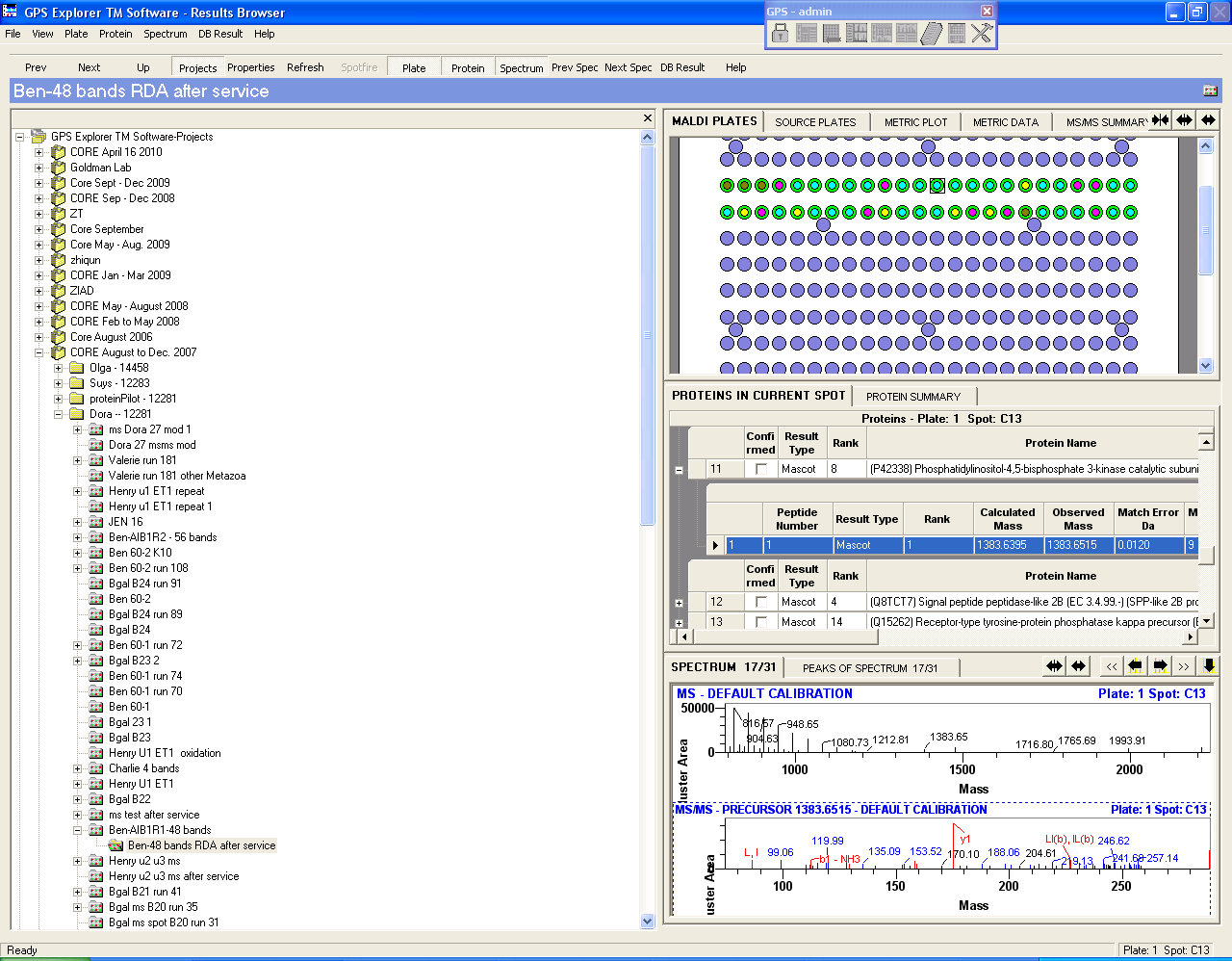 | | | | | | | | | | | | | | | | |
| S14 | O00329 | | PIK3CD | | 16 | | 58 | | LCDVQPFLPVLR | | | | 1399.71 | | | AIB1_C |
| 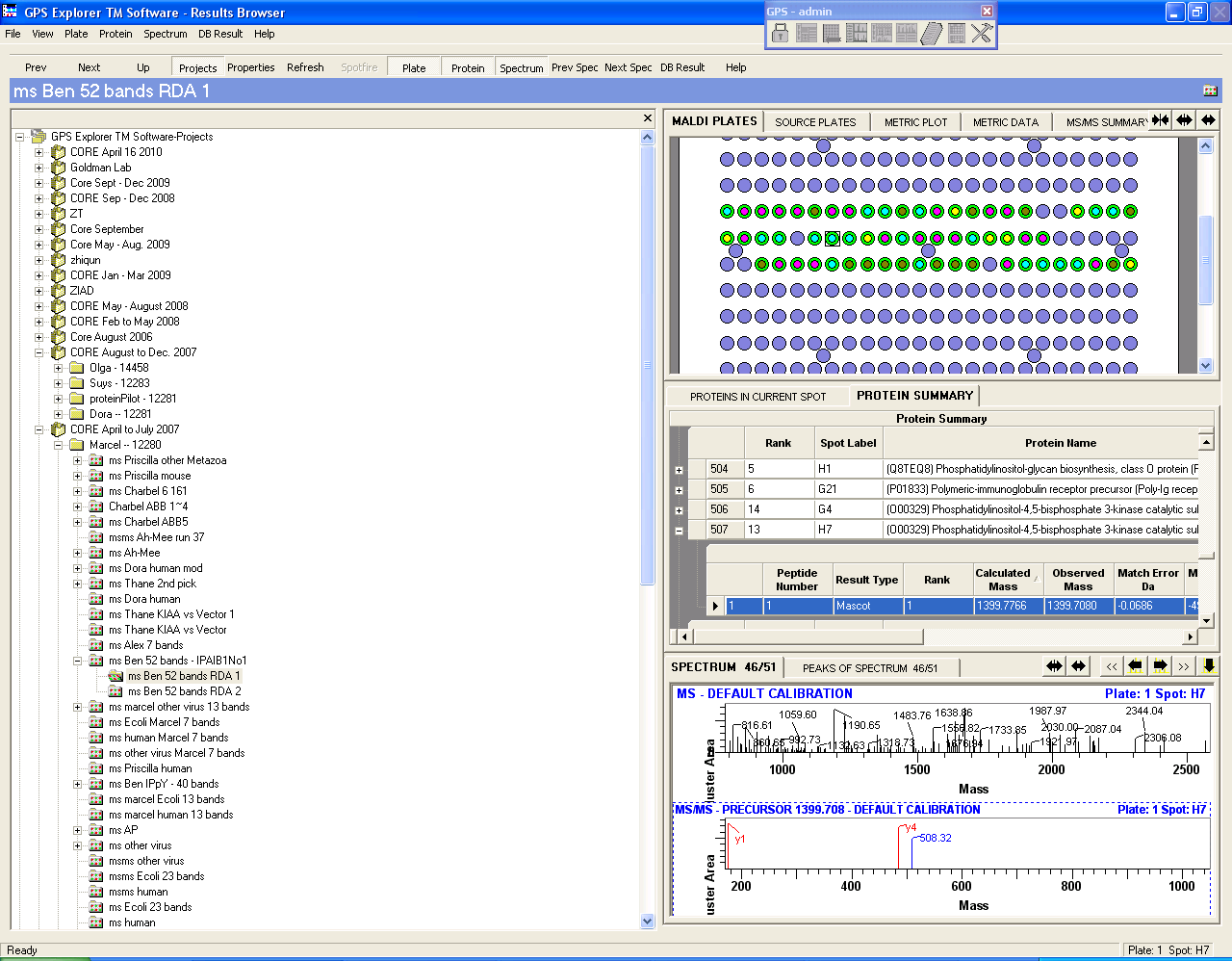 | | | | | | | | | | | | | | | | |
| S15 | Q92569 | | PIK3R3 | | 15 | | 41 | | MQGDYTLTLR | | | | 1197.68 | | | AIB1_C |
| 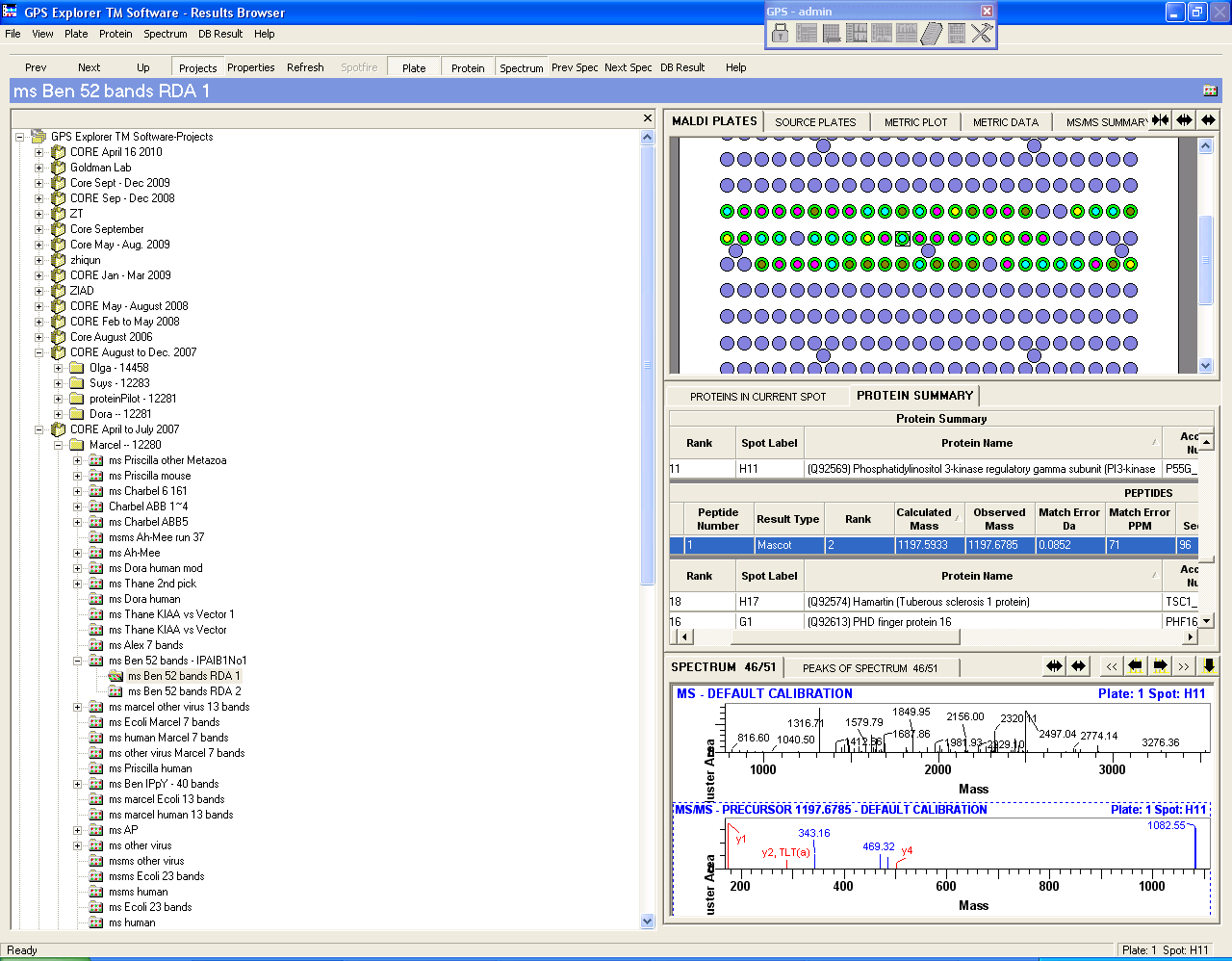 | | | | | | | | | | | | | | | | |
| S16 | Q9NQ66 | | PLCB1 | | 16 | | 40 | | RVETALEACSLPSSR | | | | 1618.81 | | | AIB1_B |
| 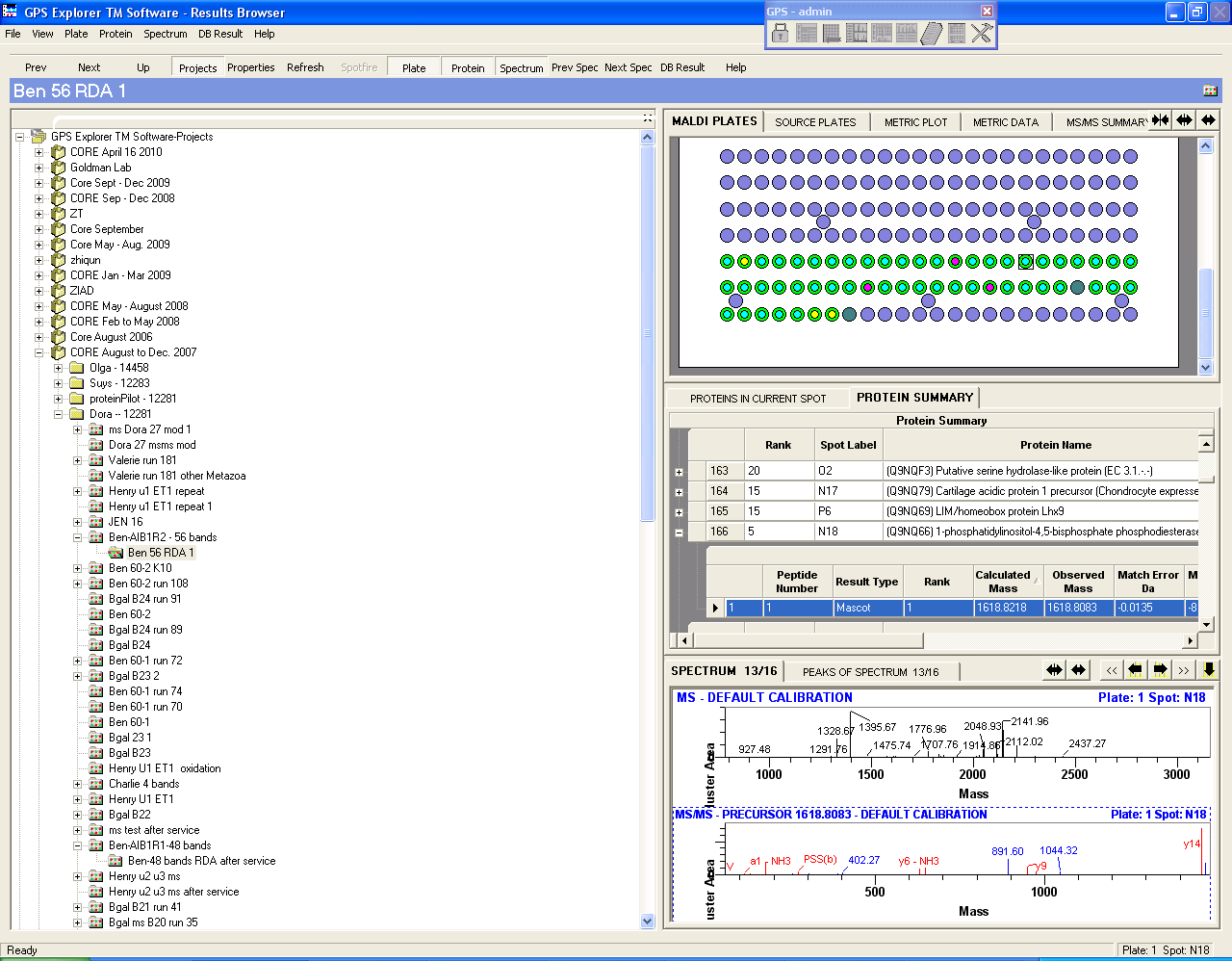 | | | | | | | | | | | | | | | | |
| S17 | Q9NQ66 | | PLCB1 | | 18 | | 63 | | RVETALEACSLPSSR | | | | 1618.83 | | | AIB1_C |
| 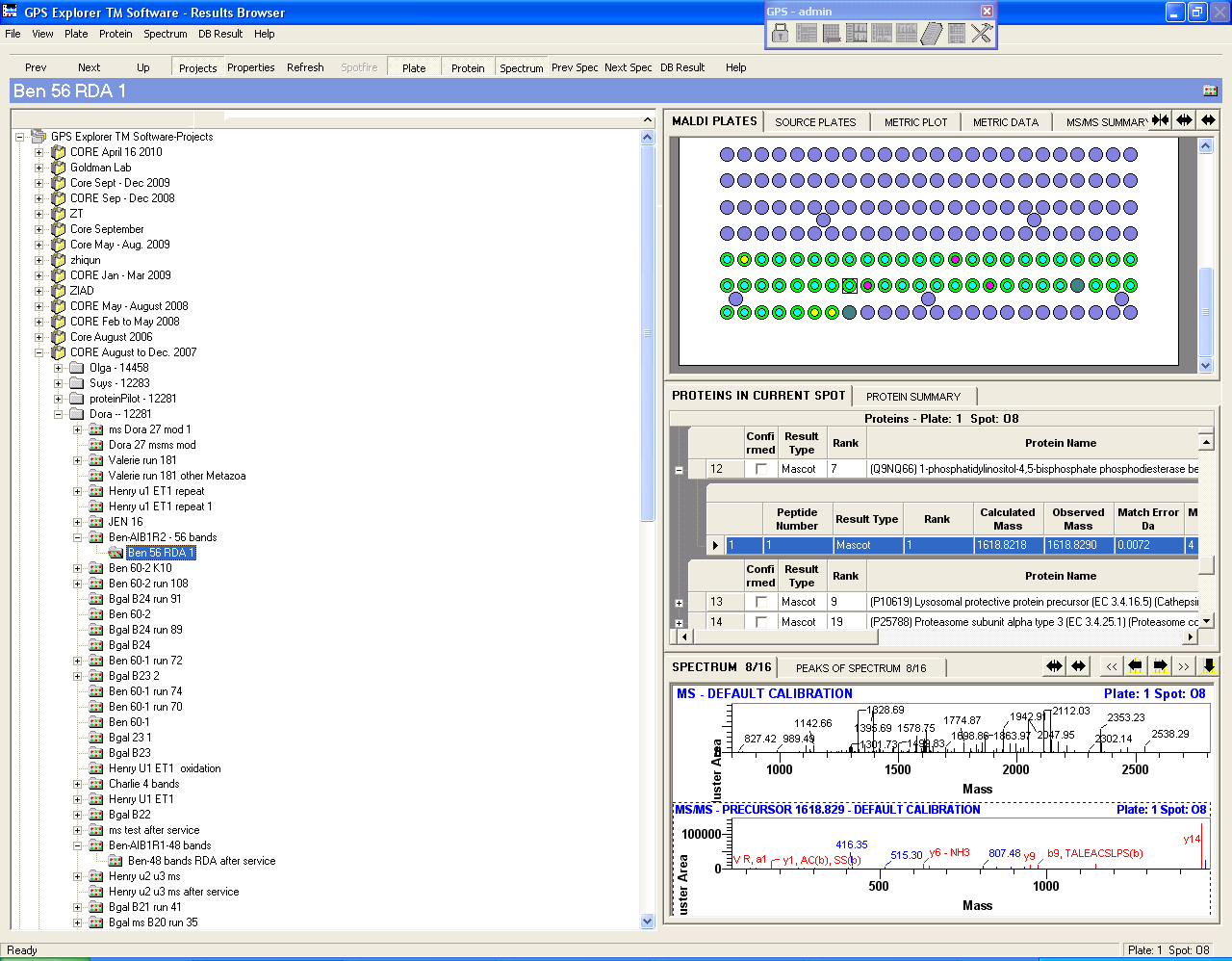 | | | | | | | | | | | | | | | | |
| S18 | Q9NQ66 | | PLCB1 | | 18 | | 59 | | RVETALEACSLPSSR | | | | 1618.83 | | | AIB1_D |
| 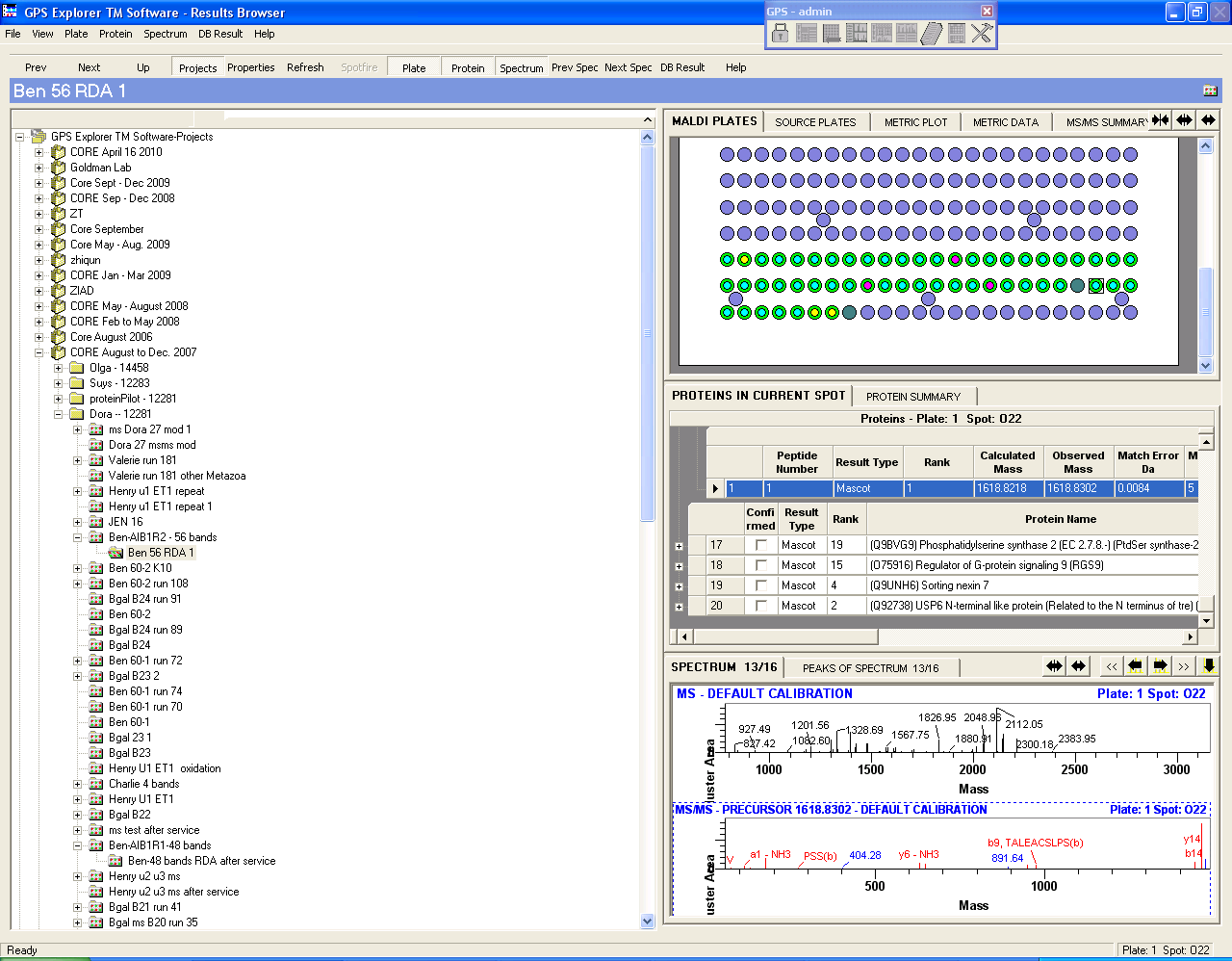 | | | | | | | | | | | | | | | | |
| S19 | O15297 | | PP2C/WIP1 | | 8 | | 39 | | YLMGEHGQSCAKMLVNR | | | | 1993.96 | | | AIB1_A |
| 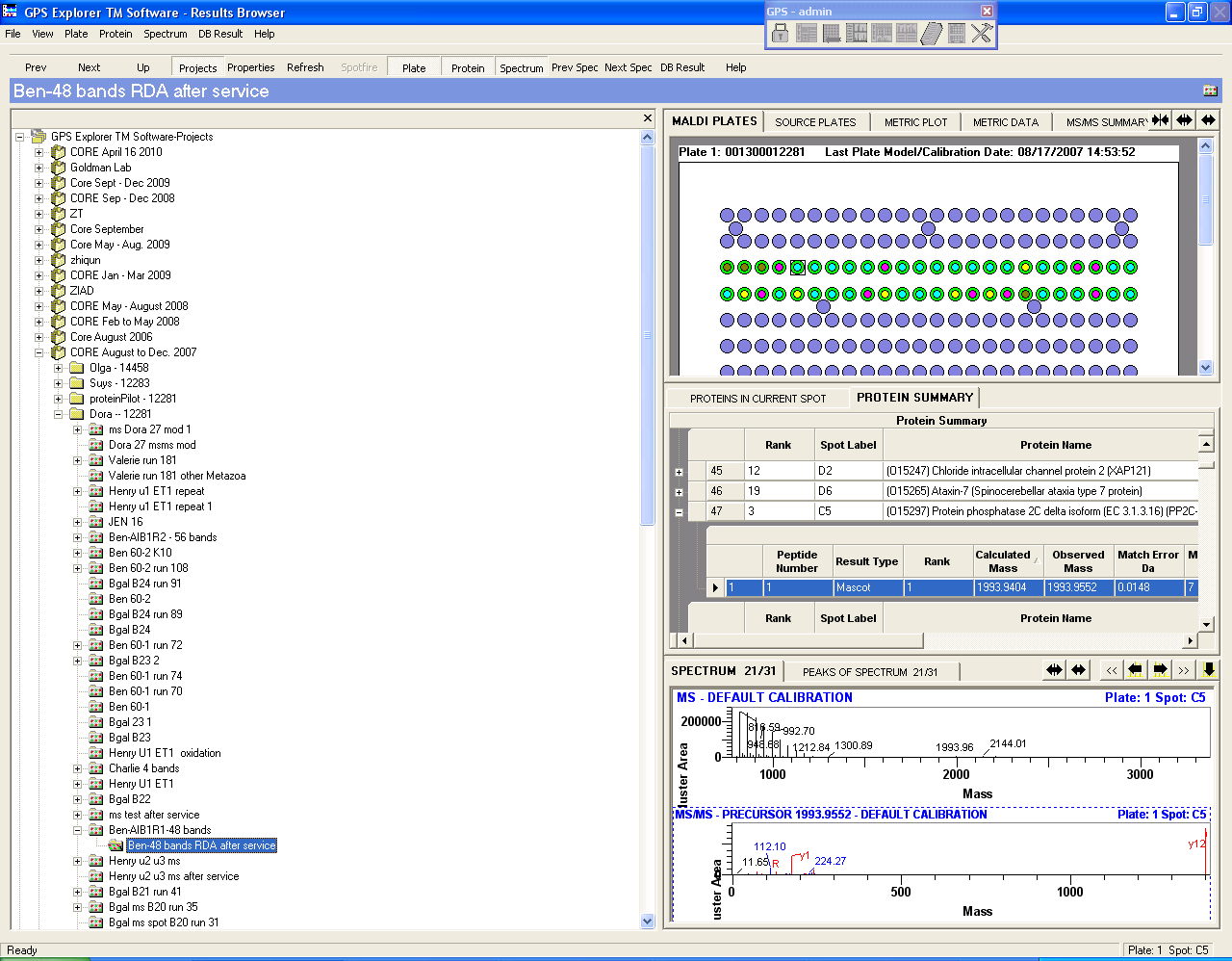 | | | | | | | | | | | | | | | | |
| S20 | P16298 | | PPP3CB | | 15 | | 80 | | AAVLKYENNVMNIR | | | | 1650.85 | | | AIB1_C |
| 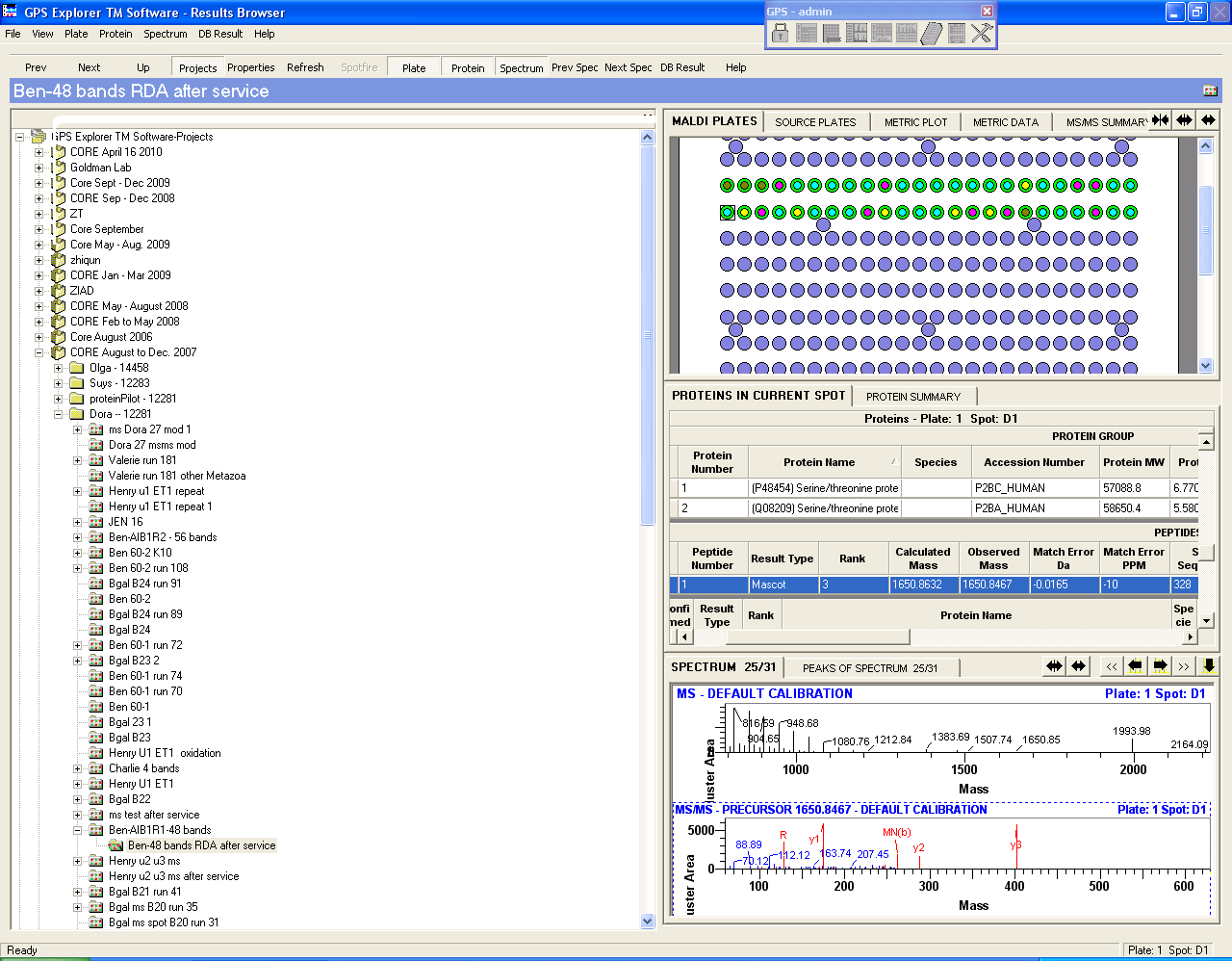 | | | | | | | | | | | | | | | | |
| S21 | P61224 | | RAP1B | | 15 | | 54 | | SKINVNEIFYDLVR | | | | 1709.89 | | | AIB1_C |
| 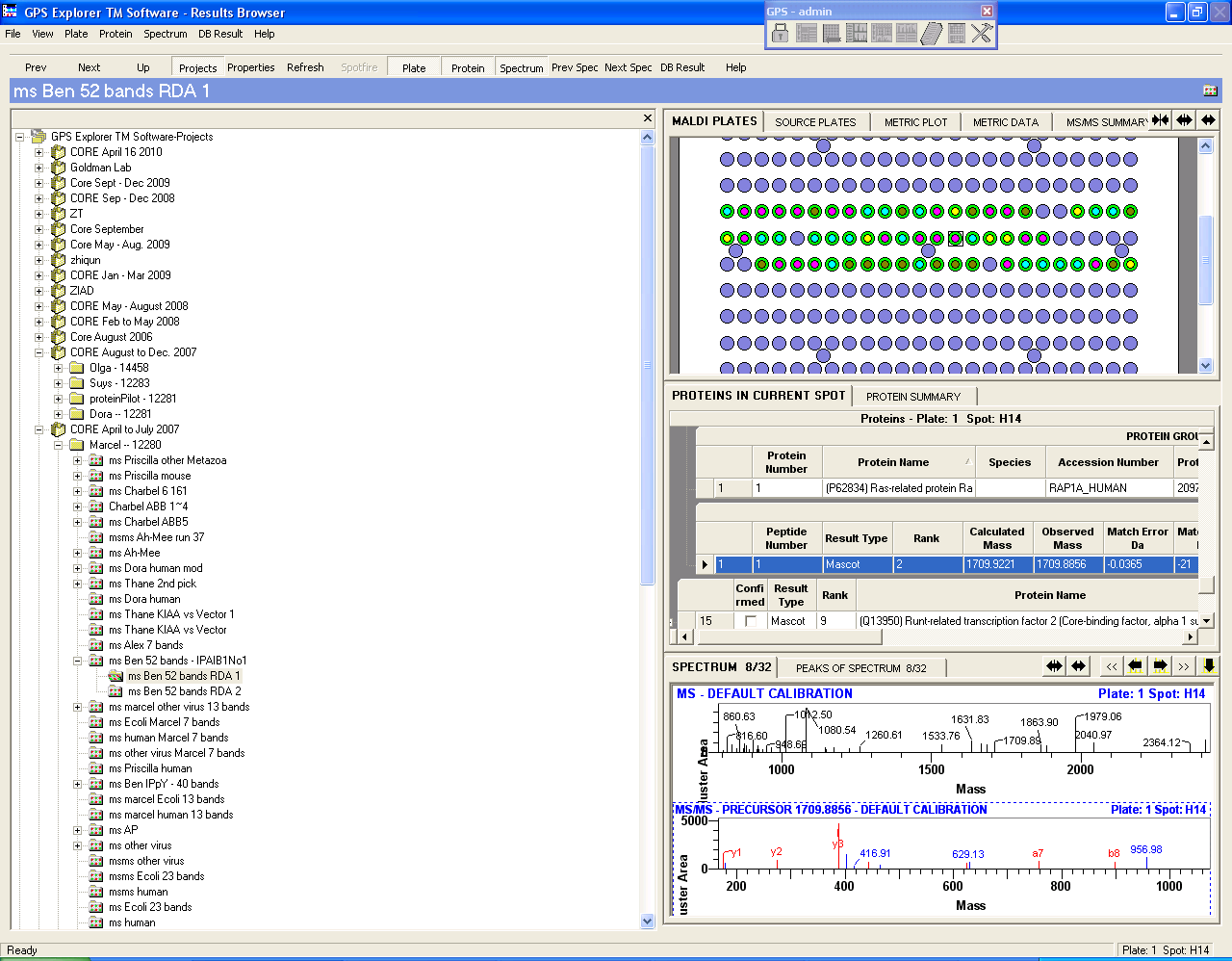 | | | | | | | | | | | | | | | | |
| S22 | P47736 | | RAP1GAP | | 21 | | 89 | | SSAIGIENIQEVQEK | | | | 1644.77 | | | AIB1_D |
| 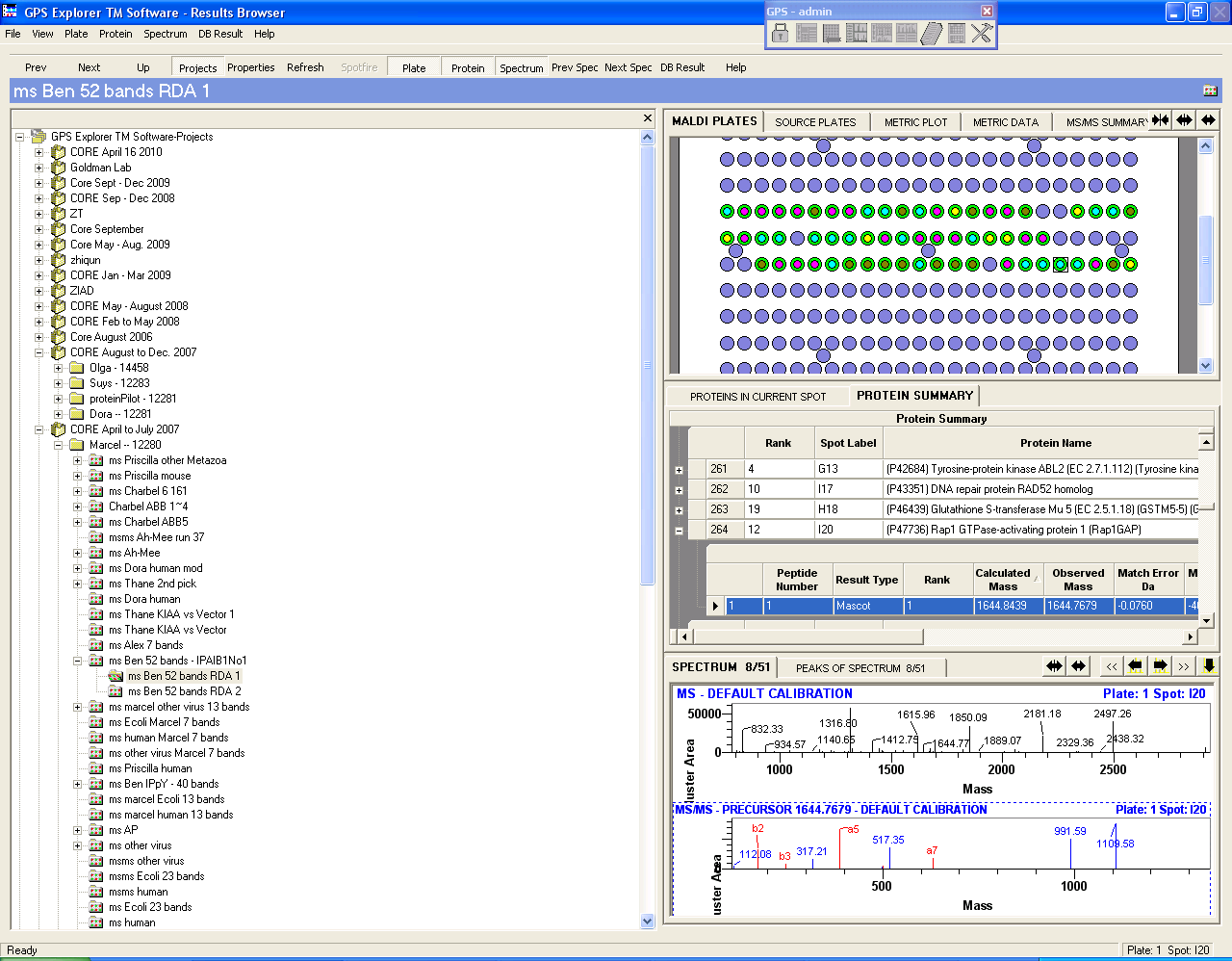 | | | | | | | | | | | | | | | | |
| S23 | Q15418 | | RPS6KA1 (RSK1) | | 16 | | 39 | | LGSGPDGAEEIKR | | | | 1328.69 | | | AIB1_C |
| 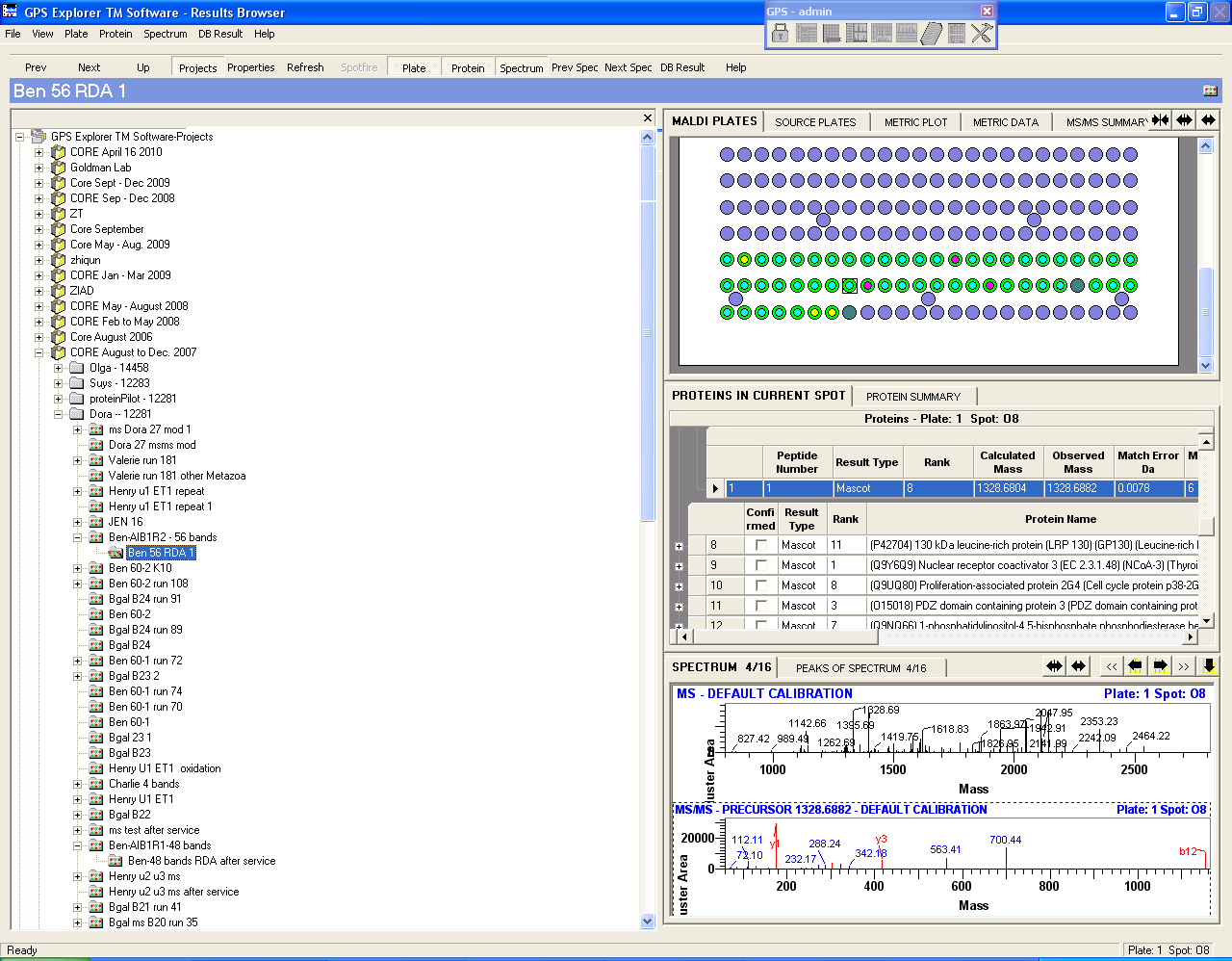 | | | | | | | | | | | | | | | | |
| S24 | Q15349 | | RPS6KA2 (RSK2) | | 12 | | 41 | | AGNGLLMTPCYTANFVAPEVLKR | | | | 2538.21 | | | AIB1_D |
| 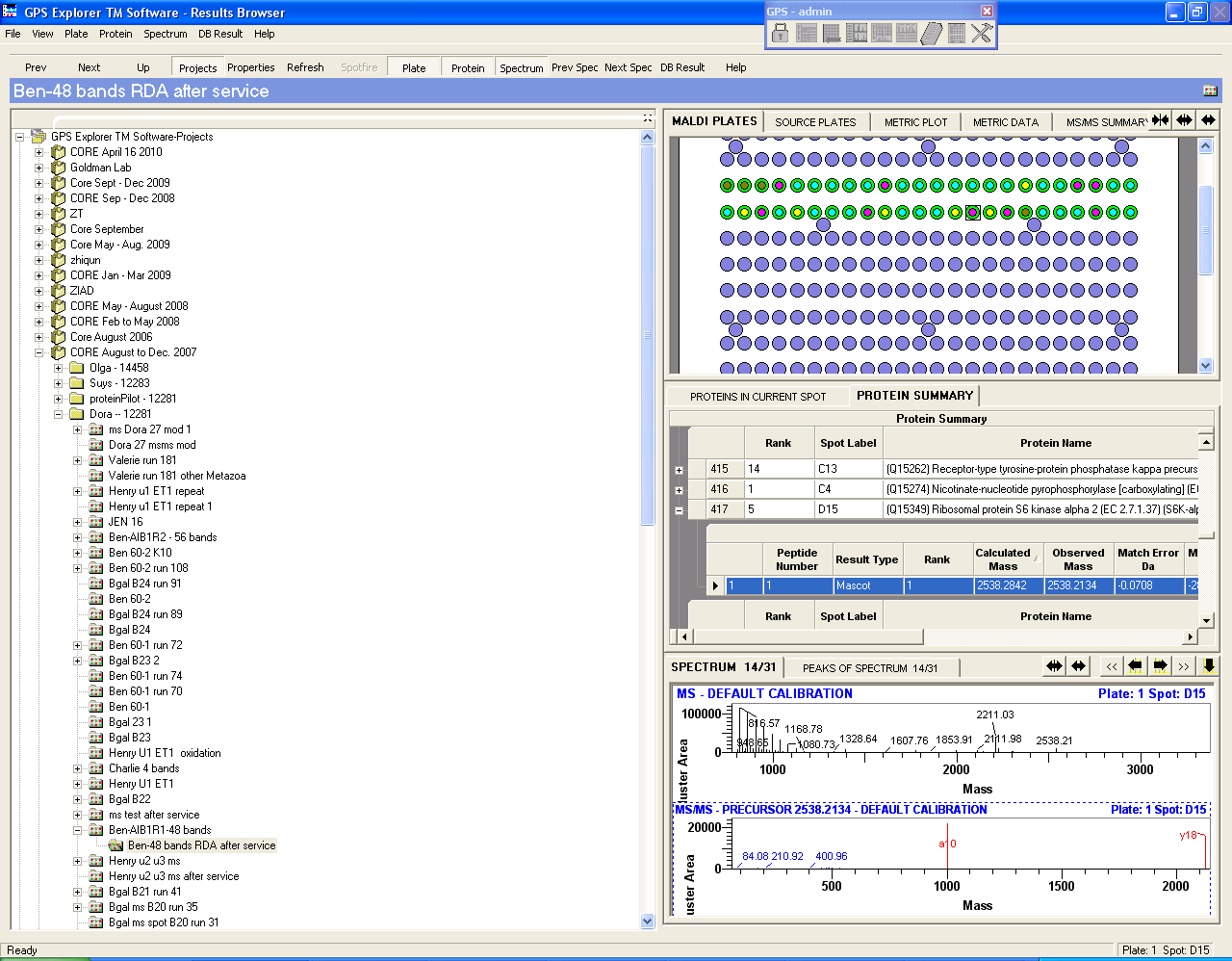 | | | | | | | | | | | | | | | | |
| S25 | P10301 | | RRAS | | 14 | | 51 | | SSGAASGTGRGRPR | | | | 1316.77 | | | AIB1_B |
| 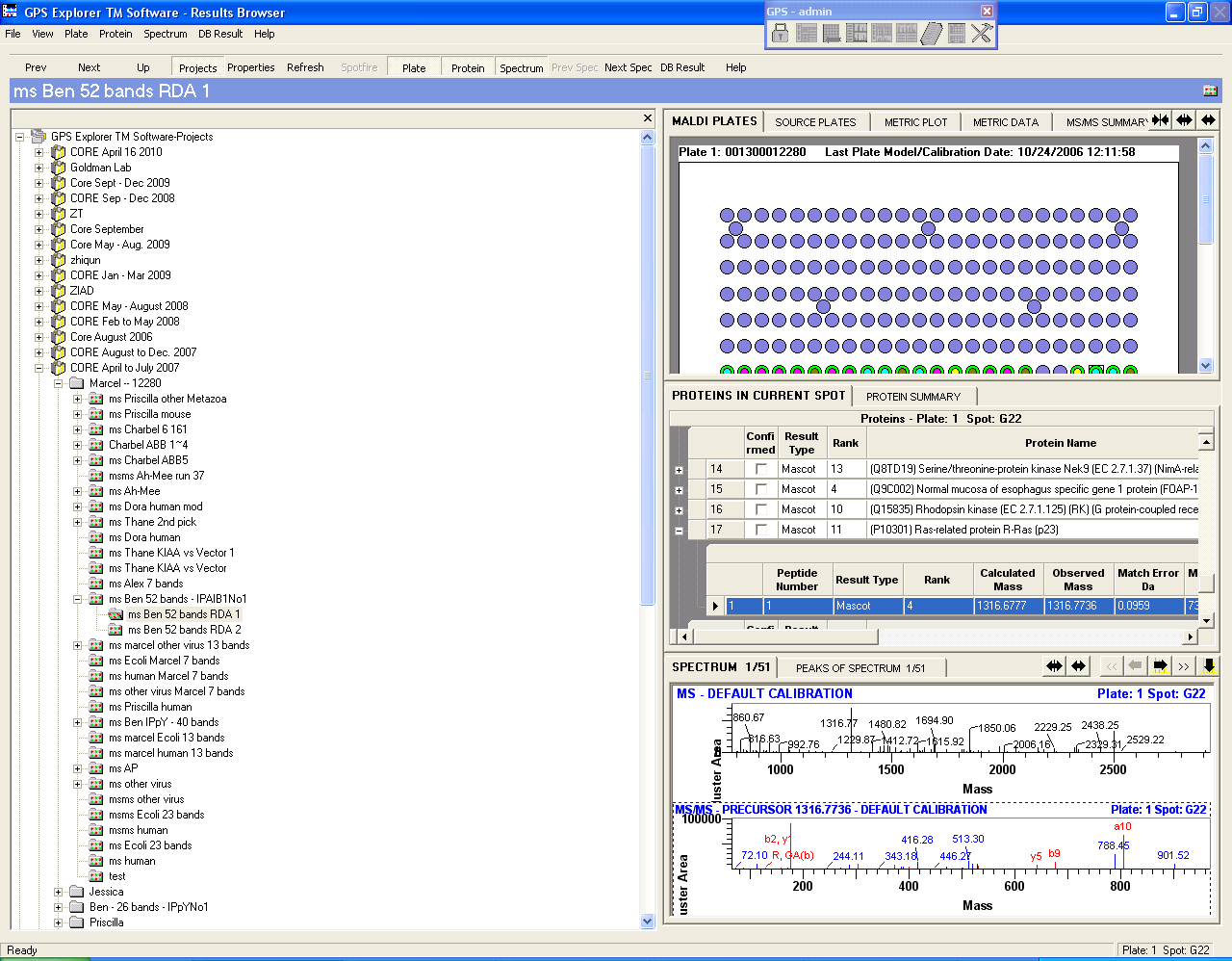 | | | | | | | | | | | | | | | | |
| S26 | P29597 | | TYK2 | | 15 | | 44 | | QDNKCLELSLPSR | | | | 1559.8 | | | AIB1_A |
| 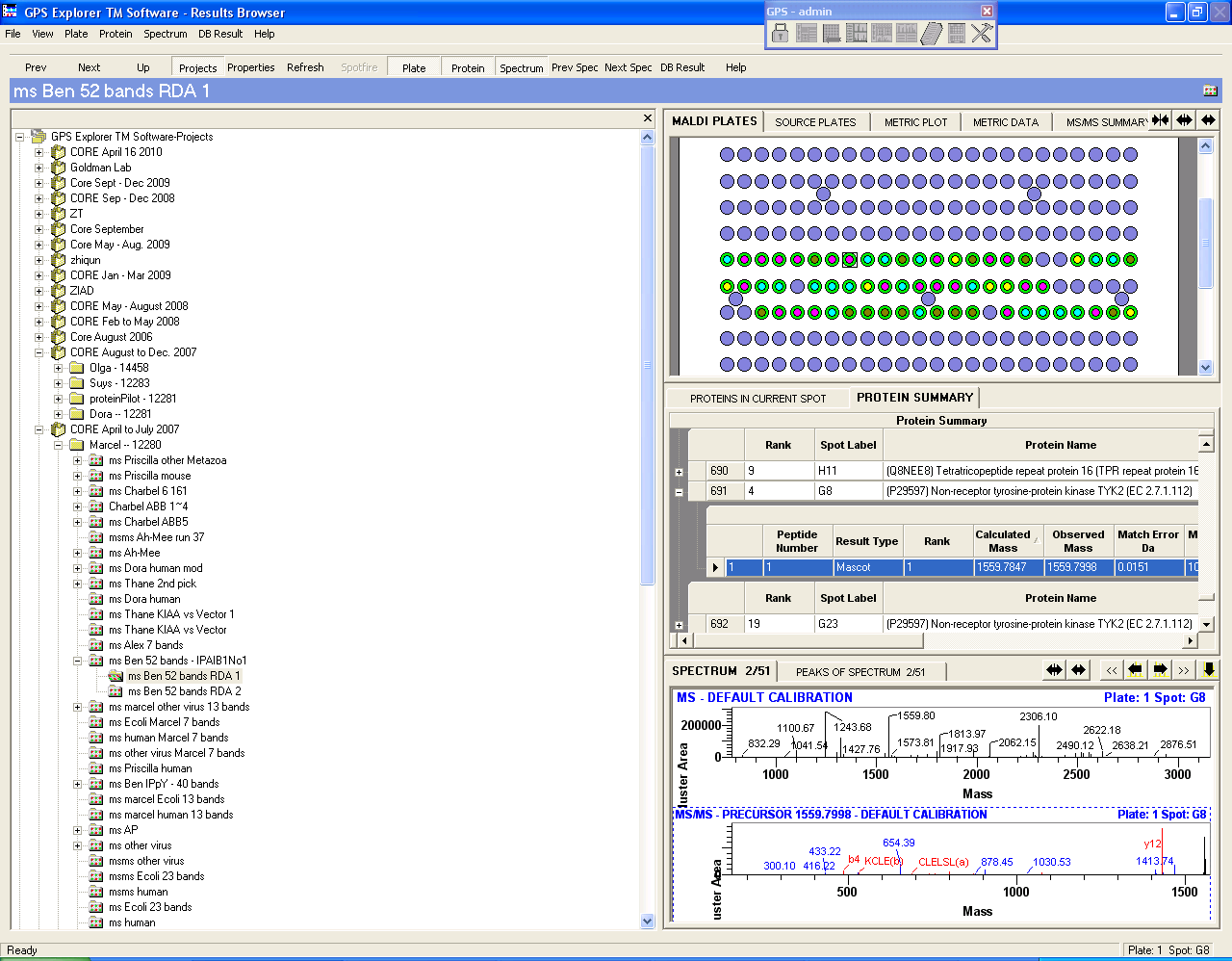 | | | | | | | | | | | | | | | | |
| S27 | P29597 | | TYK2 | | 14 | | 49 | | QDNKCLELSLPSR | | | | 1559.84 | | | AIB1_B |
| 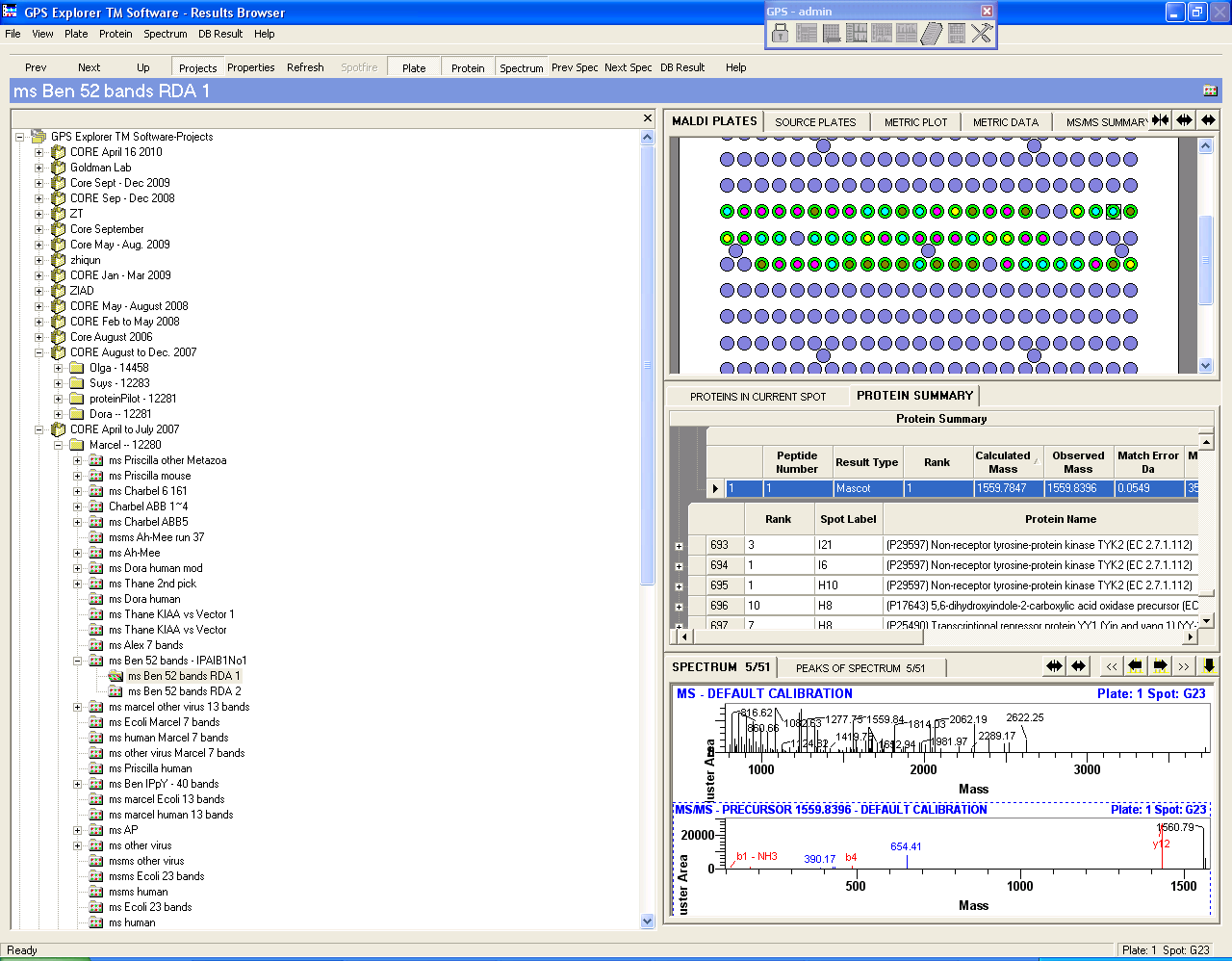 | | | | | | | | | | | | | | | | |
| S28 | P29597 | | TYK2 | | 17 | | 83 | | QDNKCLELSLPSR | | | | 1559.81 | | | AIB1_C |
| 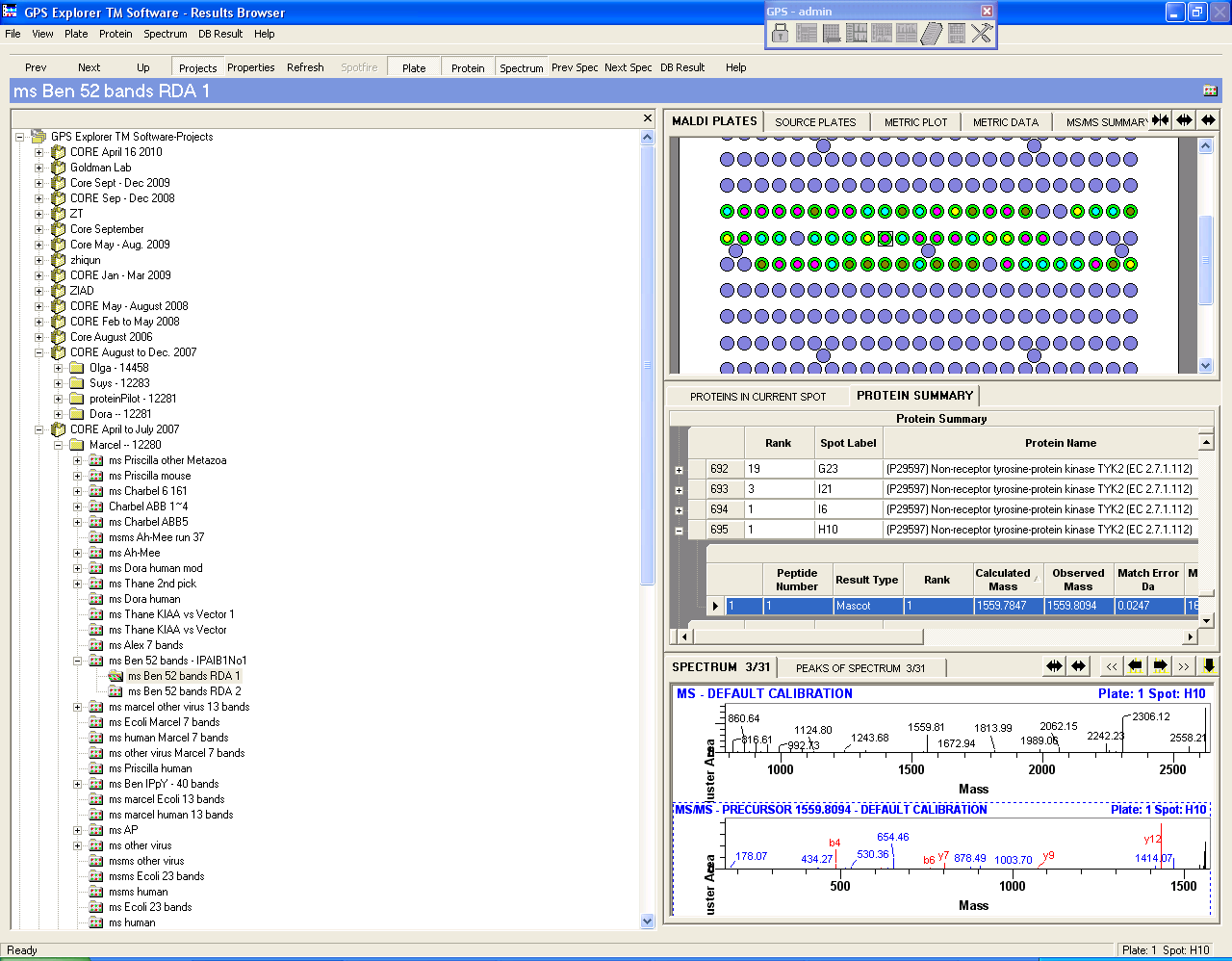 | | | | | | | | | | | | | | | | |
| S29 | P29597 | | TYK2 | | 22 | | 88 | | QDNKCLELSLPSR | | | | 1559.8 | | | AIB1_D |
| 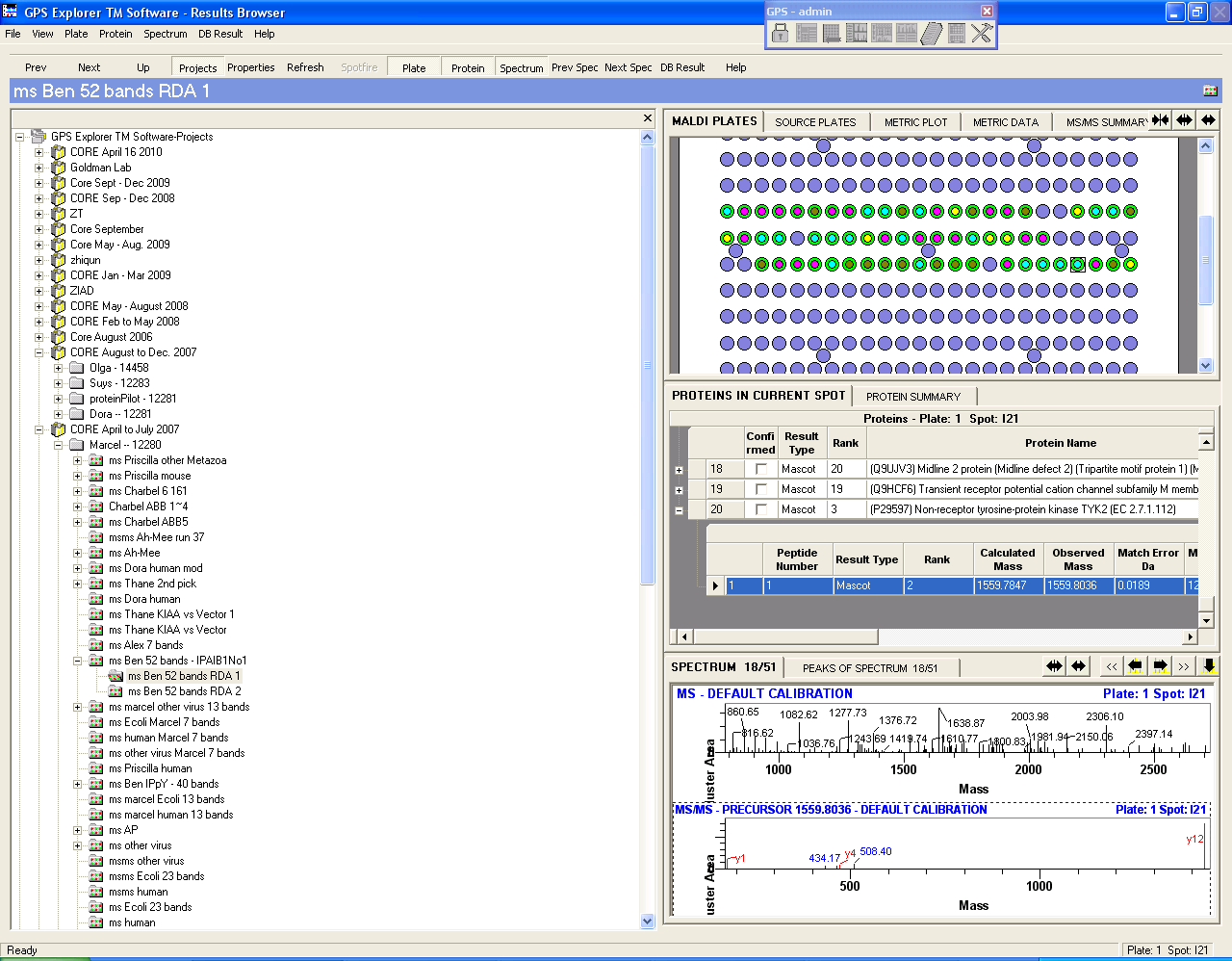 | | | | | | | | | | | | | | | | |
| S30 | O00755 | | WNT7A | | 17 | | 77 | | QNARTLMNLHNNEAGR | | | | 1838.9 | | | AIB1_C |
| 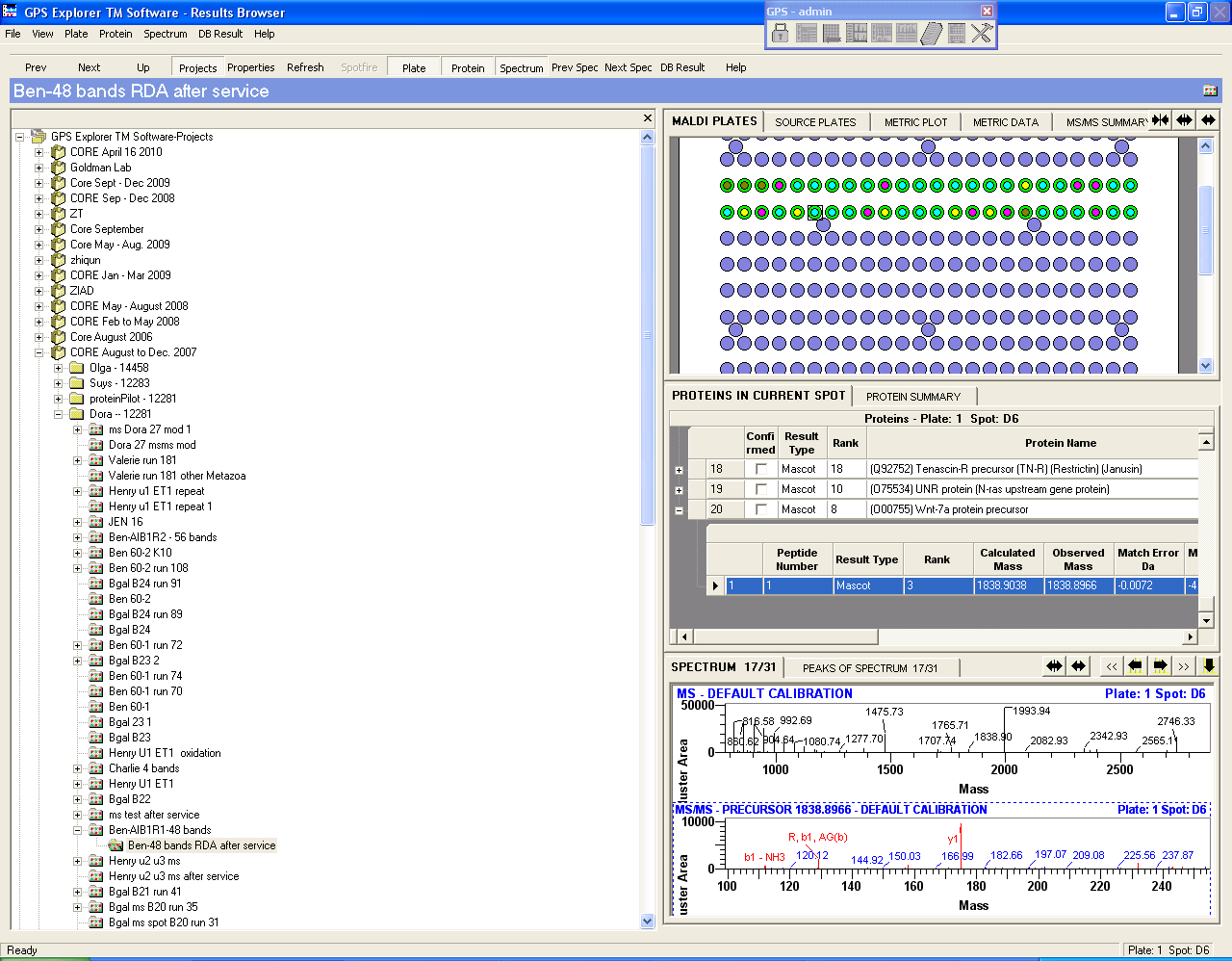 | | | | | | | | | | | | | | | | |
| S31 | Q9Y297 | | BTRCP | | 14 | | 22 | | LVVSGSSDNTIR | | | | 1247.61 | | | pY_B |
| 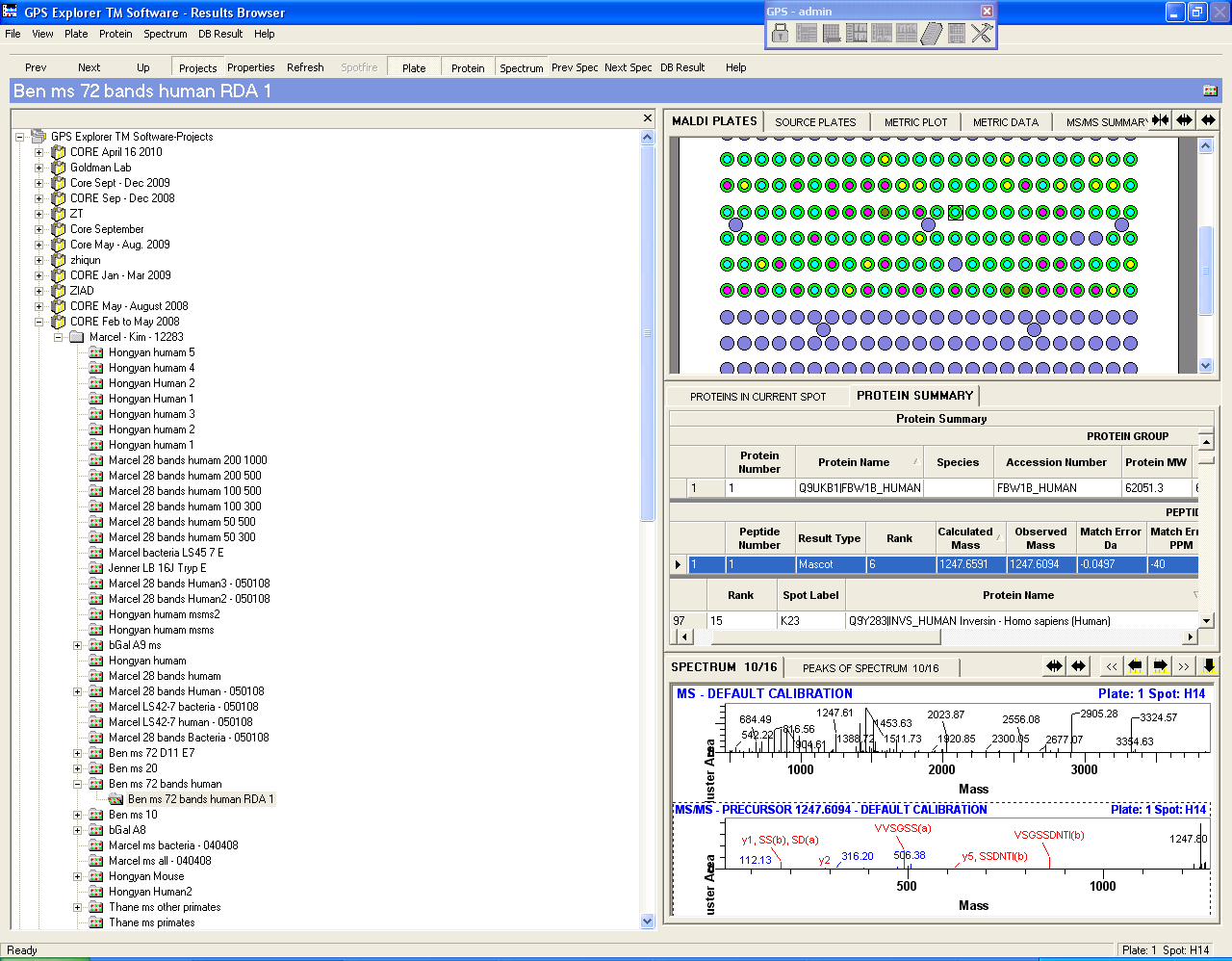 | | | | | | | | | | | | | | | | |
| S32 | Q9BT81 | | SOX7 | | 15 | | 37 | | EDRGEYSPGTALPSLR | | | | 1747.88 | | | pY_B |
| 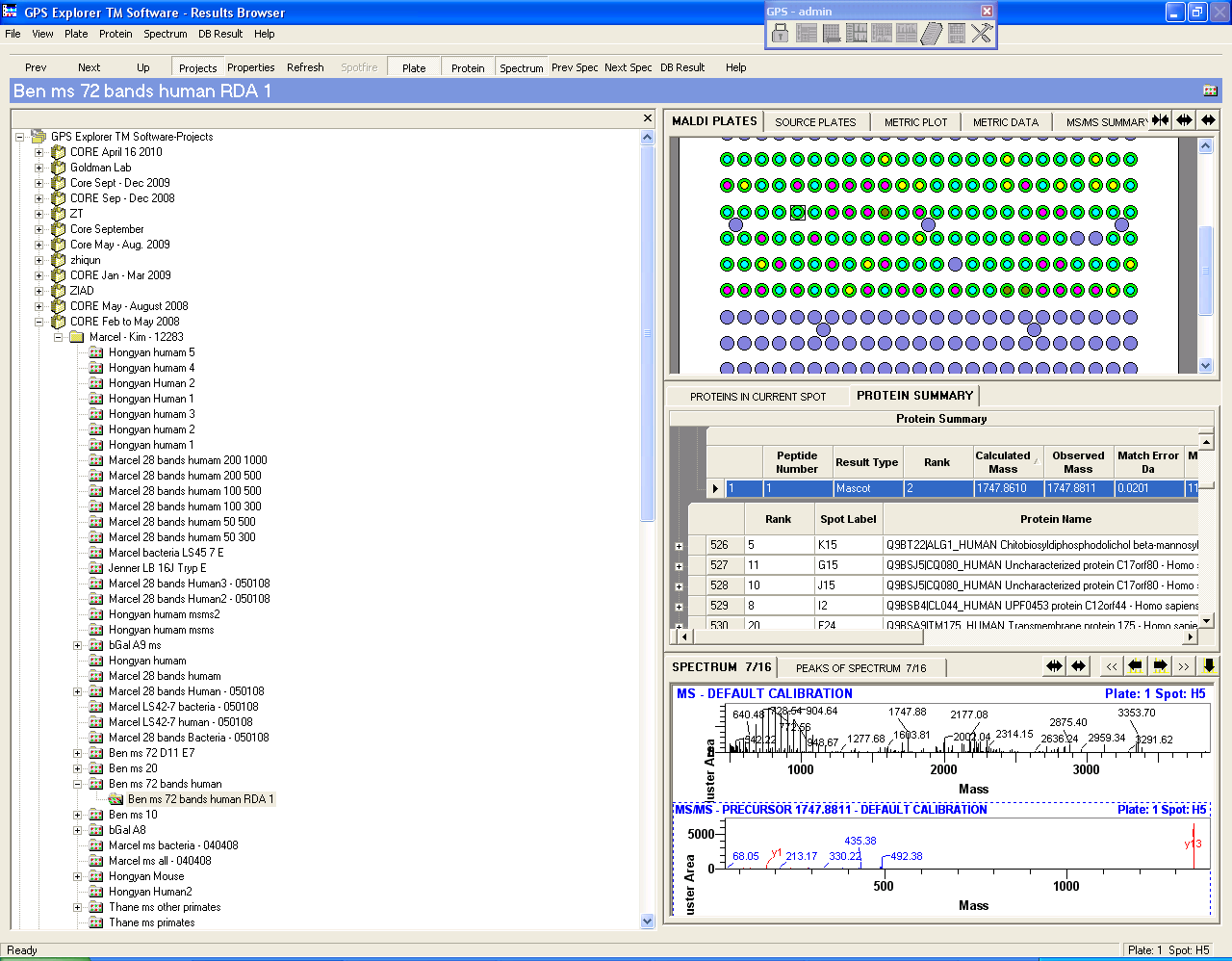 | | | | | | | | | | | | | | | | |
| S33 | P36897 | | TGFBR1 | | 15 | | 51 | | ADIYAMGLVFWEIARR | | | | 1926.86 | | | pY_D |
| 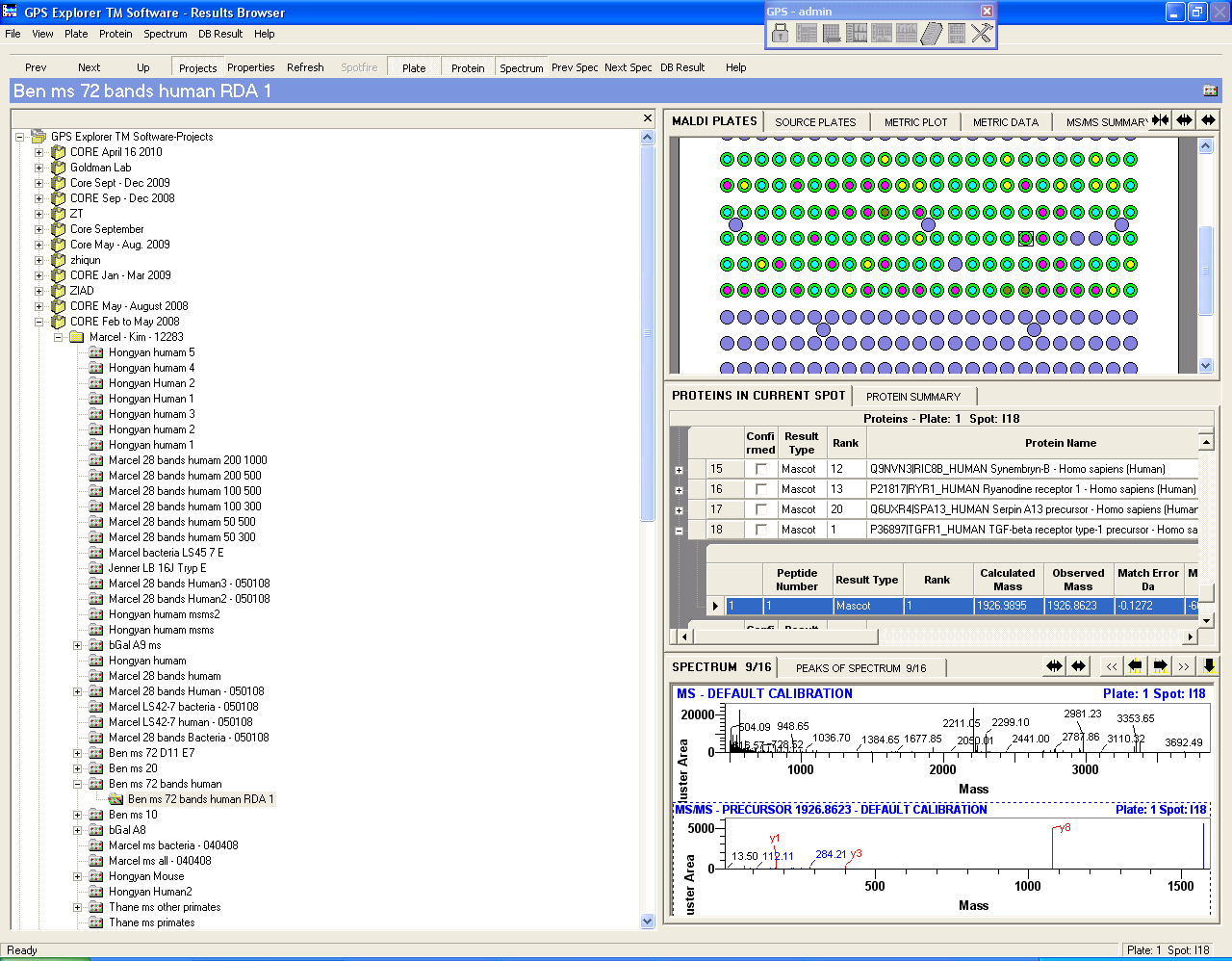 | | | | | | | | | | | | | | | | |
