# Supplementary material for: Proteomic Analysis of Pathways Involved in Estrogen-Induced Growth and Apoptosis of Breast Cancer Cells
Source: PLoS One. 2011 Jun 27;6(6):e20410. doi: 10.1371/journal.pone.0020410 (PMC3124472; doi:10.1371/journal.pone.0020410)
Supplement: Table S5 — pY-complexed proteins identified by MALDI-TOF-TOF. Proteins were identified based on single MS (MS) or tandem MS (MS/MS) using the search engine Mascot 2.0 from the Swiss-Prot database. Note that the same proteins could be identified under different experimental (“Exp.”) conditions: A, MCF-7 cells, no E2; B, MCF-7:5C cell, no E2; C, MCF-7 cell, +E2; D, MCF-7:5C cell, +E2. Proteins that were identified more than once from experimental repeats under the same conditions are labeled with * in the “Exp” column. For proteins identified from single peptide MS/MS, spectral data (Spec.) are referenced using the labels (Y1–Y38) to correspond to those shown in Table S7. All spectra for single peptides shown here were manually inspected, including those that show 90%≤ CI ≤95% but with good ion fragments. *The % coverage for single peptide MS/MS was only stated if the respective peptide covered ≥1% of the protein. The spectra and sequences are in Table S7. (DOC) [file pone.0020410.s013.doc]

| **UniProtKB AC** | **Gene Name** | **Score** | **CI%** | **# peptides** | **Coverage (%)** | **MS** | **Exp.** | **Spec.** |
| --- | --- | --- | --- | --- | --- | --- | --- | --- |
| O00159 | MYO1C | 28 | 96 | 2 | 3 | MS/MS | A |  |
| O15020 | SPTBN2 | 31 | 98 | 2 | 1 | MS/MS | B |  |
| O15020 | SPTBN2 | 29 | 97 | 2 | 1 | MS/MS | B |  |
| O43707 | ACTN4 | 88 | 100 | 17 | 30 | MS | B |  |
| O43707 | ACTN4 | 117 | 100 | 20 | 34 | MS | D |  |
| O43707 | ACTN4 | 146 | 100 | 5 | 11 | MS/MS | B |  |
| O43707 | ACTN4 | 238 | 100 | 5 | 10 | MS/MS | D |  |
| O43795 | MYO1B | 37 | 100 | 3 | 5 | MS/MS | A |  |
| O43795 | MYO1B | 27 | 95 | 2 | 3 | MS/MS | A |  |
| P05388 | RPLP0 | 36 | 99 | 1 | 9 | MS/MS | A | Y1 |
| P06493 | CDC2 | 39 | 100 | 2 | 7 | MS/MS | D |  |
| P08107 | HSPA1A | 118 | 100 | 2 | 8 | MS/MS | A |  |
| P08107 | HSPA1A | 51 | 100 | 1 | NA | MS/MS | A | Y2 |
| P08107 | HSPA1A | 74 | 100 | 13 | 28 | MS | B |  |
| P08107 | HSPA1A | 72 | 100 | 13 | 30 | MS | B |  |
| P08107 | HSPA1A | 75 | 100 | 3 | 5 | MS/MS | B |  |
| P08107 | HSPA1A | 101 | 100 | 2 | 6 | MS/MS | B |  |
| P08107 | HSPA1A | 195 | 100 | 24 | 57 | MS | C |  |
| P08107 | HSPA1A | 134 | 100 | 2 | 6 | MS/MS | C |  |
| P08107 | HSPA1A | 56 | 96 | 11 | 28 | MS | D |  |
| P08107 | HSPA1A | 118 | 100 | 18 | 40 | MS | D |  |
| P08107 | HSPA1A | 90 | 100 | 2 | 6 | MS/MS | D |  |
| P17066 | HSPA6 | 77 | 100 | 14 | 32 | MS | C |  |
| P17066 | HSPA6 | 59 | 98 | 12 | 26 | MS | D |  |
| P17066 | HSPA6 | 42 | 100 | 1 | 2 | MS/MS | D* | Y3 |
| P26045 | PTPN3 | 32 | 98 | 1 | 1 | MS/MS | D | Y4 |
| P27824 | CANX | 47 | 100 | 1 | 2 | MS/MS | D | Y5 |
| P29728 | OAS2 | 28 | 97 | 1 | NA | MS/MS | C | Y6 |
| P30050 | RPL12 | 46 | 100 | 1 | NA | MS/MS | B | Y7 |
| P30050 | RPL12 | 35 | 99 | 1 | NA | MS/MS | D | Y8 |
| P30050 | RPL12 | 28 | 96 | 1 | NA | MS/MS | C | Y9 |
| P35579 | MYH9 | 131 | 100 | 4 | 3 | MS/MS | C |  |
| P35579 | MYH9 | 48 | 100 | 2 | 2 | MS/MS | A |  |
| P38646 | HSPA9 | 53 | 100 | 2 | 5 | MS/MS | B |  |
| P38646 | HSPA9 | 120 | 100 | 3 | 5 | MS/MS | D |  |
| P38646 | HSPA9 | 27 | 95 | 1 | 2 | MS/MS | B | Y10 |
| P46783 | RPS10 | 39 | 100 | 1 | 9 | MS/MS | C | Y11 |
| P52740 | ZNF132 | 30 | 98 | 1 | NA | MS/MS | D | Y12 |
| P53618 | COPB1 | 45 | 100 | 1 | 2 | MS/MS | C | Y13 |
| P53621 | COPA | 37 | 100 | 1 | 1 | MS/MS | C | Y14 |
| P55060 | CSE1L | 61 | 100 | 1 | NA | MS/MS | D | Y15 |
| P57772 | EEFSEC | 54 | 95 | 11 | 25 | MS | C |  |
| P58107 | EPPK1 | 37 | 100 | 1 | NA | MS/MS | C | Y16 |
| P58107 | EPPK1 | 153 | 100 | 3 | 1 | MS/MS | B |  |
| P58107 | EPPK1 | 66 | 100 | 2 | 1 | MS/MS | D |  |
| P60709 | ACTB | 63 | 99 | 10 | 42 | MS | A |  |
| P61568 | SU | 62 | 99 | 6 | 48 | MS | A |  |
| P62158 | CALM1 | 83 | 100 | 1 | 10 | MS/MS | A | Y17 |
| P62269 | RPS18 | 48 | 100 | 2 | 7 | MS/MS | C |  |
| P62847 | RPS24 | 29 | 98 | 1 | 9 | MS/MS | B | Y18 |
| P62847 | RPS24 | 27 | 94 | 1 | 9 | MS/MS | C | Y19 |
| P62847 | RPS24 | 31 | 98 | 1 | 9 | MS/MS | A | Y20 |
| P62937 | PPIA | 41 | 100 | 1 | 10 | MS/MS | A* | Y21 |
| P62937 | PPIA | 32 | 98 | 1 | NA | MS/MS | C* | Y22 |
| P63261 | ACTG1 | 74 | 100 | 11 | 49 | MS | A |  |
| P63261 | ACTG1 | 133 | 100 | 4 | 22 | MS/MS | A |  |
| P63261 | ACTG1 | 83 | 100 | 2 | 12 | MS/MS | B |  |
| P63261 | ACTG1 | 145 | 100 | 3 | 15 | MS/MS | C |  |
| Q05397 | PTK2 | 40 | 100 | 1 | 1 | MS/MS | D* | Y23 |
| Q13439 | GOLGA4 | 60 | 99 | 27 | 13 | MS | B |  |
| Q13813 | SPTAN1 | 96 | 100 | 27 | 19 | MS | B |  |
| Q13813 | SPTAN1 | 89 | 100 | 26 | 18 | MS | D |  |
| Q14257 | RCN2 | 27 | 96 | 1 | 7 | MS/MS | B | Y24 |
| Q14929 | ZNF169 | 59 | 98 | 13 | 31 | MS | A |  |
| Q16531 | DDB1 | 38 | 100 | 2 | 2 | MS/MS | C |  |
| Q16539 | MAPK14 | 47 | 100 | 2 | 13 | MS/MS | C |  |
| Q16539 | MAPK14 | 28 | 97 | 2 | 13 | MS/MS | A |  |
| Q4V348 | ZNF658B | 60 | 98 | 16 | 29 | MS | D |  |
| Q4V348 | ZNF658B | 60 | 98 | 16 | 29 | MS | A |  |
| Q562R1 | ACTBL2 | 102 | 100 | 2 | 6 | MS/MS | A |  |
| Q6IN97 | FRMPD2L1 | 60 | 98 | 9 | 41 | MS | A |  |
| Q92616 | GCN1L1 | 66 | 100 | 25 | 13 | MS | C |  |
| Q92616 | GCN1L1 | 42 | 100 | 1 | NA | MS/MS | C | Y25 |
| Q92736 | RYR2 | 68 | 100 | 33 | 10 | MS | C |  |
| Q96JY6 | PDLIM2 | 54 | 100 | 2 | 12 | MS/MS | D |  |
| Q96M96 | FGD4 | 57 | 96 | 13 | 27 | MS | D |  |
| Q96NK8 | NEUROD6 | 31 | 98 | 1 | 4 | MS/MS | D | Y26 |
| Q96PK2 | MACF1 | 31 | 98 | 2 | <1 | MS/MS | B |  |
| Q96PK2 | MACF1 | 30 | 96 | 2 | <1 | MS/MS | D |  |
| Q96RW7 | HMCN1 | 29 | 96 | 3 | 1 | MS/MS | A |  |
| Q9BQE3 | TUBA1C | 38 | 100 | 1 | NA | MS/MS | C | Y27 |
| Q9BSJ2 | TUBGCP2 | 57 | 98 | 10 | 15 | MS | A |  |
| Q9BSJ2 | TUBGCP2 | 58 | 97 | 13 | 24 | MS | B |  |
| Q9NSV4 | DIAPH3 | 29 | 98 | 1 | 1 | MS/MS | C* | Y28 |
| Q9UL46 | PSME2 | 37 | 100 | 1 | NA | MS/MS | B | Y29 |
| Q9UL46 | PSME2 | 54 | 100 | 1 | NA | MS/MS | C | Y30 |
| Q9ULJ3 | ZNF295 | 56 | 97 | 15 | 19 | MS | C |  |
| Q14585 | ZNF345 | 26 | 94 | 1 | NA | MS/MS | B | Y31 |
| Q15021 | NCAPD2 | 26 | 94 | 1 | NA | MS/MS | D | Y32 |
| O00159 | MYO1C | 25 | 93 | 1 | NA | MS/MS | A | Y33 |
| O14639 | ABLIM1 | 23 | 90 | 1 | NA | MS/MS | B | Y34 |
| Q13219 | PAPPA | 24 | 93 | 1 | NA | MS/MS | B | Y35 |
| Q8IZT6 | ASPM | 27 | 93 | 1 | NA | MS/MS | D | Y36 |
| Q8N163 | KIAA1967 | 25 | 91 | 1 | NA | MS/MS | D | Y37 |
| Q96QE4 | LRRC37B | 25 | 91 | 1 | NA | MS/MS | C | Y38 |
