# Supplementary material for: Proteomic Analysis of Pathways Involved in Estrogen-Induced Growth and Apoptosis of Breast Cancer Cells
Source: PLoS One. 2011 Jun 27;6(6):e20410. doi: 10.1371/journal.pone.0020410 (PMC3124472; doi:10.1371/journal.pone.0020410)
Supplement: Table S6 — MS/MS spectra for single peptide identified AIB1-complexed proteins. The “No.” column labels the spectra sequentially as referenced in Table S4. The “Exp.” column indicates the experimental conditions under which the respective protein was identified: A, MCF-7 cells, no E2; B, MCF-7:5C cell, no E2; C, MCF-7 cell, +E2; D, MCF-7:5C cell, +E2. The underlined C and M in peptide sequences represent fixed (carbamidomethyl) and variable (oxidation) modifications, respectively. *MALDI-TOF-MS generates peptides containing only one charge and the precursor m/z (not shown) is thus equal to the precursor mass. (DOC) [file pone.0020410.s014.doc]

| **No.** | **UniProtKB AC** | **Gene name** | **Score** | **CI%** | **Peptide sequence** | **Precursor mass*** | **Exp.** |
| --- | --- | --- | --- | --- | --- | --- | --- |
|  | | | | | | | |
| A1 | O14917 | PCDH17 | 31 | 98 | ILDHNDNPPR | 1190.6202 | A |
| 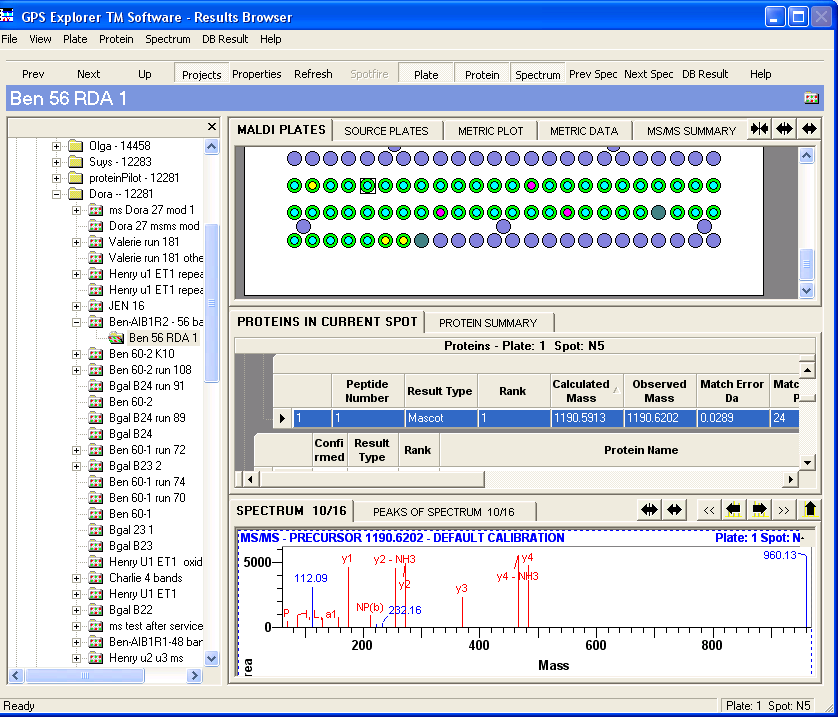 | | | | | | | |
| A2 | O14917 | PCDH17 | 31 | 98 | ILDHNDNPPR | 1190.6471 | C |
| 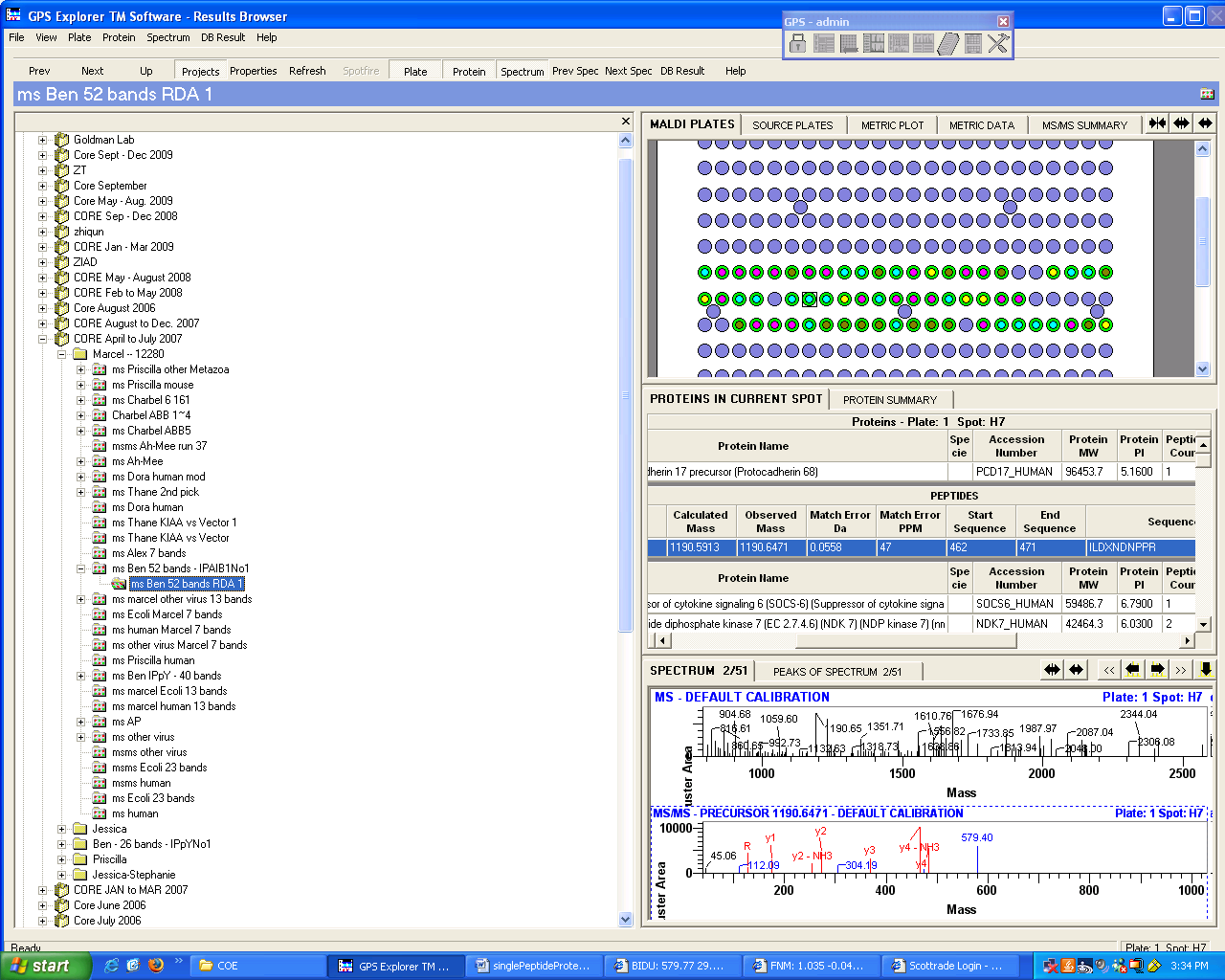 | | | | | | | |
| A3 | O75094 | SLIT3 | 43 | 100 | RTVGQFTLCMAPVHLR | 1901.9534 | A |
| 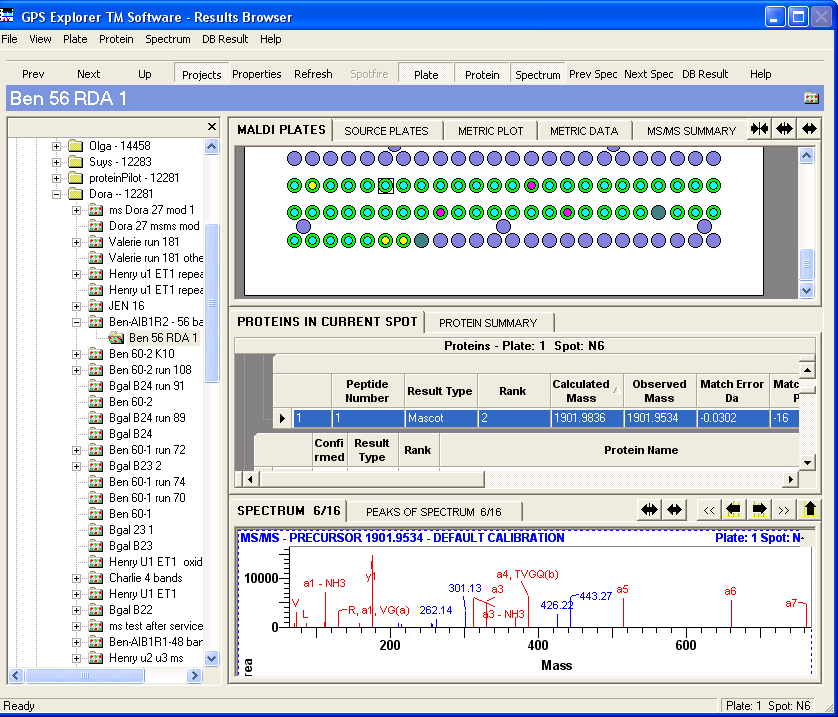 | | | | | | | |
| A4 | O75094 | SLIT3 | 28 | 96 | RTVGQFTLCMAPVHLR | 1901.9668 | C |
| 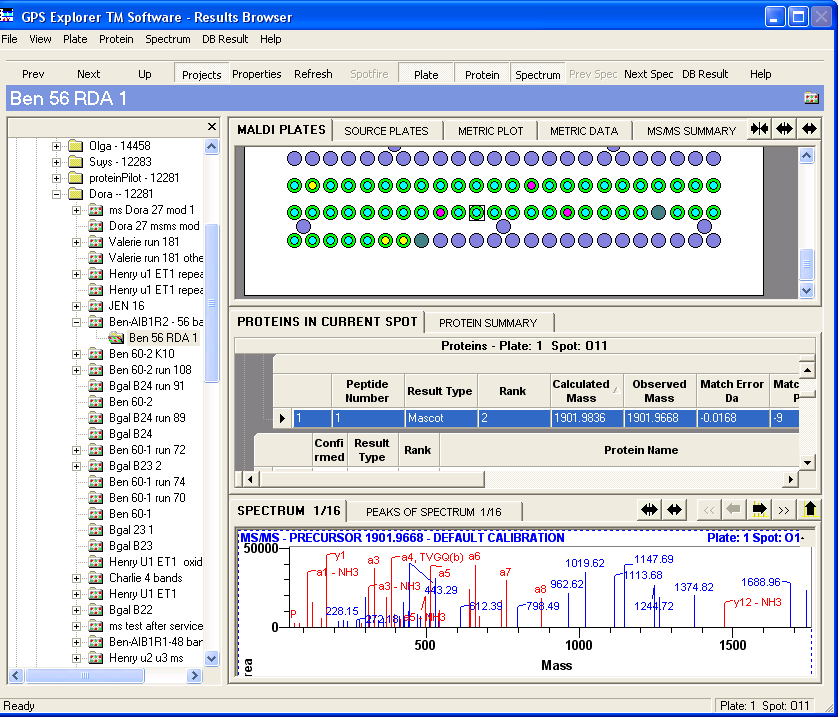 | | | | | | | |
| A5 | O75131 | CPNE3 | 27 | 98 | SSPVEFECINEKK | 1509.7249 | A |
| 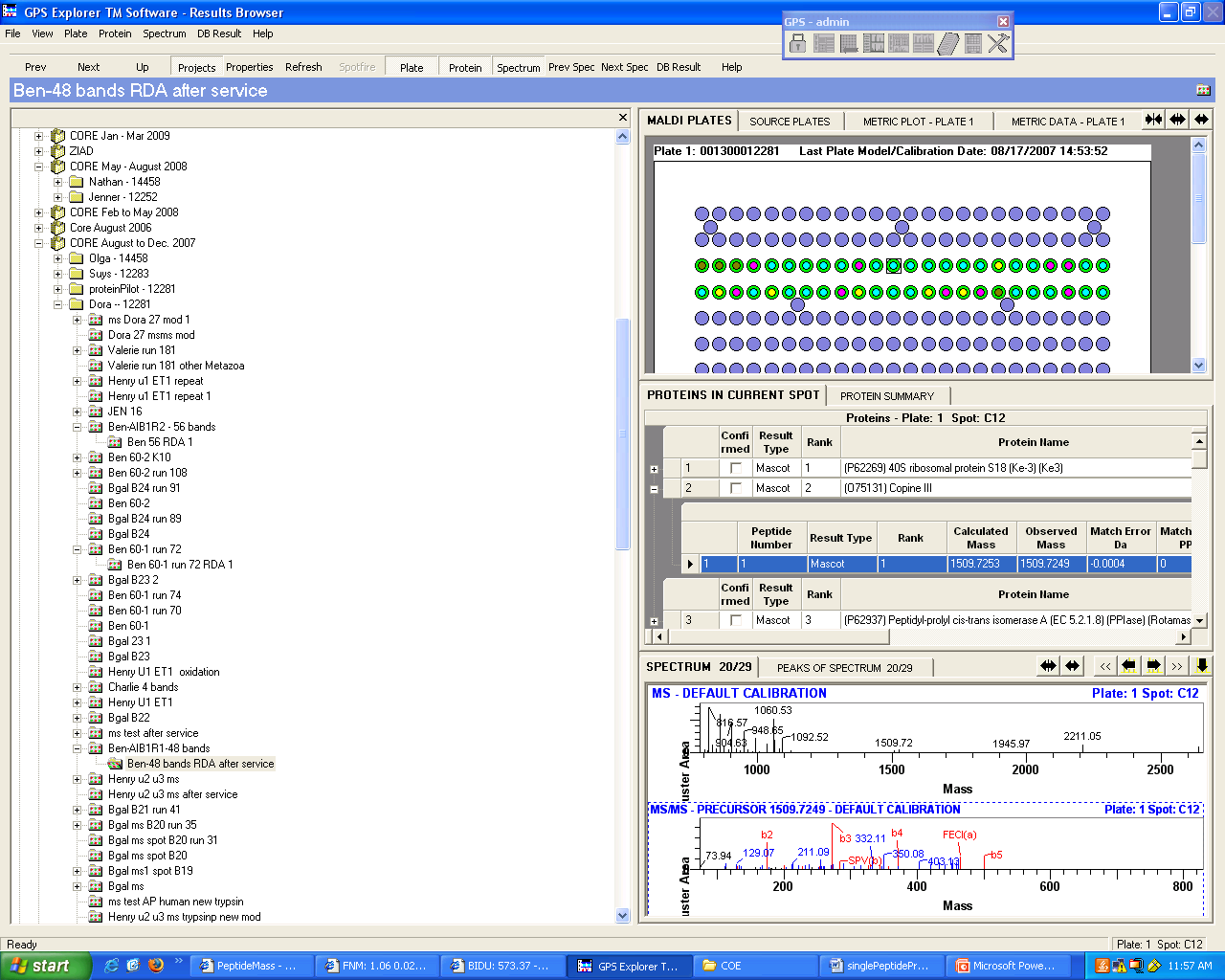 | | | | | | | |
| A6 | O75131 | CPNE3 | 27 | 98 | SSPVEFECINEKK | 1509.7714 | B |
| 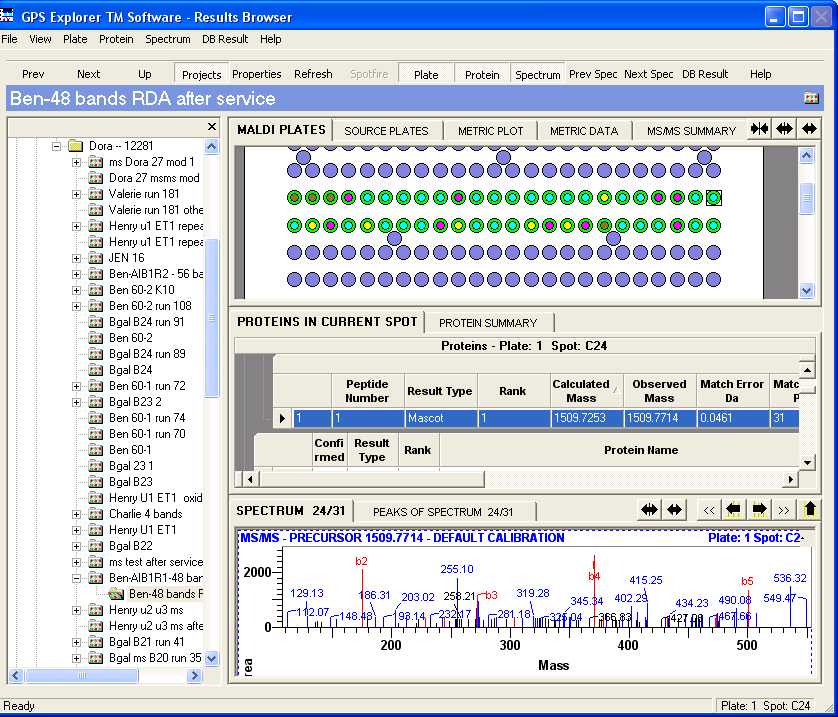 | | | | | | | |
| A7 | P04406 | GAPDH | 76 | 100 | LISWYDNEFGYSNR | 1763.84 | D |
| 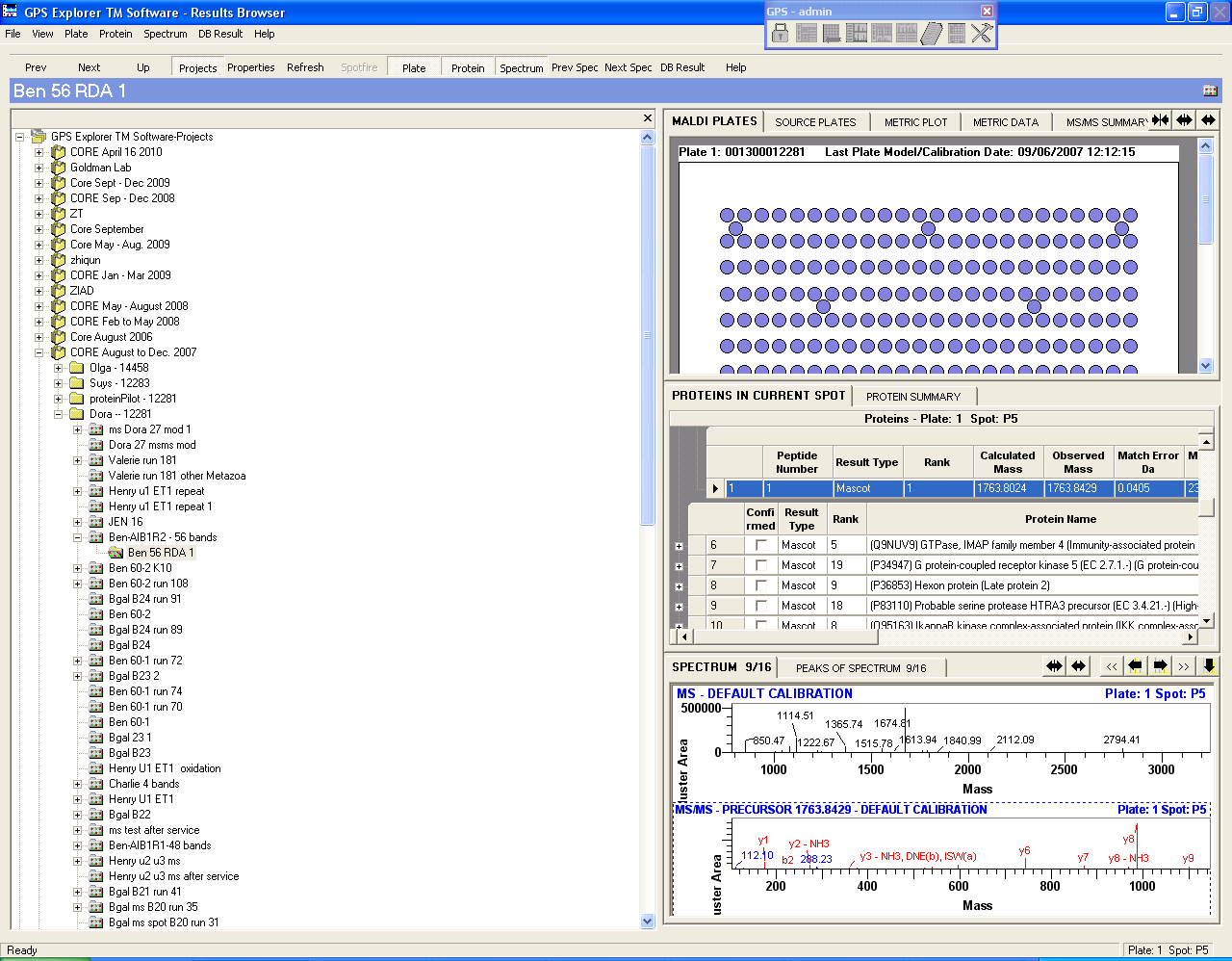 | | | | | | | |
| A8 | P04899 | GNAI2 | 29 | 97 | IAQSDYIPTQQDVLR | 1746.8706 | A |
| 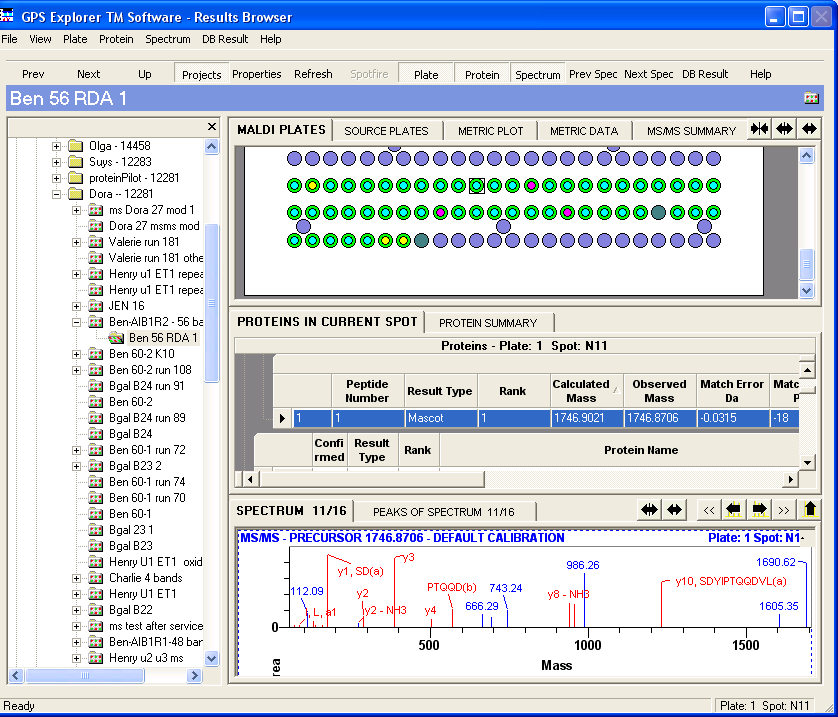 | | | | | | | |
| A9 | P49327 | FASN | 29 | 97 | DNLEFFLAGIGR | 1351.6877 | C |
| 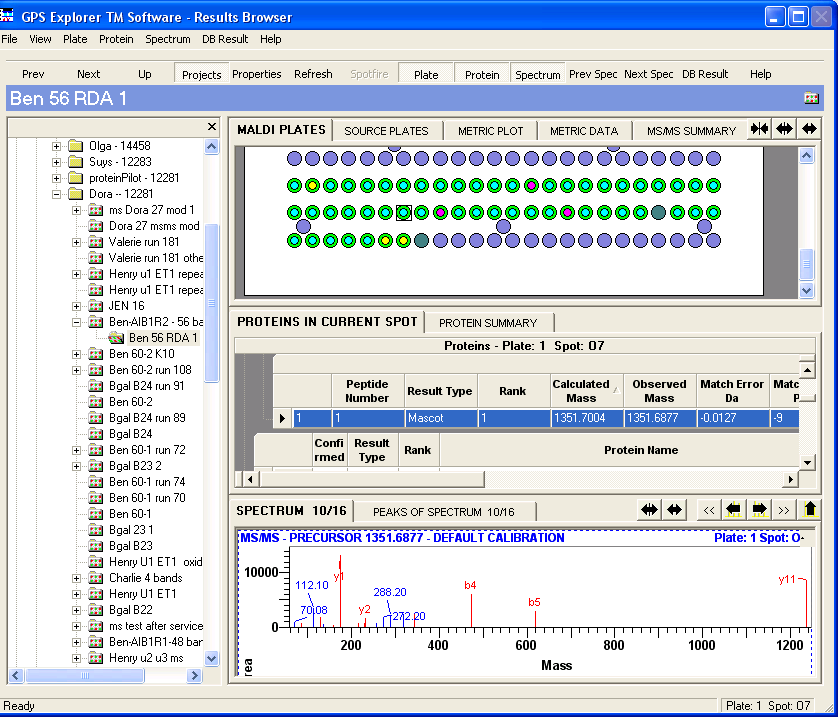 | | | | | | | |
| A10 | P50053 | KHK | 29 | 97 | EDSEIRCLSQR | 1335.6774 | D |
| 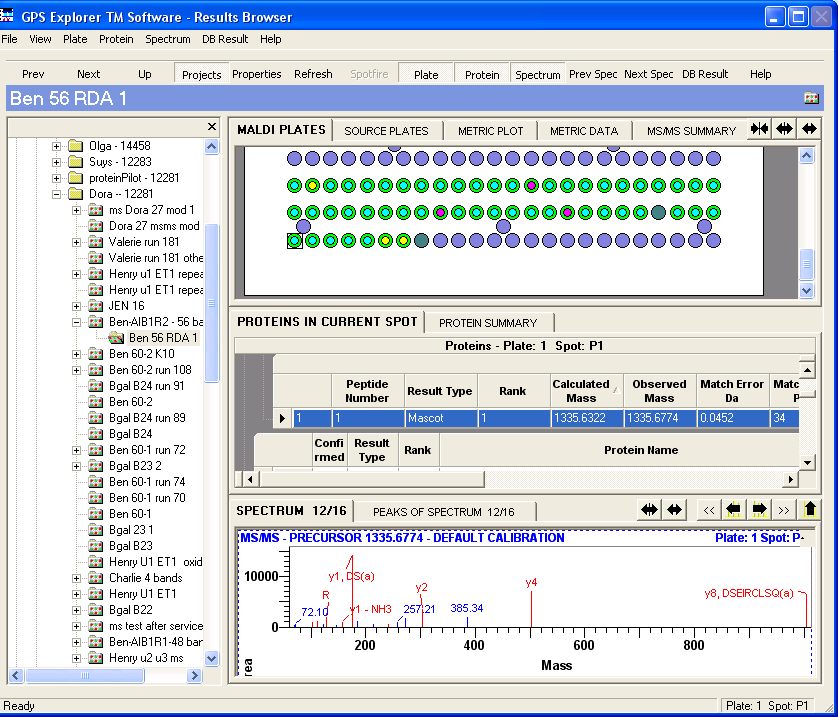 | | | | | | | |
| A11 | P60709 | ACTB | 70 | 100 | SYELPDGQVITIGNER | 1790.9302 | D |
| 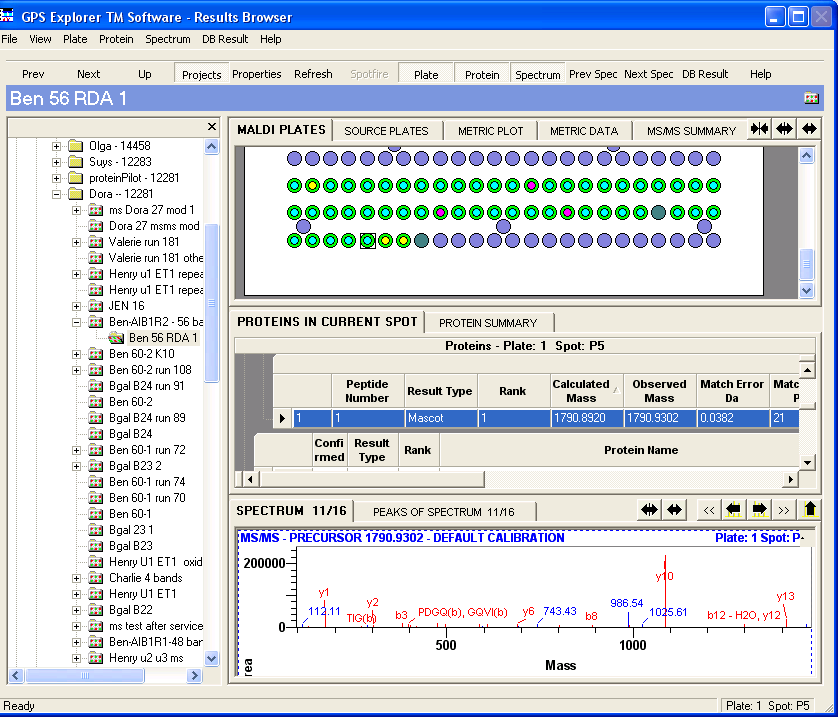 | | | | | | | |
| A12 | P62158 | CALM1 | 49 | 100 | VFDKDGNGYISAAELR | 1754.9078 | C |
| 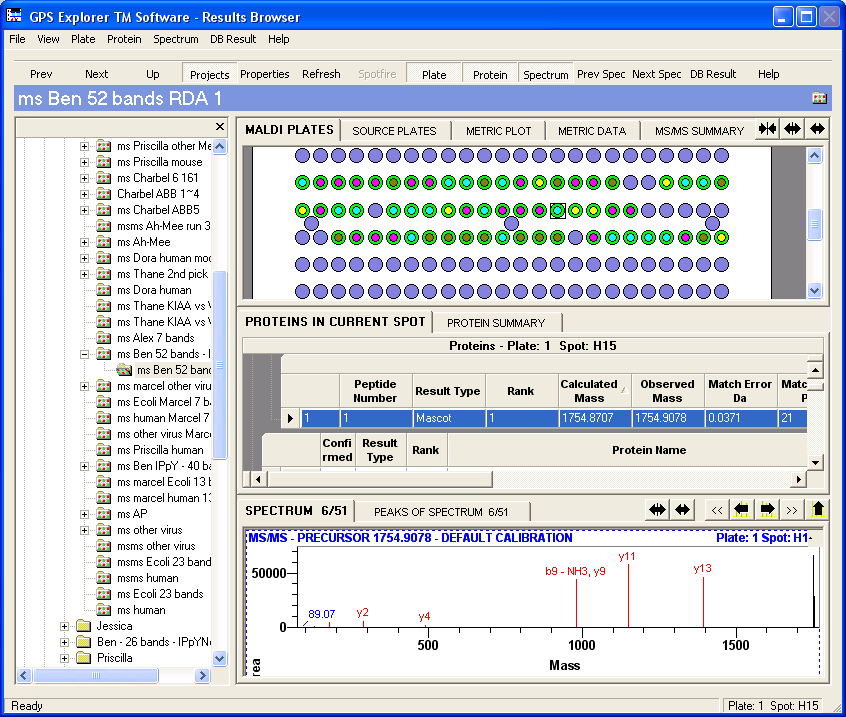 | | | | | | | |
| A13 | P62269 | RPS18 | 38 | 100 | IPDWFLNR | 1060.527 | A |
| 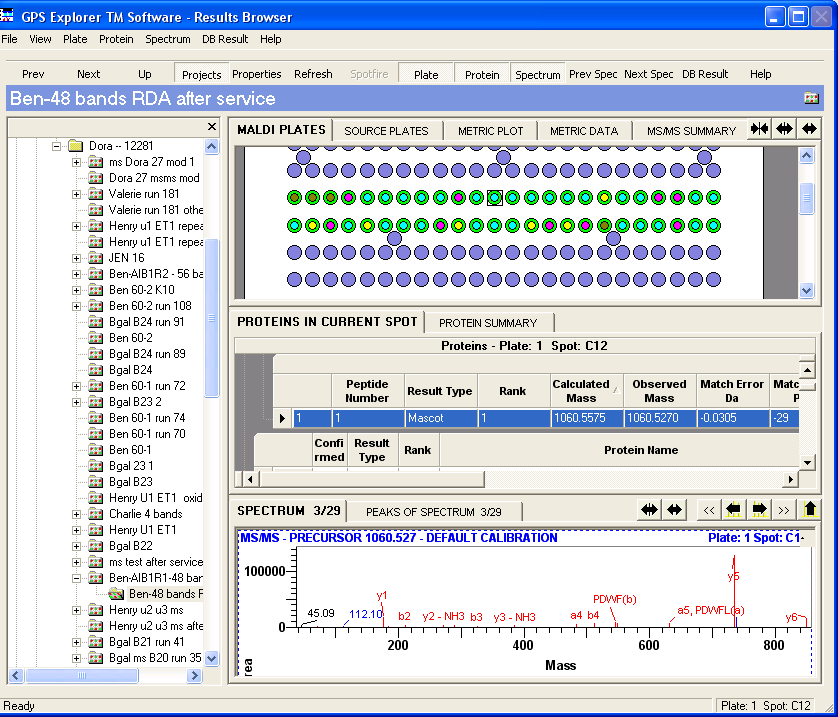 | | | | | | | |
| A14 | P62269 | RPS18 | 61 | 100 | IPDWFLNR | 1060.5648 | B |
| 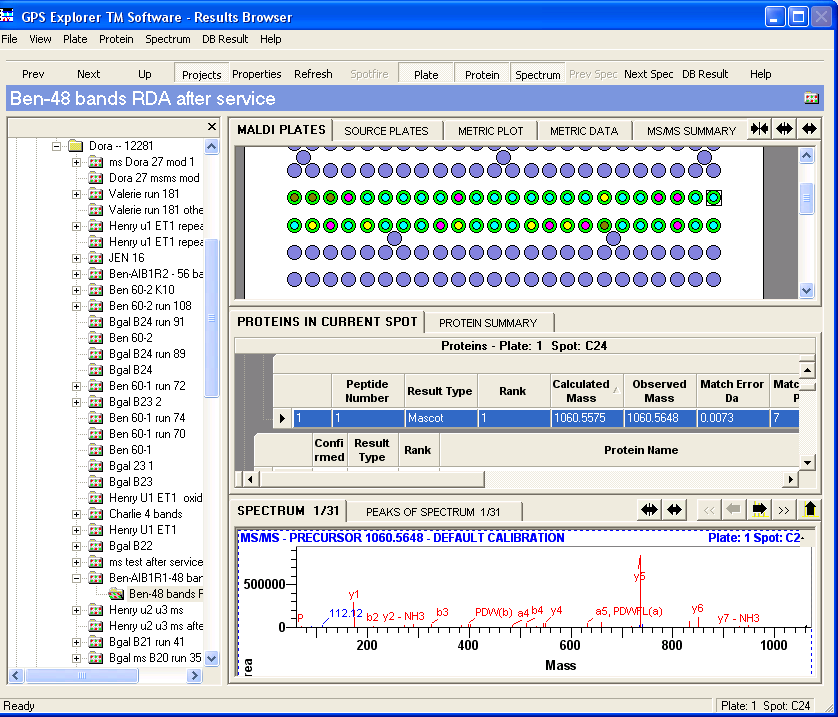 | | | | | | | |
| A15 | P62269 | RPS18 | 59 | 100 | IPDWFLNR | 1060.5182 | C |
| 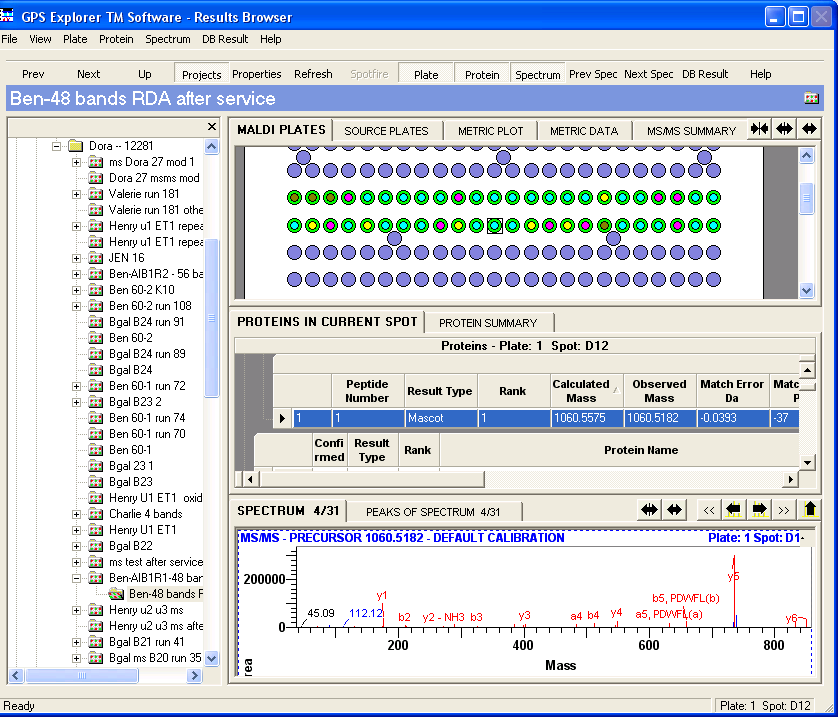 | | | | | | | |
| A16 | P62269 | RPS18 | 60 | 100 | IPDWFLNR | 1060.5426 | D |
| 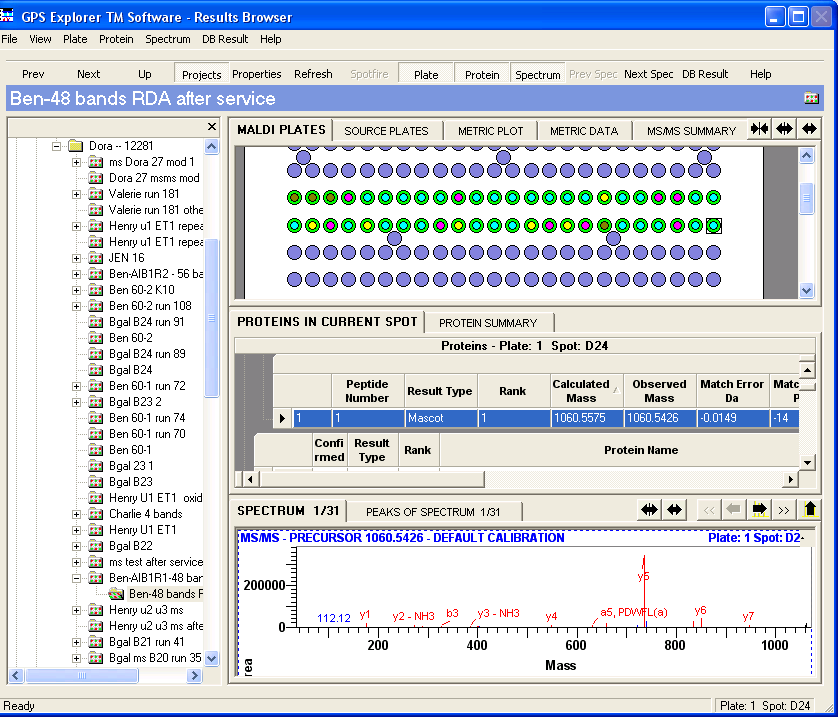 | | | | | | | |
| A17 | P62937 | PPIA | 29 | 99 | VNPTVFFDIAVDGEPLGR | 1945.941 | C |
| 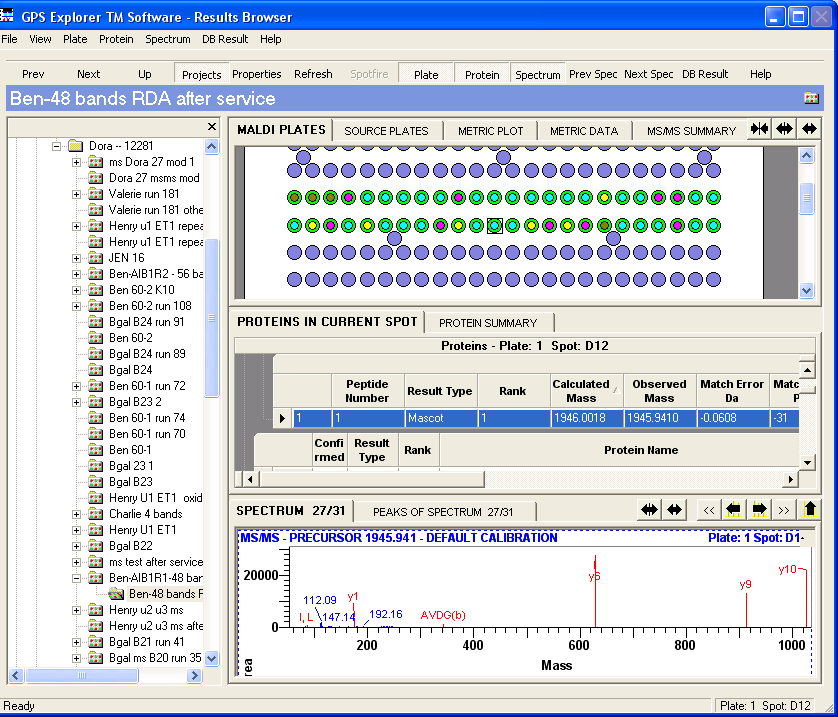 | | | | | | | |
| A18 | Q04726 | TLE3 | 28 | 96 | SMPGKPPGMDPIGIMASALR | 2025.9622 | D |
| 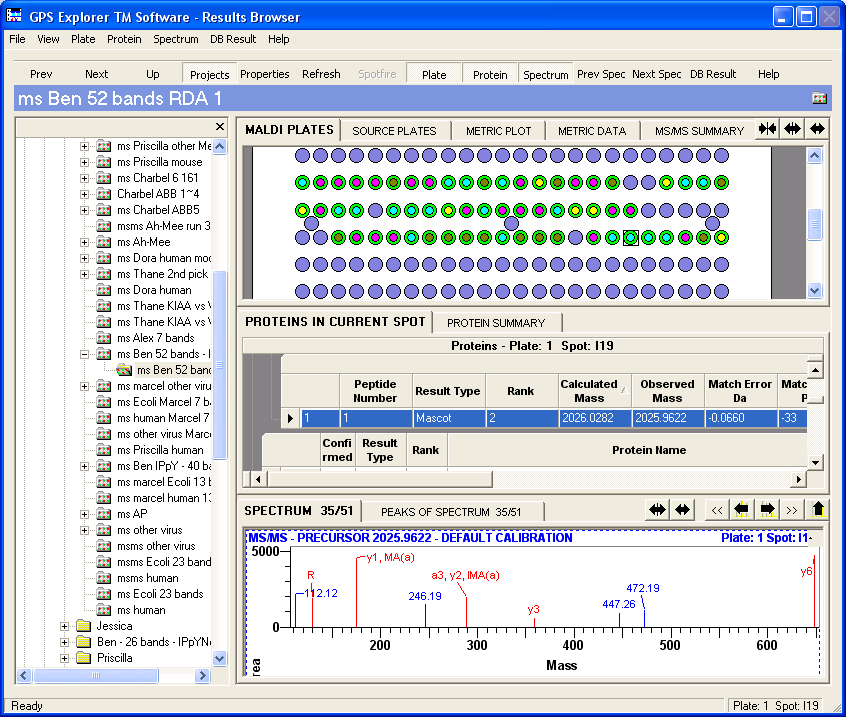 | | | | | | | |
| A19 | Q12926 | ELAVL2 | 29 | 97 | GFGFVTMTNYDEAAMAIR | 2025.9622 | D |
| 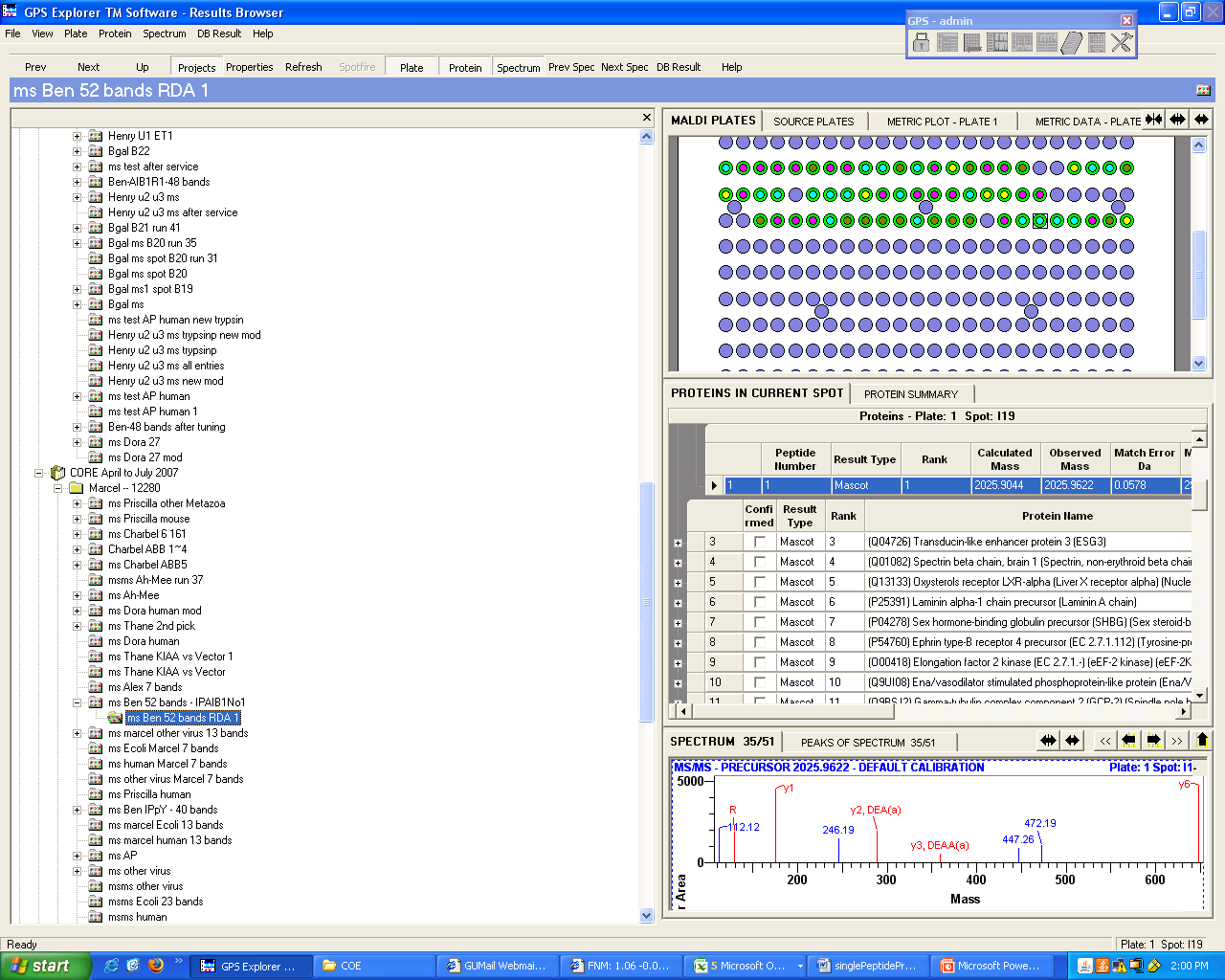 | | | | | | | |
| A20 | Q15019 | ZN169 | 31 | 98 | STLINSLFLTDLYPER | 1881.9855 | C |
| 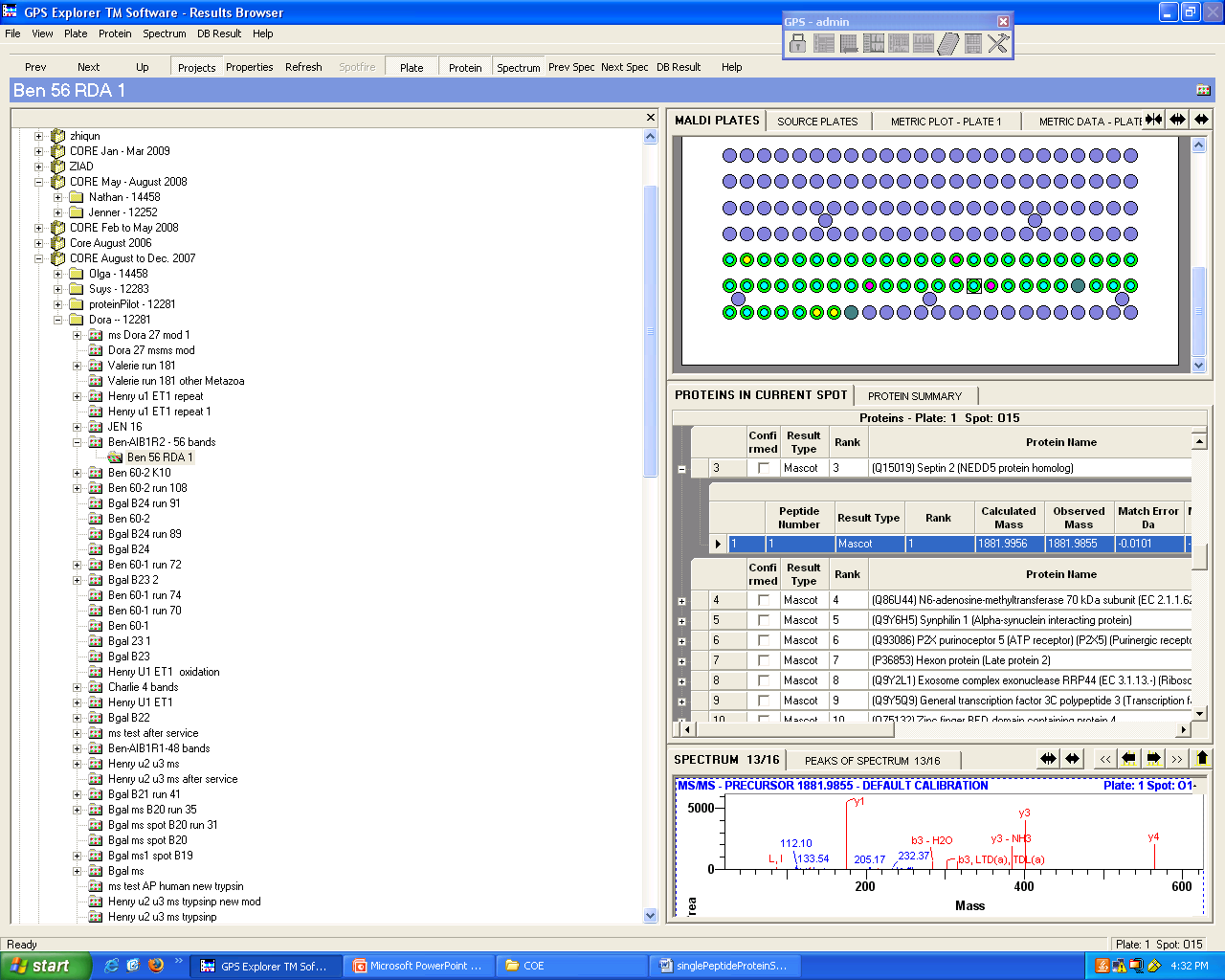 | | | | | | | |
| A21 | Q7Z407 | CSMD3 | 23 | 95 | KQPMTLTVTSFNASTGR | 1838.8966 | C |
| 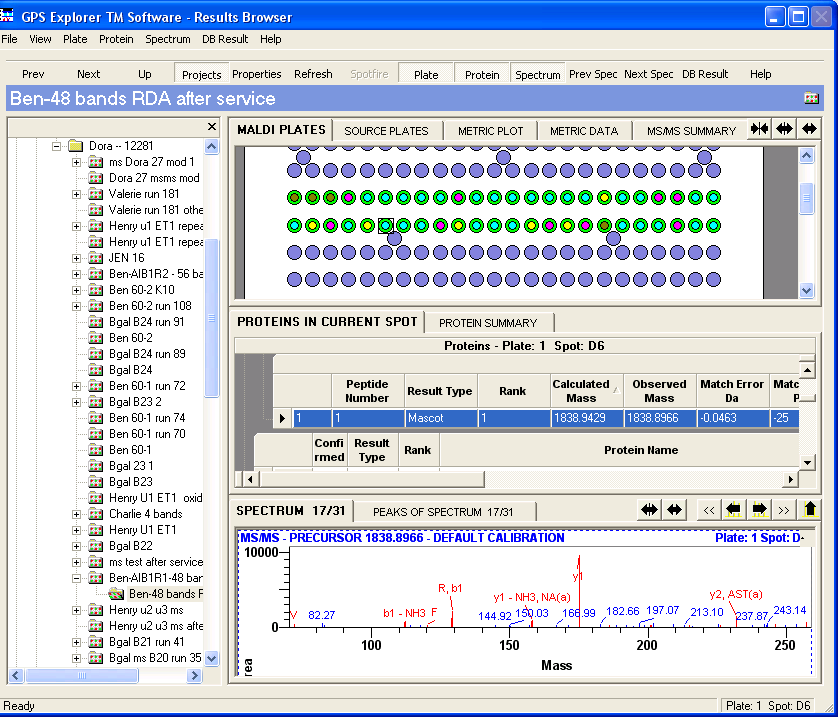 | | | | | | | |
| A22 | Q86TI0 | TBC1D1 | 23 | 95 | KQNLDLLEQLQVANGR | 1838.8966 | C |
| 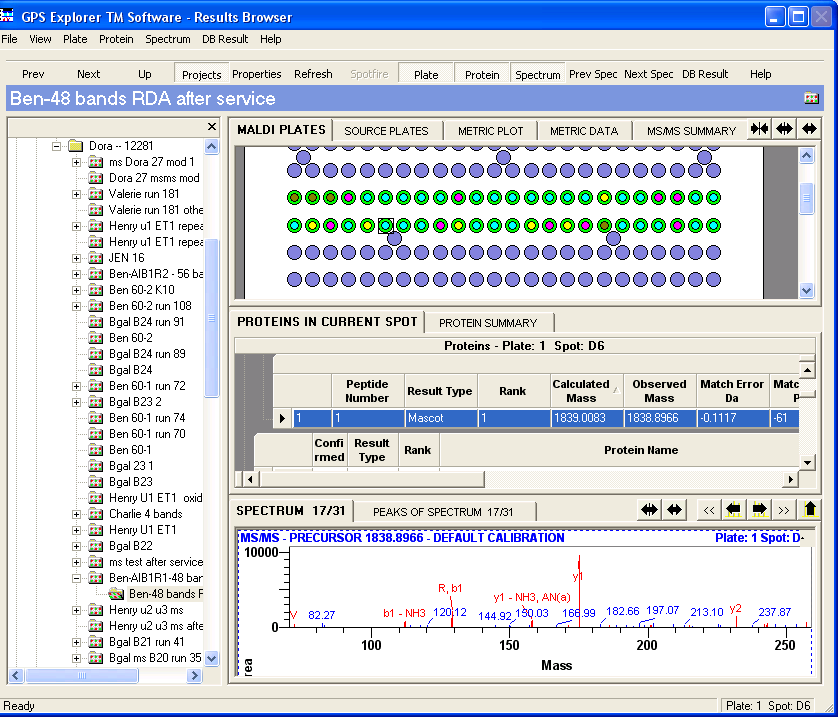 | | | | | | | |
| A23 | Q92738 | USP6NL | 36 | 99 | AYSQSPRHALYPPSPR | 1826.8999 | A |
| 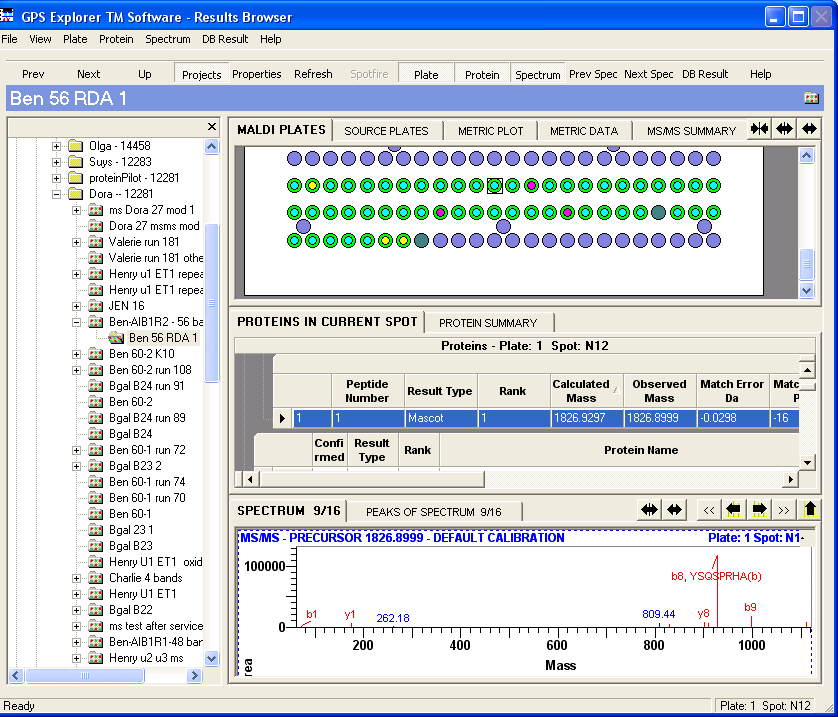 | | | | | | | |
| A24 | Q92738 | USP6NL | 26 | 95 | AYSQSPRHALYPPSPR | 1827.0109 | D |
| 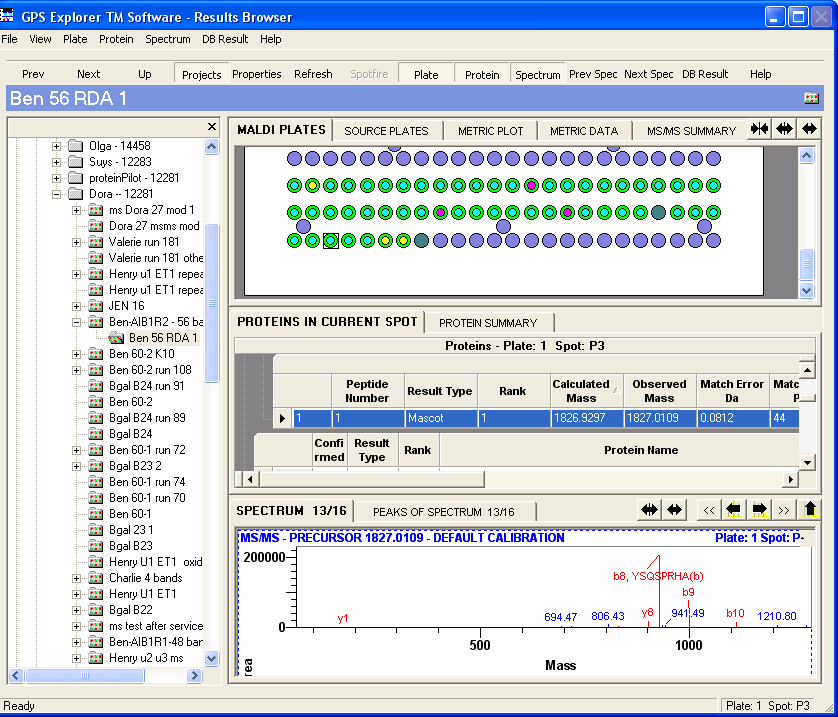 | | | | | | | |
| A25 | Q96A11 | GAL3ST3 | 28 | 98 | DDAAYLAGLIR | 1177.6764 | D |
| 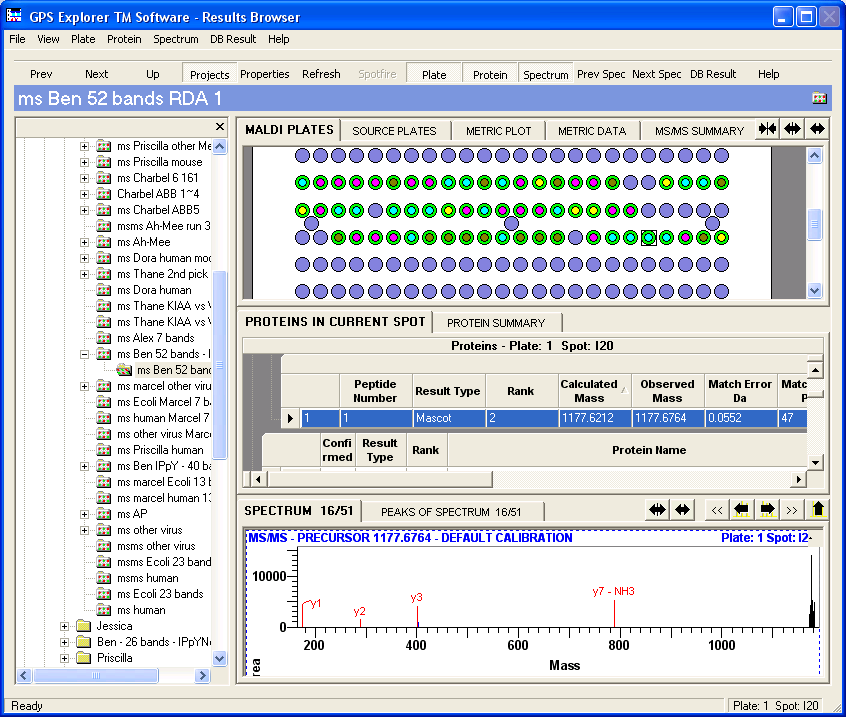 | | | | | | | |
| A26 | Q9BVK2 | ALG8 | 31 | 98 | WKSFSFVR | 1056.5354 | C |
| 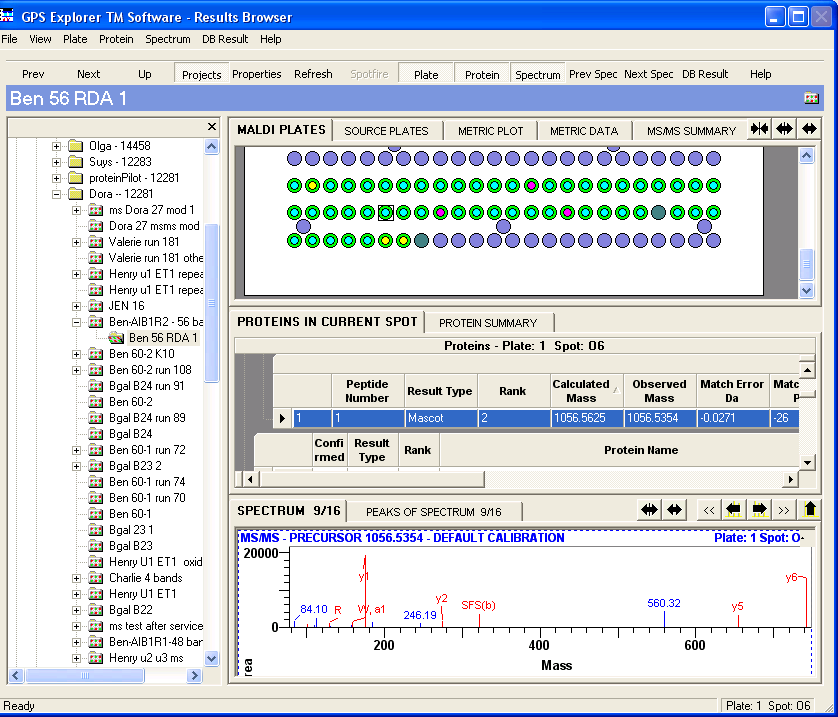 | | | | | | | |
| A27 | Q9BZL6 | PRKD2 | 61 | 100 | SVVGTPAYLAPEVLLNQGYNR | 2261.1597 | C |
| 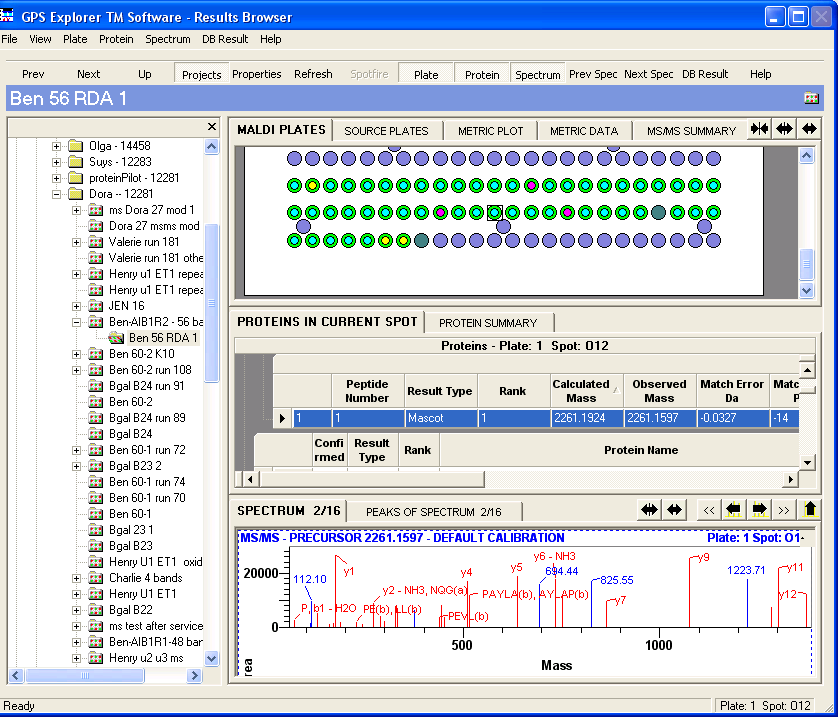 | | | | | | | |
| A28 | Q9BZL6 | PRKD2 | 32 | 99 | SVVGTPAYLAPEVLLNQGYNR | 2261.1318 | A |
| 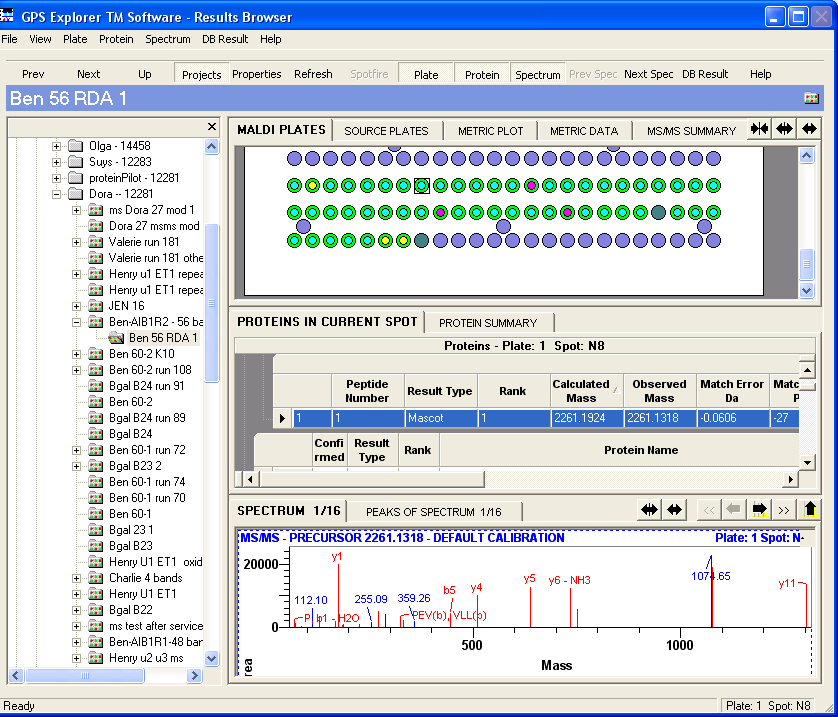 | | | | | | | |
| A29 | Q9HAU5 | UPF2 | 27 | 95 | ALFIVPR | 815.5336 | D |
| 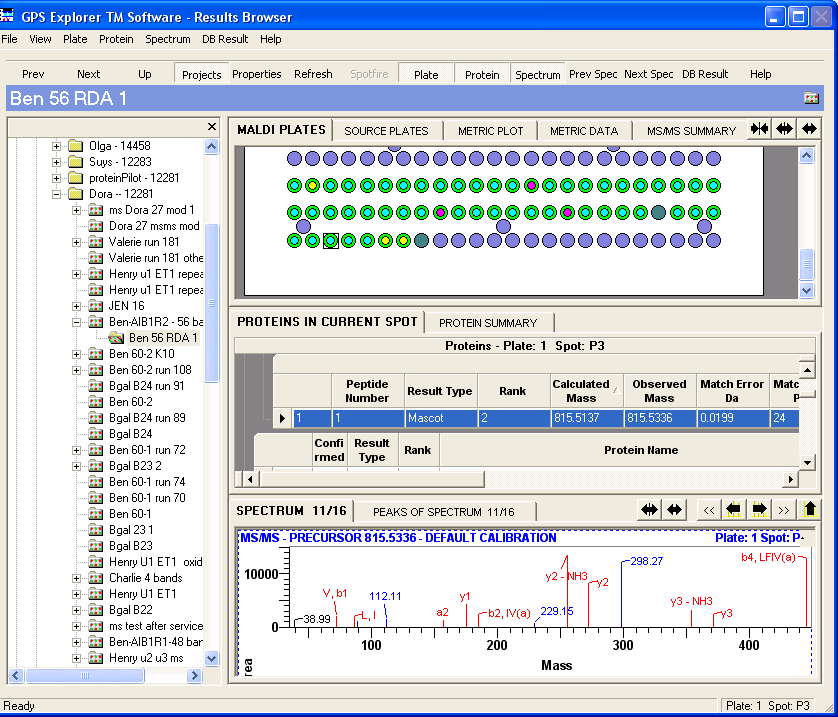 | | | | | | | |
| A30 | Q9NQX1 | PRDM5 | 29 | 97 | ALFRTPFSLQR | 1335.6774 | D |
| 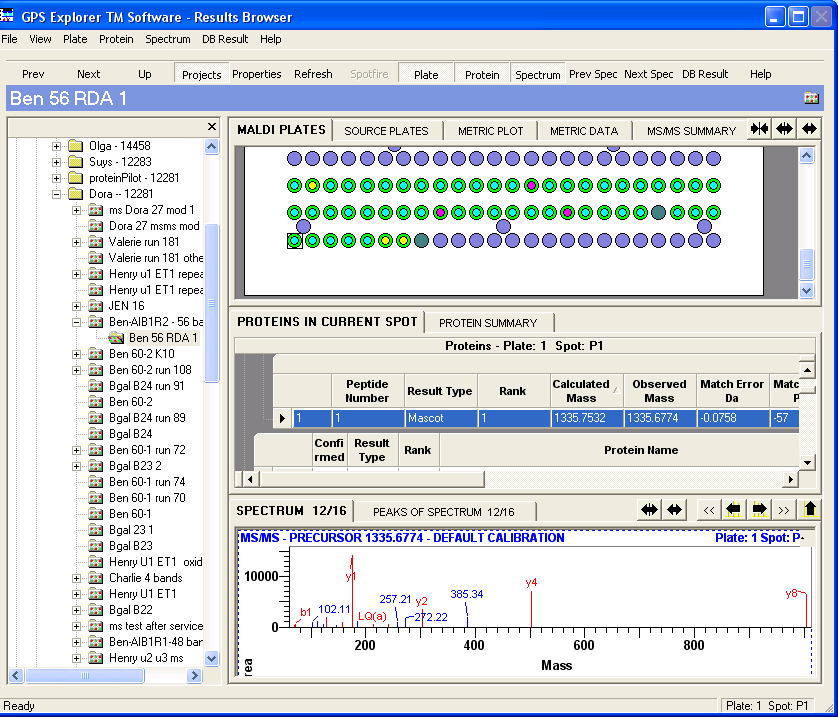 | | | | | | | |
| A31 | O14841 | OPLAH | 22 | 93 | RILEQEAGMLLPR | 1525.745 | D |
| 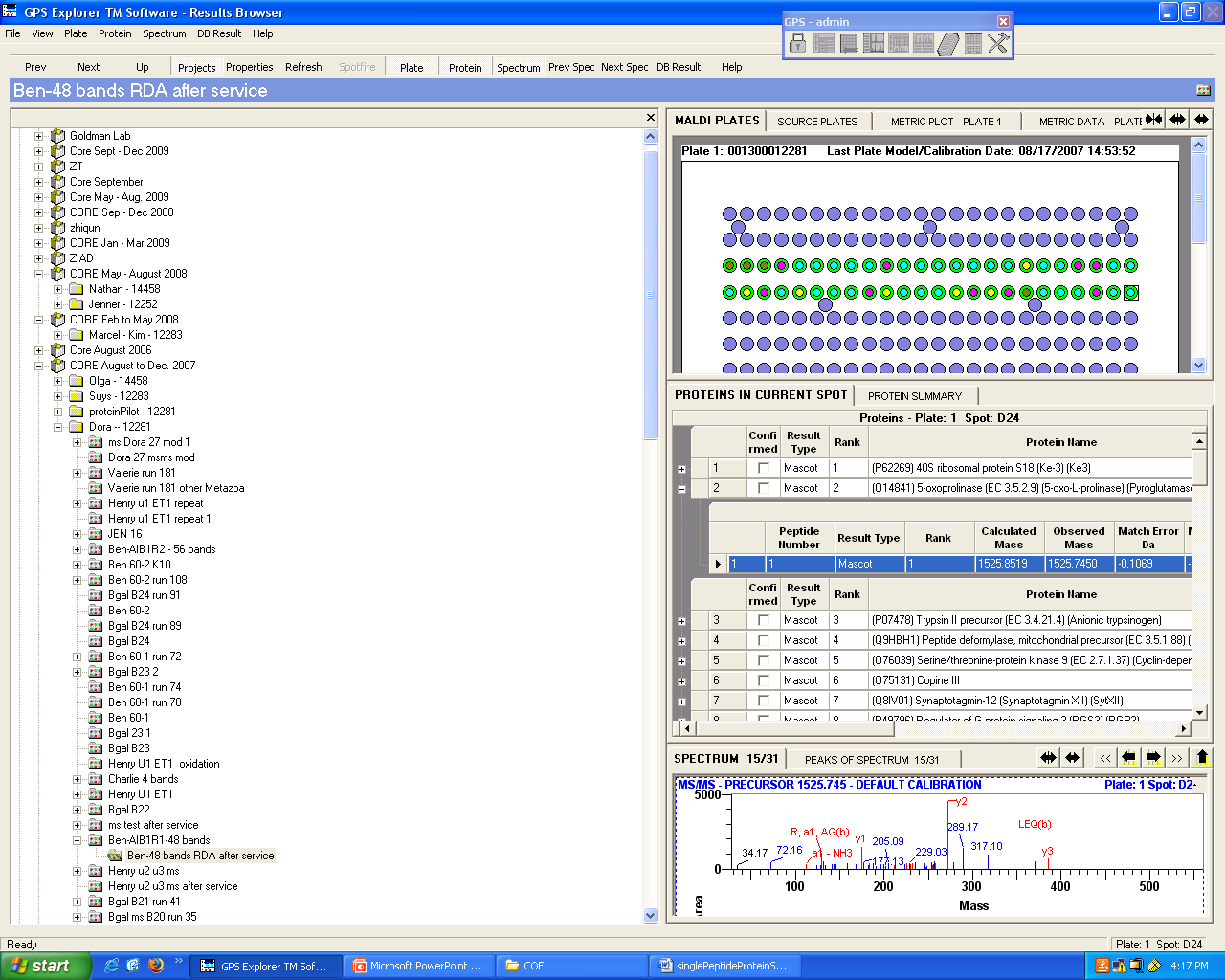 | | | | | | | |
