# Supplementary material for: Proteomic Analysis of Pathways Involved in Estrogen-Induced Growth and Apoptosis of Breast Cancer Cells
Source: PLoS One. 2011 Jun 27;6(6):e20410. doi: 10.1371/journal.pone.0020410 (PMC3124472; doi:10.1371/journal.pone.0020410)
Supplement: Table S7 — MS/MS spectra for single peptide identified pY-complexed proteins. The “No.” column labels the spectra sequentially as referenced in Table S5. The “Exp.” column indicates the experimental conditions under which the protein was identified: A, MCF-7 cells, no E2; B, MCF-7:5C cell, no E2; C, MCF-7 cell, +E2; D, MCF-7:5C cell, +E2. The underlined C and M in peptide sequences represent fixed (carbamidomethyl) and variable (oxidation) modifications, respectively. *MALDI-TOF-MS generates peptides containing only one charge and the precursor m/z (not shown) is thus equal to the precursor mass. (DOC) [file pone.0020410.s015.doc]

| **No.** | **UniProtKB AC** | **Gene name** | **Score** | **CI%** | **Peptide sequence** | **Precursor mass*** | **Exp.** |
| --- | --- | --- | --- | --- | --- | --- | --- |
|  | | | | | | | |
| Y1 | P05388 | RPLP0 | 36 | 99 | AFLADPSAFVAAAPVAAATTAAPAAAAAPAK | 2752.3799 | A |
| 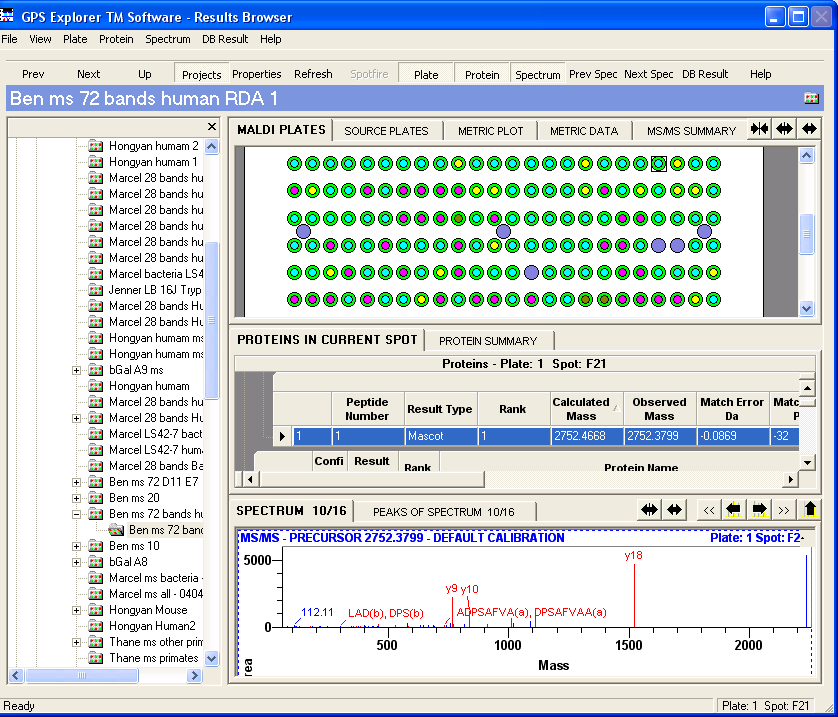 | | | | | | | |
| Y2 | P08107 | HSPA1A | 51 | 100 | QTQIFTTYSDNQPGVLIQVYEGER | 2786.3545 | A |
| 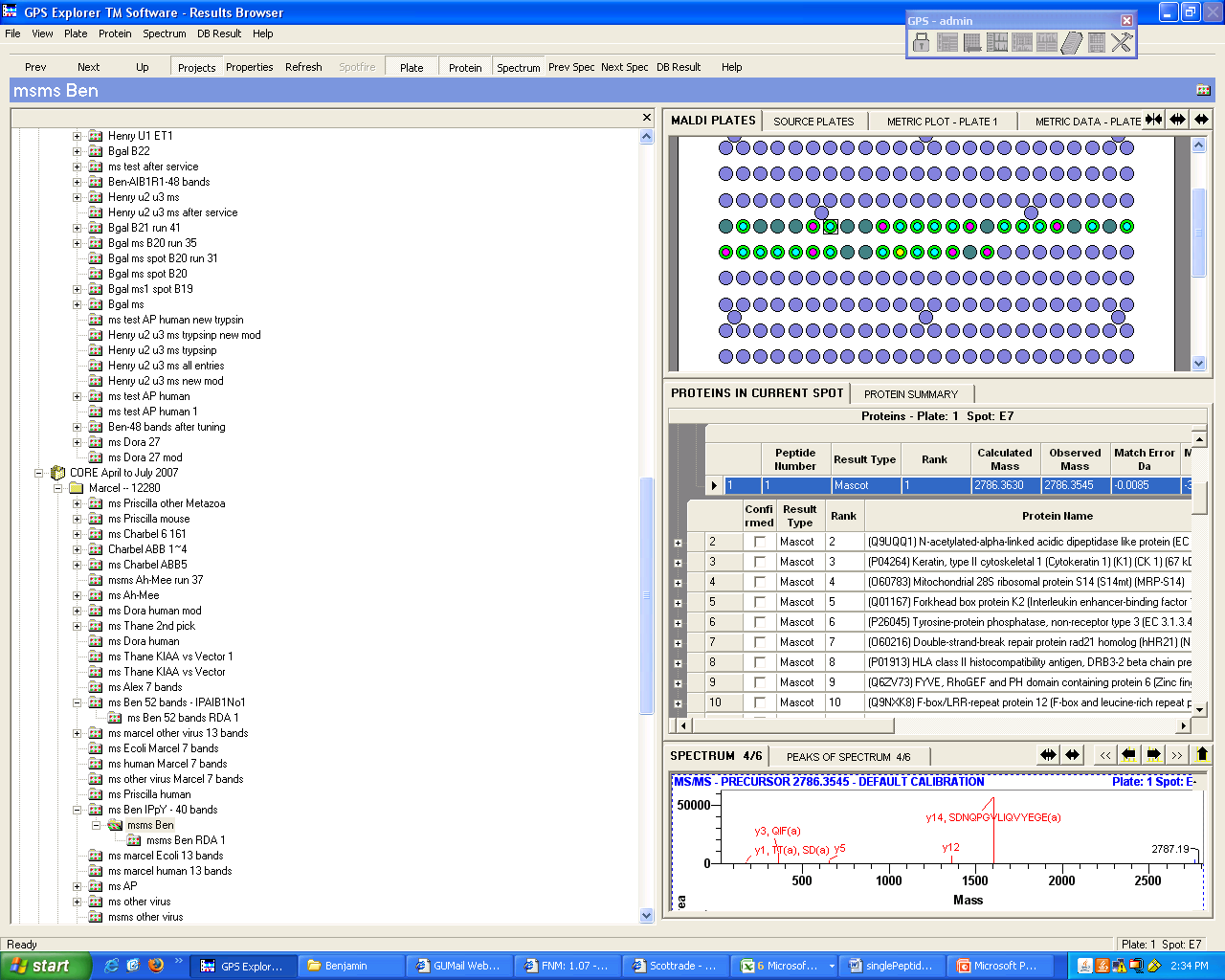 | | | | | | | |
| Y3 | P17066 | HSPA6 | 42 | 100 | IINEPTAAAIAYGLDR | 1687.851 | D |
| 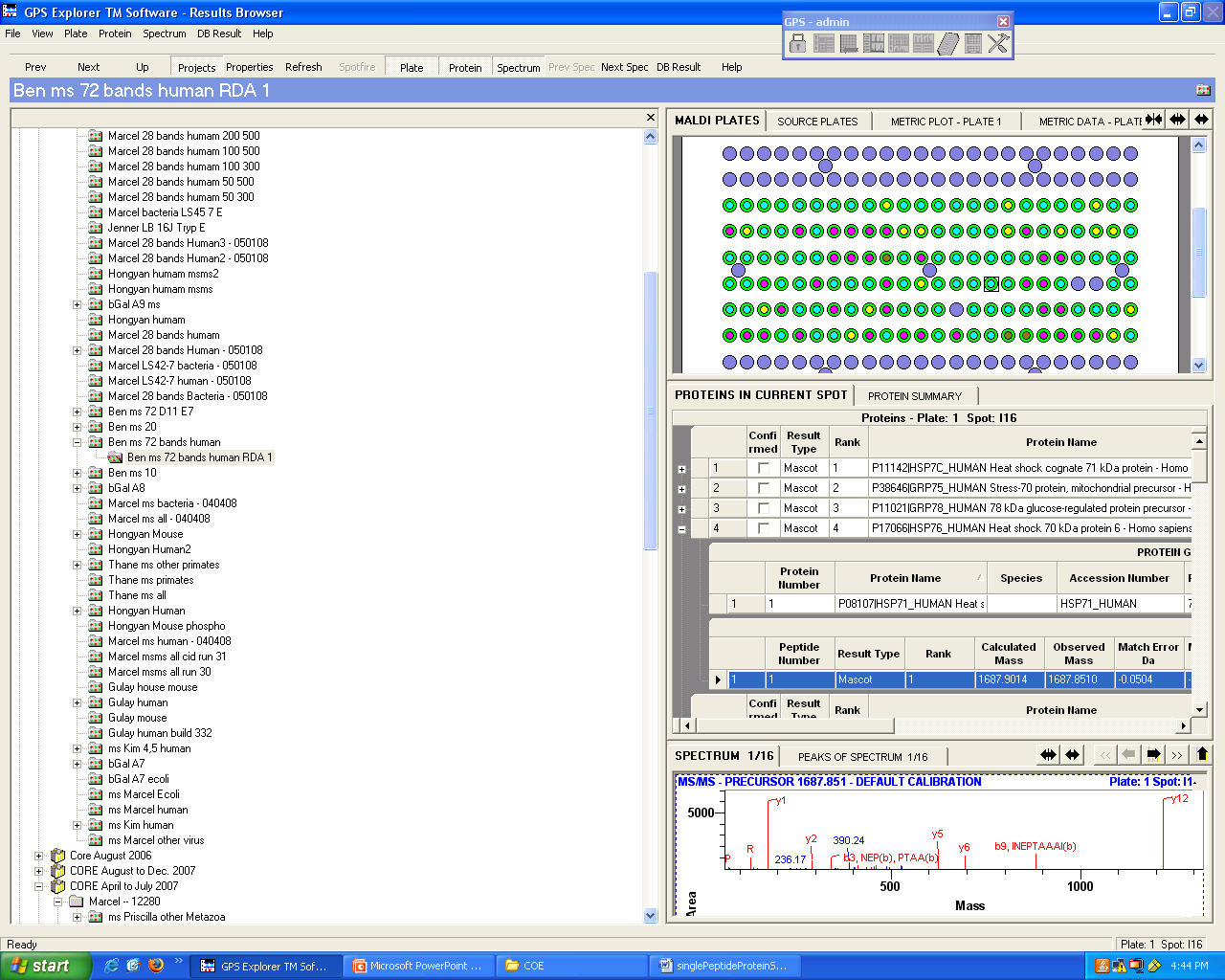 | | | | | | | |
| Y4 | P26045 | PTPN3 | 32 | 98 | VDSEPVLVHCSAGIGR | 1638.8608 | D |
| 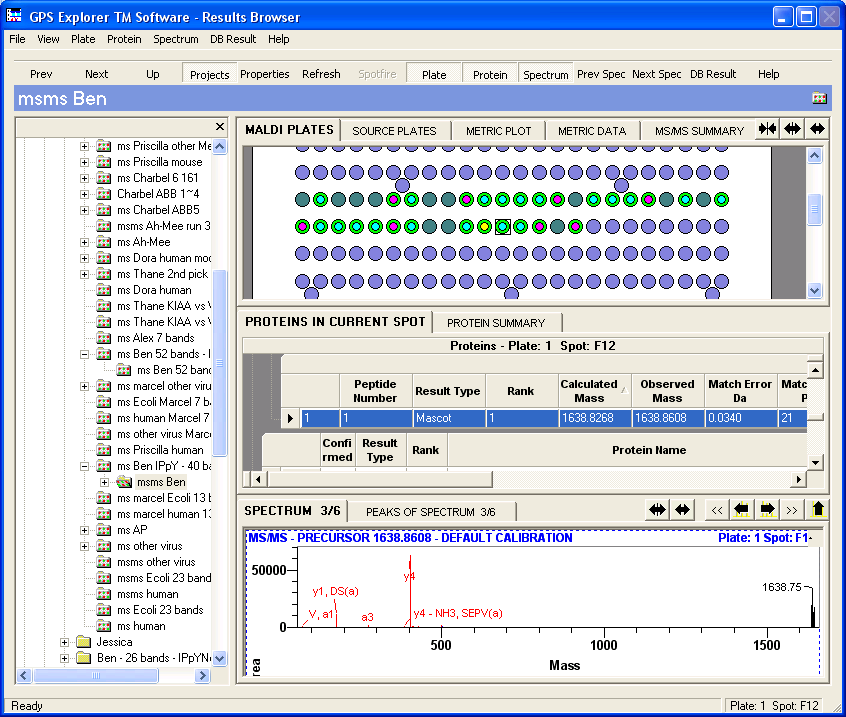 | | | | | | | |
| Y5 | P27824 | CANX | 47 | 100 | KIPNPDFFEDLEPFR | 1863.8566 | D |
| 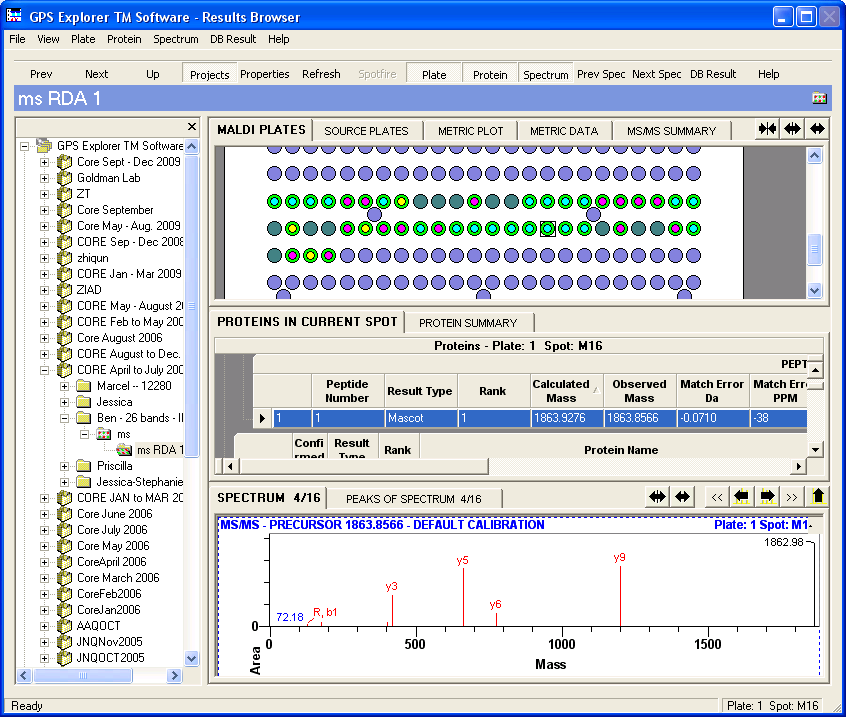 | | | | | | | |
| Y6 | P29728 | OAS2 | 28 | 97 | SSDLPGGEFSTCFTVLQR | 2000.9736 | C |
| 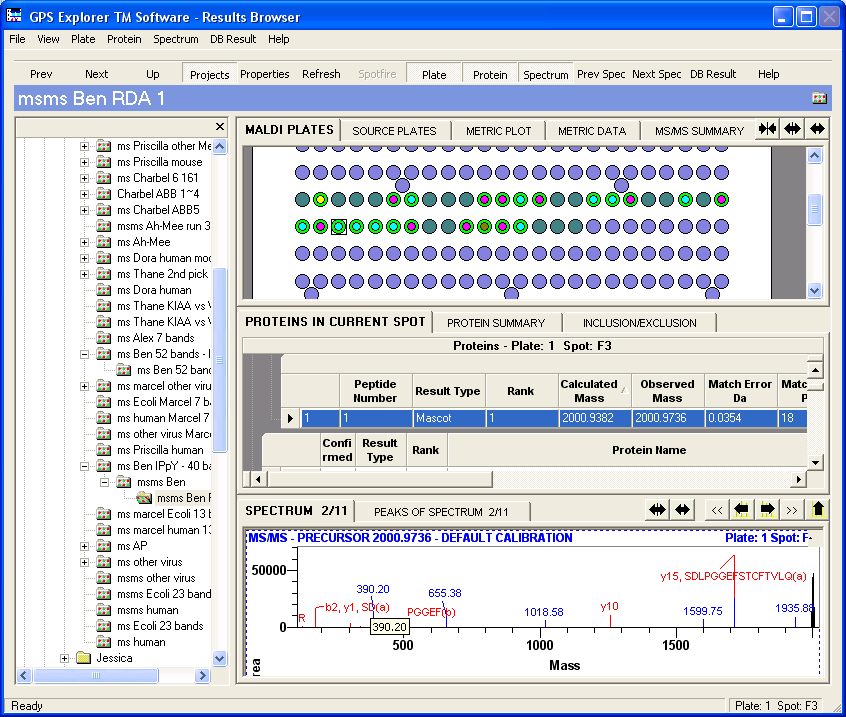 | | | | | | | |
| Y7 | P30050 | RPL12 | 46 | 100 | HSGNITFDEIVNIAR | 1685.8051 | B |
| 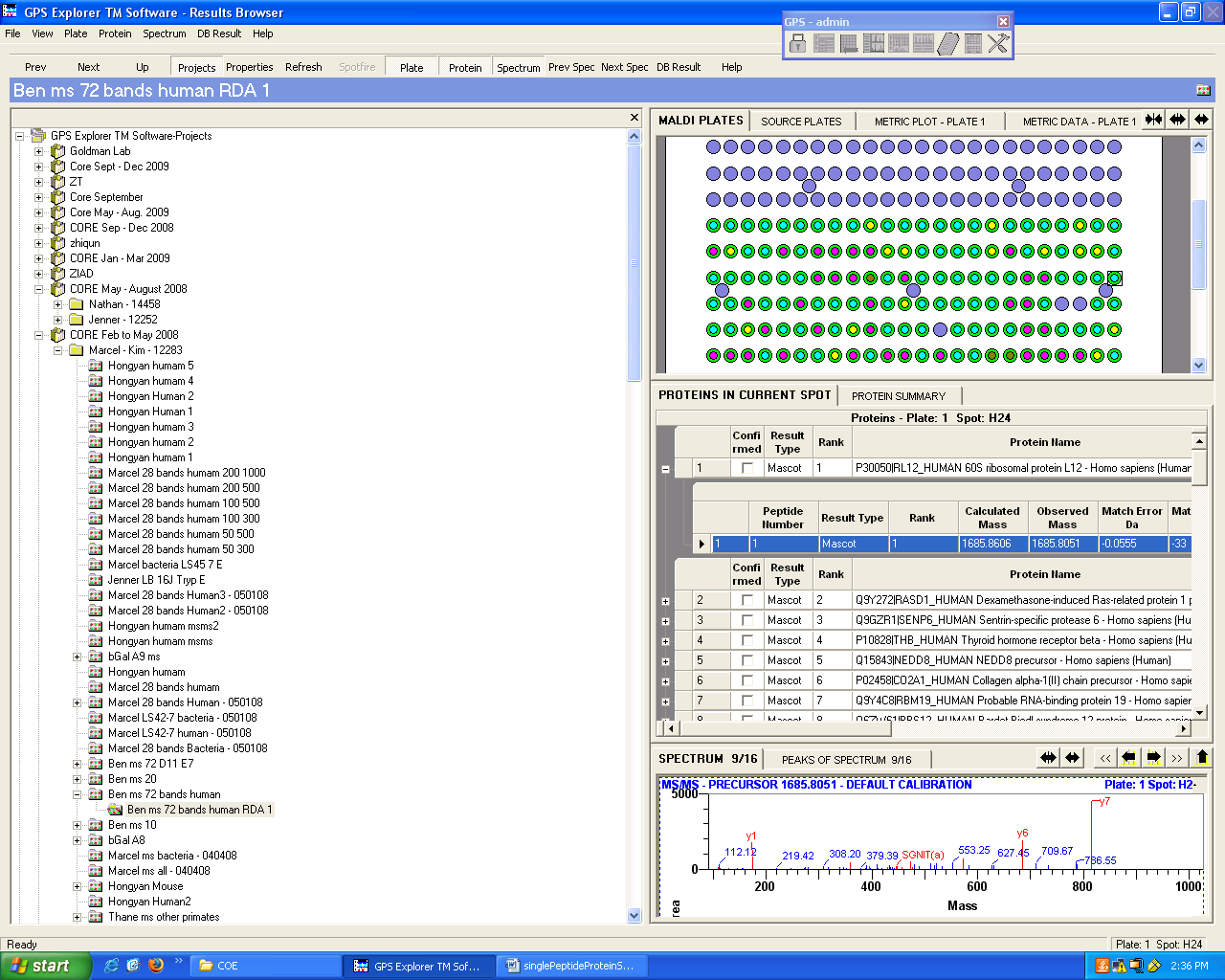 | | | | | | | |
| Y8 | P30050 | RPL12 | 35 | 99 | HSGNITFDEIVNIAR | 1685.835 | D |
| 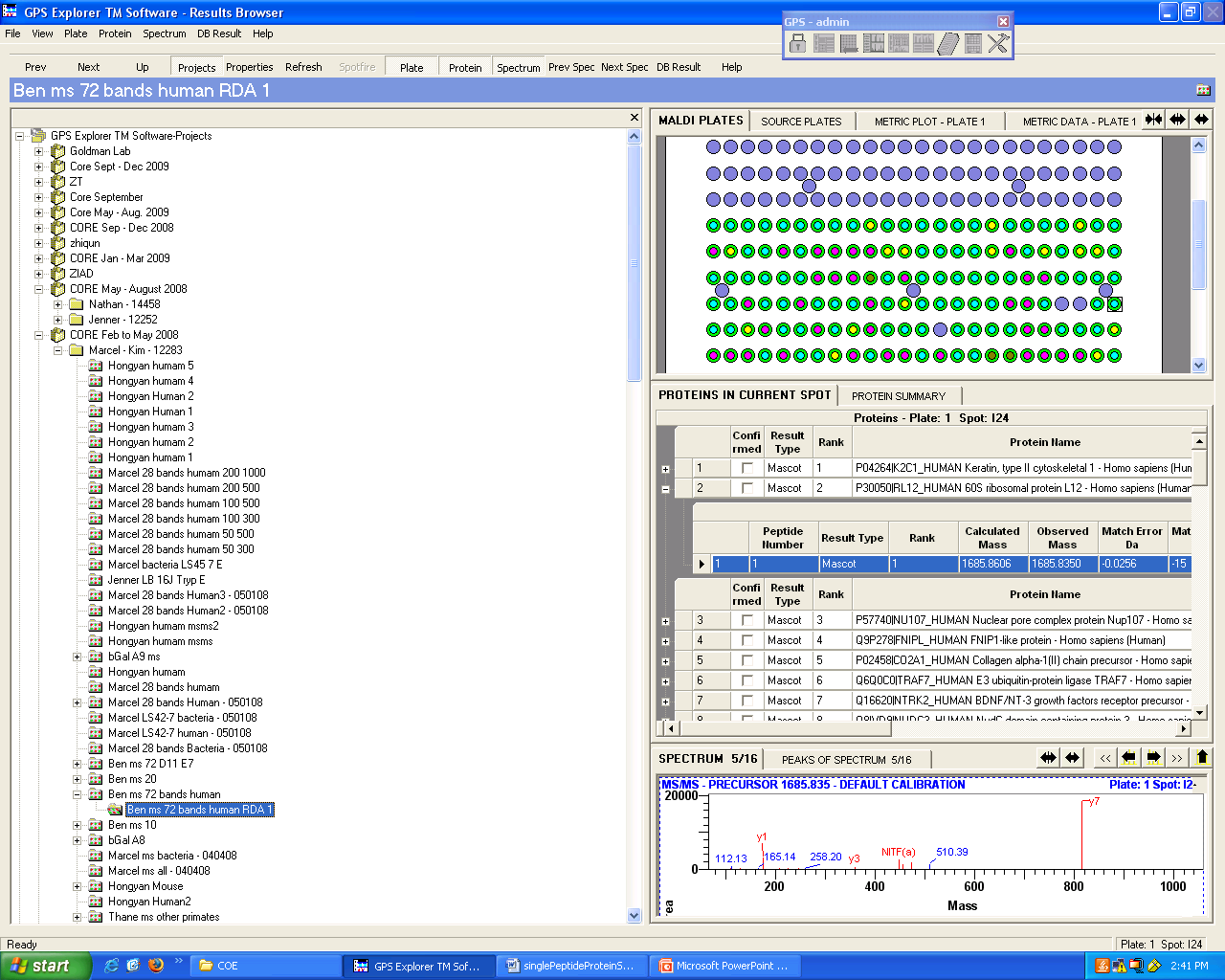 | | | | | | | |
| Y9 | P30050 | RPL12 | 28 | 96 | HSGNITFDEIVNIAR | 1685.817 | C |
| 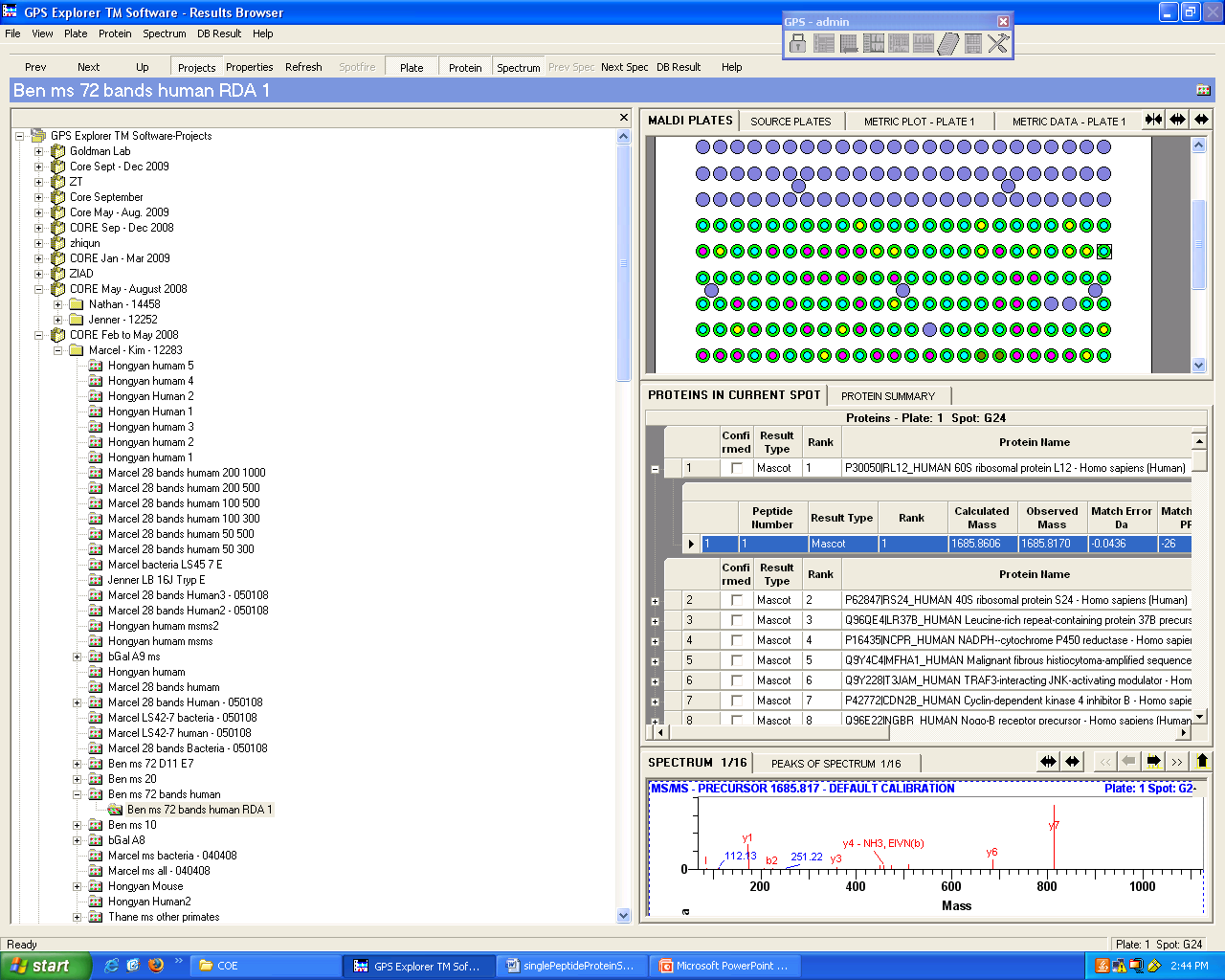 | | | | | | | |
| Y10 | P38646 | HSPA9 | 27 | 95 | STNGDTFLGGEDFDQALLR | 2055.9094 | B |
| 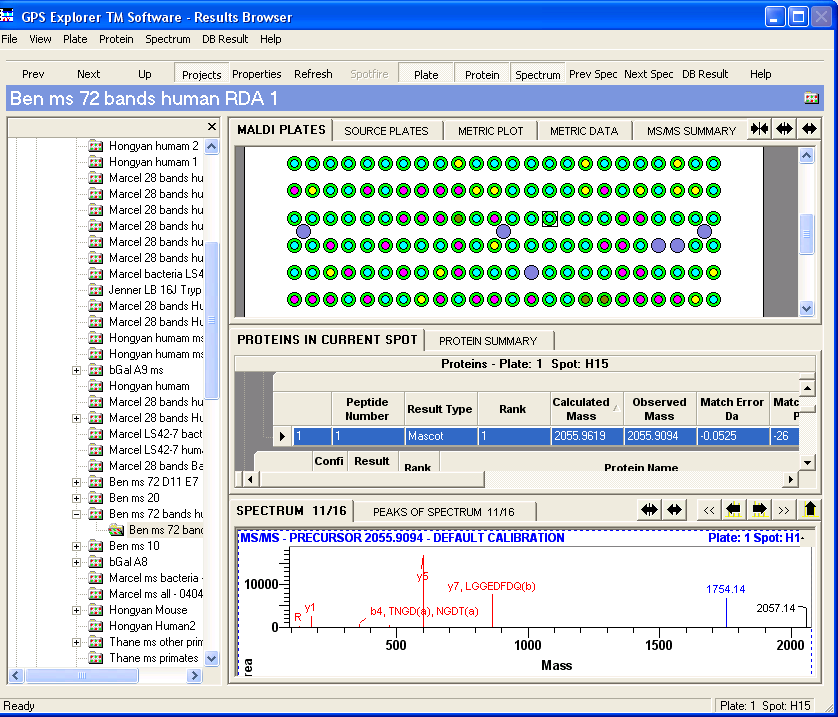 | | | | | | | |
| Y11 | P46783 | RPS10 | 39 | 100 | DYLHLPPEIVPATLR | 1733.9562 | C |
| 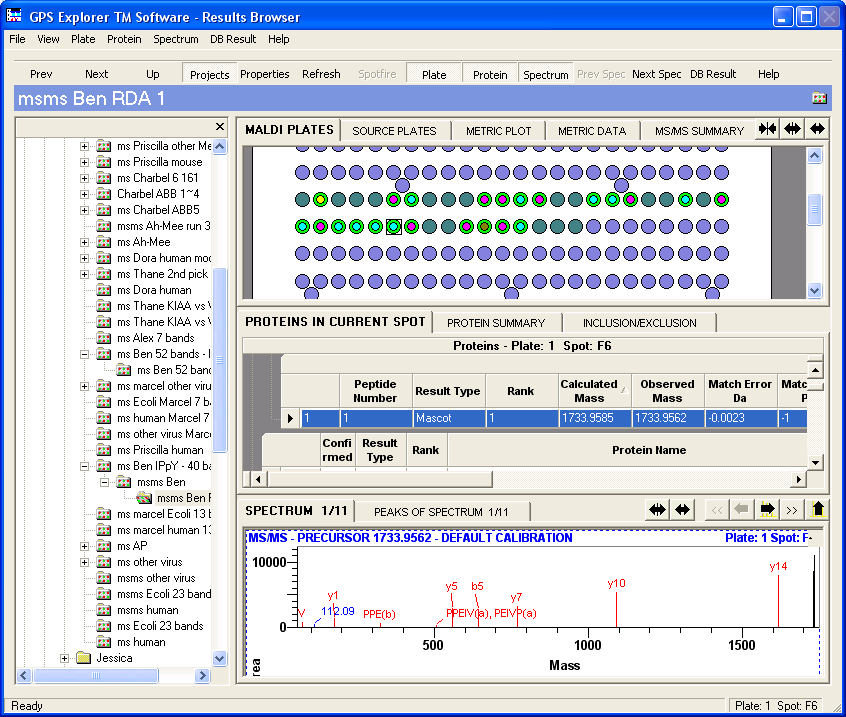 | | | | | | | |
| Y12 | P52740 | ZNF132 | 30 | 98 | SSKVHLSENPFTCR | 1661.7186 | D |
| 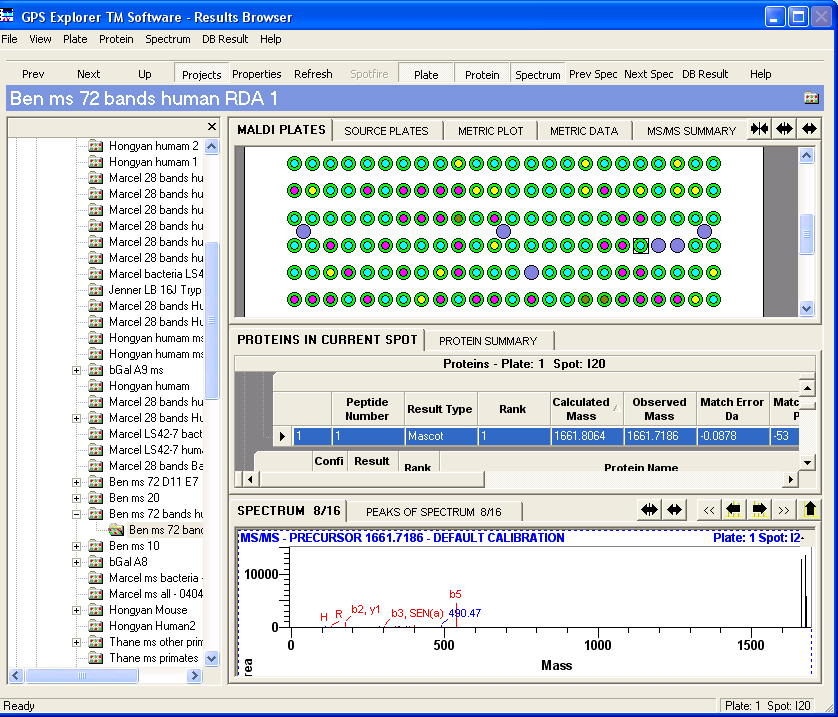 | | | | | | | |
| Y13 | P53618 | COPB1 | 45 | 100 | VASTENGIIFGNIVYDVSGAASDR | 2455.2808 | C |
| 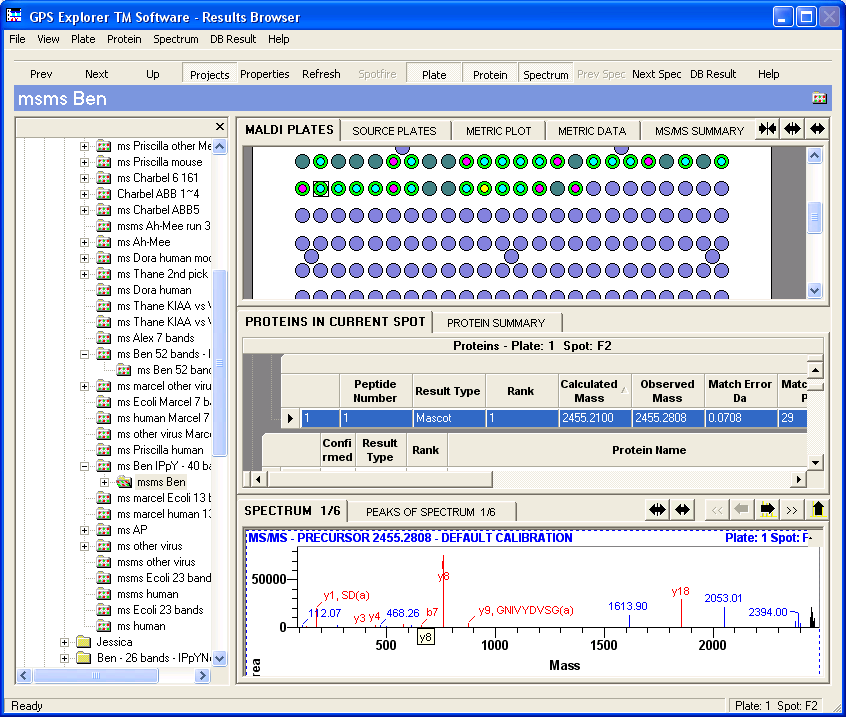 | | | | | | | |
| Y14 | P53621 | COPA | 37 | 100 | QLFLQTYAR | 1139.6401 | C |
| 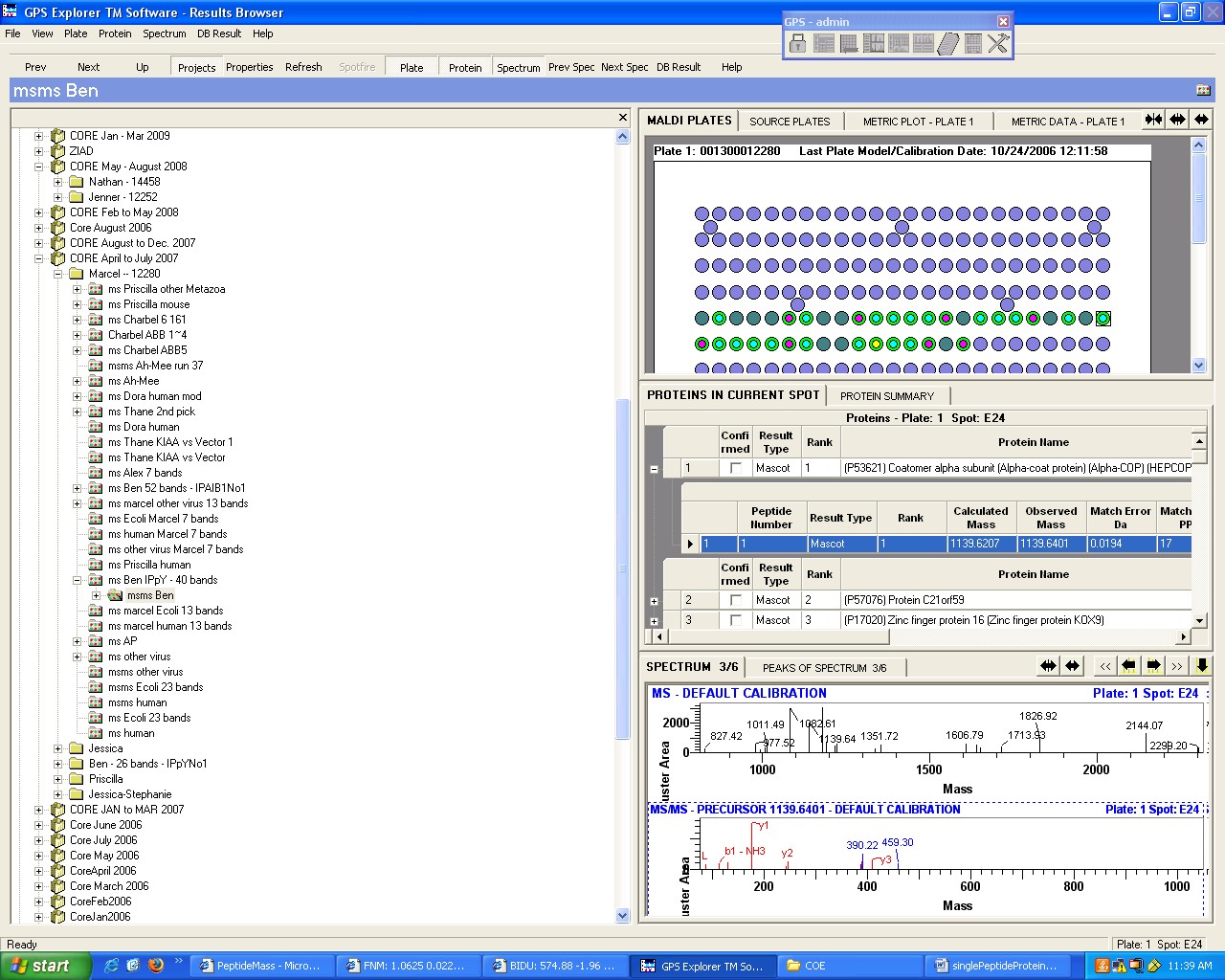 | | | | | | | |
| Y15 | P55060 | CSE1L | 61 | 100 | AADEEAFEDNSEEYIRR | 2043.8961 | D |
| 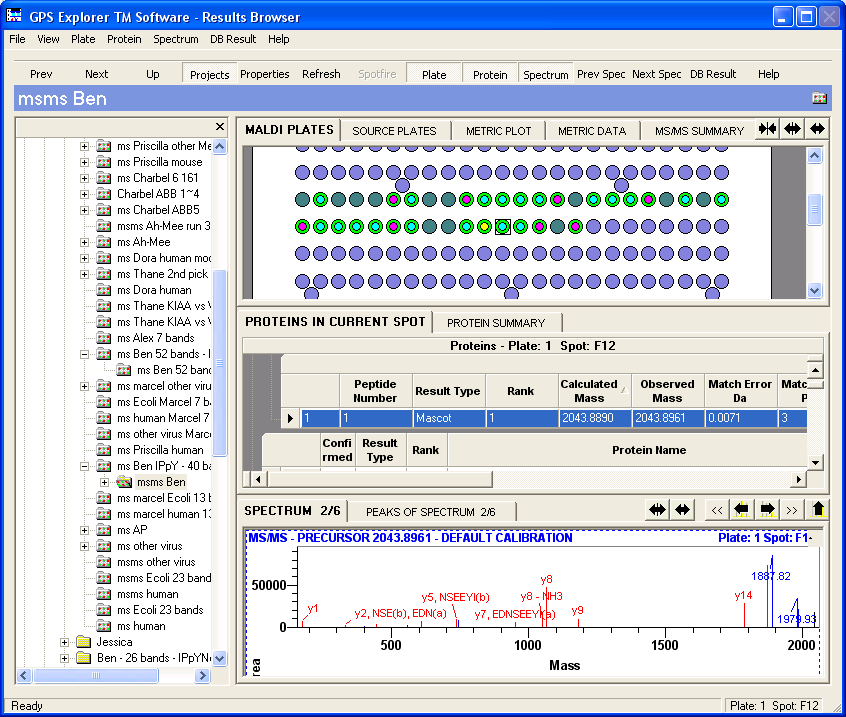 | | | | | | | |
| Y16 | P58107 | EPPK1 | 37 | 100 | AVPVWDVLASGYVSR | 1618.8922 | C |
| 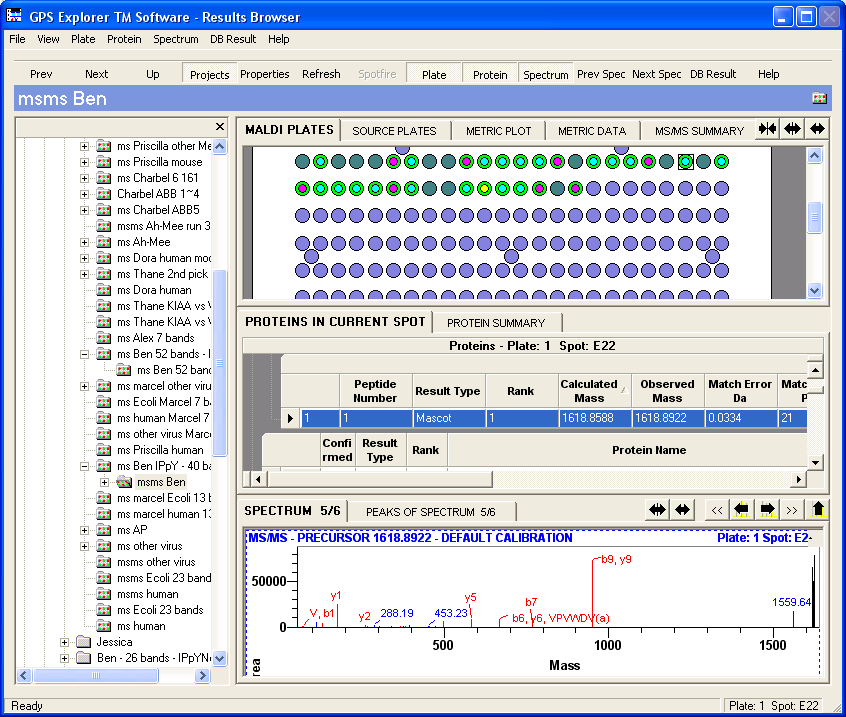 | | | | | | | |
| Y17 | P62158 | CALM1 | 83 | 100 | VFDKDGNGYISAAELR | 1754.854 | A |
| 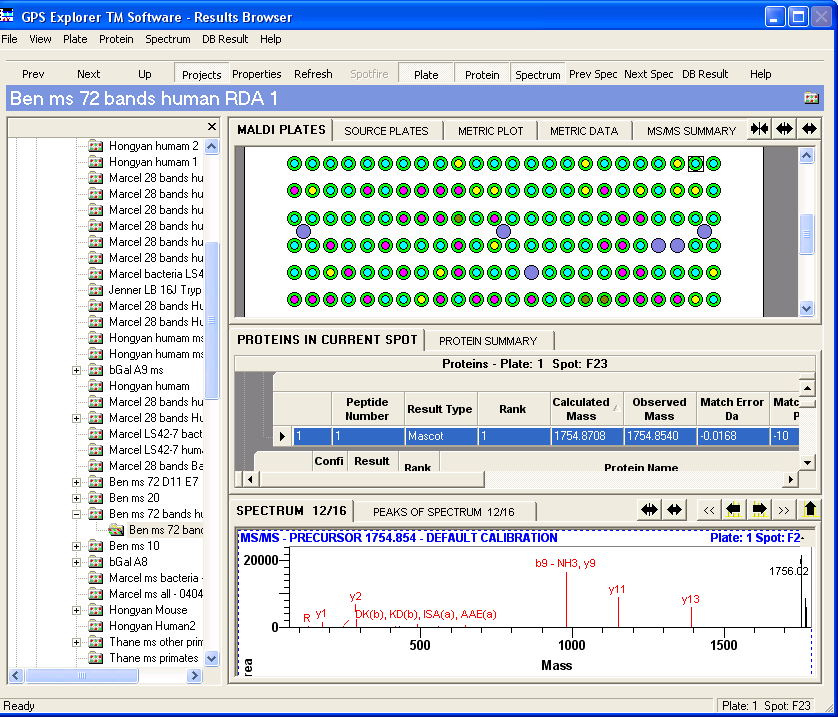 | | | | | | | |
| Y18 | P62847 | RPS24 | 29 | 98 | TTPDVIFVFGFR | 1398.7279 | B |
| 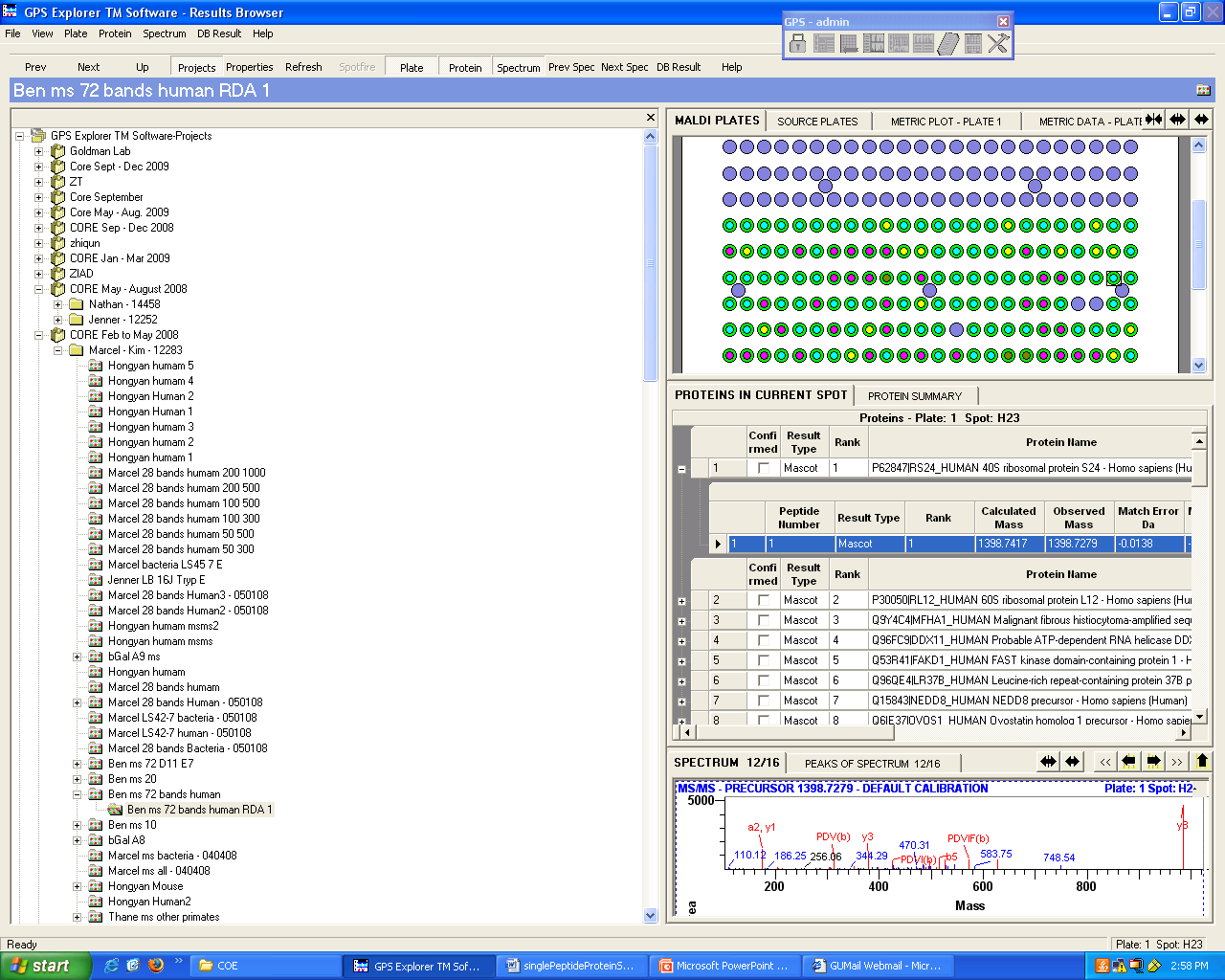 | | | | | | | |
| Y19 | P62847 | RPS24 | 27 | 94 | TTPDVIFVFGFR | 1398.6971 | C |
| 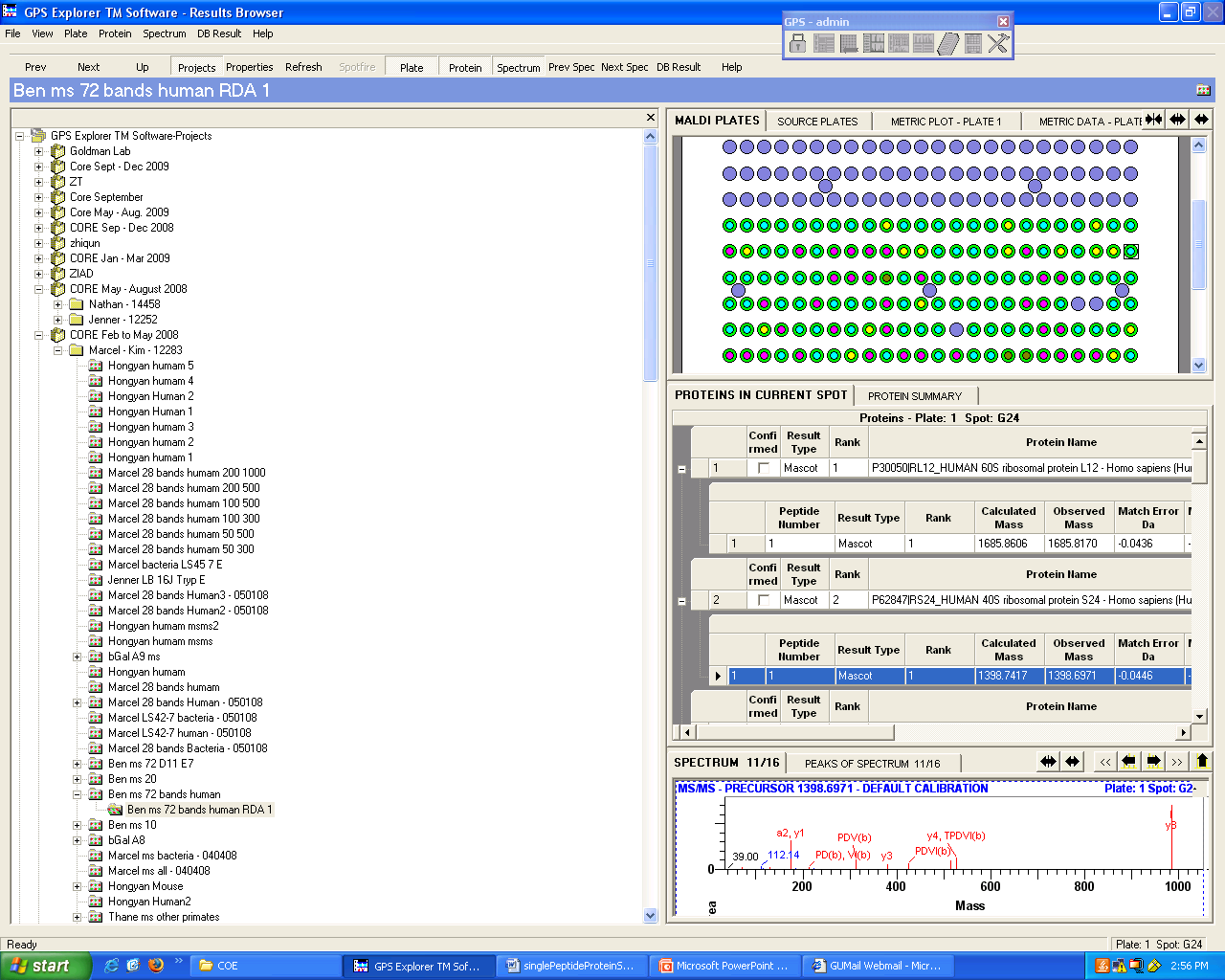 | | | | | | | |
| Y20 | P62847 | RPS24 | 31 | 98 | TTPDVIFVFGFR | 1398.6919 | A |
| 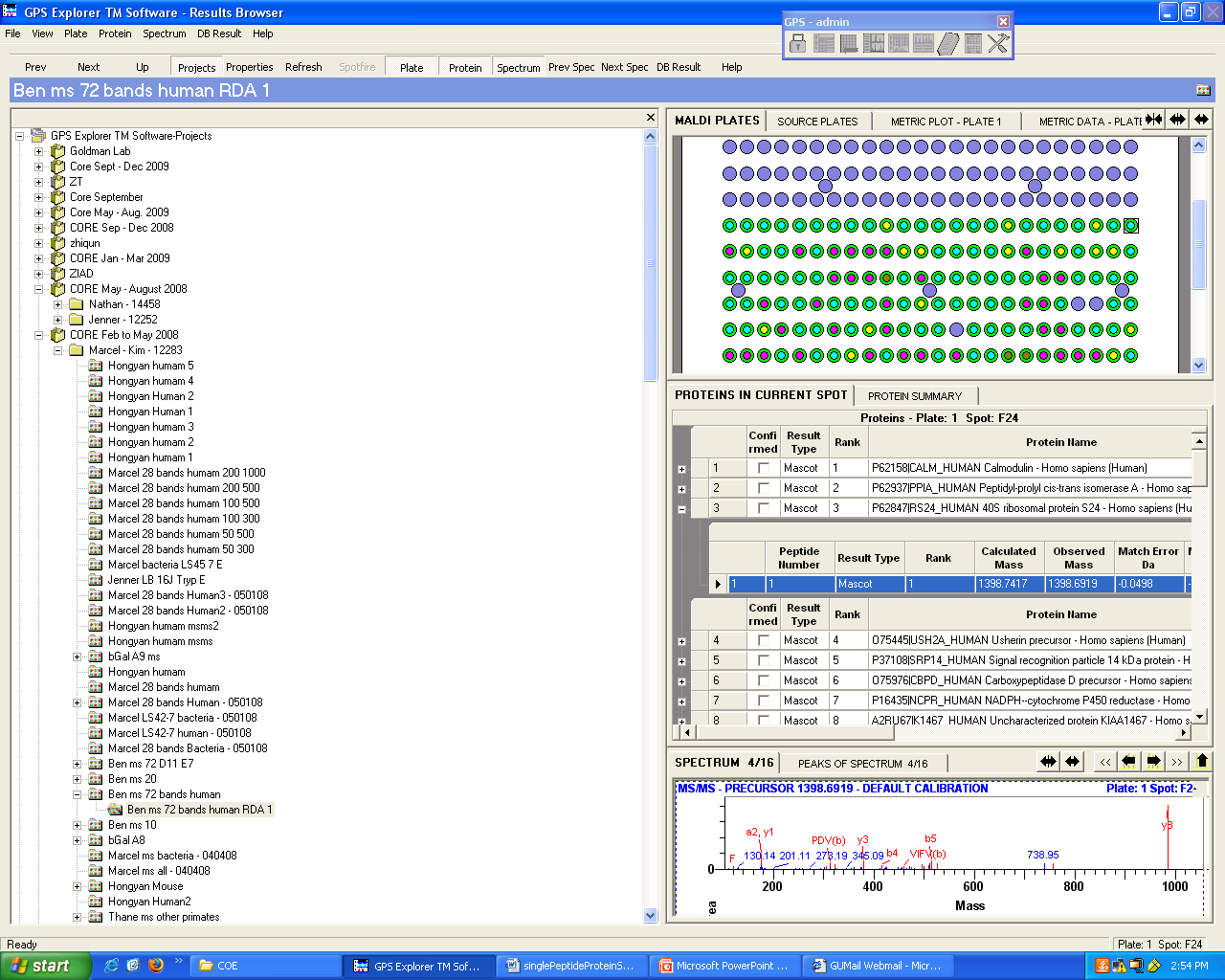 | | | | | | | |
| Y21 | P62937 | PPIA | 41 | 100 | VNPTVFFDIAVDGEPLGR | 1945.9984 | A |
| 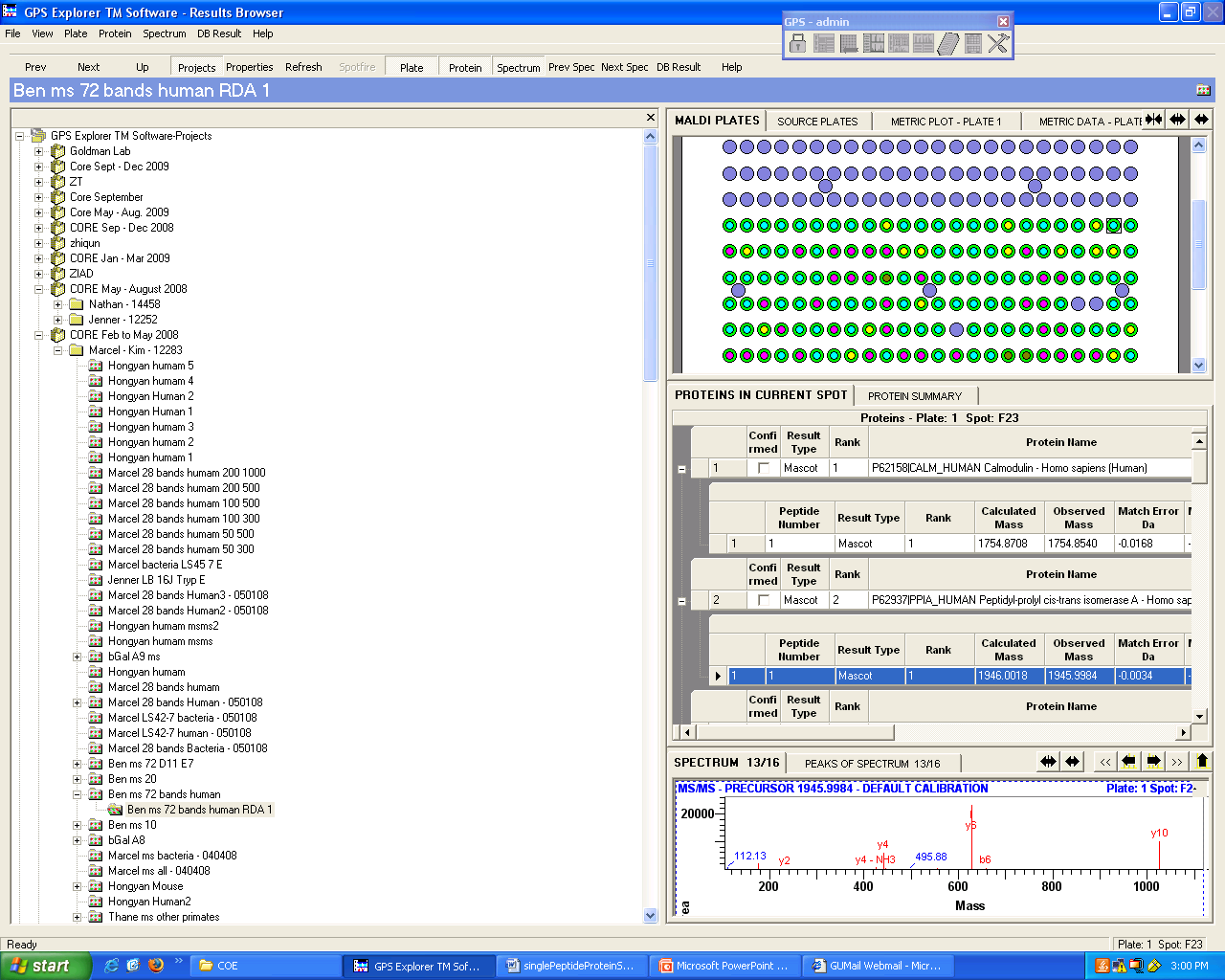 | | | | | | | |
| Y22 | P62937 | PPIA | 32 | 98 | VNPTVFFDIAVDGEPLGR | 1945.9657 | C |
| 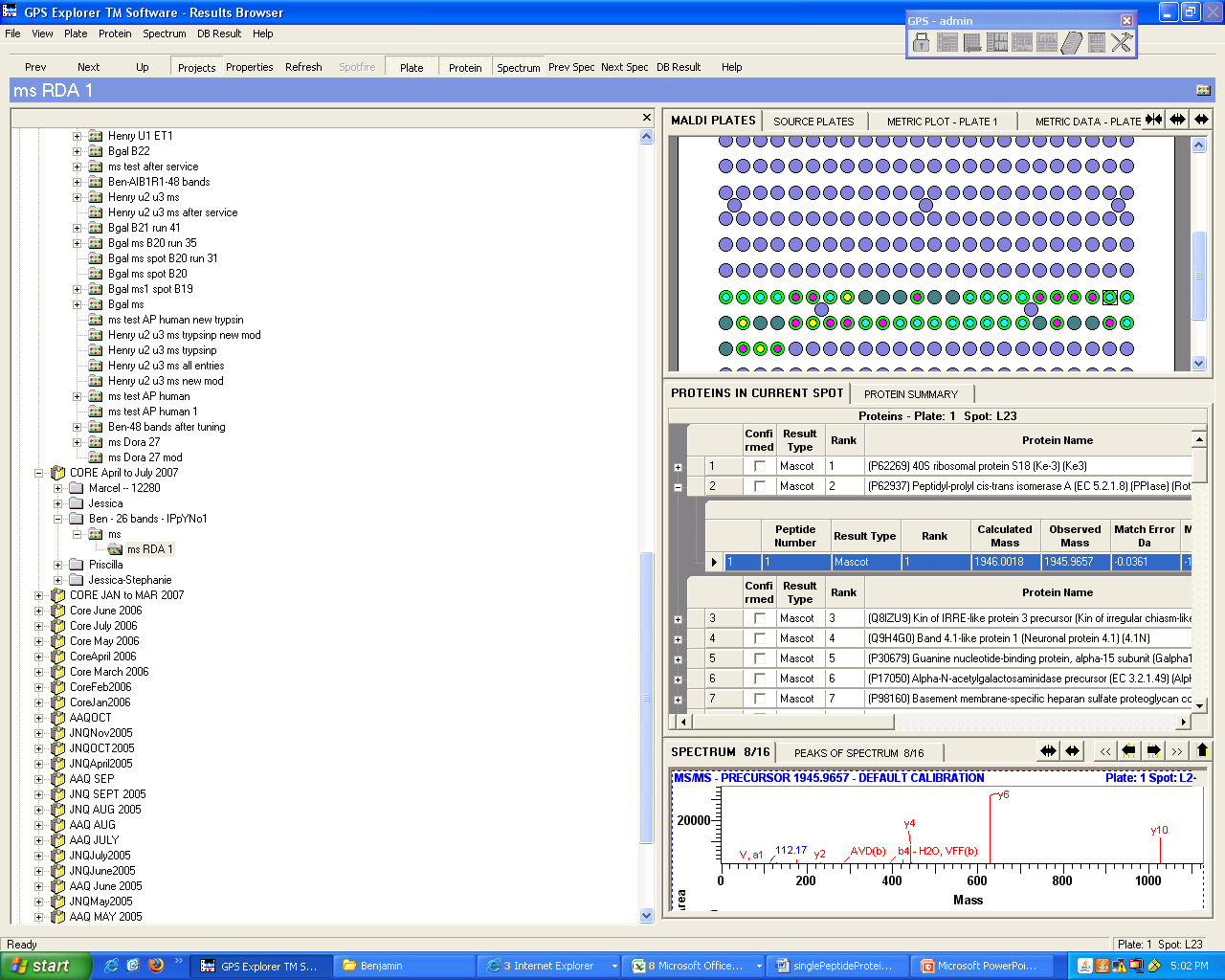 | | | | | | | |
| Y23 | Q05397 | PTK2 | 40 | 100 | TLLATVDETIPLLPASTHR | 2048.0291 | D |
| 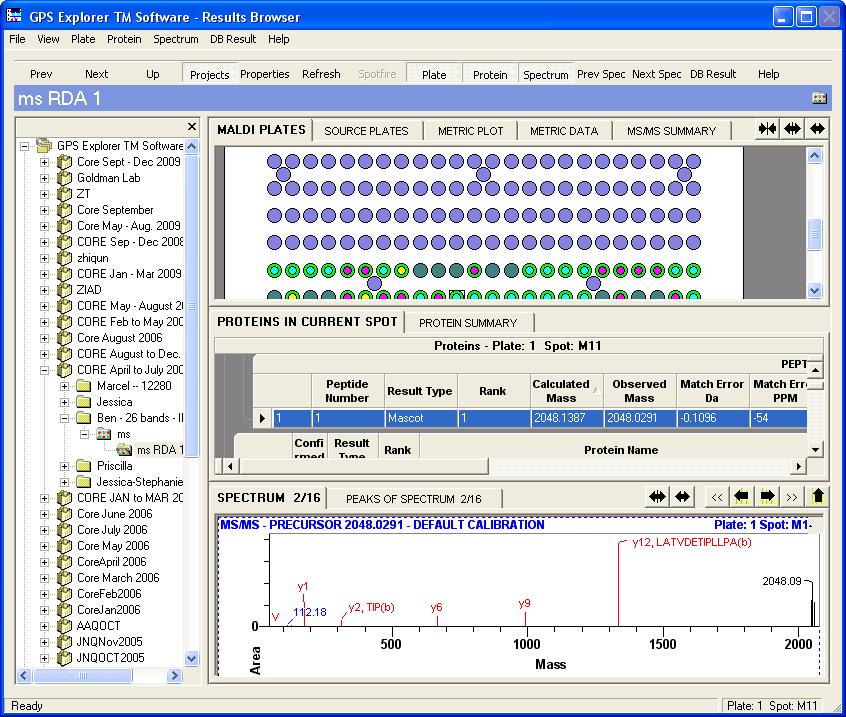 | | | | | | | |
| Y24 | Q14257 | RCN2 | 27 | 96 | LSEEEILENPDLFLTSEATDYGR | 2641.2957 | B |
| 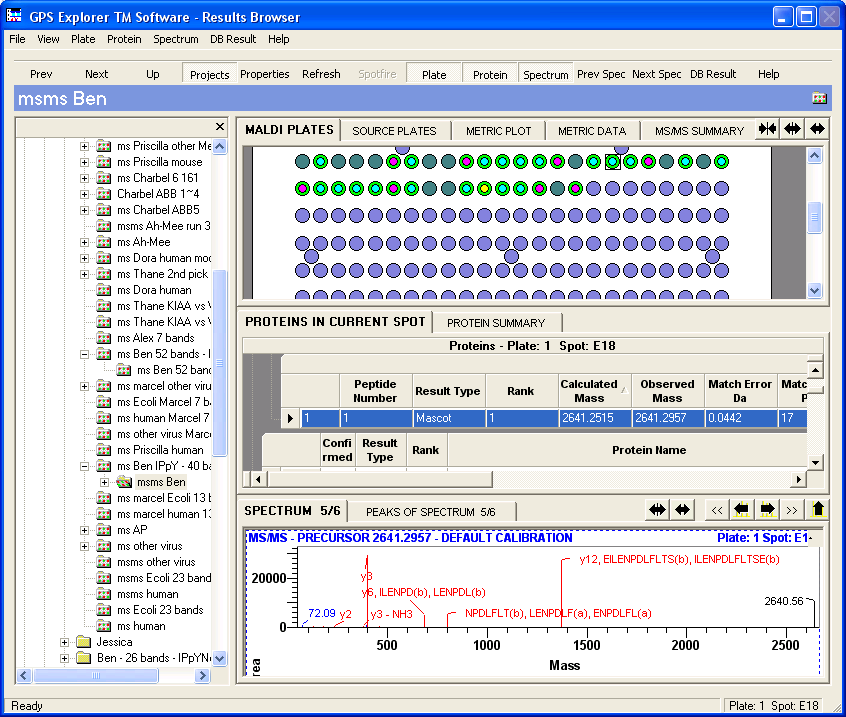 | | | | | | | |
| Y25 | Q92616 | GCN1L1 | 42 | 100 | DAVLYFSESLVPTAR | 1667.8971 | C |
| 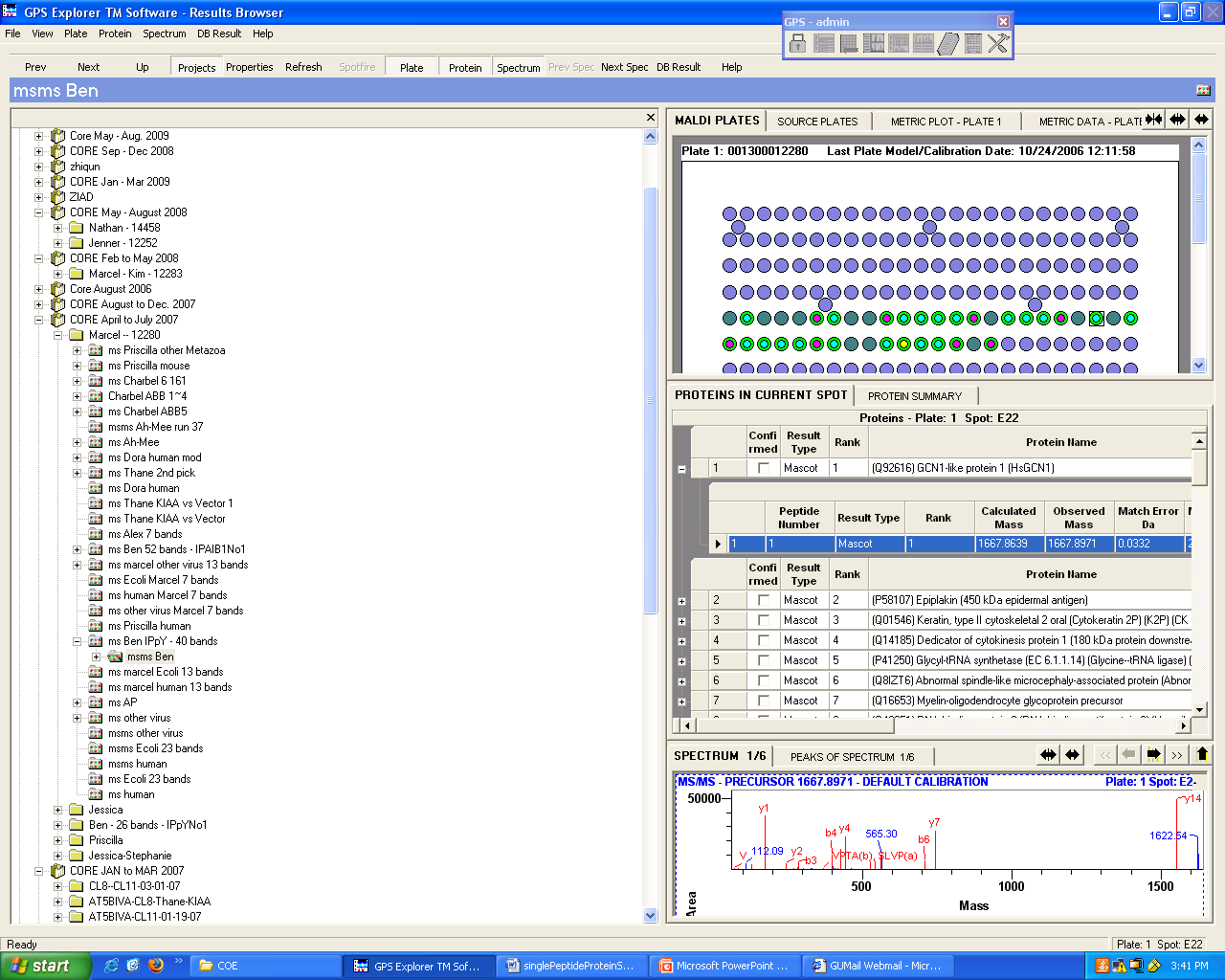 | | | | | | | |
| Y26 | Q96NK8 | NEUROD6 | 31 | 98 | NRMHGLNDALDNLR | 1638.8113 | D |
| 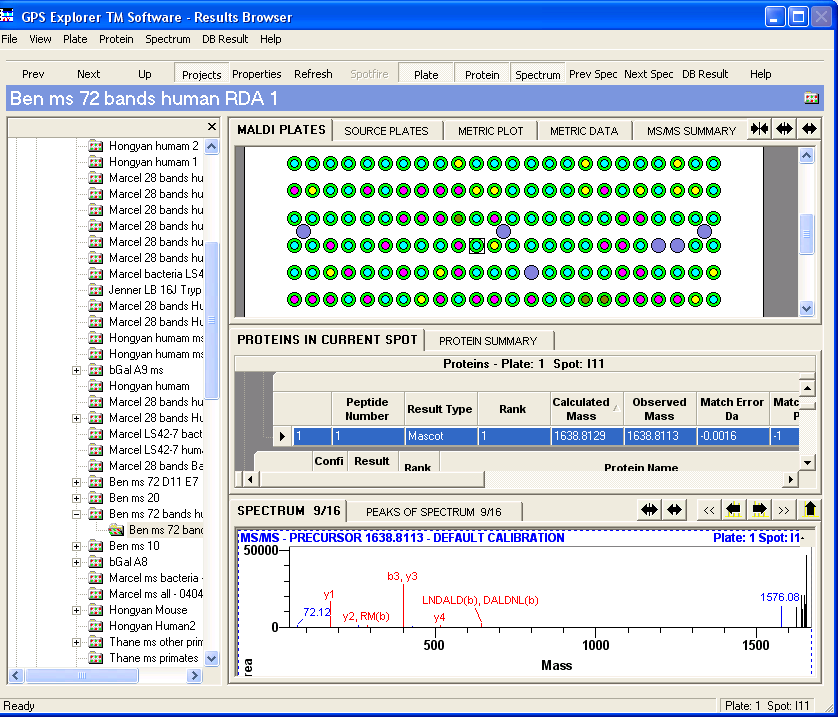 | | | | | | | |
| Y27 | Q9BQE3 | TUBA1C | 38 | 100 | AVFVDLEPTVIDEVR | 1701.9205 | C |
| 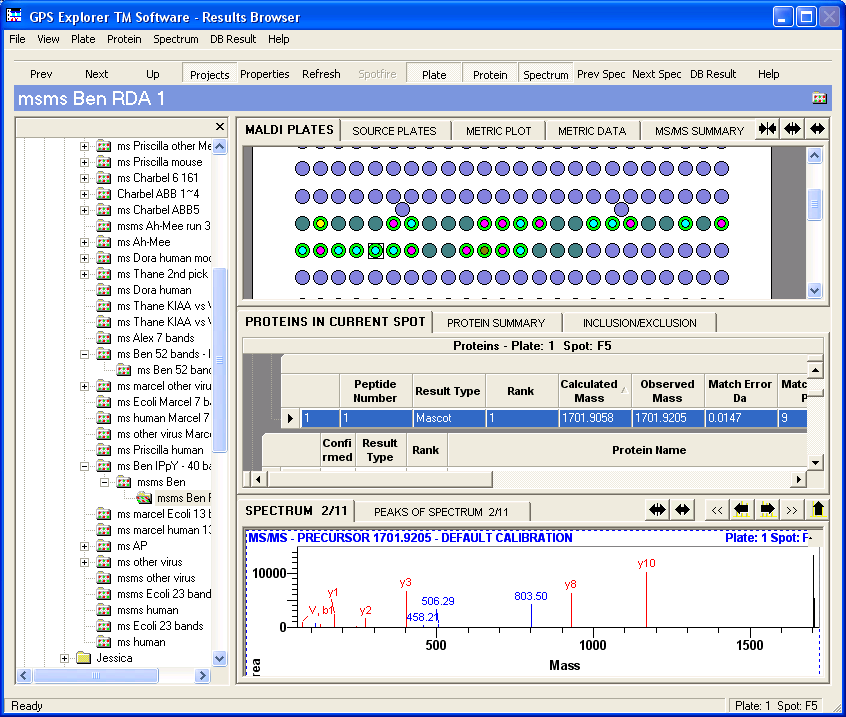 | | | | | | | |
| Y28 | Q9NSV4 | DIAPH3 | 29 | 98 | MGSADERLVTCLESLR | 1795.7345 | C |
| 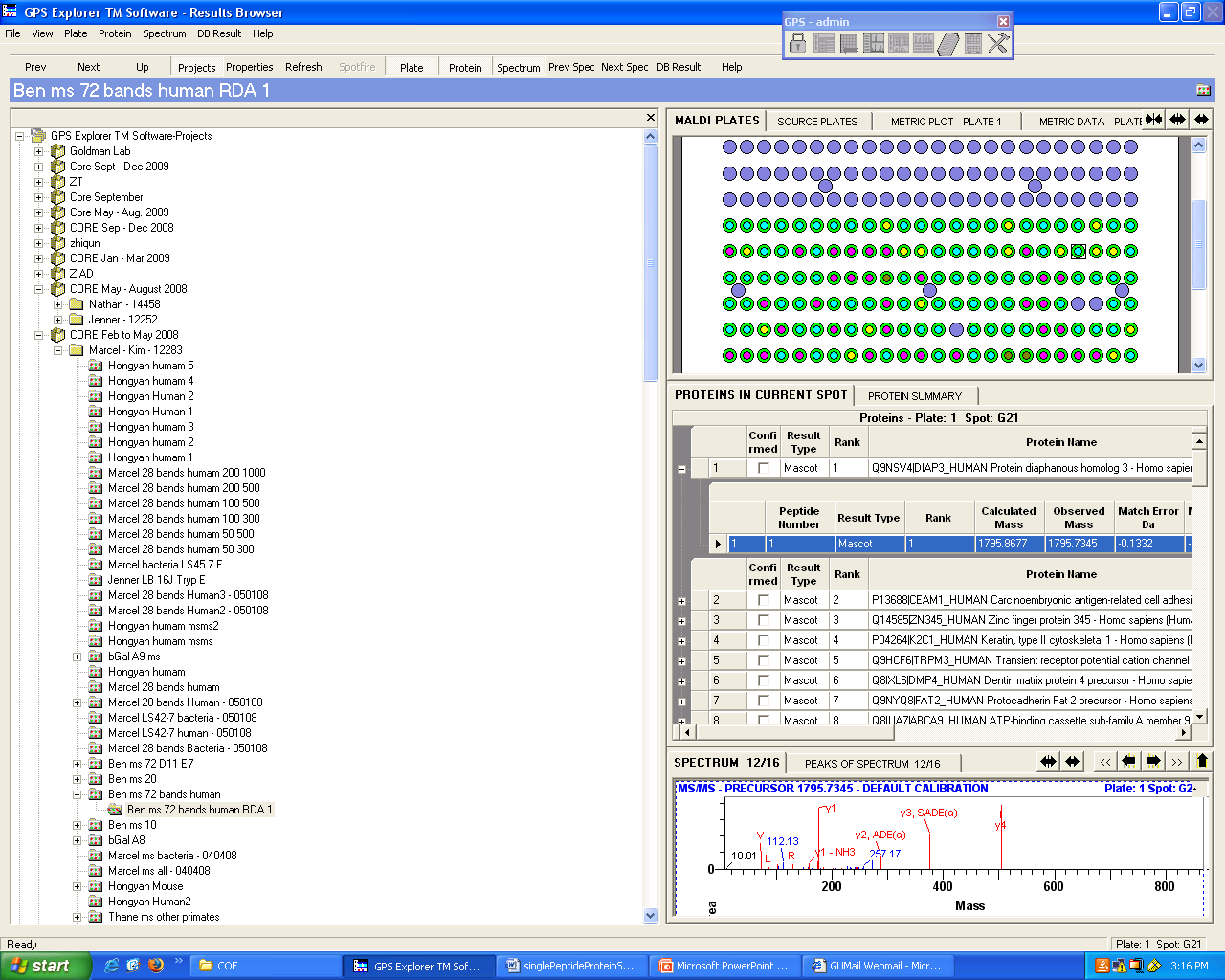 | | | | | | | |
| Y29 | Q9UL46 | PSME2 | 37 | 100 | QNLFQEAEEFLYR | 1686.8502 | B |
| 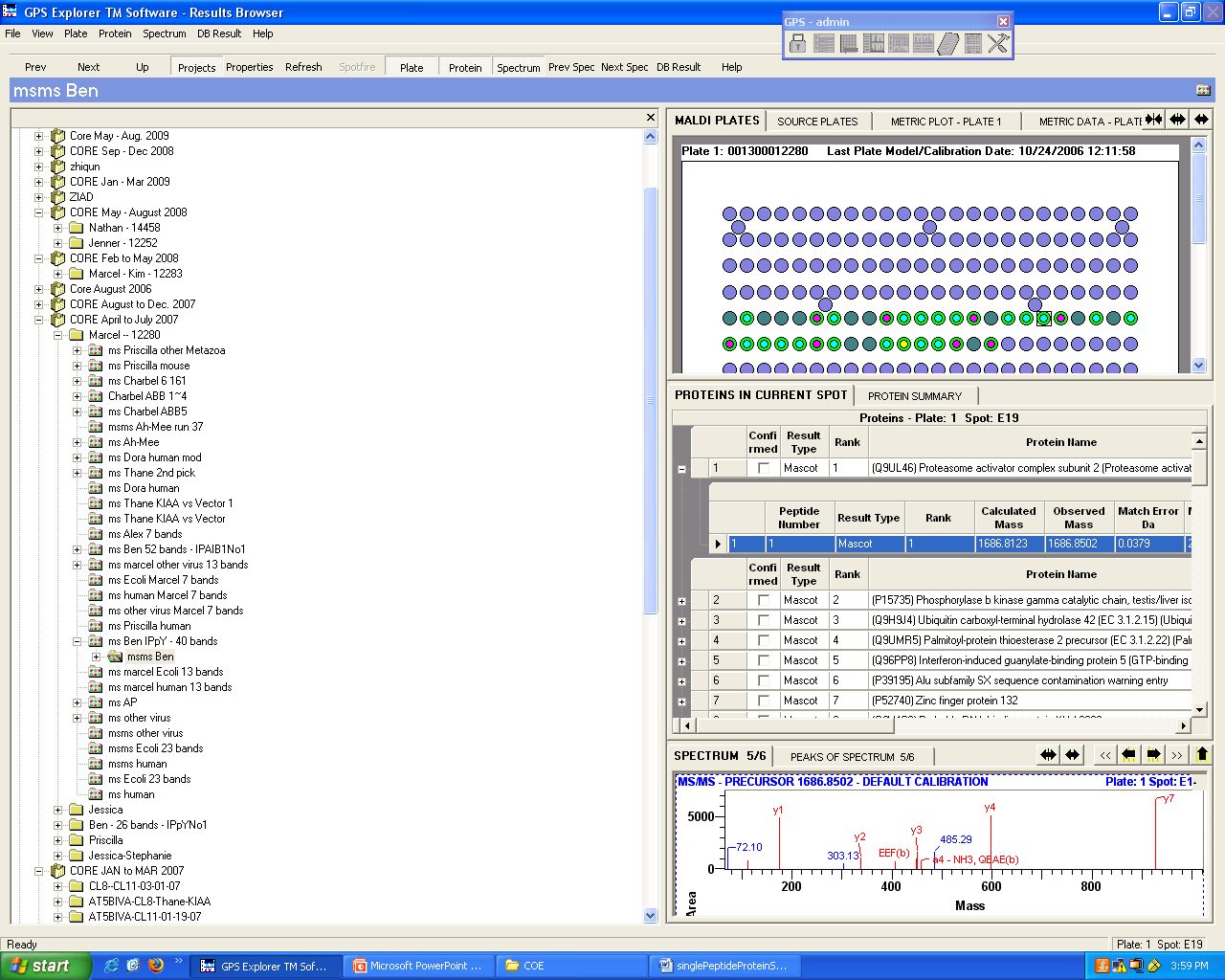 | | | | | | | |
| Y30 | Q9UL46 | PSME2 | 54 | 100 | QNLFQEAEEFLYR | 1686.8351 | C |
| 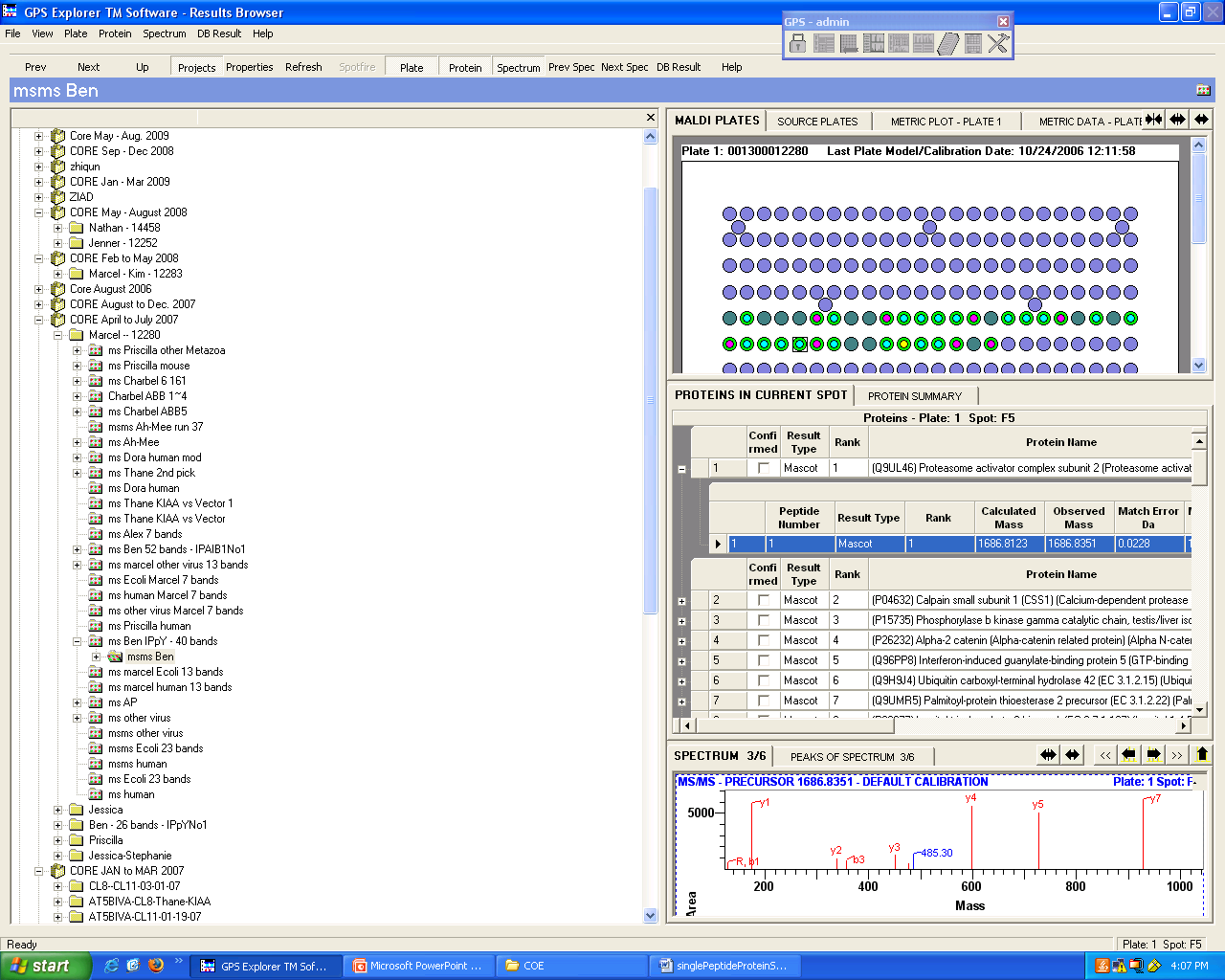 | | | | | | | |
| Y31 | Q14585 | ZNF345 | 26 | 94 | ENLTKHSIECSSFR | 1707.7882 | B |
| 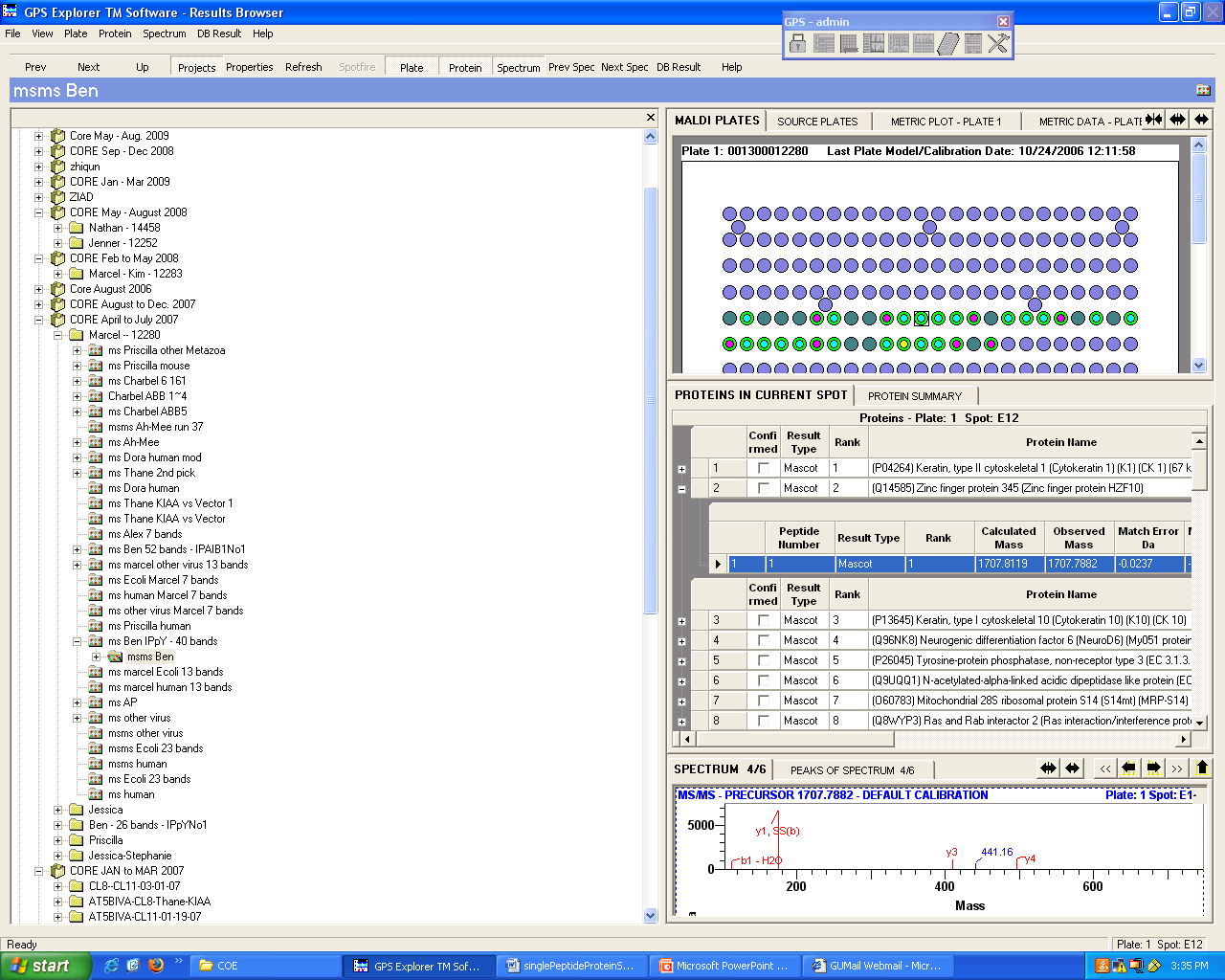 | | | | | | | |
| Y32 | Q15021 | NCAPD2 | 26 | 94 | LRDPAQQVR | 1082.5973 | D |
| 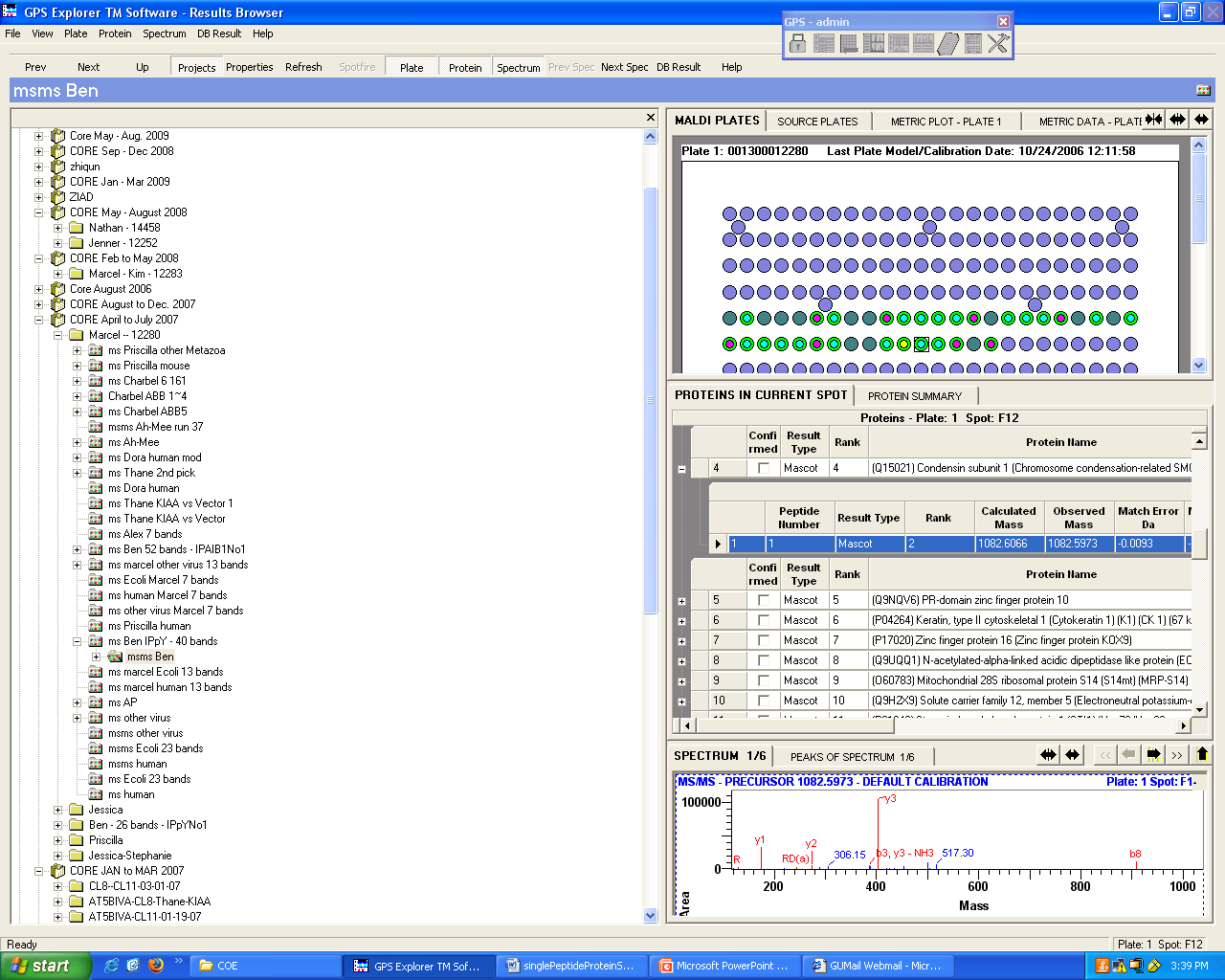 | | | | | | | |
| Y33 | O00159 | MYO1C | 25 | 93 | GEELLSPLNLEQAAYAR | 1873.892 | A |
| 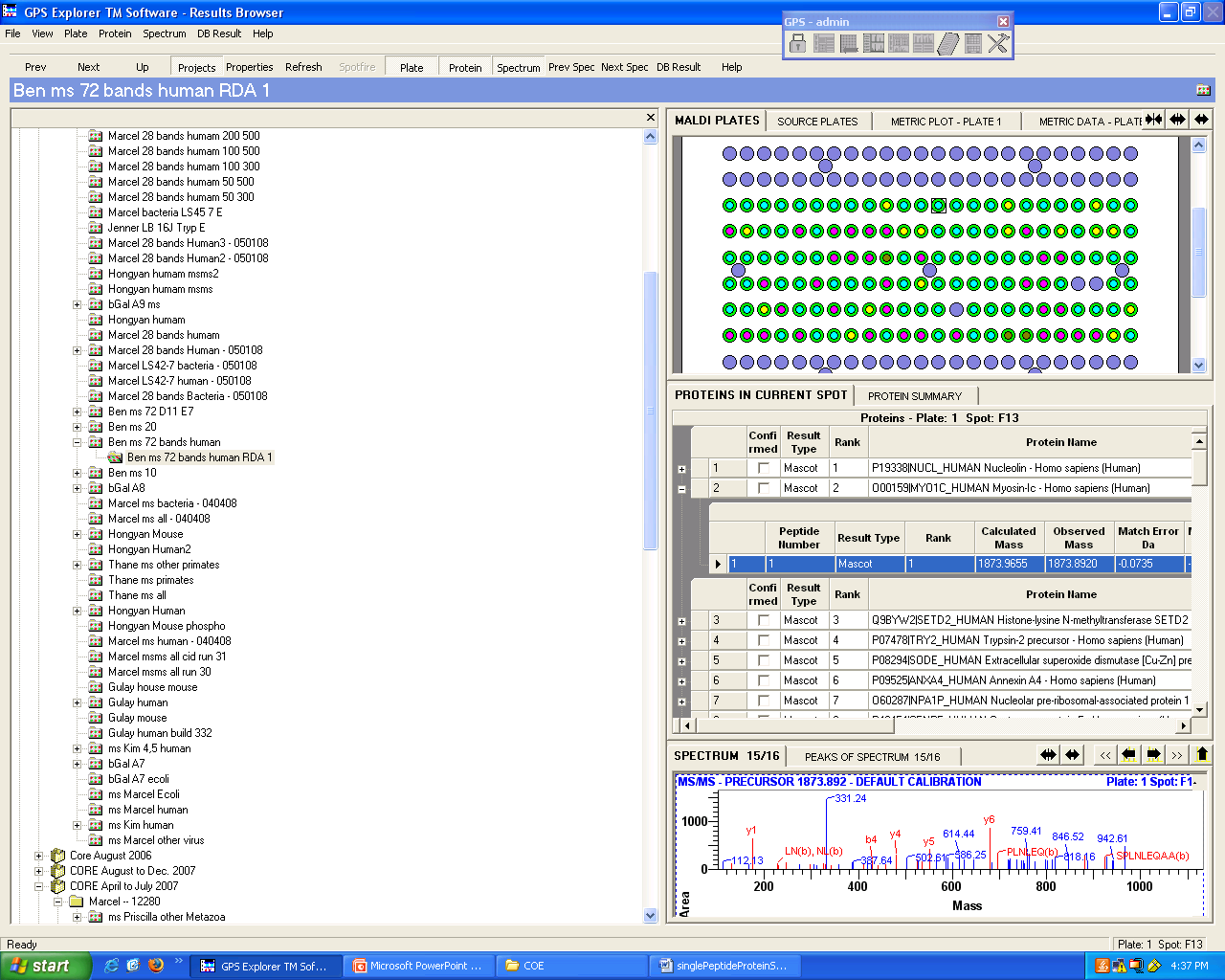 | | | | | | | |
| Y34 | O14639 | ABLIM1 | 23 | 90 | CGEPCKGEVLR | 1247.6094 | B |
| 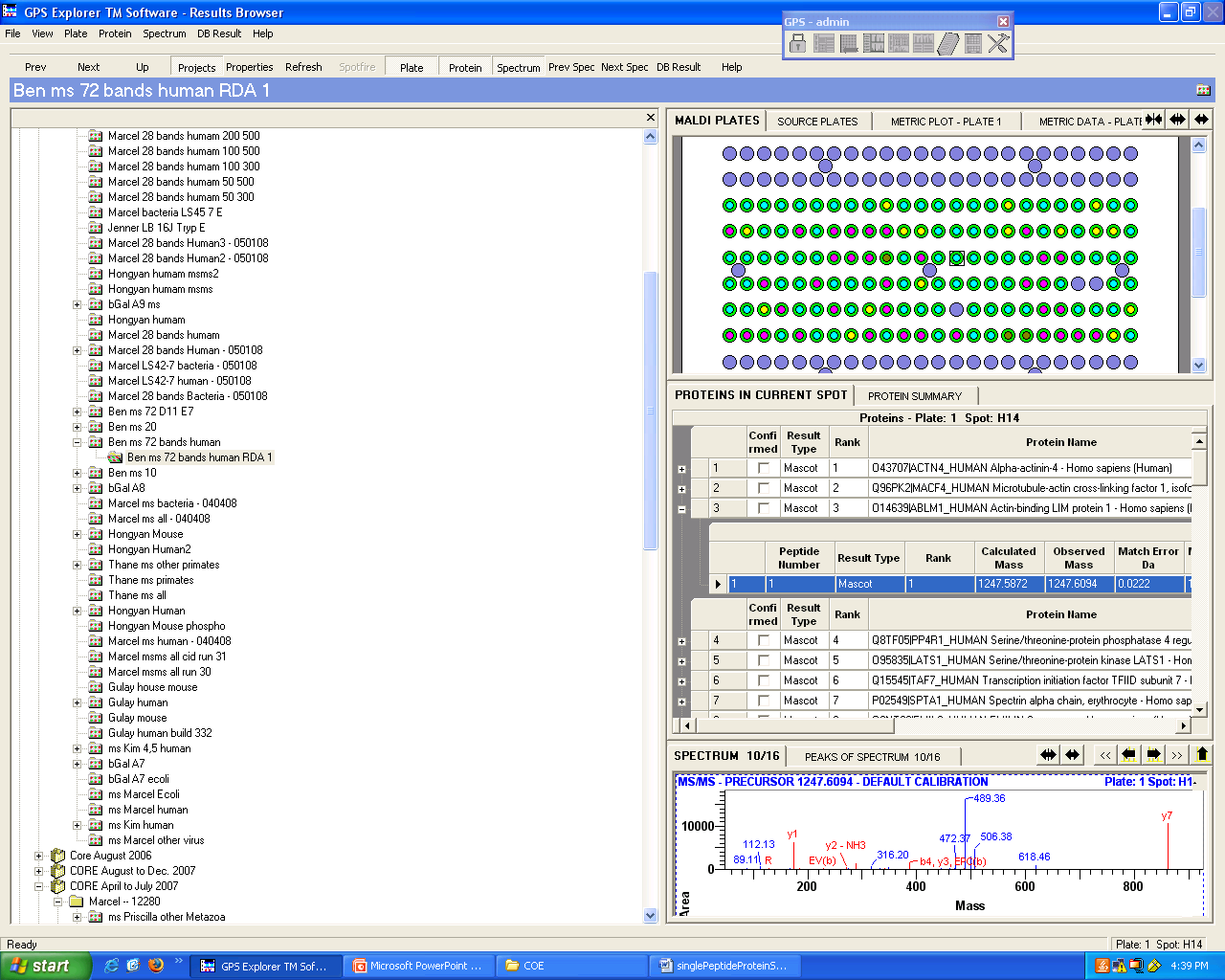 | | | | | | | |
| Y35 | Q13219 | PAPPA | 24 | 93 | DPPLQMDVASILHLNR | 1834.9351 | B |
| 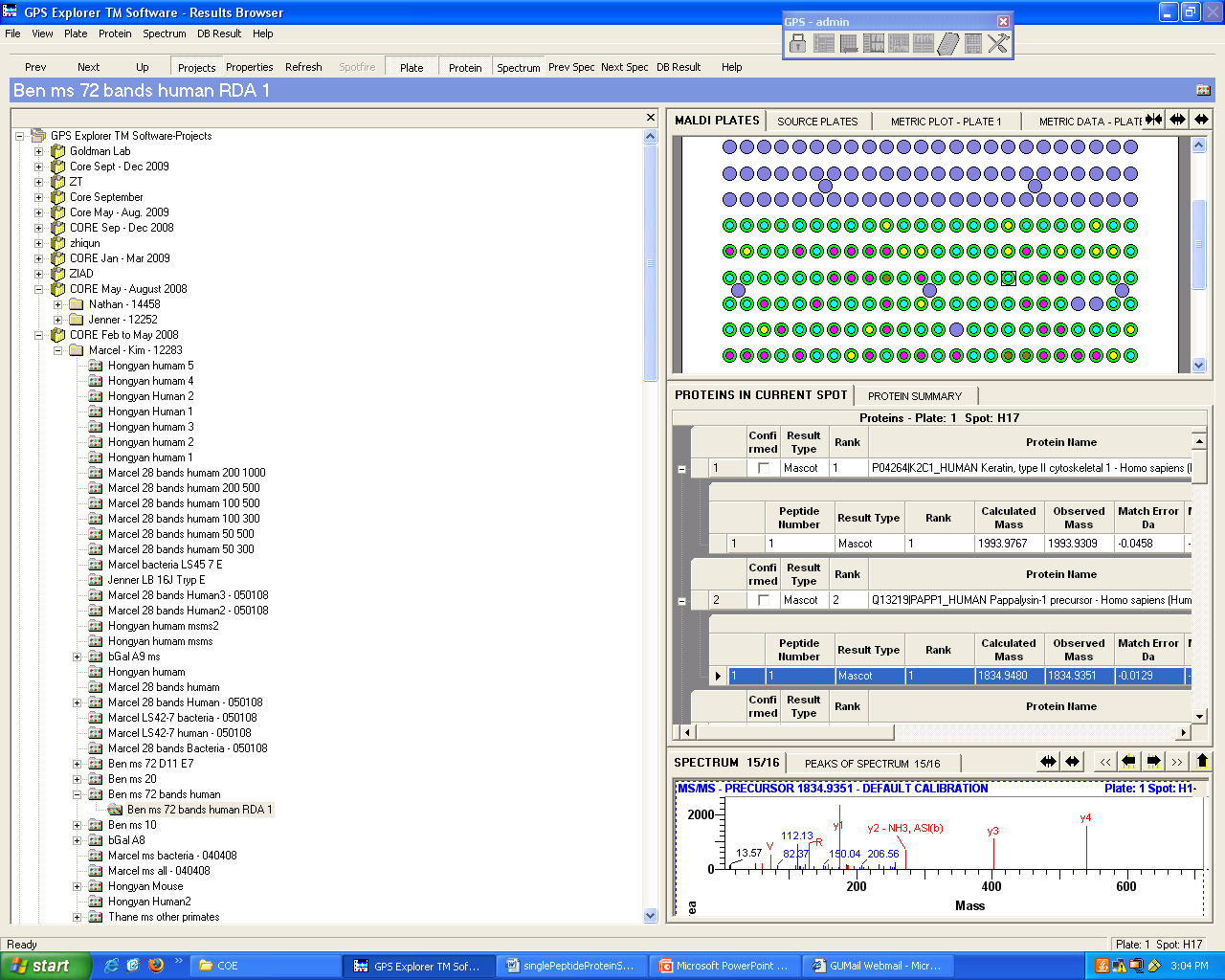 | | | | | | | |
| Y36 | Q8IZT6 | ASPM | 27 | 93 | QIRAACVIQSYWR | 1650.8562 | D |
| 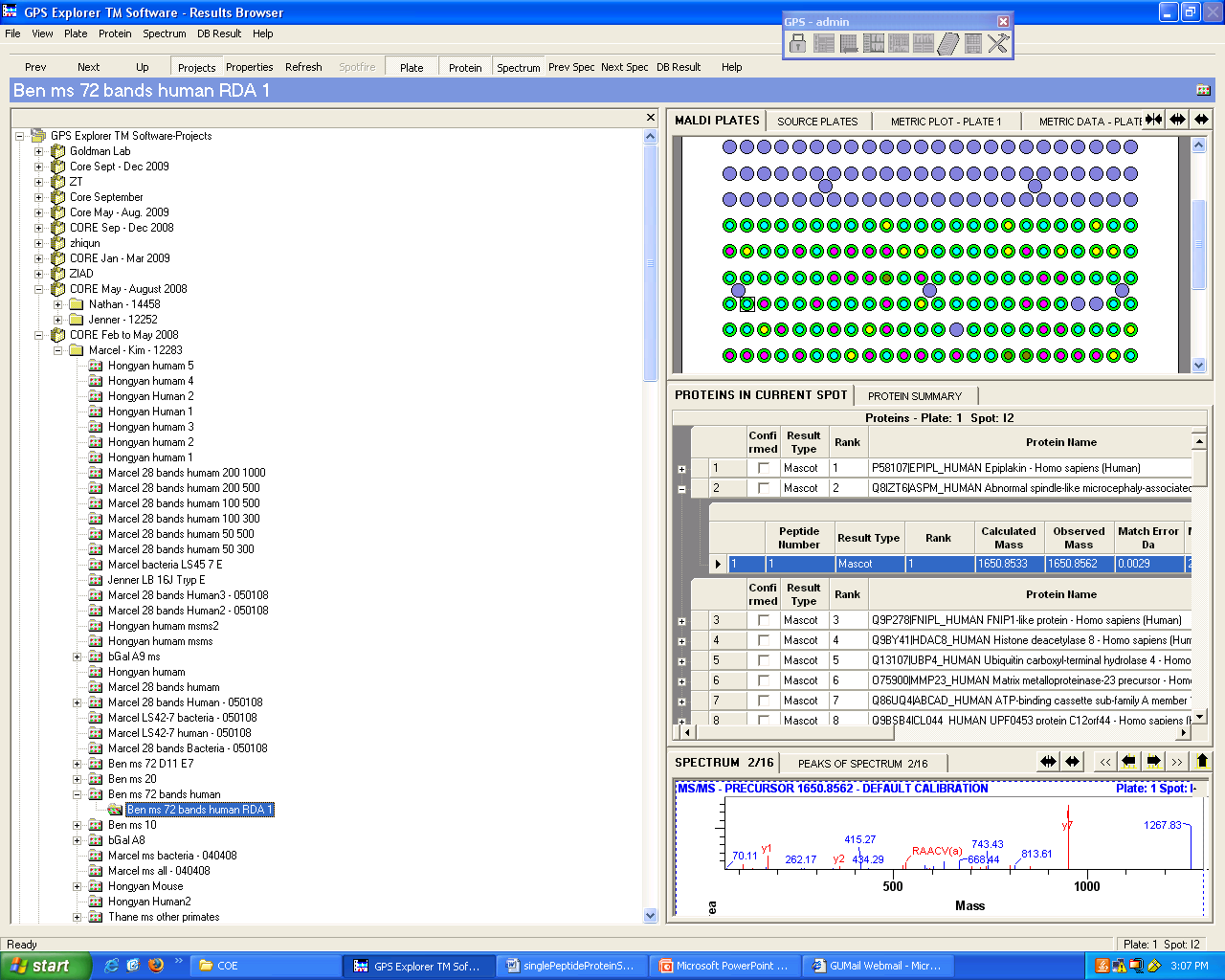 | | | | | | | |
| Y37 | Q8N163 | KIAA1967 | 25 | 91 | AAEAAPPTQEAQGETEPTEQAPDALEQAADTSR | 3380.4922 | D |
| 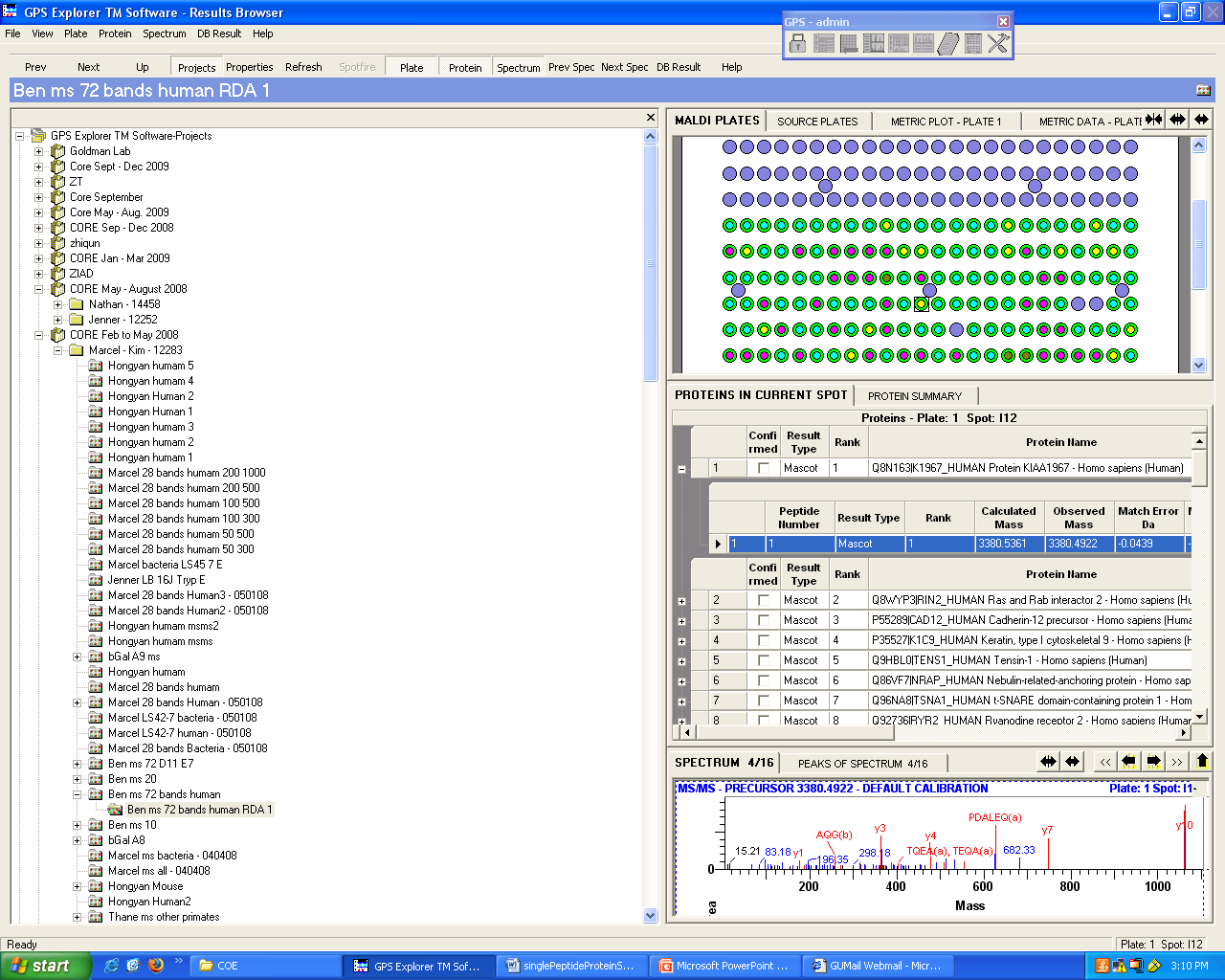 | | | | | | | |
| Y38 | Q96QE4 | LRRC37B | 25 | 91 | APHPDQVQTLHR | 1398.6971 | C |
| 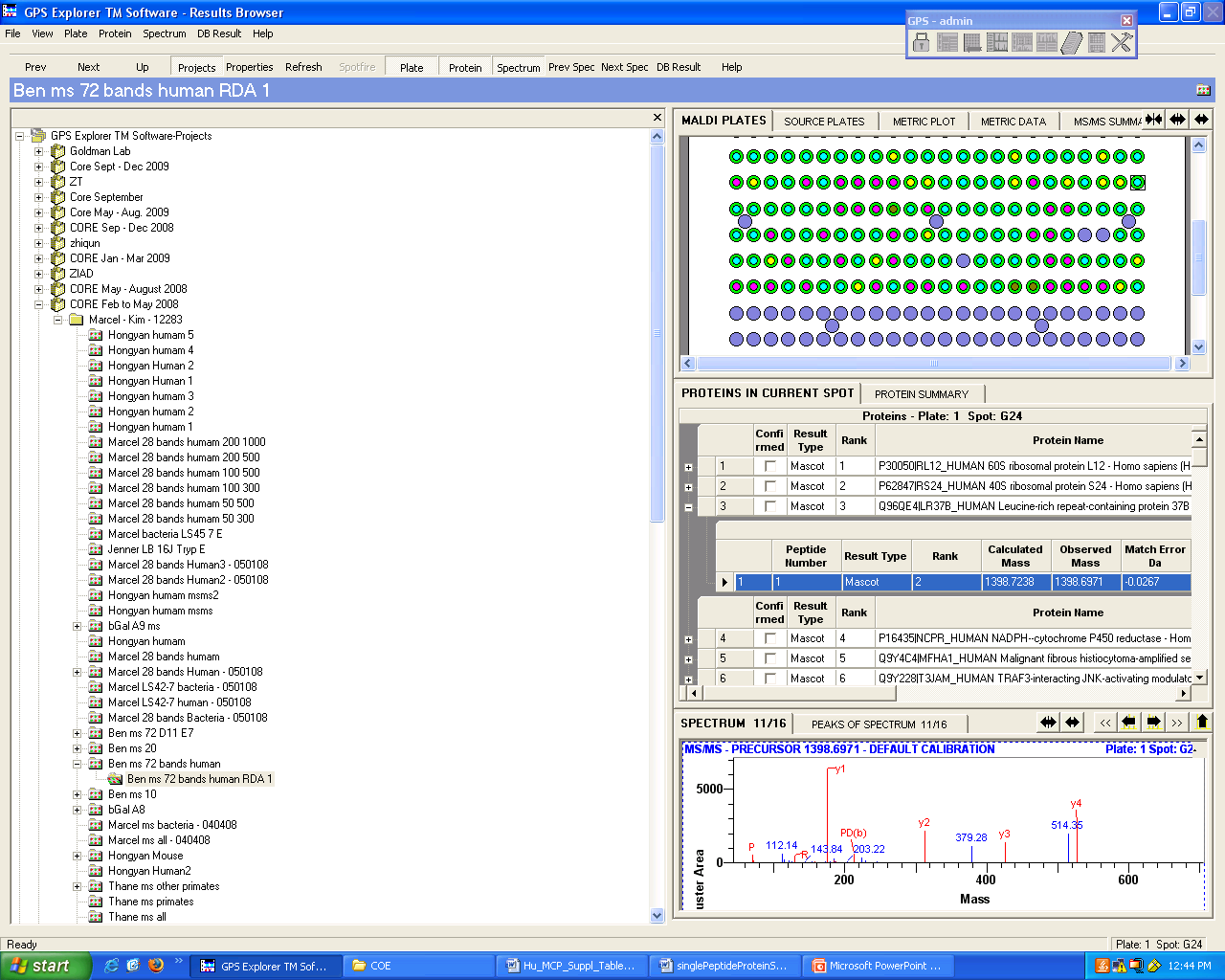 | | | | | | | |
