# Supplementary material for: Proteomic Analysis of Pathways Involved in Estrogen-Induced Growth and Apoptosis of Breast Cancer Cells
Source: PLoS One. 2011 Jun 27;6(6):e20410. doi: 10.1371/journal.pone.0020410 (PMC3124472; doi:10.1371/journal.pone.0020410)
Supplement: Table S8 — List of acronyms used. (DOC) [file pone.0020410.s016.doc]

| **Short Name** | **Long Name** |
| --- | --- |
| ABL1 | Abelson murine leukemia viral oncogene homolog 1 |
| ACTB | Beta-actin |
| ACTG1 | Gamma-actin |
| AIB1 | Amplified in breast cancer 1 = NCOA3 |
| AKT | v-akt murine thymoma viral oncogene homolog |
| ASPM | Abnormal spindle-like microcephaly-associated protein |
| BAD | Bcl2 antagonist of cell death |
| Bcl-XL | B-cell lymphoma-extra large protein |
| BCL2 | B-cell lymphoma 2-encoded protein |
| BCL3 | B-cell lymphoma 3-encoded protein |
| bHLH | Basic helix-loop-helix protein |
| BRCA1 | Breast cancer type 1 susceptibility protein |
| CALM1 | Breast cancer type 2 susceptibility protein |
| CDH22 | Cadherin-22 |
| CDC2 | Cell division control protein 2 homolog |
| CDK1, 2, 4 | Cyclin-dependent kinase 1 or 2 or 4 |
| CDKN1B | cyclin-dependent kinase inhibitor 1B (p27, Kip1) |
| CHUK | Conserved helix-loop-helix ubiquitous kinase (I kappa-B kinase alpha) |
| CK1 | Casein kinase I isoform alpha |
| CK1δ | Casein kinase I isoform delta |
| CREBBP | cAMP response element-binding protein (CREB) binding protein or CBP |
| DBC1 | Deleted in breast cancer 1 |
| DSH | Segment polarity protein dishevelled homolog |
| EGF (R) | Epidermal growth factor (receptor) |
| ERα | Estrogen receptor alpha |
| ERK | Extracellular signal-regulated kinase (MAPK) |
| EP300 | E1A binding protein p300 |
| ESR1 | Estrogen receptor 1 |
| ErbB4 | Receptor tyrosine-protein kinase erbB-4 = HER4 |
| FZD7 | Frizzed-7 (receptor for Wnt ligand) |
| GAS | G protein alpha stimulatory subunit [G alpha(s)] |
| GNAO2 | G protein alpha subunit O2 isoform |
| GPCR | G protein coupled receptor |
| GSK3β | Glycogen synthase kinase-3 beta |
| Gro | Drosophila Groucho protein |
| Gα(o) | G protein alpha (O) subunit |
| HES | Hairy and enhancer of split 1 (Transcription factor HES-1) |
| HSPA1, 5, 9 | Heat shock 70 kDa protein 1 (also HSPA1A) also 5 also 9 |
| IASPP | Inhibitor of ASPP protein (NFkB-interacting protein 1) |
| IGF (R) | Insulin-like growth factor (receptor) |
| IP3K | Inositol phosphate 3 kinase |
| IP3R | Inositol 1,4,5-trisphosphate receptor |
| ITPR3 | Type 3 inositol 1,4,5-trisphosphate receptor |
| JNK2 | c-Jun N-terminal kinase 2 |
| Kaiso | Zinc finger and BTB domain-containing protein 33 |
| MAPK | Mitogen-activated protein kinase |
| MAPK14 | mitogen-activated protein kinase 14 or p38 MAP kinase alpha |
| MCM7 | minichromosome maintenance complex component 7 |
| MEK | MAP kinase kinase (MAPKK) |
| MYC | Myc proto-oncogene protein |
| MYH9 | Myosin heavy chain 9 |
| NCOA3 | Nuclear receptor coactivator 3 = AIB1 |
| NFkB | Nuclear factor NF-kappa-B |
| Notch1 | Neurogenic locus notch homolog protein 1 |
| Notch3 | Neurogenic locus notch homolog protein 3 |
| Oct-4 | Octamer-binding transcription factor 3 (Oct-3=Oct-4) |
| PIN1 | peptidylprolyl cis/trans isomerase, NIMA-interacting 1 |
| PI3K | Phosphatidylinositol 3-kinase |
| PP2B | Protein phosphatase 2B |
| PP2C | Protein phosphatase 2C |
| PPP3CB | Serine/threonine-protein phosphatase 2B catalytic subunit beta isoform |
| PRDM5 | PR domain zinc finger protein 5 |
| PSME3 | Proteasome activator complex subunit 3 |
| p110 | PI3-kinase (p110) catalytic subunit alpha |
| p120ctn | Catenin delta-1 (p120 catenin) |
| p38 | MAP kinase p38 |
| p53 | Cellular tumor antigen p53 (Tumor suppressor p53) |
| p85 | PI3-kinase (p85) regulatory subunit alpha |
| RANBP2 | E3 SUMO-protein ligase RanBP2 (Ran-binding protein 2) |
| Rap1GAP | Rap1 GTPase-activating protein 1 |
| RAS | Family of genes encoding small GTPases |
| RSK1 / 2 | Ribosomal S6 kinase 1 / 2 |
| RUNX3 | Runt-related transcription factor 3 |
| SRC3 | Steroid receptor coactivator 3 = AIB1 |
| Sirt3 | NAD-dependent deacetylase sirtuin-3 |
| TLE3 | Transducin-like enhancer protein 3 |
| TP53 | Cellular tumor antigen p53 (Tumor suppressor p53) |
| TUBGCP2 | Tubulin gamma complex associated protein 2 |
| TYK2 | Tyrosine kinase 2 (Non-receptor type) |
| WIP1 | Wild type p53-induced protein phosphatase 1 |
| Wnt | Wnt family of ligand for frizzled receptors |
| WWP1 | WW domain-containing protein 1 |
| WWP2 | WW domain-containing protein 2 (NEDD4-like E3 ubiquitin-protein ligase) |
